# Genome-wide investigation of pentatricopeptide repeat gene family in poplar and their expression analysis in response to biotic and abiotic stresses

**DOI:** 10.1038/s41598-018-21269-1

**Published:** 2018-02-12

**Authors:** Haitao Xing, Xiaokang Fu, Chen Yang, Xiaofeng Tang, Li Guo, Chaofeng Li, Changzheng Xu, Keming Luo

**Affiliations:** 1 Key Laboratory of Eco-environments of Three Gorges Reservoir Region, Ministry of Education, Chongqing Key Laboratory of Transgenic Plant and Safety Control, Institute of Resources Botany, School of Life Sciences, Southwest University, Chongqing, 400715 China; 2 Key Laboratory of Adaptation and Evolution of Plateau Biota, Northwest Institute of Plateau Biology, Chinese Academy of Sciences, 810008 Xining, China

## Abstract

Pentatricopeptide repeat (PPR) proteins, which are characterized by tandem 30–40 amino acid sequence motifs, constitute of a large gene family in plants. Some PPR proteins have been identified to play important roles in organellar RNA metabolism and organ development in *Arabidopsis* and rice. However, functions of *PPR* genes in woody species remain largely unknown. Here, we identified and characterized a total of 626 *PPR* genes containing PPR motifs in the *Populus trichocarpa* genome. A comprehensive genome-wide analysis of the poplar *PPR* gene family was performed, including chromosomal location, phylogenetic relationships and gene duplication. Genome-wide transcriptomic analysis showed that 154 of the *PtrPPR* genes were induced by biotic and abiotic treatments, including *Marssonina brunnea*, salicylic acid (SA), methyl jasmonate (MeJA), mechanical wounding, cold and salinity stress. Quantitative RT-PCR analysis further investigated the expression profiles of 11 *PtrPPR* genes under different stresses. Our results contribute to a comprehensive understanding the roles of PPR proteins and provided an insight for improving the stress tolerance in poplar.

## Introduction

The pentatricopeptide repeat (PPR) proteins are characterized by an assembly of 2 to about 30 degenerate tandem repeats of approximately 35 amino acid residues that mediate their RNA-binding activities^1,2^. Typically, PPR proteins can be classified into two major subfamilies (P and PLS) according to the nature of their PPR motifs. The P subfamily proteins with orthodox 35 amino acid PPR motifs participate in a wide range of organelle RNA-processing activities, including determination and stabilization of 50 and/or 30 termini, RNA splicing and translation initiation^3^. Another PLS subfamily of PPR proteins, which is specific to land plants, generally contain the P motif and two P motif-derived variants, including the short (S) and the long (L) motifs^1^. Most of PLS subfamily members also possess additional C-terminal domains, termed the E, E+ and DYW domains^1,4^. It has been shown that these C-terminal domains are required for the RNA-editing activity in plant organelles that appears to be the primary function of many PLS subfamily proteins^4–10^.

Several previous studies have shown that PPR domain-containing proteins are involved in the regulation of plant growth and development. In *Arabidopsis*, for instance, *EMB175* is targeted to the plastid and essential for plant embryogenesis and mutation in *EMB175* caused defects in the rate of cell division^11^. Prasad *et al*.^12^ showed that Lateral Organ Junction (LOJ) protein gene, encoding a PPR protein was expressed specifically at the lateral organ junctions and meristematic regions in *Arabidopsis*, indicating its involvement in shoot apical meristem development. In petunia, a mitochondrial-targeted protein *Rf*-PPR592 can restore fertility to *rf/rf* cytoplasmic male sterility (CMS) lines^13^. *Rfo* (fertility restorer) and ORF68 containing multiple PPRs are able to restore fertility Ogura (*ogu*) CMS in *Brassica napus*^14–16^.

Recently, increasing molecular evidences have identified that several PPRs play important roles in various biotic and abiotic stresses. In *Arabidopsis*, genomes uncoupled 1 (GUN1) is implicated with plastid-to-nucleus retrograde signaling, regulation of *ABI4* expression and photooxidative stress responses^17^. It is established that PPR40 provides a signaling link between mitochondrial electron transport in *Arabidopsis*. Knock-out of *PPR40* resulted in enhanced accumulation of reactive oxygen species (ROS), increased in lipid peroxidation and superoxide dismutase activity^18^. ABA overly sensitive 5 (ABO5/At1g51965), encoding a PPR protein, is important for the splicing of NADH dehydrogenase subunit 2 (*NAD*2) intron 3 in mitochondria. The *abo5* mutant accumulated higher H_2_O_2_ content in roots than the wild type^19^. MITOCHONDRIAL RNA EDITING FACTOR 11 (MEF11)/LOVASTATIN INSENSITIVE 1 (LOI1) is involved in mitochondrial RNA editing, and regulates biosynthesis of isoprenoids, which are known to affect defense gene expression in response to wounding and pathogen infection^20,21^. *Hypersensitive Germination* 11 (AHG11) might be involved in RNA editing on mitochondrial protein gene *NAD4*. The *ahg11* mutants exhibited higher transcript levels of oxidative stress responsive genes^22^. PENTATRICOPEPTIDE REPEAT PROTEIN FOR GERMINATION ON NaCl (PGN) has been demonstrated to be involved in the response to biotic and abiotic stresses^23^. The *Arabidopsis* mutant of Slow Growth 1 (*slg1*) affected shoot growth and negatively regulated drought stress and ABA signaling^24^. Another PPR protein SLO2 was also identified to regulate plant growth. The *slo2* mutant exhibits elevated transcript levels of stress responsive genes. Additionally, the *slo2* mutant displays hypersensitivity to osmotic and ABA stresses during different seed germination stages, whereas their adult plants exhibit enchance tolerance to salt and drought stresses^25–27^. The *Arabidopsis* SVR7 (SUPPRESSOR OF VARIEGATION 7) is required for the translation of chloroplast ATP synthase subunits.Knock-out of *SVR7* resulted in higher levels of ROS accumulation, elevated sensitivity to H_2_O_2_ and reduced photosynthetic activity^28^. Recently, SOAR1 (suppressor of the *ABAR*-overexpressor 1), encoding a nucleo-cytoplasmic localized PPR protein, was identified as a positive regulator of various stresses, such as drought, salt and cold^29^. Liu *et al*.^30^ also reported that expression of *PPR96* gene was induced by salt, oxidative and ABA stresses in *Arabidopsis*.

Trees are major renewable resources which fulfill our requirements for wood materials and bioenergy and also provide environmental benefits such as carbon sequestration^31,32^. With the complete of the genome sequencing, *Populus trichocarpa* has been considered as an ideal model species for genomic and genetic studies of woody plants. To date, however, little information about PPR proteins relating to biotic and abiotic stress response mechanisms was reported in poplar. In this study, we predicted 626 putative *PPR* genes from the *P*. *trichocarpa* genome. A comprehensive analysis of the poplar PPR superfamily, including phylogenetic tree, chromosomal localization, gene structure, was performed. To investigate the potential functions of these genes, the expression profiles of *PtrPPR* genes under biotic and abiotic stresses were determined by RNA-sequencing analysis and quantitative RT-PCR. Our results provide an insight on the molecular mechanisms of these *PtrPPR* genes in response to environmental stresses in poplar.

## Results

### Genome-wide identification and classification of *PPR* genes in *P*. *trichocarpa*

**Figure 1:**
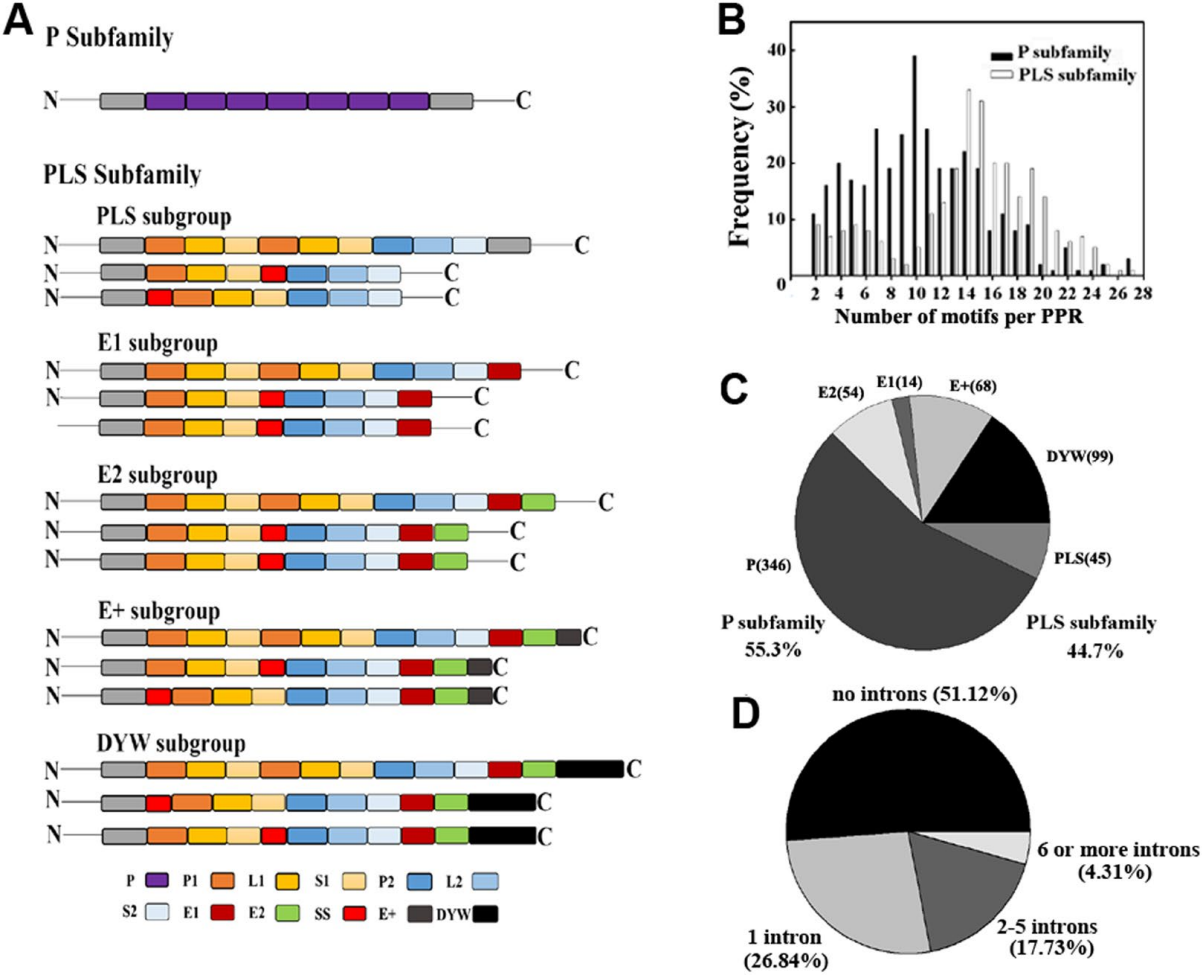
Number and structure of poplar PPR proteins. (**A**) Basic motif architecture of PPR proteins from subfamily and subgroup are shown. (**B**) Frequency of the number of PPR motifs per protein. (**C**) Numbers of PtrPPR proteins belonging to different subfamilies and subgroups. (**D**) Numbers of introns in *PtrPPR* genes.

We first performed genome-wide search of putative *PPR* genes in *P*. *trichocarpa* genome and all of the 626 putative genes encoding PPR proteins were predicted in this study. These poplar *PPRs* genes were designated PtrPPR1 to PtrPPR626 in the order of their access number in the Phytozome database (https://phytozome.jgi.doe.gov/pz/portal.html). The Phytozome locus, chromosomal location, sub-group, number of exons, motif structures, open reading frame (ORF) length, predicted protein length are listed in Supplemental Table 1. Based on the structure of the repeated motifs, the *PPR* gene family can be split into P and PLS subfamilies. As shown in Fig. 1A, 55.30% (346 of 626) and 44.70% (280 of 486) of *PPR* genes were classified into P subfamily and PLS subfamily, respectively. PPR proteins are characterized by the tandem array of PPR motifs. In *P*. *trichocarpa*, the number of PPR motifs per protein varies from 2 to 27, but there are strong peaks in the distribution at around 9–12 PPR motifs in P-class proteins and 13–16 motifs in PLS-class proteins (Fig. 1B). On the basis of the presence of the C-terminal conserved domains, the PLS subfamily members are further divided into five subgroups: PLS, E1, E2, E+ and DYW subgroups^1^. These subgroups of the poplar PLS subfamily contain 45, 14, 54, 68 and 99 members, respectively (Fig. 1A and B).

Previous studies have showed that the main majority of the *PPR* genes in different plant species with sequenced genomes is found to be lacking or containing few introns^2,4,32^. The exon-intron structures of *PtrPPR* genes were also determined. As shown in Fig. 1D, 51.12% (320/626) of the *PtrPPR* genes were predicted to lack introns, 26.84% (168/626) with 1 intron, 17.73% (111/626) with 2–5 introns, and 4.31% (27/626) with 6 or more introns.

### Chromosomal distribution and phylogenetic analysis

**Figure 2:**
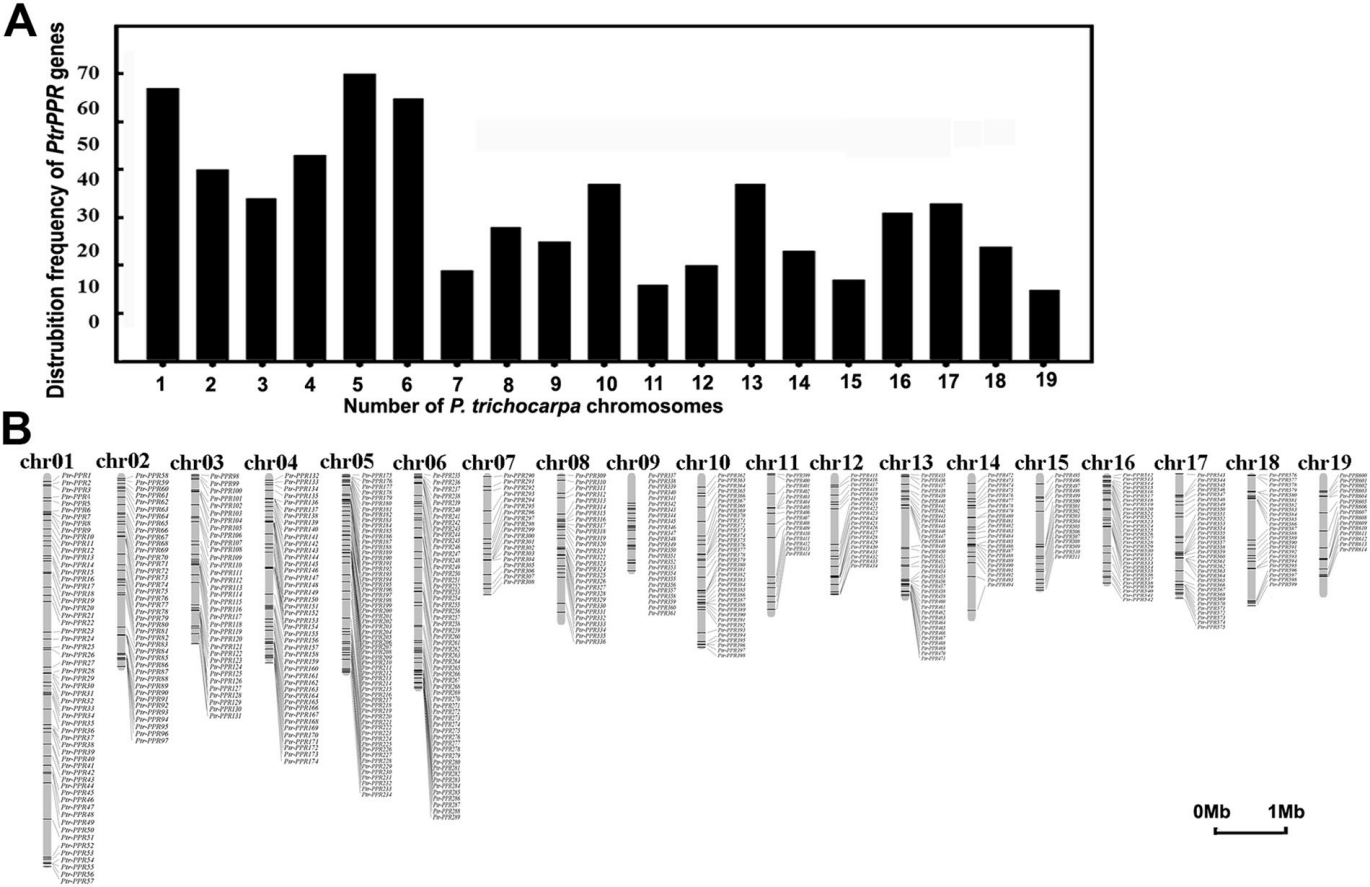
Chromosomal distributions of the *PtrPPR* genes. (**A**) Distribution frequency of the *PtrPPR* genes on poplar chromosomes is indicated. (**B**) Distributions of the *PtrPPR* genes on the scaffolds (chr 1 to chr 19) of *P*. *trichocarpa*.

To determine the genomic distribution of these *PtrPPR* genes on the *P*. *trichocarpa* genome, we downloaded their chromosome location information from the phytozome database and identified their position. The 614 *PtrPPR* genes were mapped unevenly to the 19 chromosomes (Fig. 2A), while the remaining 12 members were not mapped to specific chromosome due to their localization on isolated scaffolds (Supplemental Table 1). Chromosome 5 had the largest number (60) of *PtrPPR* genes, while the smallest number (only 15) of *PtrPPRs* was found on chromosome 19. Furthermore, the detailed position of each *PtrPPRs* on the poplar chromosomes was obtained from Phytozome (Fig. 2B). Obviously, these genes were described in clusters or individually. Substantial clustering of *PtrPPR* genes was evident on all of the chromosomes, indicating a clue to their evolution.

**Figure 3:**
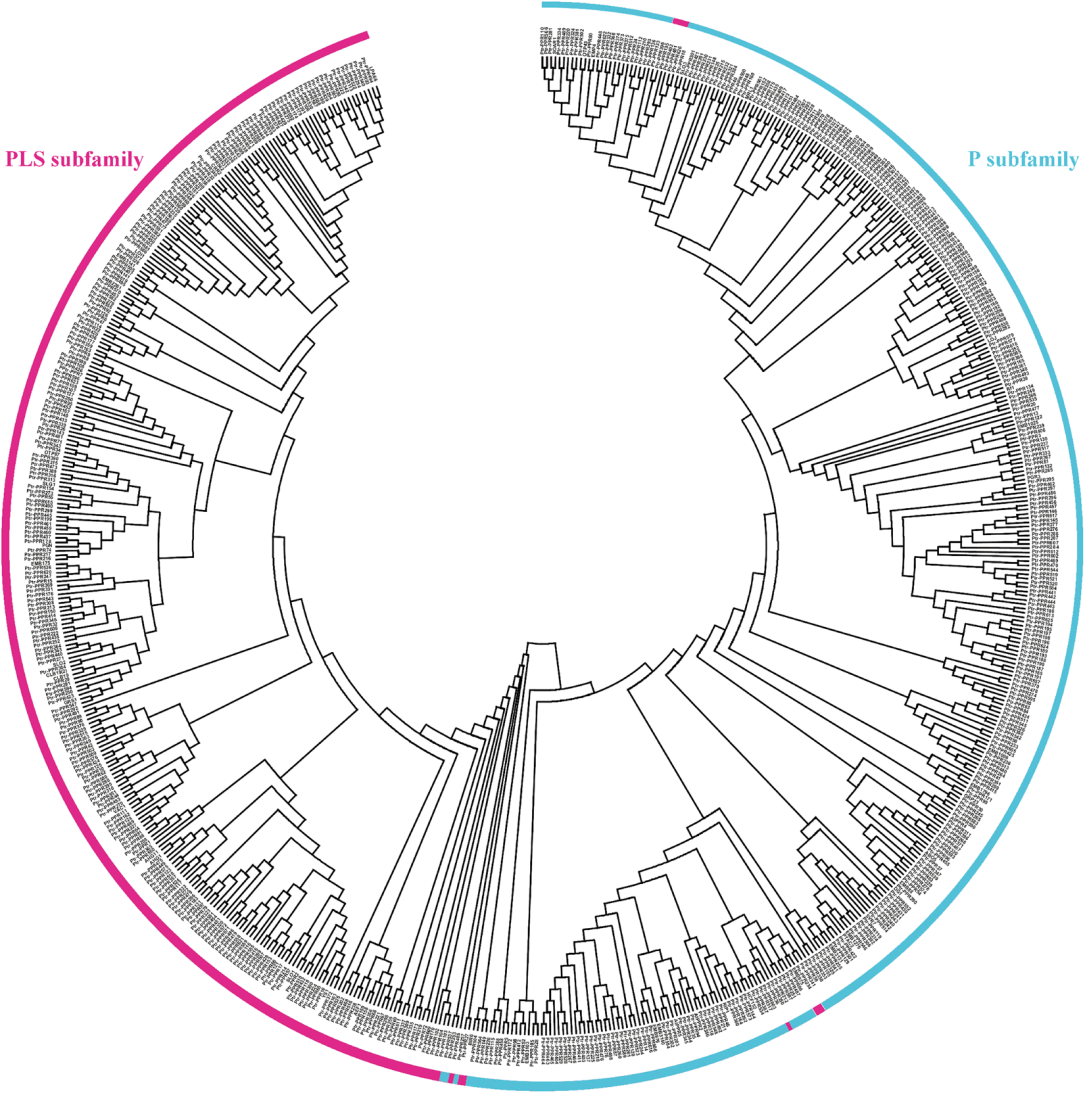
Phylogentic trees of the *PtrPPR* family genes. The complete amino acid sequences of 626 PtrPPR proteins and other 48 PPR proteins from rice,maize and rice were aligned by MUSCLE, and the Maximum-likelihood tree was constructed using MEGA7.0 with 1000 bootstrap replicates. P and PLS subfamilies are highlighted in different colors.

To further gain insights into the evolutionary relationship among these members of PtrPPR family, the phylogenetic tree was constructed using Maximum likelihood based on the full-length amino acid sequences of 626 PtrPPR proteins, 48 PPRs from *Arabidopsis*, maize (*Zea mays*) and rice (Supplemental Table 1). These *PtrPPR* genes were classified into two distinct subfamilies (P subfamily, PLS subfamily) (Fig. 3). Interestingly, in the phylogenetic tree, these members, including PtrPPR135, PtrPPR136, PtrPPR402, PtrPPR401, PtrPPR626, PtrPPR487 and PtrPPR86 were clustered into the P subfamily, but they possessed the structure of the repeated motifs of PLS subfamily members.

### Predication of MicroRNA target sites in *PtrPPR* genes

Previous studies have shown that miRNAs have perfect/near-perfect complementarity to their target genes, allowing an effective prediction of the target sequences through computational analysis^33^. Lu *et al*.^34^ reported that at least 7 *PtrPPR* genes contain complementary sites of PtrmiR474, PtrmiR475 and PtrmiR476 in poplar. In this study, we further searched the MircroRNA target sites in all *PtrPPR* genes in *P*. *trichocarpa*. The results showed that 279 putative MircroRNA target sites were predicted in 100 *PtrPPR* genes (Supplemental Table 1). These MircroRNAs included PtrmiR156, PtrmiR396, PtrmiR472, PtrmiR474, PtrmiR475, PtrmiR476, PtrmiR477, PtrmiR482, PtrmiR6421, PtrmiR6423, PtrmiR6425, PtrmiR6428, PtrmiR6445, PtrmiR6450, PtrmiR6466, PtrmiR6470, PtrmiR6480, PtrmiR7814, PtrmiR7816, PtrmiR7817, PtrmiR7823 and PtrmiR7826. Among these putative target sites, interestingly, 218 sites were predicted to be complementary to PtrmiR475 and PtrmiR476, which were species-specific MircroRNAs in poplar.

### Expression profiling of *PtrPPR* genes under different stresses

**Figure 4:**
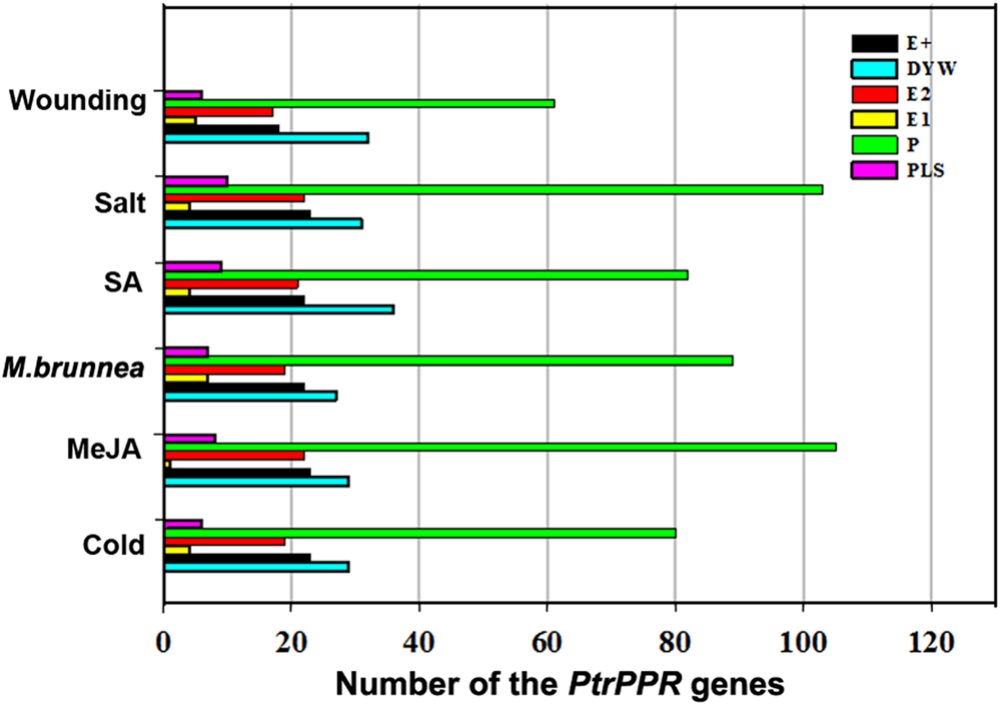
Determination of the expression of the *PtrPPR* genes in response to different stresses by transcriptomic analysis.

Previously, many PPR proteins have been identified to function in post-transcriptional and post-translational processes in response to biotic and abiotic stresses^20–24^. To determine their potential roles to respond to various environmental stresses, we investigated the expression profiles of *PtrPPR* genes in different induction treatments by using high-throughput sequencing analysis. Total RNA was extracted from the poplar leaves which were treated with salicylic acid (SA), methyl jasmonate (MeJA), *Marssonina brunnea*, mechanical wounding, low temperature, and salinity, respectively. Transcriptomic analysis showed that the *PtrPPR* genes had a variety of distinct expression profiles after different treatments (Supplemental Table 1). Among the *PtrPPR* genes, 122 members were activated by cold treatment but the expression levels of 50 members were down-regulated at least 2-fold (Fig. 4). Only 67 genes were induced by *M*. *brunnea* infection and meanwhile 104 genes were repressed, whereas other *PtrPPR* genes were not affected. More than 150 *PtrPPR* genes responded to MeJA signaling and most of them were also upregualted after GA treatment. In addition, the expression levels of a number of *PtrPPR* genes were increased significantly after exposure to salt stress. In contrast, relatively few *PtrPPR* genes were induced by wounding treatment (Fig. 4, Supplemental Table 1). Among these *PtrPPR* genes in response to different stresses, most of them belong to P subgroup while only a few members belong to the E+ subgroup. As shown in Fig. 4, only 4, 1, 7, 4, 4, 5 genes of the E+ subgroup responded to cold, MeJA, *Marssonina brunnea*, SA, salt and wound treatments, respectively.

**Figure 5:**
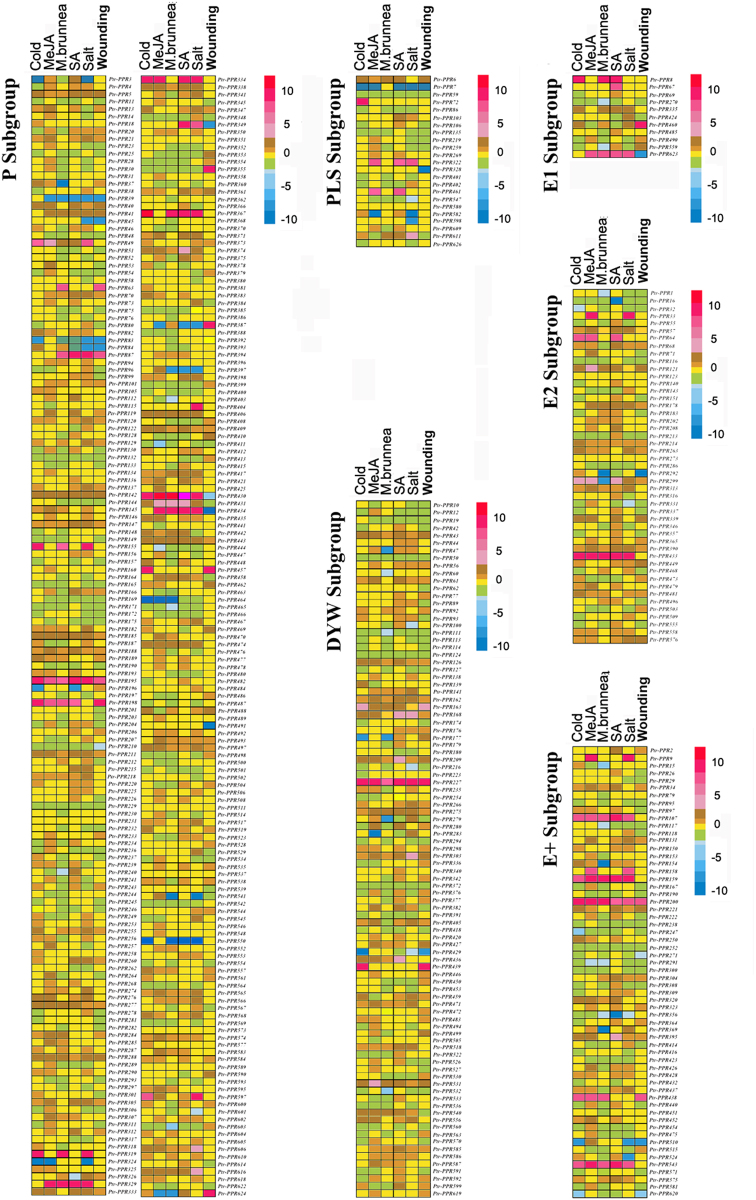
Expression profiles of the *PtrPPR* genes under different stresses. Heatmap shows different expression levels of the *PtrPPR* genes in response to various stress conditions. Color scale erected vertically at the right side of the picture represents expression values. Blue represents low level and red indicates high level of transcript abundances.

Finally, the heatmap was produced based on the trancriptomic data of the *PtrPPR* genes under various environmental stresses (Fig. 5). Interestingly, the expression levels of certain *PtrPPR* genes, such as *PtrPPR193*, *PtrPPR198*, *PtrPPR200*, *PtrPPR227*, *PtrPPR433*, *PtrPPR439*, *PtrPPR543*, were upregulated following various treatments, indicating that these *PtrPPR* genes might play multiple roles in different physiological processes in poplar.

### Verification of RNA-seq data by using quantitative RT-PCR

**Figure 6:**
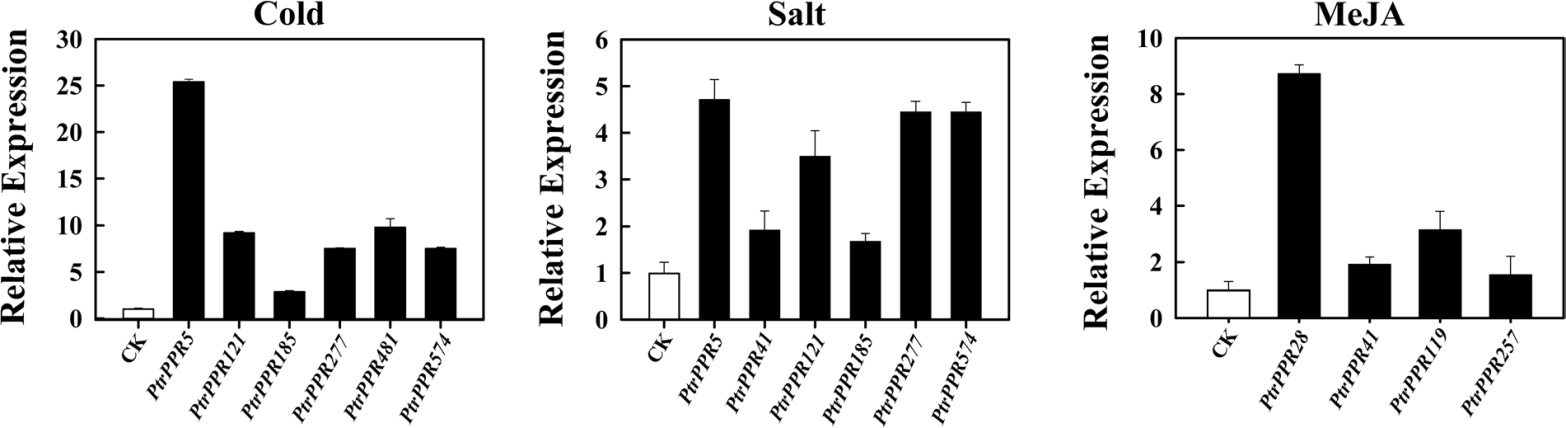
Expression patterns of 11 selected *PtrPPR* genes under cold, salt and MeJA treatments as revealed by qRT-PCR. The poplar 18 S *rRNA* gene was used as an internal control. Error bars, 3 ± SE.

Quantitative real-time PCR (qRT-PCR) was performed to verify the transcriptomic data by using Takara TP800 Real-Time PCR system. *P*. *trichocarpa* were subjected to different stresses, including cold, MeJA and salinity. The untreated plants were used as controls. We determined expression levels of 11 *PtrPPR* genes, including *PtrPPR5*, *PtrPPR8*, *PtrPPR30*, *PtrPPR41*, *PtrPPR119*, *PtrPPR121*, *PtrPPR185*, *PtrPPR257*, *PtrPPR277*, *PtrPPR481* and *PtrPPR574*, in response to different treatments. The specific primer sequences used in qRT-PCR are shown in Supplemental Table 5. As shown in Fig. 6, 7 (*PtrPPR5*, *PtrPPR41*, *PtrPPR121*, *PtrPPR185*, *PtrPPR277*, *PtrPPR481* and *PtrPPR574*) of 11 *PtrPPR* genes were distinctly induced and 3 genes (*PtrPPR8*, *PtrPPR30*, *PtrPPR119*) were downregulated in response to salt treatment. For cold treatment, 7 genes (*PtrPPR5*, *PtrPPR8*, *PtrPPR121*, *PtrPPR185*, *PtrPPR277*, *PtrPPR481* and *PtrPPR574*) were obviously upregulated, whereas the expression of *PtrPPR30* and *PtrPPR257* changed only slightly. In addition, *PtrPPR41* and *PtrPPR119* were dramatically repressed by salt stress but induced under MeJA treatment (Fig. 6). These results are consistent with the transcriptomic data above.

## Discussion

Poplar is one of the most important tree species for large-scale forestation in temperate latitudes. Meanwhile, because of its relatively compact genetic complement (approximately 480 Mbp), poplar is also considered as an ideal model system for tree studying. The PPR proteins represent one of the largest families in land plants, but the function of most of PPR proteins remain unclear, especially in woody species. In this study, 626 PPR proteins, of which 346 members belong to the P subfamily and 280 ones belong to the PLS subfamily, were identified in *P*. *trichocarpa* (Supplemental Table 1). Compared with the 450 and 477 PPR genes in *Arabidopsis* and in rice (*O*. *sativa*)^32^, respectively, the family has greatly expended in poplar, implying that the *P*. *trichocarpa* PPR proteins might possess more diversified functions than herbaceous plants.

MicroRNAs exert their roles by targeting mRNAs for cleavage or translational repression^35–37^. The target mRNAs of microRNAs involved in plant defense responses have been increasingly identified. For instance, Young *et al*.^38^ described that miR400-guided cleavage of *PPR1* and *PPR2* renders *Arabidopsis* more susceptible to pathogenic bacteria and fungi. In poplar, a previous study has shown that seven *PtrPPR* genes have complementary sites of PtrmiR474, PtrmiR475 and PtrmiR476^34^. In this study, we had further predicted the complementary sites in all *PtrPPR* genes targeted by miRNAs in *P*. *trichocarpa*. At least 279 target sites were found in 100 *PtrPPR* genes (Supplemental Table 1). Among these target sites, interestingly, most (218) of the complementary sites were predicted to be targeted by PtrmiR475 and PtrmiR476, which were speices-specific in poplar (Supplemental Table 1). This finding suggests that PtrmiR475 and PtrmiR476 might be involved in the regulation of the expression of *PtrPPR* genes in poplar.

Previous studies showed that these PPR proteins characterized in *Arabidopsis*, rice and maize (*Z*. *mays*) are mainly involved in the regulation of various post-transcriptional processes related to gene expression in plant organelles^1–3^. Only a few of PPR proteins were reported to play roles in biotic and abiotic stresses response in higher plants. For example, *Arabidopsis* GUN1 is implicated with the regulation of *ABI4* expression and photooxidative stress responses^17^. MEF11/LOI1 is associated with mitochondrial RNA editing, and regulates biosynthesis of isoprenoids, which are known to affect defense gene expression in response to wounding and pathogen infection^20,21^. PPR40 is involved in oxidative respiration that contributes to abiotic stress tolerance in *Arabidopsis* and its mutant *ppr40* displays enhanced sensitivity to ABA and salinity that correlates with elevated accumulation ROS^18^. Another PPR gene *PGN* is associated with the regulation of mitochondria-nucleus retrograde signaling, affecting ROS homeostasis by modulating RNA editing in mitochodria during biotic and abiotic stress responses^23^. AHG11 regulates the *nad4* (mitochondrion complex I) transcriptional level and changes in oxidative levels by controlling RNA editing events in mitochondria, resulting in affecting plant responses to ABA^22^. Similarly, SLG1 participates in the regulation of the *nad3* transcriptional level by modulating RNA editing events in mitochondria and influencing the expression of genes involved in the alternative respiratory pathway^24^. However, little is known about the physiological and molecular mechanism that PPR proteins are involved in RNA editing in plastids or mitochondria under different stresses. In this study, we explored the expression patterns of all *PtrPPR* genes under different stresses, including *M*. *brunnea*-infected, SA, MeJA, wounding, cold and salinity stresses by RNA-sequencing analyses. The results showed that many *PtrPPR* genes were responsive to abiotic and biotic stresses (Fig. 5). Furthermore, quantitative RT-PCR also confirmed that 11 *PtrPPR* genes were upregulated or downregulated after cold, MeJA and salinity treatments (Fig. 6). These results suggest that *PtrPPR* genes may play roles in environmental adaptation in poplar.

In conclusion, a total of 626 PPR protein genes were identified in *P*. *trichocarpa* genome. The classification of these genes by PPR motif type and expression pattern in abiotic and abiotic treatments were performed and provides valuable information for future studies on characterizing the biological functions of PPR protein genes in *P*. *trichocarpa*. Our study provides some useful information for comparative analyses of the *PtrPPR* gene family, but additional physiological and biochemical experiments still need to be performed to further determine the detailed functions of these *PtrPPR* genes in the future.

## Materials and Methods

### Poplar growth conditions and stress treatments

*Populus trichocarpa* Torr. & A. Gray was grown in a plant growth chamber at 25 °C under a 14/10 h light/dark cycle. Five-old-month poplar plants were used for various treatments as previously described^39,40^.

### Prediction of *PPR* genes in *P*. *trichocarpa*

We analyzed *P*. *trichocarpa* genome using profile hidden Markov models (HMMs) for PPR motifs generated by Yin *et al*.^41^. The P motifs are usually present in PPR proteins as tandem arrays of a dozen repeats. These sequences of PtrPPR proteins were then used for HMM construction by HMMBUILD from the HMMER3 package^53^. The models constructed by HMMBUILD were used to direct HMMSEARCH, using a relatively stringent threshold for retaining PPR proteins. Finally, 626 nonredundant PPR proteins in *P*. *trichocarpa*, exhibited the presence of PPR motif with confidence (E-value < 0.1) in SMART (http://smart.embl-heidelberg.de/). In addition, The HMMsearch program from the HMMER package^42^ was applied to the translated sequence data to identify clusters of all of the PPR motifs, including P, L, S, L2, E/E+, and DYW. We further predicted the C-terminal domains including E and DYW after the tandem arrays of P motifs.

### Chromosome location and gene structure analysis

Positional information of *PtrPPR* genes on chromosomes of *P*. *trichocarpa* was obtained from the Phytozome database (http://www.icugi.org/Phytozome). All *PtrPPR* genes were mapped proportionally to 19 chromosomes of *P*. *trichocarpa*.

The gene structures of the *PtrPPR* genes were parsed from the General Feature Format (GFF) files. The numbers of introns and exons were detected by comparing the full-length cDNA of the putative *PtrPPR* genes with their corresponding genomic sequences in *P*. *trichocarpa*.

### Phylogenetic analysis

Physical multiple sequence alignment of 626 PPR proteins from *P*. *trichocarpa* and 48 PPR proteins from other species including *Arabidopsis*, maize and rice was conducted by using the MUSCLE method. A phylogenetic tree was constructed by using the Maximum- likelihood method with MEGA7 (http://www.megasoftware.net/mega.html)^43^ software and bootstrap analysis of 1,000 replicates.

### Transcriptomic analysis

Briefly, total RNA was isolated using TRIzol reagent (Invitrogen) from the third-fifth leaves of poplar plants after various treatments and then treated with RNase free DNase I (Takara, Dalian, China) according to the manufacturer’s recommendations. RNA-seq analysis was completed by Huada Genomics Institute (BGI) (Shenzhen, China) and detailed description according to the previous description^39^.

As previously described^39^, the information of the *P*. *trichocarpa* genome and annotated gene set were downloaded from the DOE Joint Genome Institute website (http://genome.jgi-psf.org/cgi-bin/). Transcriptomic analysis was performed as described in our previous report^39^. The obtained reads were aligned to the *P*. *trichocarpa* genome using SOAPDENOVO2^43^. All data of transcriptomic analysis was deposited in GEO (accession number: GSE109609).

### Quantitative RT-PCR analysis

qRT-PCR analysis was conducted to detect the expression profiles of 11 representative *PtrPPR* genes after cold, salt and MeJA treatments. The third-fifth leaves of poplar plants were collected after various treatments. Total RNA was extracted from plant samples using TRIzol reagent (Invitrogen), which was reverse transcribed into cDNA subsequently using a PrimeScript™ RT Reagent Kit (Takara, Dalian, China). The gene-specific primer sequences used for qRT-PCR are shown in Supplemental Table 1. qRT-PCR analysis was performed three biological replicates for each sample.

## Electronic supplementary material

Supplementary data
