# Supplementary material for: Genome-wide investigation of pentatricopeptide repeat gene family in poplar and their expression analysis in response to biotic and abiotic stresses
Source: Sci Rep. 2018 Feb 12;8:2817. doi: 10.1038/s41598-018-21269-1 (PMC5809412; doi:10.1038/s41598-018-21269-1)
Supplement: Supplementary file 1 — Supplementary data [file 41598_2018_21269_MOESM1_ESM.docx]

**TITLE: Genome-wide investigation of pentatricopeptide repeat gene family in poplar and their expression analysis in response to biotic and abiotic stresses**

**Author**: Haitao Xing, Xiaokang Fu, Chen Yang, Xiaofeng Tang, Li Guo, Chaofeng Li, Changzheng Xu, Keming Luo

| **Supplemental Table 1. The members of PtrPPR gene family in poplar** | | | | | | | | | | | | | |
| --- | --- | --- | --- | --- | --- | --- | --- | --- | --- | --- | --- | --- | --- |
| **Gene name** | **Gene ID V3.0** | **Gene ID V2.0** | **Chromosome** | **Strand** | **Start** | **End** | **No. of exons** | **Exon structure** | **Exon region** | **amino acid length** | **PPR type** | **No. of motifs** | **Motif structure** |
| PtrPPR1 | Potri.001G014900.1 | POPTR_0001s05260.1 | Chr01 | + | 4026583 | 4028836 | 3 | exon-exon-exon | 4026583_4028275-4028499_4028521-4028753_4028836 | 599 | E2 | 15 | 23-L1-S1-P1-L1-S1-P1-P1-P1-L1-S1-P2-L2-S2-E1i-E2-64 |
| PtrPPR2 | Potri.001G019500.1 | POPTR_0001s05700.1 | Chr01 | + | 1418782 | 1420964 | 1 | exon | (1418834-1420840) | 668 | E+ | 16 | 117-P1-L1-S1-P1-L1-S1-SS-P1-L1-S1-P2-L2-S2-E1-E2-E+ |
| PtrPPR3 | Potri.001G020500.1 | POPTR_0001s05780.1 | Chr01 | + | 1562893 | 1563722 | 2 | exon-exon | (1562893-1563203)(1563254-1563722) | 260 | P | 7 | 41-P-P-P-P-P-P-P |
| PtrPPR4 | Potri.001G043500.1 | POPTR_0001s03510.1 | Chr01 | + | 3150482 | 3153589 | 2 | exon-exon | (3150517-3151106)(3151402-3152440) | 542 | P | 9 | 187-P-P-Pi-P-P-P-Pi-P-P-44 |
| PtrPPR5 | Potri.001G047200.1 | POPTR_0001s14810.1 | Chr01 | - | 3504695 | 3512199 | 14 | exon-exon-exon-exon-exon-exon-exon-exon-exon-exon-exon-exon-exon-exon | (3504695-3504727)(3504827-3504878)(3504982-3505039)(3505120-3505313)(3505796-3505903)(3506184-3506425)(3506601-3507289)(3507792-3507884)(3507995-3508168)(3508582-3509109)(3509731-3509874)(3509980-3510168)(3511122-3511241)(3511399-3511803) | 869 | P | 9 | 129-P-P-193-P-66-P-48-P-P-P-P-P-113 |
| PtrPPR6 | Potri.001G062600.1 | POPTR_0001s13230.1 | Chr01 | + | 4850887 | 4853034 | 8 | exon-exon-exon-exon-exon-exon-exon-exon | (4850887-4851120)(4851177-4851414)(4851461-4851724)(4851780-4851988)(4852024-4852344)(4852570-4852678)(4852717-4852921)(4852962-4853035) | 550 | PLS | 7 | SS-P1-43-P1-L1-155-S1-P2-L2-133 |
| PtrPPR7 | Potri.001G063800.1 | POPTR_0008s18580.1 | Chr01 | + | 5034185 | 5034866 | 3 | exon-exon-exon | (5034185-5034276)(5034395-5034675)(5034717-5034866) | 172 | PLS | 2 | 85-P1-18-S1-3 |
| PtrPPR8 | Potri.001G064300.1 | POPTR_0008s18520.1 | Chr01 | + | 5071762 | 5073601 | 6 | exon-exon-exon-exon-exon-exon | (5071762-5071931)(5072013-5072499)(5072606-5072655)(5072813-5073348)(5073193-5073348)(5073438-5073601) | 446 | E1 | 12 | 5-S1-P1-S1-P1-L1-S1-P1-L1-S1-P2-16-S2-E1-42 |
| PtrPPR9 | Potri.001G065200.1 | POPTR_0008s18430.1 | Chr01 | + | 5163758 | 5164215 | 2 | exon-exon | (5163758-5163950)(5164031-5164215) | 125 | E+ | 2 | 10-S2-34-E+ |
| PtrPPR10 | Potri.001G066100.1 | POPTR_0001s13120.1 | Chr01 | - | 5240525 | 5242957 | 1 | exon | 5240525_5242831 | 768 | DYW | 17 | 93-L1-S1-P1-L1-S1-P1-L1-S1-P1-L1-S1-P2-L2-S2-E1-E2-DYW |
| PtrPPR11 | Potri.001G075700.1 | POPTR_0001s12210.1 | Chr01 | + | 6018502 | 6022126 | 1 | exon | (6018505-6019283) | 258 | P | 2 | 131-P-P-55 |
| PtrPPR12 | Potri.001G075800.1 | POPTR_0001s12200.1 | Chr01 | - | 6025447 | 6028170 | 1 | exon | (6025520-6028170) | 883 | DYW | 21 | 43-P1-L1-S1-P1-L1-S1-P1-L1-S1-P1-L1-S1-P1-16-L1-S1-P2-L2-S2-E1-E2-DYW |
| PtrPPR13 | Potri.001G075900.1 | POPTR_0001s12190.1 | Chr01 | + | 6031982 | 6035109 | 1 | exon | (6032021-6034033) | 670 | P | 14 | 89-P-P-P-P-P-P-P-P-P-P-P-P-P-P-85 |
| PtrPPR14 | Potri.001G090800.1 | POPTR_0001s10690.1 | Chr01 | - | 7147077 | 7151245 | 12 | exon-exon-exon-exon-exon-exon-exon-exon-exon-exon-exon-exon | (7147119-7147581)(7147665-7147819)(7147907-7147984)(7148123-7148202)(7148413-7148497)(7149155-7149574)(7149937-7150044)(7150214-7150281)(7150388-7150475)(7150625-7150696)(7150780-7150848)(7151166-7151245) | 588 | P | 2 | 120-P-13-P-385 |
| PtrPPR15 | Potri.001G100600.1 | POPTR_0001s09740.1 | Chr01 | - | 7961657 | 7964452 | 1 | exon | 7961657_7964116 | 819 | E+ | 20 | 56-S1-P1-L1-S1-P1-34-S1-32-L1-S1-P1-L1-S1-P1-L1-S1-P2-L2-S2-E1-E2-E+ |
| PtrPPR16 | Potri.001G108500.1 | POPTR_0001s08940.1 | Chr01 | - | 8624936 | 8626669 | 2 | exon-exon | (8624936-8625683)(8625900-8626669) | 505 | E2 | 13 | 21-L1-S1-P1-L1-S1-SS-SS-31-SS-P2-L2-S2-E1-E2-16 |
| PtrPPR17 | Potri.001G116300.1 | POPTR_0001s08060.1 | Chr01 | - | 9319777 | 9321395 | 6 | exon-exon-exon-exon-exon-exon | (9319777-9320028)(9320119-9320337)(9320393-9320493)(9320556-9320647)(9320821-9321011)(9321123-9321395) | 375 | E2 | 9 | 85-S1-SS-SS-SS-P2-L2-S2-E1-E2-5 |
| PtrPPR18 | Potri.001G130400.1 | POPTR_0001s01250.1 | Chr01 | + | 10522239 | 10525018 | 1 | exon | (10522462-10524000) | 512 | P | 10 | 109-P-P-P-P-P-P-P-P-P-P-50 |
| PtrPPR19 | Potri.001G135400.1 | POPTR_0001s01780.1 | Chr01 | + | 10867432 | 10869703 | 1 | exon | (10867837-10869645 | 602 | DYW | 13 | 61-SS-P1-L1-S1-P1-L1-S1-P2-L2-S2-E1-E2-DYW |
| PtrPPR20 | Potri.001G139200.1 | POPTR_0001s02160.1 | Chr01 | - | 11164891 | 11168275 | 1 | exon | (11164917-11167397) | 826 | P | 19 | 98-P-P-P-P-P-P-P-P-P-P-P-P-P-P-P-P-P-14-P-P-48 |
| PtrPPR21 | Potri.001G139300.1 | POPTR_0001s02170.1 | Chr01 | - | 11171351 | 11174450 | 1 | exon | (11171654-11173939) | 761 | P | 16 | 178-P-P-P-P-P-P-P-P-P-P-P-P-P-P-P-P-17 |
| PtrPPR22 | Potri.001G152900.1 | POPTR_0001s15285.1 | Chr01 | - | 12613531 | 12614143 | 2 | exon-exon | (12613531-12613891)(12613935-12614143) | 189 | PLS | 5 | P1-L1-S1-P2-L2-53 |
| PtrPPR23 | Potri.001G154900.1 | POPTR_0001s15470.1 | Chr01 | - | 12788458 | 12790049 | 1 | exon | (12788458-12789889) | 476 | P | 9 | 129-P-P-P-P-P-P-P-P-P-28 |
| PtrPPR24 | Potri.001G171300.1 | scaffold_1:F:13998792-13999994 | Chr01 | + | 14537548 | 14538983 | 1 | exon | (14537779-14538983) | 401 | P | 7 | 106-P-36-P-P-P-P-P-P-14 |
| PtrPPR25 | Potri.001G180000.1 | POPTR_0001s18030.1 | Chr01 | - | 15572703 | 15575173 | 2 | exon-exon | (15572703-15572827)(15572938-15574784) | 656 | P | 13 | 141-P-P-P-P-34-P-P-P-P-P-P-P-P-P-23 |
| PtrPPR26 | Potri.001G186500.1 | POPTR_0001s18710.1 | Chr01 | - | 16435289 | 16439069 | 1 | exon | 16435842_16437725 | 627 | E+ | 18 | 3-L1-S1-P1-L1-S1-31-SS-P1-L1-S1-P1-L1-S1-P2-L2-S2-E1-E2-E+ |
| PtrPPR27 | Potri.001G204500.1 |  | Chr01 | - | 20332606 | 20333035 | 1 | exon | (20332766-20333023) | 85 | PLS | 4 | SS-SS-P2-L2 |
| PtrPPR28 | Potri.001G211300.1 | POPTR_0001s21880.1 | Chr01 | + | 21183482 | 21192595 | 10 | exon-exon-exon-exon-exon-exon-exon-exon-exon-exon | (21183499-21183821)(21184432-21184516)(21186108-21186371)(21187053-21187355)(21188047-21188151)(21188359-21188458)(21188085-21189404)(21189698-21189874)(21189954-21190082)(21192061-21192276) | 673 | P | 11 | 114-P-Pi-P-Pi-P-P-P-P-P-P-P-174 |
| PtrPPR29 | Potri.001G217600.1 | POPTR_0001s22530.1 | Chr01 | - | 22359570 | 22362385 | 1 | exon | (22359574_22364333) | 528 | E+ | 13 | 63-P1-L1-S1-SS-P1-L1-S1-P2-L2-S2-E1-E2-E+ |
| PtrPPR30 | Potri.001G220300.1 | POPTR_0001s22750.1 | Chr01 | + | 22596826 | 22603070 | 7 | exon-exon-exon-exon-exon-exon-exon | (22596986-22597041)(22597618-22597930)(22598515-22598805)(22599766-22600014)(22600654-22600778)(22601370-22601468)(22601889-22602549) | 597 | P | 10 | 137-P-P-P-P-P-P-P-P-P-P-110 |
| PtrPPR31 | Potri.001G236800.1 | POPTR_0001s24340.1 | Chr01 | - | 24818494 | 24821647 | 1 | exon | (24818583_24821310) | 915 | P | 22 | 104-P-18-P-P-P-P-P-P-P-P-P-P-P-P-P-P-P-P-P-P-P-P-P-26 |
| PtrPPR32 | Potri.001G243800.1 | POPTR_0001s25060.1 | Chr01 | - | 25488878 | 25490251 | 2 | exon-exon | (25488878_25489194)(25489247_25490168) | 392 | E2 | 11 | 9-L1-S1-P1-P1-L1-S1-P2-L2-S2-E1-E2-11 |
| PtrPPR33 | Potri.001G245600.1 | POPTR_0001s25240.1 | Chr01 | - | 25616287 | 25617500 | 4 | exon-exon-exon-exon | (25616287-25616434)(25616491-25616692)(25616968-25617252)(25617305-25617500) | 276 | E2 | 3 | 123-S2-E1-E2-53 |
| PtrPPR34 | Potri.001G258500.1 | POPTR_0001s26570.1 | Chr01 | - | 26750305 | 26753233 | 1 | exon | (26753273_26755897) | 861 | E+ | 24 | 25-P1-L1-S1-P1-L1-S1-P1-L1-S1-P1-L1-S1-P1-L1-S1-P1-L1-S1-P2-L2-S2-E1-E2-E+ |
| PtrPPR35 | Potri.001G263500.1 | POPTR_0001s27070.1 | Chr01 | - | 27222119 | 27225538 | 4 | exon-exon-exon-exon | (27222119-27222134)(27224815-27224934)(27224976-27225229)(27225270-27225538) | 252 | P | 6 | P-P-15-P-P-P-P-P-12 |
| PtrPPR36 | Potri.001G272500.1 |  | Chr01 | - | 27965468 | 27966817 | 3 | exon-exon-exon | (27965468-27965931)(27966059-27966132)(27966561-27966817) | 264 | E+ | 2 | 168-E2-E+ |
| PtrPPR37 | Potri.001G273100.1 | POPTR_0001s27990.1 | Chr01 | - | 27997556 | 28000850 | 2 | exon-exon | (27997556-27997692)(27997974-27999073) | 411 | P | 6 | 121-P-70-P-P-P-P-P-10 |
| PtrPPR38 | Potri.001G275000.1 | POPTR_0001s28180.1 | Chr01 | - | 28143831 | 28146875 | 2 | exon-exon | (28144050-28144373)(28146010-28146426) | 246 | P | 2 | 122-P-P-54 |
| PtrPPR39 | Potri.001G275100.1 | POPTR_0001s28190.1 | Chr01 | - | 28147989 | 28148919 | 3 | exon-exon-exon | (28147989-28148221)(28148312-28148597)(28148611-28148919) | 275 | P | 5 | 17-P-P-P-P-21-P-58 |
| PtrPPR40 | Potri.001G276500.1 | POPTR_0001s28320.1 | Chr01 | - | 28310468 | 28313674 | 2 | exon-exon | (28310468_28312389)(28312440_28312465) | 648 | P | 15 | 123-P-18-P-P-P-P-P-P-P-P-P-P-P-P-P-P |
| PtrPPR41 | Potri.001G297300.1 | POPTR_0001s30450.1 | Chr01 | - | 30122680 | 30130061 | 1 | exon | (30122747-30125236) | 829 | P | 19 | 145-P-P-P-P-P-P-P-P-P-P-P-P-P-P-P-P-P-P-P-15 |
| PtrPPR42 | Potri.001G316500.1 | POPTR_0001s32380.1 | Chr01 | + | 32194226 | 32196818 | 2 | exon-exon | (32194226-32195553)(32195670-32196502) | 719 | DYW | 14 | 143-P1-L1-S1-SS-SS-P1-L1-S1-P2-L2-S2-E1-E2-DYW |
| PtrPPR43 | Potri.001G321700.1 | POPTR_0001s32890.1 | Chr01 | - | 32611480 | 32613506 | 1 | exon | (32611603-32613506) | 634 | DYW | 15 | 26-L1-S1-P1-L1-S1-SS-P1-L1-S1-P2-L2-S2-E1-E2-DYW |
| PtrPPR44 | Potri.001G322100.1 | POPTR_0001s32930.1 | Chr01 | + | 32658615 | 32661639 | 2 | exon-exon | (32658786-32659742)(32659973-32661016) | 666 | DYW | 15 | 46-P1-L1-S1-P1-9-L1-S1-P1-L1-S1-P2-L2-S2-E1-E2-DYW |
| PtrPPR45 | Potri.001G326800.1 | POPTR_0001s33450.1 | Chr01 | - | 33103672 | 33107449 | 2 | exon-exon | (33103672-33105326)(33105513-33105829) | 656 | P | 10 | 82-P-P-P-P-P-104-P-P-P-P-P-119 |
| PtrPPR46 | Potri.001G341400.1 | POPTR_0001s35760.1 | Chr01 | - | 34623745 | 34632105 | 13 | exon-exon-exon-exon-exon-exon-exon-exon-exon-exon-exon-exon-exon | (34623911-34623929)(34624026-34624161)(34624255-34624417)(34624496-34624561)(34624647-34624744)(34625165-34625282)(34625374-34625586)(34625736-34625812)(34628365-34628458)(34630180-34630245)(34630326-34630403)(34630519-34630620)(34631557-34631706) | 459 | P | 6 | 36-P-P-P-P-76-P-P-136 |
| PtrPPR47 | Potri.001G354400.1 | POPTR_0001s34330.1 | Chr01 | - | 36198527 | 36200873 | 1 | exon | (36198527..36200747) | 739 | DYW | 17 | 75-SS-SS-SS-SS-SS-P1-L1-S1-P1-L1-S1-P2-L2-S2-E1-E2-DYW |
| PtrPPR48 | Potri.001G361500.1 | POPTR_0001s37250.1 | Chr01 | - | 37335114 | 37337661 | 1 | exon | (37335250-37336980) | 576 | P | 8 | 233-P-P-P-36-P-P-P-P-P-26 |
| PtrPPR49 | Potri.001G368700.1 | POPTR_0001s37740.1 | Chr01 | + | 38277912 | 38281578 | 2 | exon-exon | (38277912-38278078)(38278200-38281435) | 1133 | P | 27 | 109-P-16-P-P-P-P-P-P-P-P-P-P-P-P-P-P-P-P-P-P-P-P-P-P-P-P-P-P-62 |
| PtrPPR50 | Potri.001G369900.1 | POPTR_0001s37860.1 | Chr01 | - | 38394692 | 38396851 | 1 | exon | (38394735_38396618) | 627 | DYW | 15 | 20-L1-S1-P1-L1-S1-SS-P1-L1-S1-P2-L2-S2-E1-E2-DYW |
| PtrPPR51 | Potri.001G379500.1 | POPTR_0001s38850.1 | Chr01 | + | 39568590 | 39569831 | 2 | exon-exon | (39568590-39568690)(39568775-39569831) | 385 | P | 5 | 178-P-P-P-P-P-32 |
| PtrPPR52 | Potri.001G417400.1 | POPTR_0001s44310.1 | Chr01 | - | 44240424 | 44248232 | 16 | exon-exon-exon-exon-exon-exon-exon-exon-exon-exon-exon-exon-exon-exon-exon-exon | (44240575-44240836)(44240919-44240969)(44241317-44241943)(44242129-44242308)(44242790-44242854)(44243873-44243980)(44244182-44244295)(44244640-44244735)(44244896-44244994)(44245920-44246039)(44246217-44246321)(44246756-44246920)(44247001-44247128)(44247237-44247315)(44247665-44247882)(44247990-44248176) | 867 | P | 7 | 204-P-176-P-35-P-P-32-P-P-33-P-143 |
| PtrPPR53 | Potri.001G457300.1 | POPTR_0001s46150.1 | Chr01 | - | 49153258 | 49157209 | 3 | exon-exon-exon | (49153258-49153852)(49153952-49154702)(49156818-49156843) | 456 | P | 10 | 61-P-P-P-P-P-P-P-P-34-P-P-9 |
| PtrPPR54 | Potri.001G459900.1 | POPTR_0001s46420.1 | Chr01 | - | 49382841 | 49390243 | 9 | exon-exon-exon-exon-exon-exon-exon-exon-exon | (49383157-49384973)(49385623-49385814)(49386227-49386308)(49386447-49386635)(49387425-49387538)(49387762-49387852)(49387930-49388159)(49388622-49389053)(49389646-49390005) | 1168 | P | 10 | 631-P-P-P-14-P-P-P-P-24-P-P-P-135 |
| PtrPPR55 | Potri.001G464200.1 | POPTR_0001s46850.1 | Chr01 | + | 49813847 | 49815761 | 2 | exon-exon | (49813847-49815518)(49815628-49815761) | 601 | E2 | 11 | 38-P1-L1-S1-P1-L1-S1-SS-P1-L1-S1-P2-L2-S2-E1-E2-52 |
| PtrPPR56 | Potri.001G466100.1 | POPTR_0001s47030.1 | Chr01 | - | 49940948 | 49942706 | 2 | exon-exon | (49940948-49940989)(49941103-49942684) | 540 | DYW | 13 | S1-P1-L1-S1-P1-L1-S1-P2-L2-S2-E1-E2-DYW |
| PtrPPR57 | Potri.001G471600.1 | POPTR_0001s47560.1 | Chr01 | + | 50290567 | 50292096 | 2 | exon-exon | (50290567-50290835)(50290899-50292096) | 488 | E2 | 14 | 9-L1-S1-P1-L1-S1-SS-P1-L1-S1-P2-L2-S2-E1-E2-29 |
| PtrPPR58 | Potri.002G010900.1 | POPTR_0002s01200.1 | Chr02 | - | 634752 | 639502 | 4 | exon-exon-exon-exon | (634891-636687)(637330-637482)(637583-637654)(638387-638494) | 709 | P | 15 | 168-P-P-P-P-P-P-P-P-P-P-P-P-P-P-P-19 |
| PtrPPR59 | Potri.002G014800.1 | POPTR_0002s01620.1 | Chr02 | - | 863513 | 865588 | 2 | exon-exon | (863530-864631)(864716-865392) | 592 | PLS | 10 | 44-L1-S1-P1-L1-S1-P1-L1-S1-P2-L2-208 |
| PtrPPR60 | Potri.002G021300.1 | POPTR_0002s02290.1 | Chr02 | - | 1284391 | 1287088 | 1 | exon | (1284391-1286530) | 712 | DYW | 17 | 34-P1-L1-S1-SS-P1-L1-S1-SS-P1-L1-S1-P2-L2-S2-E1-E2-DYW |
| PtrPPR61 | Potri.002G027800.1 | POPTR_0002s02930.1 | Chr02 | - | 1795251 | 1797679 | 1 | exon | (1795251-1797635) | 797 | DYW | 20 | 12-L1-S1-SS-P1-L1-SS-SS-P1-L1-S1-SS-P1-L1-S1-P2-L2-S2-E1-E2-DYW |
| PtrPPR62 | Potri.002G030200.1 | POPTR_0002s03160.1 | Chr02 | + | 1950976 | 1954544 | 1 | exon | (1951019-1953697) | 892 | DYW | 21 | 78-L1-S1-P1-L1-S1-P1-P1-P1-L1-S1-P1-P1-P1-L1-S1-P2-L2-S2-E1-E2-DYW |
| PtrPPR63 | Potri.002G034900.1 | POPTR_0002s03630.1 | Chr02 | - | 2244722 | 2246607 | 4 | exon-exon-exon-exon | (2244722-2244873)(2245111-2245700)(2245809-2246291)(2246441-2246607) | 463 | P | 5 | 249-P-P-P-P-P-39 |
| PtrPPR64 | Potri.002G047600.1 | POPTR_0002s04850.1 | Chr02 | - | 3071654 | 3074597 | 6 | exon-exon-exon-exon-exon-exon | (3071654-3072503)(3072554-3072700)(3072928-3073058)(3073663-3073811)(3073855-3074166)(3074357-3074597) | 526 | E2 | 13 | S1-P1-L1-S1-P1-L1-41-S1-SS-P2-L2-S2-E1-E2-75 |
| PtrPPR65 | Potri.002G059500.1 |  | Chr02 | - | 4032362 | 4032969 | 2 | exon-exon | (4032405-4032660)(4032699-4032969) | 175 | P | 2 | 41-L1-18-P2-46 |
| PtrPPR66 | Potri.002G072500.1 | POPTR_0002s07340.1 | Chr02 | - | 5007678 | 5009207 | 1 | exon | 5007678_5009001 | 440 | E2 | 12 | S1-SS-SS-SS-SS-P1-L1-S1-P2-L2-S2-38-E2-17 |
| PtrPPR67 | Potri.002G075000.1 | POPTR_0002s07570.1 | Chr02 | - | 5191952 | 5194432 | 6 | exon-exon-exon-exon-exon-exon | (5191952-5192104)(5192183-192440)(5192456-5192564)(5193387-5193424)(5193617-5193875)(5193888-5194432) | 453 | E1 | 8 | 53-SS-P1-27-P1-20-P1-30-S1-32-L2-S2-E1-22 |
| PtrPPR68 | Potri.002G075200.1 | POPTR_0002s07580.1 | Chr02 | - | 5196367 | 5198214 | 1 | exon | (5196367-5198214) | 615 | E2 | 18 | 5-SS-SS-P1-L1-S1-SS-P1-L1-S1-SS-P1-L1-S1-P2-L2-S2-E1-E2-9 |
| PtrPPR69 | Potri.002G075300.1 | POPTR_0002s07590.1 | Chr02 | - | 5199792 | 5202625 | 2 | exon-exon | (5199831-5200538)(5200642-5200712)(5200982-5201960) | 585 | E1 | 15 | 56-L1-S1-P1-L1-S1-P1-L1-S1-P1-L1-S1-P2-L2-S2-E1-15 |
| PtrPPR70 | Potri.002G080300.1 | POPTR_0002s08080.1 | Chr02 | + | 5574252 | 5576691 | 1 | exon | (5574506-5575852) | 448 | P | 7 | 132-P-P-P-P-P-P-P-52 |
| PtrPPR71 | Potri.002G087400.1 | POPTR_0002s08800.1 | Chr02 | + | 6174091 | 6176402 | 1 | exon | (6174091-6176167) | 691 | E2 | 20 | 2-L1-S1-P1-L1-S1-SS-P1-L1-S1-P1-L1-S1-P1-L1-S1-P2-L2-S2-E1-E2-10 |
| PtrPPR72 | Potri.002G091100.1 | POPTR_0002s09170.1 | Chr02 | + | 6486739 | 6488539 | 5 | exon-exon-exon-exon-exon | (6486739-6487098)(6487185-6487248)(6487675-6487851)(6487915-6487977)(6488406-6488534) | 263 | PLS | 4 | P1-L1-S1-53-P1-86 |
| PtrPPR73 | Potri.002G103600.1 | POPTR_0002s10380.1 | Chr02 | + | 7508631 | 7510748 | 1 | exon | (7509098-7510627) | 509 | P | 11 | 82-P-P-37-P-P-P-P-P-P-P-P-P-3 |
| PtrPPR74 | Potri.002G109100.1 | POPTR_0002s10980.1 | Chr02 | - | 8030615 | 8031490 | 3 | exon-exon-exon | (8030615-8030772)(8030914-8030949)(8031040-8031490) | 214 | PLS | 7 | S1-SS-P2-L2-S2-E1-E2 |
| PtrPPR75 | Potri.002G109500.1 | POPTR_0002s11020.1 | Chr02 | - | 8047541 | 8050082 | 2 | exon-exon | (8047858-8049615)(8049728-8049883) | 637 | P | 14 | 94-P-P-P-P-P-P-P-P-P-P-P-P-P-P-56 |
| PtrPPR76 | Potri.002G139400.1 | POPTR_0002s14020.1 | Chr02 | + | 10342221 | 10344853 | 2 | exon-exon | (10342306-10342671)(10343145-10344398) | 539 | P | 9 | 100-P-35-P-P-P-P-P-P-P-P-88 |
| PtrPPR77 | Potri.002G155100.1 | POPTR_0002s15650.1 | Chr02 | - | 11687123 | 11689552 | 1 | exon | (11687123..11689526) | 800 | DYW | 20 | 26-SS-SS-P1-L1-S1-P1-L1-S1-P1-L1-S1-P1-L1-S1-P2-L2-S2-E1-E2-DYW |
| PtrPPR78 | Potri.002G164500.1 | POPTR_0002s16600.1 | Chr02 | - | 12423253 | 12424381 | 2 | exon-exon | (12423253-12423597)(12424283-12424381) | 147 | P | 4 | P-P-P-P-28 |
| PtrPPR79 | Potri.002G175900.1 | POPTR_0002s17640.1 | Chr02 | + | 13522191 | 13524102 | 2 | exon-exon | (13522311-13523237)(13523342-13523959) | 514 | E+ | 14 | 15-S1-P1-L1-S1-SS-P1-L1-S1-P2-L2-S2-E1-E2-E+ |
| PtrPPR80 | Potri.002G176100.1 | POPTR_0002s17660.1 | Chr02 | - | 13531233 | 13534259 | 1 | exon | (13531323-13533257) | 644 | P | 12 | 144-P-P-P-P-P-P-P-P-P-P-P-P-79 |
| PtrPPR81 | Potri.002G177200.1 | POPTR_0002s17780.1 | Chr02 | + | 13677638 | 13678027 | 1 | exon | (13677638..13678027) | 129 | P | 4 | P-P-P-P-30 |
| PtrPPR82 | Potri.002G179300.1 | POPTR_0002s18000.1 | Chr02 | + | 13939193 | 13941626 | 1 | exon | (13939880-13941401) | 503 | P | 11 | 44-P-P-P-P-P-P-36-P-P-P-P-P-31 |
| PtrPPR83 | Potri.002G182900.1 | POPTR_0002s18370.1 | Chr02 | - | 14319333 | 14321663 | 2 | exon-exon | (14319333-14320872)(14320958-14321663) | 748 | P | 15 | 64-P-P-P-46-P-P-P-P-P-P-P-76-P-P-P-P-P-32 |
| PtrPPR84 | Potri.002G183100.1 | POPTR_0002s18390.1 | Chr02 | - | 14329031 | 14331361 | 1 | exon | (14329031-14331361) | 776 | P | 15 | 64-P-P-P-46-P-P-P-P-P-P-P-104-P-P-P-P-P-32 |
| PtrPPR85 | Potri.002G192100.1 |  | Chr02 | - | 15302956 | 15305767 | 5 | exon-exon-exon-exon-exon | (15302956-15303063)(15303179-15303430)(15303491-15303538)(15303620-15303806)(15305472-15305767) | 296 | P | 4 | 58-P-P-P-P-113 |
| PtrPPR86 | Potri.002G193900.1 | POPTR_0002s19470.1 | Chr02 | - | 15502781 | 15508201 | 4 | exon-exon-exon-exon | (15503175-15505129)(15505784-15506001)(15506107-15506291)(15506385-15506649) | 873 | PLS | 13 | 192-SS-P1-P1-P1-P1-P1-P1-P1-P1-P1-P1-27-P1-P1-198 |
| PtrPPR87 | Potri.002G194400.1 | POPTR_0002s19520.1 | Chr02 | - | 15541499 | 15543762 | 2 | exon-exon | (15541499-15541760)(15542386-15543660) | 511 | P | 9 | 65-P-33-P-P-P-P-13-P-P-P-39-P-45 |
| PtrPPR88 | Potri.002G214600.1 |  | Chr02 | + | 19652348 | 19655520 | 1 | exon | (19652354-19653829) | 491 | PLS | 12 | 36-L1-S1-P1-L1-S1-SS-P1-L1-S1-P2-L2-S2-53 |
| PtrPPR89 | Potri.002G214900.1 | POPTR_0002s22590.1 | Chr02 | - | 19705942 | 19708123 | 2 | exon-exon | (19705942-19705988)(19706113-19708123) | 685 | DYW | 15 | 47-S1-P1-L1-S1-P1-32-S1-P1-L1-S1-P2-L2-S2-E1-E2-DYW |
| PtrPPR90 | Potri.002G220300.1 |  | Chr02 | + | 20622624 | 20625318 | 2 | exon-exon | (20622624-20622670)(20622795-20624806) | 685 | DWY | 15 | 47-S1-P1-L1-S1-P1-32-S1-P1-L1-S1-P2-L2-S2-E1-E2-DYW |
| PtrPPR91 | Potri.002G220600.1 |  | Chr02 | + | 20701167 | 20704273 | 2 | exon-exon | (20701167-20701869)(20701926-20702642) | 472 | PLS | 11 | 36-L1-S1-P1-L1-S1-P1-L1-S1-P2-L2-S2-53 |
| PtrPPR92 | Potri.002G237100.1 | POPTR_0002s23820.1 | Chr02 | - | 23011713 | 23014157 | 1 | exon | (23011713..23014157) | 814 | DYW | 20 | 41-SS-SS-P1-L1-S1-P1-L1-S1-P1-L1-S1-P1-L1-S1-P2-L2-S2-E1-E2-DYW |
| PtrPPR93 | Potri.002G239600.1 | POPTR_0002s24060.1 | Chr02 | - | 23234394 | 23236501 | 2 | exon-exon | (23234598-23235226)(23235266-23236501) | 621 | DYW | 13 | 63-S1-P1-L1-S1-SS-P1-L1-S1-P2-L2-S2-E1-E2-DYW |
| PtrPPR94 | Potri.002G243600.1 | POPTR_0002s24510.1 | Chr02 | - | 23578298 | 23581716 | 4 | exon-exon-exon-exon | (23579587-23579631)(23580049-23580455)(23580901-23581615) | 388 | P | 10 | 29-P-P-P-P-P-P-P-P-P-P-7 |
| PtrPPR95 | Potri.002G245200.1 | POPTR_0002s24650.1 | Chr02 | + | 23673217 | 23675025 | 1 | exon | (2367321-23675025) | 602 | E+ | 16 | 39-SS-P1-L1-S1-P1-L1-S1-P1-L1-S1-P2-L2-S2-E1-E2-E+ |
| PtrPPR96 | Potri.002G248100.1 | POPTR_0002s24950.1 | Chr02 | + | 23870091 | 23872073 | 1 | exon | (23870180-23871343) | 387 | P | 7 | 115-P-P-P-P-P-P-P-17 |
| PtrPPR97 | Potri.002G258800.1 | POPTR_0002s26050.1 | Chr02 | + | 24759658 | 24763407 | 5 | exon-exon-exon-exon-exon | (24759658-24761247)(24761315-24761364)(24761955-24762200)(24762277-24762523)(24762739-24763407) | 933 | E+ | 14 | 44-P1-L1-S1-SS-SS-P1-L1-S1-P2-L2-S2-E1-E2-E+ |
| PtrPPR98 | Potri.003G000700.1 | POPTR_0003s00850.1 | Chr03 | + | 149648 | 153018 | 9 | exon-exon-exon-exon-exon-exon-exon-exon-exon | (149648-149666)(150042-150143)(150249-150911)(151051-151336)(151384-151835)(151876-151965)(152067-152214)(152267-152398)(153000-153018) | 636 | P | 8 | 171-P-61-P-P-P-P-35-P-P-P-107 |
| PtrPPR99 | Potri.003G003000.1 | POPTR_0022s00240.1 | Chr03 | - | 311516 | 315637 | 6 | exon-exon-exon-exon-exon-exon | (311516-311722)(311742-312128)(312248-313761)(313978-314277)(314519-314818)(315061-315637) | 1094 | P | 12 | 182-P-P-P-P-P-P-P-P-P-P-P-P-481 |
| PtrPPR100 | Potri.003G006800.1 | POPTR_0022s00580.1 | Chr03 | - | 554200 | 559298 | 1 | exon | (554200-556600) | 799 | DYW | 20 | 8-L1-S1-P1-L1-S1-P1-L1-S1-P1-L1-S1-P1-L1-S1-P2-L2-S2-E1-E2-DYW |
| PtrPPR101 | Potri.003G008900.1 | POPTR_0022s00740.1 | Chr03 | - | 717007 | 719686 | 1 | exon | (717007-718861) | 617 | P | 13 | 148-P-P-P-P-P-P-P-P-P-P-P-P-P-14 |
| PtrPPR102 | Potri.003G016800.1 |  | Chr03 | + | 2075579 | 2076432 | 3 | exon-exon-exon | (2075579-2075853)(2075975-2076084)(2076332-2076432) | 161 | PLS | 3 | S1-P1-26-P1-35 |
| PtrPPR103 | Potri.003G018000.1 | POPTR_0003s01245.1 | Chr03 | + | 2216355 | 2217664 | 2 | exon-exon | (2216355-2216527)(2216631-2217238) | 259 | PLS | 5 | P1-22-P1-26-S1-SS-P2-44 |
| PtrPPR104 | Potri.003G018300.1 |  | Chr03 | + | 2233541 | 2236130 | 3 | exon-exon-exon | (2233541-2233883)(2235672-2235883)(2235975-2236130) | 236 | PLS | 4 | 27-S1-75-SS-31-SS-P2 |
| PtrPPR105 | Potri.003G019000.1 | POPTR_0003s01330.1 | Chr03 | - | 2302883 | 2317768 | 10 | exon-exon-exon-exon-exon-exon-exon-exon-exon-exon | (2302893-2303209)(2305056-2305140)(2306814-2307077)(2307674-2307916)(2308569-2308673)(2309222-2309321)(2309958-2310277)(2312447-2312623)(2312704-2312832)(2313876-2314106) | 656 | P | 11 | 112-P-34-P-16-P-P-P-P-P-P-P-P-68-P-41 |
| PtrPPR106 | Potri.003G031600.1 | POPTR_0003s02590.1 | Chr03 | - | 3969151 | 3971680 | 2 | exon-exon | (3969373-3970031)(3970284-3971607) | 660 | PLS | 14 | 35-L1-S1-P1-L1-S1-SS-SS-P1-L1-S1-P1-L1-S1-P2-169 |
| PtrPPR107 | Potri.003G041500.1 | POPTR_0003s04280.1 | Chr03 | - | 5509094 | 5511846 | 4 | exon-exon-exon-exon | (5509326-5509996)(5510171-5510232)(5510294-5510407)(5511441-5511846) | 417 | E+ | 10 | 46-L1-S1-P1-L1-S1-P1-19-S2-E1-E2-E+ |
| PtrPPR108 | Potri.003G041800.1 | POPTR_0019s02820.1 | Chr03 | - | 5548063 | 5549954 | 2 | exon-exon | (5548063-5548083)(5549490-5549954) | 161 | E+ | 4 | 11-S2-E1-E2-E+ |
| PtrPPR109 | Potri.003G042600.1 | POPTR_0003s04250.1 | Chr03 | - | 5691316 | 5692257 | 3 | exon-exon-exon | 5691316_5691360,5691555_5691721,5691876_5692257 | 197 | E+ | 5 | 5-P2-16-S2-E1-E2-E+ |
| PtrPPR110 | Potri.003G045800.1 | POPTR_0003s03950.1 | Chr03 | - | 6152297 | 6153934 | 1 | exon | (6152359-6153777) | 472 | P | 10 | 89-P-P-P-P-P-P-P-P-P-P-32 |
| PtrPPR111 | Potri.003G058700.1 | POPTR_0003s05740.1 | Chr03 | - | 8646244 | 8649234 | 1 | exon | (8646244-8648881) | 878 | DYW | 19 | 137-S1-P1-L1-S1-P1-L1-S1-P1-L1-S1-P1-L1-S1-P2-L2-S2-E1-E2-DYW |
| PtrPPR112 | Potri.003G062700.1 | POPTR_0003s06110.2 | Chr03 | - | 9094647 | 9099202 | 1 | exon | (9094918-9096123) | 401 | P | 7 | 106-P-36-P-P-P-P-P-P-14 |
| PtrPPR113 | Potri.003G074500.1 | POPTR_0003s07210.1 | Chr03 | - | 10314394 | 10316373 | 1 | exon | (10314464-10316373) | 636 | DYW | 14 | 57-L1-S1-P1-L1-S1-P1-L1-S1-P2-L2-S2-E1-E2-DYW |
| PtrPPR114 | Potri.003G081700.1 | POPTR_0003s07960.1 | Chr03 | + | 10963559 | 10965268 | 1 | exon | (10963559-10965268) | 569 | DYW | 14 | 5-SS-SS-SS-SS-SS-P1-L1-S1-P2-L2-S2-E1-E2-DYW |
| PtrPPR115 | Potri.003G084400.1 | POPTR_0003s08270.1 | Chr03 | + | 11195090 | 11197566 | 1 | exon | (11195090-11197196) | 701 | P | 8 | 228-P-38-P-59-P-P-P-45-P-P-P-46 |
| PtrPPR116 | Potri.003G087100.1 | POPTR_0003s08540.1 | Chr03 | - | 11441615 | 11443468 | 1 | exon | (11441615..11443468) | 617 | E2 | 14 | 121-P1-L1-S1-SS-SS-SS-P1-L1-S1-P2-L2-S2-E1-E2-29 |
| PtrPPR117 | Potri.003G087300.1 | POPTR_0003s08560.1 | Chr03 | - | 11457754 | 11460485 | 1 | exon | (11457754-11459989) | 744 | E+ | 21 | 43-SS-SS-SS-SS-SS-SS-SS-P1-L1-S1-SS-SS-P1-L1-S1-P2-L2-S2-E1-E2-E+ |
| PtrPPR118 | Potri.003G088600.1 | POPTR_0003s08690.1 | Chr03 | - | 11604826 | 11608333 | 2 | exon-exon | (11604826-11606666)(11606793-11606806) | 617 | E+ | 18 | L1-S1-P1-L1-S1-P1-L1-S1-SS-P1-L1-S1-P2-L2-S2-E1-E2-E+ |
| PtrPPR119 | Potri.003G089600.1 | POPTR_0003s08800.1 | Chr03 | - | 11682642 | 11685418 | 1 | exon | (11682918-11685083) | 721 | P | 10 | 192-P-P-P-P-P-P-P-P-P-67-P-111 |
| PtrPPR120 | Potri.003G105700.1 | POPTR_0003s10490.1 | Chr03 | + | 12999229 | 13002215 | 2 | exon-exon | (12999229-13001721)(13002126-13002215) | 860 | P | 18 | 226-P-P-P-P-P-P-P-P-P-P-P-P-P-P-P-P-P-P-4 |
| PtrPPR121 | Potri.003G116100.1 | POPTR_0003s11580.1 | Chr03 | + | 13950028 | 13953914 | 2 | exon-exon | (13950075-13950101)(13951145-13952908) | 596 | E2 | 15 | 88-P1-L1-S1-SS-SS-SS-SS-P1-L1-S1-P2-L2-S2-E1-E2-10 |
| PtrPPR122 | Potri.003G154800.1 | POPTR_0003s15360.1 | Chr03 | - | 16776328 | 16778478 | 1 | exon | (16776328-16778254) | 641 | P | 14 | 59-P-P-P-P-P-P-P-P-P-P-P-P-P-P-86 |
| PtrPPR123 | Potri.003G155600.1 | POPTR_0003s15430.1 | Chr03 | - | 16826922 | 16830128 | 1 | exon | (16827123-16828418) | 431 | E2 | 12 | 17-P1-L1-SS-SS-P1-L1-S1-P2-L2-S2-E1-E2-7 |
| PtrPPR124 | Potri.003G160100.1 | POPTR_0003s15840.1 | Chr03 | + | 17178903 | 17181144 | 1 | exon | 17178903_17180995 | 663 | DYW | 15 | 58-P1-L1-S1-P1-L1-S1-P1-L1-S1-P2-L2-S2-E1-E2-DYW |
| PtrPPR125 | Potri.003G163700.1 |  | Chr03 | + | 17449844 | 17450603 | 3 | exon-exon-exon | (17449844-17450099)(17450147-17450245)(17450341-17450603) | 205 | PLS | 2 | 18-P-19-SS-102 |
| PtrPPR126 | Potri.003G164900.1 | POPTR_0003s16340.1 | Chr03 | - | 17551510 | 17558128 | 1 | exon | (17551510-17554156) | 881 | DYW | 20 | 104-L1-S1-P1-L1-S1-P1-L1-S1-P1-L1-S1-P1-L1-S1-P2-L2-S2-E1-E2-DYW |
| PtrPPR127 | Potri.003G191000.1 | POPTR_0003s19010.1 | Chr03 | - | 19547558 | 19550321 | 1 | exon | 19547558_19549997 | 812 | DYW | 19 | 71-S1-P1-L1-S1-P1-L1-S1-P1-L1-S1-P1-L1-S1-P2-L2-S2-E1-E2-DYW |
| PtrPPR128 | Potri.003G193600.1 | POPTR_0003s19300.1 | Chr03 | - | 19738859 | 19741613 | 2 | exon-exon | (19739066-19739347)(19740114-19741406) | 524 | P | 7 | 141-P-34-P-36-P-P-P-P-P-70 |
| PtrPPR129 | Potri.003G203100.1 | POPTR_0003s20190.1 | Chr03 | + | 20447584 | 20451939 | 1 | exon | (20447627-20449813) | 728 | P | 15 | 159-P-P-P-P-P-P-P-P-P-P-P-P-P-P-P-45 |
| PtrPPR130 | Potri.003G204100.1 | POPTR_0003s20290.1 | Chr03 | - | 20500178 | 20502258 | 1 | exon | (20500264-20501655) | 463 | P | 11 | 45-P-P-P-P-P-P-P-P-P-P-P-33 |
| PtrPPR131 | Potri.003G223900.1 | POPTR_0003s20710.1 | Chr03 | + | 21763525 | 21767700 | 1 | exon | (21763525-21769311) | 508 | E+ | 13 | 42-P1-L1-S1-SS-P1-L1-S1-P2-L2-S2-E1-E2-E+ |
| PtrPPR132 | Potri.004G013300.1 | POPTR_0004s01330.1 | Chr04 | - | 843117 | 845439 | 1 | exon | (843167-845439) | 757 | P | 17 | 88-P-36-P-P-P-P-P-P-P-P-P-P-P-P-P-P-P-33-P-3 |
| PtrPPR133 | Potri.004G018000.1 | POPTR_0004s01760.1 | Chr04 | - | 1187812 | 1189421 | 1 | exon | 1187840_1189054 | 404 | P | 9 | 68-P-P-P-P-P-P-P-P-P-12 |
| PtrPPR134 | Potri.004G020900.1 | POPTR_0004s02020.1 | Chr04 | - | 1401792 | 1403512 | 1 | exon | (1401819-1403512) | 564 | P | 12 | 135-P-P-P-P-P-37-P-P-P-P-P-P-13 |
| PtrPPR135 | Potri.004G029900.1 | POPTR_0004s02930.1 | Chr04 | + | 2166514 | 2168136 | 1 | exon | (2166563-2167708) | 381 | PLS | 6 | 93-S1-P1-P1-P1-P1-P1-77 |
| PtrPPR136 | Potri.004G030000.1 | POPTR_0004s02940.1 | Chr04 | + | 2169699 | 2171277 | 1 | exon | (2169822-2170943) | 373 | P | 8 | 90-P-P-P-P-P-P-P-P-2 |
| PtrPPR137 | Potri.004G042500.1 | POPTR_0004s04250.1 | Chr04 | - | 3223479 | 3225725 | 2 | exon-exon | (3223533-3223778)(3224232-3225464) | 492 | P | 7 | 60-P-34-P-P-P-36-P-P-P-117 |
| PtrPPR138 | Potri.004G043400.1 | POPTR_0004s04340.1 | Chr04 | + | 3288183 | 3290573 | 1 | exon | (3288232-3290007) | 591 | DYW | 14 | 8-L1-S1-P1-L1-S1-P1-L1-S1-P2-L2-S2-E1-E2-DYW |
| PtrPPR139 | Potri.004G047800.1 | POPTR_0004s04740.1 | Chr04 | - | 3680718 | 3683294 | 1 | exon | (3680718..3683294) | 858 | DYW | 23 | L1-S1-P1-L1-S1-P1-L1-S1-P1-L1-S1-P1-L1-S1-P1-L1-S1-P2-L2-S2-E1-E2-DYW |
| PtrPPR140 | Potri.004G054000.1 | POPTR_0004s05320.1 | Chr04 | + | 4234746 | 4236876 | 1 | exon | (4234789-4236495) | 568 | E2 | 13 | 79-L1-S1-P1-L1-S1-SS-P1-L1-S1-P2-L2-S2-E1-E2-13 |
| PtrPPR141 | Potri.004G059400.1 | POPTR_0004s05810.1 | Chr04 | + | 4767068 | 4770047 | 1 | exon | (4767251-4769890) | 879 | DYW | 20 | 103-L1-S1-P1-L1-S1-P1-L1-S1-P1-L1-S1-P1-L1-S1-P2-L2-S2-E1-E2-DYW |
| PtrPPR142 | Potri.004G066600.1 | POPTR_0004s06520.1 | Chr04 | - | 5577029 | 5579431 | 1 | exon | (557702-5579431) | 800 | P | 18 | 94-P-34-P-P-P-P-P-P-P-P-P-P-P-31-P-P-P-P-P-P |
| PtrPPR143 | Potri.004G071800.1 | POPTR_0004s07030.1 | Chr04 | + | 6046636 | 6049267 | 1 | exon | (6046636-6048952) | 771 | E2 | 22 | 17-L1-S1-P1-L1-S1-P1-34-S1-P1-L1-S1-P1-L1-S1-P1-L1-S1-P2-L2-S2-E1-E2-12 |
| PtrPPR144 | Potri.004G074100.1 | POPTR_0004s07240.1 | Chr04 | - | 6182278 | 6186944 | 6 | exon-exon-exon-exon-exon-exon | (6182716_6183901)(6184286_6184375)(6184476_61846517)(6185716_6185836)(6186375_6186443)(6186547_6186641) | 581 | P | 4 | 106-P-P-P-P-326 |
| PtrPPR145 | Potri.004G074500.1 | POPTR_0004s07290.1 | Chr04 | + | 6223641 | 6227243 | 1 | exon | (6223803-6225572) | 589 | P | 15 | 57-P-P-P-P-P-P-P-P-P-P-P-P-P-P-P-6 |
| PtrPPR146 | Potri.004G074700.1 | POPTR_0004s07300.1 | Chr04 | + | 6234349 | 6236355 | 1 | exon | (6234349-6236035) | 561 | P | 13 | 100-P-P-P-P-P-P-P-P-P-P-P-P-P-6 |
| PtrPPR147 | Potri.004G090800.1 | POPTR_0004s08990.1 | Chr04 | - | 7728307 | 7730871 | 1 | exon | (7729016-7730686) | 556 | P | 12 | 108-P-19-P-P-P-P-P-P-P-P-P-P-P-9 |
| PtrPPR148 | Potri.004G102700.1 | POPTR_0004s10260.1 | Chr04 | + | 9021681 | 9023527 | 1 | exon | (9021717-9023288) | 523 | P | 10 | 111-P-P-P-P-P-P-P-P-P-P-62 |
| PtrPPR149 | Potri.004G108000.1 | POPTR_0004s10780.1 | Chr04 | + | 9550401 | 9552609 | 1 | exon | (9550448-9552427) | 659 | P | 9 | 150-P-102-P-P-37-P-P-P-P-P-31-P-22 |
| PtrPPR150 | Potri.004G111300.1 | POPTR_0004s11010.1 | Chr04 | + | 10071567 | 10075259 | 2 | exon-exon | (10071567-10074247)(10074276-10074289) | 897 | E+ | 24 | 48-L1-S1-SS-P1-L1-S1-P1-L1-S1-P1-L1-S1-P1-L1-S1-P1-L1-S1-P2-L2-S2-E1-E2-E+ |
| PtrPPR151 | Potri.004G124400.1 | POPTR_0004s12330.1 | Chr04 | + | 12019037 | 12021943 | 1 | exon | (12019037-12021068) | 676 | E2 | 19 | 20-L1-S1-P1-L1-S1-P1-L1-S1-P1-L1-S1-P1-L1-S1-P2-L2-S2-E1-E2-14 |
| PtrPPR152 | Potri.004G124900.1 | POPTR_0004s12370.1 | Chr04 | - | 12080684 | 12081028 | 1 | exon | (12080684-22095357) | 78 | P | 2 | P1-P-14 |
| PtrPPR153 | Potri.004G125500.1 | POPTR_0004s12430.1 | Chr04 | + | 12141970 | 12143760 | 1 | exon | (12141970-12143760) | 597 | E+ | 17 | 15-L1-S1-P1-L1-S1-SS-SS-SS-P1-L1-S1-P2-L2-S2-E1-E2-E+ |
| PtrPPR154 | Potri.004G134300.1 | POPTR_0004s14190.1 | Chr04 | - | 15473821 | 15479276 | 1 | exon | (15473821-15476434) | 870 | E+ | 25 | 5-P1-L1-S1-P1-L1-S1-P1-L1-S1-P1-L1-S1-P1-L1-S1-SS-P1-L1-S1-P2-L2-S2-E1-E2-E+ |
| PtrPPR155 | Potri.004G145400.1 | POPTR_0004s15263.1 | Chr04 | - | 16857993 | 16860426 | 8 | exon-exon-exon-exon-exon-exon-exon-exon | (16857993-16858001)(16858052-16858163)(16858199-16858290)(16858774-16858861)(16858905-16859147)(16859203-16859871)(16859948-16860097)(16860311-16860426) | 492 | P | 4 | 203-P-P-34-P-P-114 |
| PtrPPR156 | Potri.004G147700.1 | POPTR_0004s15480.1 | Chr04 | - | 17050750 | 17053686 | 1 | exon | (17050864-17052810) | 648 | P | 13 | 170-P-P-P-P-P-P-P-P-P-P-P-P-P-23 |
| PtrPPR157 | Potri.004G149400.1 | POPTR_0004s15660.1 | Chr04 | - | 17212576 | 17214993 | 1 | exon | (17212576-17214157) | 526 | P | 9 | 107-P-49-P-48-P-P-P-P-P-P-P-6 |
| PtrPPR158 | Potri.004G151500.1 | POPTR_0004s15870.1 | Chr04 | - | 17357094 | 17359685 | 1 | exon | (17357094-17359128) | 677 | E+ | 19 | 22-S1-SS-P1-L1-S1-SS-P1-L1-S1-SS-P1-L1-S1-P2-L2-S2-E1-E2-E+ |
| PtrPPR159 | Potri.004G152000.1 | POPTR_0004s15930.1 | Chr04 | - | 17394422 | 17397013 | 1 | exon | (17394422-17396455 | 677 | E+ | 19 | 22-S1-SS-P1-L1-S1-SS-P1-L1-S1-SS-P1-L1-S1-P2-L2-S2-E1-E2-E+ |
| PtrPPR160 | Potri.004G166600.1 | POPTR_0004s17400.1 | Chr04 | - | 18652692 | 18662368 | 19 | exon-exon-exon-exon-exon-exon-exon-exon-exon-exon-exon-exon-exon-exon-exon-exon-exon-exon-exon | (18652852-18653186)(18653750-18654485)(18654591-18654804)(18655040-18655103)(18655206-18655350)(18655448-18655563)(18655987-18656115)(18656267-18656440)(18656514-18656678)(18656968-18657108)(18657551-18657650)(18658587-18658695)(18658833-18658924)(18659143-18659233)(18659627-18659858)(18659954-18660046)(18660487-18660535)(18660742-18660835)(18661403-18661637) | 1104 | P | 12 | 413-P-32-P-P-P-P-P-P-P-P-P-P-91-P-146 |
| PtrPPR161 | Potri.004G171800.1 |  | Chr04 | + | 19071475 | 19071901 | 1 | exon | (19071475-19071550) | 101 | PLS | 3 | 6-P2-L2-S2-10 |
| PtrPPR162 | Potri.004G178200.1 | POPTR_0004s18470.1 | Chr04 | - | 19596791 | 19599344 | 2 | exon-exon | (19596791-19597690)(19597739-19599194) | 784 | DYW | 18 | 57-SS-P1-L1-S1-P1-L1-S1-P1-L1-S1-P1-L1-S1-P2-L2-S2-E1-E2-DYW |
| PtrPPR163 | Potri.004G184800.1 | POPTR_0004s19560.1 | Chr04 | - | 20080342 | 20082972 | 1 | exon | 20080342_20082972 | 876 | DYW | 20 | 65-P1-L1-S1-P1-L1-S1-P1-L1-S1-P1-31-S1-P1-L1-S1-P2-L2-S2-E1-E2-DYW |
| PtrPPR164 | Potri.004G185200.1 | POPTR_0004s19610.1 | Chr04 | - | 20111590 | 20117583 | 6 | exon-exon-exon-exon-exon-exon | (20111658-20112566)(20113262-20113363)(20113484-20113662)(20113923-20114043)(20116968-20117036)(20117185-20117484) | 559 | P | 3 | 18-P-P-35-P-393 |
| PtrPPR165 | Potri.004G200900.1 | POPTR_0004s21110.1 | Chr04 | - | 21238571 | 21241612 | 2 | exon-exon | (21238571-21240561)(21240922-21241612) | 893 | P | 18 | 125-P-P-P-P-P-P-P-P-72-P-P-P-P-P-30-P-P-P-P-P-33 |
| PtrPPR166 | Potri.004G205600.1 | POPTR_0004s21560.1 | Chr04 | - | 21489219 | 21493062 | 2 | exon-exon | (21489232-21491729)(21492117-21492243) | 874 | P | 20 | 74-P-P-P-P-P-P-P-P-P-P-P-P-23-P-P-P-P-P-34-P-P-72 |
| PtrPPR167 | Potri.004G205900.1 | POPTR_0004s21590.1 | Chr04 |  | 21495003 | 21497448 | 1 | exon | (21495026-21497275) | 749 | E+ | 21 | 11-P1-L1-S1-P1-L1-SS-P1-L1-S1-P1-L1-S1-P1-L1-S1-P2-L2-S2-E1-E2-E+ |
| PtrPPR168 | Potri.004G208000.1 | POPTR_0004s21780.1 | Chr04 | + | 21647766 | 21649806 | 1 | exon | (21647766-21649524) | 585 | DYW | 14 | 15-L1-S1-P1-L1-S1-P1-L1-S1-P2-L2-S2-E1-E2-DYW |
| PtrPPR169 | Potri.004G209500.1 | POPTR_0004s21920.1 | Chr04 | + | 21734783 | 21738225 | 1 | exon | (21734783-21736059) | 414 | P | 4 | 242-P-P-P-P-32 |
| PtrPPR170 | Potri.004G217900.1 |  | Chr04 | - | 22472049 | 22474064 | 2 | exon-exon | 22472049_22472997,22473187-22474064 | 608 | E+ | 15 | 48-L1-S1-P1-L1-S1-P1-L1-S1-39-SS-P2-L2-S2-E1-E2-E+ |
| PtrPPR171 | Potri.004G227800.1 | POPTR_0004s23640.1 | Chr04 | + | 23289961 | 23292194 | 2 | exon-exon | (23290018-23290499)(23290608-23292194) | 689 | P | 11 | 145-P-P-P-P-P-P-P-P-P-P-P-148 |
| PtrPPR172 | Potri.004G229100.1 | POPTR_0004s23800.1 | Chr04 | - | 23431063 | 23433771 | 2 | exon-exon | 23431063-23432199,23432242-23433771 | 888 | P | 15 | 199-P-34-P-P-22-P-P-P-P-P-P-35-P-P-P-P-67-P-P |
| PtrPPR173 | Potri.004G230400.1 |  | Chr04 | + | 23568192 | 23570412 | 6 | exon-exon-exon-exon-exon-exon | (23568192-23568270)(23569230-23569353)(23569415-23569490)(23569694-23569830)(23569909-23569977)(23570235-23570412) | 220 | PLS | 3 | 42-SS-SS-P2-77 |
| PtrPPR174 | Potri.004G237000.1 | POPTR_0004s24500.1 | Chr04 | + | 24259420 | 24261371 | 1 | exon | (24259420-24261319) | 632 | DYW | 15 | 18-L1-S1-P1-L1-S1-SS-P1-L1-S1-P2-L2-S2-E1-E2-DYW |
| PtrPPR175 | Potri.005G003200.1 | POPTR_0005s00500.1 | Chr05 | + | 180292 | 184707 | 1 | exon | (180292-182470) | 725 | P | 16 | 91-P-P-P-P-P-P-P-P-P-P-P-P-P-P-P-35-P-43 |
| PtrPPR176 | Potri.005G005700.1 | POPTR_0005s00730.1 | Chr05 | + | 328460 | 330599 | 1 | exon | (328460_330494) | 677 | DYW | 14 | 70-S1-P1-L1-S1-P1-33-P1-L1-S1-P2-L2-S2-E1-E2-DYW |
| PtrPPR177 | Potri.005G006400.1 | POPTR_0005s00800.1 | Chr05 | - | 367535 | 369481 | 2 | exon-exon | (367535-368008)(368249-369481) | 568 | DYW | 15 | SS-SS-SS-SS-SS-P1-P1-L1-S1-P2-L2-S2-E1-E2-DYW |
| PtrPPR178 | Potri.005G010900.1 | POPTR_0005s01440.1 | Chr05 | + | 803098 | 805088 | 2 | exon-exon | (803191-803811)(803995-805088) | 571 | E2 | 15 | 43-SS-P1-L1-S1-P1-L1-S1-P1-L1-S1-P2-L2-S2-E1-E2-21 |
| PtrPPR179 | Potri.005G011000.1 | POPTR_0005s01450.1 | Chr05 | - | 805421 | 809564 | 1 | exon | (805421-807803) | 793 | DYW | 18 | 90-P1-L1-S1-SS-SS-P1-L1-S1-SS-P1-L1-S1-P2-L2-S2-E1-E2-DYW |
| PtrPPR180 | Potri.005G012600.1 | POPTR_0005s01560.1 | Chr05 | - | 977260 | 979632 | 2 | exon-exon | (977276-977471)(977586-979273) | 627 | DYW | 14 | 56-P1-L1-S1-SS-SS-P1-L1-S1-P2-L2-S2-E1-E2-DYW |
| PtrPPR181 | Potri.005G014500.1 |  | Chr05 | - | 1127709 | 1131564 | 2 | exon-exon | (1127709-1128195)(1129038-1130897) | 781 | P | 11 | 358-P-P-P-P-P-P-P-P-P-P-P-35 |
| PtrPPR182 | Potri.005G017500.1 | POPTR_0005s01750.1 | Chr05 | - | 1418422 | 1421433 | 2 | exon-exon | (1418422-1418758)(1419575-1421433) | 731 | P | 11 | 308-P-P-P-P-P-P-P-P-P-P-P-35 |
| PtrPPR183 | Potri.005G019400.1 | POPTR_0005s01930.1 | Chr05 | - | 1521199 | 1522656 | 1 | exon | (1521199-1522656) | 485 | E2 | 14 | 5-L1-S1-P1-L1-S1-P1-P1-L1-S1-P2-L2-S2-E1-E2-8 |
| PtrPPR184 | Potri.005G035900.1 | scaffold_5:F:2534003-2535931 | Chr05 | + | 2647148 | 2649138 | 1 | exon | (2647148-2649080) | 643 | DYW | 16 | 1-L1-S1-P1-L1-S1-SS-SS-P1-L1-S1-P2-L2-S2-E1-E2-DYW |
| PtrPPR185 | Potri.005G038400.1 | POPTR_0005s04060.1 | Chr05 | - | 2818472 | 2820711 | 2 | exon-exon | (2818486-2820332)(2820628-2820711) | 643 | P | 13 | 130-P-P-P-P-P-P-P-P-P-P-P-P-P-58 |
| PtrPPR186 | Potri.005G038500.1 | POPTR_0005s04070.1 | Chr05 | - | 2822128 | 2823031 | 3 | exon-exon-exon | (2822128-2822254)(2822309-2822524)(2822607-2823031) | 255 | P | 5 | 3-P-13-P-P-41-P-P-22 |
| PtrPPR187 | Potri.005G045000.1 | POPTR_0005s04650.1 | Chr05 | - | 3302453 | 3304441 | 1 | exon | (3302453-3304307) | 617 | P | 15 | 60-P-P-P-P-P-P-P-P-P-P-P-P-P-P-P-32 |
| PtrPPR188 | Potri.005G046000.1 | POPTR_0005s04750.1 | Chr05 | - | 3371522 | 3375085 | 3 | exon-exon-exon | (3371522-3371589)(3371687-3371835)(3372954-3375085) | 782 | P | 16 | 221-P-P-P-P-P-P-P-P-P-P-P-P-P-P-P-P-1 |
| PtrPPR189 | Potri.005G046100.1 | POPTR_0005s04760.1 | Chr05 | - | 3375302 | 3379539 | 4 | exon-exon-exon-exon | (3375354-3376833)(3378254-3378453)(3378652-3378822)(3378886-3379140) | 701 | P | 18 | 44-P-P-P-P-P-P-P-P-P-P-P-P-P-P-P-P-14-P-P-18 |
| PtrPPR190 | Potri.005G046200.1 | POPTR_0005s04770.1 | Chr05 | - | 3381037 | 3383190 | 1 | exon | (3381037-3382876) | 612 | P | 15 | 56-P-P-P-P-P-P-P-P-P-P-P-P-P-P-P-31 |
| PtrPPR191 | Potri.005G047100.1 |  | Chr05 | + | 3439316 | 3439879 | 1 | exon | (3439316-3439879) | 187 | P | 5 | 6-P-P-P-P-P-7 |
| PtrPPR192 | Potri.005G047200.1 |  | Chr05 | + | 3439958 | 3440425 | 1 | exon | (3439958-3440425) | 155 | P | 3 | 9-P-P-P-41 |
| PtrPPR193 | Potri.005G047400.1 | POPTR_0005s04890.1 | Chr05 | + | 3443440 | 3445575 | 1 | exon | (3443538-3445043) | 501 | P | 10 | 120-P-P-P-P-P-P-P-P-P-P-31 |
| PtrPPR194 | Potri.005G050100.1 | POPTR_0005s05180.1 | Chr05 | - | 3610891 | 3612914 | 2 | exon | 3610891_3612433,3612895-3612914 | 520 | P | 15 | P-P-P-P-P-P-P-P-P-P-P-P-P-P-P-4 |
| PtrPPR195 | Potri.005G050200.1 | POPTR_0005s05190.1 | Chr05 | - | 3616400 | 3618082 | 1 | exon | (3616491-3618082 ) | 530 | P | 14 | 9-P-P-P-P-P-P-P-P-P-P-P-P-P-P-31 |
| PtrPPR196 | Potri.005G050300.1 | POPTR_0005s05170.1 | Chr05 | - | 3620778 | 3622708 | 1 | exon | (3620868-3622708) | 613 | P | 14 | 92-P-P-P-P-P-P-P-P-P-P-P-P-P-P-31 |
| PtrPPR197 | Potri.005G050400.1 | POPTR_1306s00200.1 | Chr05 | - | 3625397 | 3627229 | 1 | exon | (3625397-3627229) | 610 | P | 14 | 94-P-P-P-P-P-P-P-P-P-P-P-P-P-P-26 |
| PtrPPR198 | Potri.005G050500.1 | POPTR_0005s05210.1 | Chr05 | - | 3628552 | 3632619 | 3 | exon-exon-exon | (3630292-3632092)(3632344-3632465)(3632522-3632619) | 673 | P | 14 | 84-P-P-P-P-P-P-P-P-P-P-P-P-P-P-99 |
| PtrPPR199 | Potri.005G053600.1 | POPTR_0005s05510.1 | Chr05 | + | 3822542 | 3824839 | 1 | exon | (3822542-3824489) | 648 | E+ | 16 | 99-P1-L1-S1-P1-L1-S1-SS-P1-L1-S1-P2-L2-S2-E1-E2-E+ |
| PtrPPR200 | Potri.005G060600.1 | POPTR_0005s06190.1 | Chr05 | + | 4253490 | 4255172 | 2 | exon-exon | (4253490-3824007)(3824152-4255172) | 512 | E+ | 14 | 5-L1-S1-P1-L1-S1-P1-L1-S1-P2-L2-S2-E1-E2-E+ |
| PtrPPR201 | Potri.005G066700.1 | POPTR_0005s06780.1 | Chr05 | + | 4799548 | 4801428 | 1 | exon | (4799548-4801195) | 548 | P | 10 | 139-P-P-P-P-P-P-P-P-P-P-48 |
| PtrPPR202 | Potri.005G074000.1 | POPTR_0005s07530.1 | Chr05 | - | 5390492 | 5393549 | 1 | exon | (5390492_5392832) | 779 | E2 | 21 | 63-S1-P1-L1-S1-P1-L1-S1-P1-L1-S1-P1-L1-S1-P1-L1-S1-P2-L2-S2-E1-E2-7 |
| PtrPPR203 | Potri.005G082400.1 | POPTR_0005s08410.1 | Chr05 | + | 6067849 | 6071373 | 2 | exon-exon | (6067976-6068317)(6068911-6070032) | 487 | P | 10 | 92-P-P-P-P-P-P-P-P-P-P-39 |
| PtrPPR204 | Potri.005G090600.1 | POPTR_0005s09280.1 | Chr05 | - | 6747162 | 6750128 | 1 | exon | (6747216-6749720) | 834 | P | 18 | 102-P-P-P-P-P-P-P-33-P-16-P-P-P-P-P-P-P-P-P-P-53 |
| PtrPPR205 | Potri.005G097200.1 | POPTR_0005s09990.1 | Chr05 | + | 7339605 | 7343367 | 3 | exon-exon-exon | (7339605-7339735)(7339852-7340474)(7343155-7343367) | 321 | P | 7 | 60-P-P-P-P-P-P-P-15 |
| PtrPPR206 | Potri.005G102400.1 | POPTR_0005s10540.1 | Chr05 | - | 7840902 | 7842802 | 3 | exon-exon-exon | (7840902-7841341)(7841444-7842624)(7842714-7842802) | 569 | P | 13 | 23-P-P-35-P-P-P-P-P-P-P-P-P-P-P-58 |
| PtrPPR207 | Potri.005G103300.1 | POPTR_0005s10620.1 | Chr05 | + | 7905317 | 7907565 | 1 | exon | (7905402-7906790) | 462 | P | 11 | 45-P-P-P-P-P-P-P-P-P-P-P-24 |
| PtrPPR208 | Potri.005G109800.1 | POPTR_0005s11170.1 | Chr05 | + | 8451144 | 8453489 | 1 | exon | (8451385-8453079) | 564 | E2 | 12 | 111-P1-L1-S1-SS-P1-L1-S1-P2-L2-S2-E1-E2-37 |
| PtrPPR209 | Potri.005G119000.1 | POPTR_0005s12100.1 | Chr05 | + | 9231129 | 9233168 | 1 | exon | (9231205-9233168) | 654 | DYW | 13 | 110-P1-L1-S1-SS-P1-L1-S1-P2-L2-S2-E1-E2-DYW |
| PtrPPR210 | Potri.005G125400.1 | POPTR_0005s12690.1 | Chr05 | - | 9793354 | 9794547 | 1 | exon | (9793354-9794547) | 397 | P | 5 | 161-P-P-34-P-P-P-27 |
| PtrPPR211 | Potri.005G127500.1 | POPTR_0005s12880.1 | Chr05 | + | 10046632 | 10049406 | 2 | exon-exon | (10046717-10047479)(10048178-10049178) | 587 | P | 8 | 135-P-P-P-P-P-152-P-P-P-22 |
| PtrPPR212 | Potri.005G128600.1 | POPTR_0005s12980.1 | Chr05 | + | 10166998 | 10169318 | 1 | exon | (10166998-10168978) | 659 | P | 11 | 135-P-P-P-P-P-104-P-P-P-P-P-P-32 |
| PtrPPR213 | Potri.005G131800.1 | POPTR_0005s14010.1 | Chr05 | - | 10642653 | 10644764 | 1 | exon | (10642764-10644596) | 610 | E2 | 18 | S1-P1-L1-S1-P1-L1-S1-P1-L1-S1-P1-L1-S1-P2-L2-S2-E1-E2-11 |
| PtrPPR214 | Potri.005G137900.1 | POPTR_0005s18110.1 | Chr05 | - | 11412096 | 11415391 | 2 | exon-exon | (11412402-11414313) (11414440-11415314) | 928 | E2 | 24 | 82-P1-L1-S1-P1-L1-S1-P1-L1-S1-P1-L1-S1-P1-L1-S1-P1-19-P1-L1-S1-P2-L2-S2-E1-E2-16 |
| PtrPPR215 | Potri.005G147200.1 | POPTR_0005s19090.1 | Chr05 | + | 12974311 | 12976081 | 1 | exon | (12974311-2823448) | 439 | P | 10 | 43-P-P-P-P-P-P-P-P-32-P-P-10 |
| PtrPPR216 | Potri.005G151200.1 | POPTR_0005s13290.1 | Chr05 | - | 13857185 | 13860046 | 2 | exon-exon | (13857185-13857269)(13857579-13859786) | 763 | DYW | 17 | 69-L1-S1-P1-L1-S1-P1-L1-S1-SS-L1-S1-P2-L2-S2-E1-E2-DYW |
| PtrPPR217 | Potri.005G155000.1 |  | Chr05 | + | 15078656 | 15080677 | 1 | exon | (15078656-15080677) | 673 | DWY | 17 | L1-S1-P1-L1-S1-P1-L1-S1-SS-L1-S1-P2-L2-S2-E1-E2-DYW |
| PtrPPR218 | Potri.005G160100.1 | POPTR_0005s17540.1 | Chr05 | - | 16101956 | 16103442 | 1 | exon | (16101956-16103233) | 408 | P | 8 | 43-P-P-P-P-35-P-P-P-38-P-11 |
| PtrPPR219 | Potri.005G170300.1 | POPTR_0005s16420.1 | Chr05 | - | 18453037 | 18455642 | 2 | exon-exon | (18453037-18454578)(18455607-18455642) | 525 | PLS | 14 | 30-SS-P1-L1-S1-P1-L1-S1-P1-L1-S1-P1-L1-S1-P2-26 |
| PtrPPR220 | Potri.005G181900.1 | POPTR_0005s20430.1 | Chr05 | - | 19840089 | 19841886 | 1 | exon | (19840352-19841788) | 478 | P | 10 | 93-P-P-P-P-P-P-P-P-P-P-38 |
| PtrPPR221 | Potri.005G185300.1 | POPTR_0005s20740.1 | Chr05 | + | 20261448 | 20263587 | 1 | exon | (20261504-20263396) | 630 | E+ | 17 | 15-L1-S1-SS-P1-33-S1-P1-L1-S1-P1-L1-S1-P2-L2-S2-E1-E2-E+ |
| PtrPPR222 | Potri.005G187800.1 | POPTR_0005s21020.1 | Chr05 | - | 20523141 | 20527182 | 1 | exon | (20523141-20526222) | 1026 | E+ | 27 | 58-L1-S1-P1-L1-S1-P1-P1-P1-L1-S1-P1-L1-S1-P1-L1-S1-P1-L1-S1-P1-L1-S1-P2-L2-S2-E1-E2-E+ |
| PtrPPR223 | Potri.005G199800.1 | POPTR_0005s22150.1 | Chr05 | + | 21512218 | 21514046 | 1 | exon | (21512218-21513955) | 578 | DYW | 12 | 69-P1-L1-S1-P1-L1-S1-P2-L2-S2-E1-E2-DYW |
| PtrPPR224 | Potri.005G202600.1 | POPTR_0005s22430.1 | Chr05 | + | 21762907 | 21764876 | 1 | exon | (21763103-21764632) | 509 | E+ | 15 | L1-S1-P1-L1-S1-SS-P1-L1-S1-P2-L2-S2-E1-E2-E+ |
| PtrPPR225 | Potri.005G208900.1 | POPTR_0005s23050.1 | Chr05 | - | 22283813 | 22286022 | 2 | exon-exon | (22283920-22284198)(22284323-22285585) | 513 | P | 10 | 71-P-34-P-P-P-P-P-P-P-37-P-P-23 |
| PtrPPR226 | Potri.005G212400.1 | POPTR_0005s23410.1 | Chr05 | + | 22585442 | 22588129 | 1 | exon | (22585442-22587695) | 750 | P | 10 | 54-P-128-P-38-P-128-P-P-P-P-P-P-P-45 |
| PtrPPR227 | Potri.005G215500.1 | POPTR_0005s23690.1 | Chr05 | + | 22848668 | 22851541 | 1 | exon | (22848668-22851541) | 957 | DYW | 23 | 77-L1-S1-P1-L1-S1-P1-L1-S1-P1-L1-S1-P1-L1-S1-P1-L1-S1-P2-L2-S2-E1-E2-DYW |
| PtrPPR228 | Potri.005G220100.1 | POPTR_0005s24150.1 | Chr05 | + | 23184687 | 23186371 | 3 | exon-exon-exon | (23184687-23185039)(23185091-23185308)(23185983-23186371) | 319 | P | 6 | 71-P-17-P-P-P-P-P-15 |
| PtrPPR229 | Potri.005G228200.1 | POPTR_0005s24980.1 | Chr05 | + | 23769812 | 23772901 | 2 | exon-exon | (23770114-23771214)(23771428-23772474) | 715 | P | 8 | 381-P-P-P-P-P-P-P-35-P-19 |
| PtrPPR230 | Potri.005G245400.1 | POPTR_0005s26670.1 | Chr05 | - | 24980075 | 24981931 | 1 | exon | (24980126-24981727) | 533 | P | 11 | 146-P-P-P-P-P-P-P-P-P-P-P-2 |
| PtrPPR231 | Potri.005G245500.1 | POPTR_0005s26680.1 | Chr05 | - | 24982510 | 24984407 | 3 | exon-exon-exon | (24982510-24983140)(24983197-24983607)(24983685-24984134) | 496 | P | 11 | 106-P-P-P-15-P-P-P-P-P-P-P-P-2 |
| PtrPPR232 | Potri.005G247200.1 | POPTR_0005s26850.1 | Chr05 | - | 25100746 | 25104040 | 1 | exon | (25100775-25102466) | 563 | P | 11 | 166-P-P-P-P-P-P-P-P-P-P-P-12 |
| PtrPPR233 | Potri.005G250200.1 | POPTR_0005s27160.1 | Chr05 | + | 25302366 | 25306628 | 4 | exon-exon-exon-exon | (25302400-25304193)(25304782-25304934)(25305018-25305089)(25305910-25306020) | 709 | P | 15 | 167-P-P-P-P-P-P-P-P-P-P-P-P-P-P-P-20 |
| PtrPPR234 | Potri.005G252300.1 | POPTR_0005s27370.1 | Chr05 | - | 25433387 | 25436748 | 2 | exon-exon | (25433677-25435154)(25436324-25436381) | 511 | P | 5 | 266-P-37-P-P-P-P-33 |
| PtrPPR235 | Potri.006G001200.1 | POPTR_0006s00330.1 | Chr06 | - | 114978 | 119754 | 1 | exon | (114994-116766) | 590 | DYW | 14 | 14-L1-S1-P1-L1-S1-P1-L1-S1-P2-L2-S2-E1-E2-DYW |
| PtrPPR236 | Potri.006G005100.1 | POPTR_0006s00700.1 | Chr06 | + | 355303 | 359071 | 1 | exon | (356308-358437) | 709 | P | 20 | 14-P-P-P-P-P-P-P-P-P-P-P-P-P-P-P-P-P-P-P-P |
| PtrPPR237 | Potri.006G006500.1 | POPTR_0006s00830.1 | Chr06 | - | 445611 | 447245 | 1 | exon | (445875-447229) | 454 | P | 12 | 6-P-P-P-P-P-P-P-P-P-P-P-P-35 |
| PtrPPR238 | Potri.006G007800.1 | POPTR_0006s00960.1 | Chr06 | - | 525043 | 528796 | 1 | exon | (525054-527521) | 822 | E+ | 22 | 43-SS-P1-L1-S1-P1-L1-S1-P1-L1-S1-P1-L1-S1-P1-L1-S1-P2-L2-S2-E1-E2-E+ |
| PtrPPR239 | Potri.006G015900.1 | POPTR_0006s01700.1 | Chr06 | + | 1085218 | 1088779 | 1 | exon | (1085349-1087523) | 724 | P | 18 | 103-P-P-P-P-P-P-P-P-P-P-P-P-P-P-P-P-P-P |
| PtrPPR240 | Potri.006G018300.1 | POPTR_0006s01920.1 | Chr06 | - | 1191570 | 1193710 | 3 | exon-exon-exon | (1191570-1191676)(1191708-1192405)(1193673-1193710) | 280 | P | 7 | 10-P-P-P-P-P-P-P-34 |
| PtrPPR241 | Potri.006G018600.1 | POPTR_0006s01950.1 | Chr06 | - | 1210902 | 1213518 | 3 | exon-exon-exon | (1210902-1211317)(1211436-1212038)(1212097-1212986) | 635 | P | 9 | 129-P-P-P-P-P-P-16-P-P-66-P-109 |
| PtrPPR242 | Potri.006G018800.1 |  | Chr06 | - | 1256197 | 1256998 | 3 | exon-exon-exon | (1256197-1256498)(1256555-1256682)(1256721-1256963) | 223 | P | 4 | 16-P-P-13-P-25-P-29 |
| PtrPPR243 | Potri.006G019000.1 | POPTR_0006s01990.1 | Chr06 | - | 1265592 | 1268396 | 1 | exon | (1265646-1267748) | 700 | P | 10 | 175-P-P-P-P-P-P-P-P-P-66-P-109 |
| PtrPPR244 | Potri.006G039600.1 | POPTR_0006s03790.1 | Chr06 | - | 2811613 | 2815493 | 3 | exon-exon-exon | (2811732-2811766)(2812577-2813165)(2813855-2815102) | 623 | P | 7 | 255-P-34-P-P-32-P-P-P-P-56 |
| PtrPPR245 | Potri.006G049400.1 | POPTR_0006s04800.1 | Chr06 | + | 3572786 | 3575302 | 1 | exon | (3572786-3575302) | 838 | P | 17 | 125-P-P-P-P-P-P-P-P-P-P-13-P-P-P-P-34-P-P-P-68 |
| PtrPPR246 | Potri.006G049900.1 | POPTR_0006s04840.1 | Chr06 | - | 3585352 | 3590231 | 9 | exon-exon-exon-exon-exon-exon-exon-exon-exon | (3585352-3585715)(3585935-3586107)(3586424-3586529)(3587245-3588354)(3588442-3588547)(3588642-3588846)(3588981-3589113)(3589353-3589457)(3590083-3590231) | 816 | P | 16 | 148-P-P-P-P-P-P-P-P-P-35-P-P-P-P-P-35-P-P-36 |
| PtrPPR247 | Potri.006G053100.1 | POPTR_0006s05170.1 | Chr06 | + | 3831484 | 3834966 | 2 | exon-exon | (3831484-3831627)(3832897-3834966) | 737 | E+ | 19 | 46-L1-S1-P1-L1-S1-31-L1-S1-P1-L1-S1-P1-L1-S1-P2-L2-S2-E1-E2-E+ |
| PtrPPR248 | Potri.006G057000.1 |  | Chr06 | - | 4153756 | 4154604 | 4 | exon-exon-exon-exon | (4153756-4153903)(4153942-4154117)(4154153-4154304)(4154394-4154604) | 228 | E1 | 3 | 18-S1-17-L2-19-E1-73 |
| PtrPPR249 | Potri.006G068700.1 | POPTR_0006s06790.1 | Chr06 | + | 5157238 | 5158715 | 1 | exon | (5157244-5158715) | 490 | P | 10 | 155-P-P-P-P-P-P-P-P-P-P |
| PtrPPR250 | Potri.006G076100.1 | POPTR_0006s07570.1 | Chr06 | - | 5714452 | 5716480 | 1 | exon | (5714452-5716204) | 583 | E+ | 17 | 2-L1-S1-P1-L1-S1-P1-L1-S1-P1-L1-S1-P2-L2-S2-E1-E2-E+ |
| PtrPPR251 | Potri.006G078400.1 | POPTR_0006s07800.1 | Chr06 | + | 5939935 | 5940783 | 2 | exon-exon | forward(5939935-5940381)(5940676-5940783) | 184 | P | 4 | 16-P-P-P-P-33 |
| PtrPPR252 | Potri.006G090900.1 | POPTR_0006s09190.1 | Chr06 | - | 6956237 | 6958132 | 1 | exon | (6956237..6958132) | 631 | E+ | 17 | 31-L1-S1-P1-L1-S1-P1-L1-S1-P1-L1-S1-P2-L2-S2-E1-E2-E+ |
| PtrPPR253 | Potri.006G091500.1 | POPTR_0006s09260.1 | Chr06 | - | 6985339 | 6993522 | 7 | exon-exon-exon-exon-exon-exon-exon | (6985585-6985587)(6986962-6987096)(6987239-6987337)(6987847-6988038)(6990466-6990678)(6991561-6991762)(6993010-6993026) | 286 | P | 3 | 121-P-P-P-59 |
| PtrPPR254 | Potri.006G105700.1 | POPTR_0006s10640.1 | Chr06 | - | 8154913 | 8157233 | 2 | exon-exon | (8155097-8155498)(8155589-8157145) | 652 | DYW | 15 | 6-L1-S1-P1-36-P1-L1-S1-P1-L1-S1-P2-L2-S2-E1-E2-DYW |
| PtrPPR255 | Potri.006G106400.1 | POPTR_0006s10700.1 | Chr06 | - | 8224129 | 8227530 | 1 | exon | (8224129-8225650) | 506 | P | 10 | 112-P-P-P-P-P-P-P-P-P-P-41 |
| PtrPPR256 | Potri.006G124900.1 | POPTR_0006s12670.1 | Chr06 | + | 10037158 | 10041203 | 1 | exon | (10037273-10040047) | 924 | P | 22 | 139-P-P-P-P-P-P-P-P-P-P-P-P-P-P-P-P-P-P-P-P-P-P-17 |
| PtrPPR257 | Potri.006G132700.1 | POPTR_0006s13500.1 | Chr06 | - | 10906198 | 10912199 | 12 | exon-exon-exon-exon-exon-exon-exon-exon-exon-exon-exon-exon | (10906220-10906766)(10906906-10906993)(10907147-10907354)(10908046-10908153)(10908645-10908809)(10908985-10909566)(10910073-10910138)(10910275-10910355)(10910484-10910581)(10910916-10911126)(10911365-10911425)(10911576-10911913) | 850 | P | 10 | 221-P-P-33-P-P-P-P-41-P-P-P-P-198 |
| PtrPPR258 | Potri.006G141800.1 | POPTR_0006s14410.1 | Chr06 | - | 11963843 | 11967193 | 2 | exon-exon | (11963843-11963954)(11964054-11965478) | 511 | P | 11 | 72-P-35-P-P-P-P-P-P-P-P-P-P-19 |
| PtrPPR259 | Potri.006G155400.1 | POPTR_0006s16020.1 | Chr06 | + | 13986855 | 13991462 | 2 | exon-exon | (13986855-13986938)(13988698-13990624) | 669 | PLS | 16 | 133-L1-S1-P1-L1-S1-P1-L1-S1-P1-L1-S1-P1-P1-P1-L1-S1 |
| PtrPPR260 | Potri.006G157400.1 | POPTR_0006s16250.1 | chr06 | - | 14172558 | 14174368 | 2 | exon-exon | (14660142-14660369)(14660708-14661952) | 490 | P | 7 | 122-P-P-P-P-P-P-P-122 |
| PtrPPR261 | Potri.006G159700.1 |  | Chr06 | - | 15065852 | 15068619 | 2 | exon-exon | (15066576-15066803)(15067142-15068386) | 490 | P | 8 | 122-P-P-P-P-P-P-P-41-P-46 |
| PtrPPR262 | Potri.006G160300.1 | POPTR_0006s16330.1 | Chr06 | - | 15126558 | 15132433 | 7 | exon-exon-exon-exon-exon-exon-exon-exon | (15126600-15128426)(15128527-15128616)(15128706-15128887)(15129103-15129132)(15129330-15129398)(15131091-15131226)(15132072-15132310) | 887 | P | 4 | 196-P-136-P-P-P-415 |
| PtrPPR263 | Potri.006G164100.1 | POPTR_0006s17740.1 | Chr06 | + | 16513791 | 16517388 | 1 | exon | (16513791-16515213) | 473 | E2 | 11 | 87-P1-L1-S1-P1-L1-S1-P2-L2-S2-E1-E2-11 |
| PtrPPR264 | Potri.006G166200.1 | POPTR_0006s17990.1 | Chr06 | + | 16889189 | 16893729 | 2 | exon-exon | (16889403-16889643)(16889695-16891919) | 821 | P | 17 | 208-P-P-P-P-P-P-P-P-P-P-P-P-P-P-P-P-P-17 |
| PtrPPR265 | Potri.006G180400.1 | POPTR_0006s19410.1 | Chr06 | + | 19456232 | 19456420 | 1 | exon | (19456232-19456420) | 62 | P | 2 | P-P-11 |
| PtrPPR266 | Potri.006G181800.1 | POPTR_0006s19570.1 | Chr06 | + | 19612555 | 19615345 | 1 | exon | (19612689-19615091) | 800 | DYW | 18 | 87-P1-L1-S1-P1-L1-S1-P1-L1-S1-P1-L1-S1-P2-L2-S2-E1-E2-DYW |
| PtrPPR267 | Potri.006G197800.1 |  | Chr06 | + | 21329984 | 21330999 | 3 | exon-exon-exon | (21329984-21330366)(21330420-21330648)(21330670-21330999) | 313 | P | 5 | 24-P-36-P-18-P-26-P-P-31 |
| PtrPPR268 | Potri.006G200800.1 | POPTR_0006s21510.1 | Chr06 | - | 21578127 | 21581329 | 1 | exon | (21578127-21581274) | 1048 | P | 19 | 203-P-P-P-P-P-P-P-P-P-P-143-P-P-P-P-P-P-P-P-P-37 |
| PtrPPR269 | Potri.006G207500.1 | POPTR_0006s22400.1 | Chr06 | - | 22290455 | 22292595 | 2 | exon-exon | (22290464-22291471)(22291514-22292155) | 549 | PLS | 12 | 84-P1-L1-S1-SS-SS-SS-P1-L1-18-S1-SS-SS-P2-44 |
| PtrPPR270 | Potri.006G211400.1 | POPTR_0006s22800.1 | Chr06 | - | 22564953 | 22566474 | 2 | exon-exon | (22564953-22566177)(22566245-22566280) | 419 | E1 | 12 | 9-L1-S1-P1-L1-S1-P1-L1-S1-P2-L2-S2-E1-3 |
| PtrPPR271 | Potri.006G213200.1 | POPTR_0006s22970.1 | Chr06 | + | 22683910 | 22685505 | 1 | exon | (22683936-22685489) | 517 | E+ | 14 | 39-S1-P1-L1-S1-SS-P1-L1-S1-P2-L2-S2-E1-E2-E+ |
| PtrPPR272 | Potri.006G215900.1 | POPTR_0006s23240.1 | Chr06 | - | 22898884 | 22899738 | 2 | exon-exon | (22898884-22899237)(22899460-22899738) | 210 | E2 | 4 | 46-S1-P2-18-E2-E+ |
| PtrPPR273 | Potri.006G216100.1 | POPTR_0006s23260.1 | Chr06 | + | 22903465 | 22905423 | 2 | exon-exon | (22903465-22905211)(22905305-22905423) | 621 | E2 | 14 | 16-L1-S1-P1-L1-S1-P1-L1-S1-SS-P2-101-L2-S2-E1-E2-27 |
| PtrPPR274 | Potri.006G223300.1 | POPTR_0006s23980.1 | Chr06 | + | 23561487 | 23563184 | 1 | exon | (23561487-23563184) | 565 | P | 10 | 197-P-P-P-P-P-P-P-P-P-P-19 |
| PtrPPR275 | Potri.006G231200.1 | POPTR_0006s24730.1 | Chr06 | + | 24170290 | 24174471 | 1 | exon | (24170290-24172099) | 602 | DYW | 13 | 61-P1-L1-S1-SS-P1-L1-S1-P2-L2-S2-E1-E2-DYW |
| PtrPPR276 | Potri.006G242200.1 | POPTR_0006s25890.1 | Chr06 | - | 25089445 | 25091217 | 1 | exon | (25089445-25091217) | 590 | P | 13 | 129-P-P-P-P-P-P-P-P-P-P-P-P-P-6 |
| PtrPPR277 | Potri.006G242500.1 | POPTR_0006s25910.1 | Chr06 | - | 25097642 | 25099556 | 1 | exon | (25097764-25099556) | 597 | P | 15 | 65-P-P-P-P-P-P-P-P-P-P-P-P-P-P-P-6 |
| PtrPPR278 | Potri.006G243000.1 | POPTR_0006s25960.1 | Chr06 | - | 25108528 | 25110984 | 2 | exon-exon | (25108593-25108871)(25109375-25110370) | 424 | P | 7 | 109-P-P-P-P-P-P-72-P |
| PtrPPR279 | Potri.006G244500.1 | POPTR_0006s26090.1 | Chr06 | - | 25237632 | 25239593 | 1 | exon | (25237632..25239593 ) | 653 | DYW | 16 | 6-S1-P1-L1-S1-P1-L1-S1-P1-L1-S1-P2-L2-S2-E1-E2-DYW |
| PtrPPR280 | Potri.006G245300.1 | POPTR_0006s26170.1 | Chr06 | - | 25307271 | 25310026 | 1 | exon | (25307271-25309836) | 854 | DYW | 19 | 97-P1-L1-S1-P1-L1-S1-SS-P1-L1-S1-SS-P1-L1-S1-P2-L2-S2-E1-16-DYW |
| PtrPPR281 | Potri.006G247400.1 | POPTR_0006s26360.1 | Chr06 | + | 25440008 | 25443382 | 2 | exon-exon | (25440049-25440643)(25441212-25442440) | 607 | P | 11 | 146-P-P-P-P-P-P-P-P-P-P-P-67 |
| PtrPPR282 | Potri.006G250700.1 | POPTR_0006s26690.1 | Chr06 | - | 25678069 | 25680383 | 2 | exon | (25678078-25679569)(25679675-25679916) | 577 | P | 17 | P-P-P-P-P-P-P-P-P-P-P-P-P-P-P-P-P |
| PtrPPR283 | Potri.006G252400.1 | POPTR_0006s26860.1 | Chr06 | + | 25789326 | 25793062 | 2 | exon-exon | (25789326-25789350)(25791375-25793062) | 570 | DYW | 11 | 92-L1-S1-P1-L1-S1-P2-L2-S2-E1-E2-DYW |
| PtrPPR284 | Potri.006G257300.1 | POPTR_0006s27340.1 | Chr06 | + | 26142511 | 26146892 | 1 | exon | (26142694-26144550) | 618 | P | 15 | 87-P-P-P-P-P-P-P-P-P-P-P-P-P-P-P-5 |
| PtrPPR285 | Potri.006G264800.1 | POPTR_0006s28060.1 | Chr06 | + | 26758505 | 26762948 | 1 | exon | (26758841-26762188) | 1115 | P | 27 | 123-P-P-P-P-P-P-P-P-P-P-P-P-P-P-P-P-P-36-P-P-P-P-P-P-P-P-P-P-11 |
| PtrPPR286 | Potri.006G265200.1 | POPTR_0006s28100.1 | Chr06 | - | 26780930 | ..26782815 | 2 | exon-exon | (26781011-26781686)(26781798-26782513) | 463 | E2 | 13 | 14-P1-L1-S1-SS-SS-SS-SS-SS-P2-L2-S2-E1-E2-19 |
| PtrPPR287 | Potri.006G271200.1 | POPTR_0006s28580.1 | Chr06 | - | 27265727 | 27267859 | 1 | exon | (27266512-27267723) | 403 | P | 12 | P-P-P-P-P-P-P-P-P-P-P-P-9 |
| PtrPPR288 | Potri.006G271400.1 | POPTR_0006s28610.1 | Chr06 | - | 27273343 | 27276078 | 2 | exon-exon | (27273343-27275128)(27275977-27276054) | 620 | P | 14 | 56-P-36-P-P-P-P-P-P-P-P-P-P-P-P-P-38 |
| PtrPPR289 | Potri.006G273400.1 | POPTR_0006s28820.1 | Chr06 | + | 27430807 | 27436475 | 11 | exon-exon-exon-exon-exon-exon-exon-exon-exon-exon-exon | (27430907-27431311)(27431404-27431536)(27431850-27431947)(27432050-27432211)(27432327-27432395)(27432617-27432752)(27434502-27434735)(27434895-27434972)(27435369-27435437)(27435787-27435963)(27436098-27436376) | 612 | P | 8 | 90-P-P-P-P-P-P-P-P-221 |
| PtrPPR290 | Potri.007G004900.1 | POPTR_0007s14930.1 | Chr07 | + | 366115 | 371138 | 9 | exon-exon-exon-exon-exon-exon-exon-exon-exon | (366150-366709)(366794-366985)(367687-367768)(367872-368063)(368160-368273)(368883-368976)(369066-369223)(369394-369807)(369942-370775) | 879 | P | 10 | 212-P-P-P-15-P-P-P-P-P-P-P-293 |
| PtrPPR291 | Potri.007G021700.1 | POPTR_0007s13220.1 | Chr07 | - | 1654116 | 1657053 | 2 | exon-exon | (1654116-1656113)(1656192-1656198) | 667 | E+ | 19 | SS-P1-L1-S1-P1-L1-S1-P1-L1-S1-P1-L1-S1-P2-L2-S2-E1-E2-E+ |
| PtrPPR292 | Potri.007G022500.1 | POPTR_0007s13130.1 | Chr07 | - | 1694575 | 1696869 | 2 | exon-exon | (1694617-1695250)(1695389-1696239) | 494 | E2 | 13 | 25-SS-P1-L1-S1-P1-L1-S1-34-SS-P2-L2-S2-E1-E2-10 |
| PtrPPR293 | Potri.007G028400.1 | POPTR_0007s12560.1 | Chr07 | - | 2130192 | 2131823 | 1 | exon | (2130281-2131471) | 396 | P | 7 | 89-P-P-P-P-34-P-P-P-27 |
| PtrPPR294 | Potri.007G050200.1 | POPTR_0007s10370.1 | Chr07 | - | 4808493 | 4810741 | 1 | exon | (4808602-4810497) | 631 | DYW | 14 | 29-L1-S1-P1-31-S1-SS-P1-L1-S1-P2-L2-S2-E1-E2-DYW |
| PtrPPR295 | Potri.007G059500.1 | POPTR_0007s09390.1 | Chr07 | + | 6400395 | 6401501 | 2 | exon-exon | (6400395-6400440)(6400987-6401501) | 186 | P | 5 | 7-P-P-P-P-P-3 |
| PtrPPR296 | Potri.007G065900.1 | POPTR_0007s08370.1 | Chr07 | + | 8385936 | 8387177 | 4 | exon-exon-exon-exon | (8385936-8386038)(8386125-8386312)(8386508-8386677)(8387165-8387177 ) | 157 | P | 4 | 9-P-P-P-P-15 |
| PtrPPR297 | Potri.007G068400.1 | POPTR_0007s08110.1 | Chr07 | - | 8958194 | 8959840 | 1 | exon | (8958314-8959588) | 424 | P | 11 | 4-P-P-P-P-P-P-P-P-P-P-P-35 |
| PtrPPR298 | Potri.007G068600.1 | POPTR_0007s08080.1 | Chr07 | + | 8971572 | 8974332 | 1 | exon | (8971572-8974299) | 908 | DWY | 24 | P1-L1-S1-P1-L1-S1-P1-L1-S1-P1-L1-S1-P1-L1-S1-P1-L1-S1-P2-L2-S2-E1-E2-DYW |
| PtrPPR299 | Potri.007G068900.1 | POPTR_0007s08060.1 | Chr07 | + | 8996188 | 8998149 | 2 | exon-exon | (8996223-8996898)(8997007-8998082) | 583 | E2 | 16 | 28-L1-S1-P1-L1-S1-P1-L1-S1-P1-L1-S1-P2-L2-S2-E1-E2-14 |
| PtrPPR300 | Potri.007G073100.1 | POPTR_0007s07610.1 | Chr07 | - | 9578902 | 9580598 | 1 | exon | (9578928-9580547) | 539 | E+ | 13 | 75-P1-L1-S1-SS-P1-L1-S1-P2-L2-S2-E1-E2-E+ |
| PtrPPR301 | Potri.007G079400.1 | POPTR_0007s06780.1 | Chr07 | - | 10337653 | 10339329 | 1 | exon | (10337653-10339329) | 558 | P | 9 | 159-P-36-P-P-P-P-P-P-P-P-52 |
| PtrPPR302 | Potri.007G084300.1 |  | Chr07 | + | 11001235 | 11003155 | 4 | exon-exon-exon-exon | (11001235-11001319)(11001536-11001723)(11001836-11001861)(11002949-11003124) | 157 | P | 2 | 81-P-P-6 |
| PtrPPR303 | Potri.007G085500.1 | POPTR_0007s06200.1 | Chr07 | + | 11096707 | 11099063 | 1 | exon | (11096821-11099063) | 747 | DYW | 19 | 9-S1-SS-P1-L1-S1-SS-P1-L1-S1-SS-P1-L1-S1-P2-L2-S2-E1-E2-DYW |
| PtrPPR304 | Potri.007G104700.1 | POPTR_0007s04280.1 | Chr07 | + | 12925630 | 12927901 | 1 | exon | (12925630-12927763) | 710 | E+ | 19 | 23-L1-S1-SS-P1-L1-S1-P1-L1-S1-SS-P1-L1-S1-P2-L2-S2-E1-E2-E+ |
| PtrPPR305 | Potri.007G123600.1 | POPTR_0007s02430.1 | Chr07 | + | 14128316 | 14132447 | 2 | exon-exon | (14128392-14131394)(14132404-14132447) | 1015 | P | 21 | 217-P-P-P-P-P-P-P-P-P-P-P-P-17-P-P-P-P-P-P-33-P-P-P-9 |
| PtrPPR306 | Potri.007G123900.1 | POPTR_0007s02400.1 | Chr07 | - | 14150768 | 14157603 | 4 | exon-exon-exon-exon | (14150768-14154112)(14154206-14154406)(14154913-14155012)(14155057-14155602) | 1396 | P | 27 | 135-P-P-P-P-P-P-P-P-P-P-P-31-P-P-P-P-P-P-P-P-P-P-P-P-P-P-P-P-283 |
| PtrPPR307 | Potri.007G134300.1 | POPTR_0007s01450.1 | Chr07 | - | 14758572 | 14761378 | 3 | exon-exon-exon | (14758694-14759011)(14759701-14760093)(14760949-14760978) | 246 | P | 4 | 84-P-P-P-P-20 |
| PtrPPR308 | Potri.007G146800.1 | POPTR_0007s00250.1 | Chr07 | + | 15555245 | 15557551 | 1 | exon | (15555260-15557395) | 711 | E+ | 19 | 35-SS-P1-L1-S1-P1-L1-S1-P1-L1-S1-P1-L1-S1-P2-L2-S2-E1-E2-E+ |
| PtrPPR309 | Potri.008G003600.1 | POPTR_0008s00430.1 | Chr08 | + | 204482 | 208603 | 4 | exon-exon-exon-exon | (204482-204577)(206555-206594)(206693-206763)(207146-208603) | 554 | E+ | 13 | 90-P1-L1-SS-SS-P1-L1-S1-P2-L2-S2-E1-E2-E+ |
| PtrPPR310 | Potri.008G017200.1 |  | Chr08 | + | 911056 | 912487 | 4 | exon-exon-exon-exon | (911056-911165)(911709-911854)(911980-912265)(912328-912487) | 233 | PLS | 3 | S1-SS-L2-155 |
| PtrPPR311 | Potri.008G017400.1 | POPTR_0008s01830.1 | Chr08 | + | 916969 | 924458 | 9 | exon-exon-exon-exon-exon-exon-exon-exon-exon | (916969-917866)(918234-918361)(918803-918943)(919930-920083)(920390-920449)(921419-921847)(922489-922784)(922961-923090)(923635-924192) | 930 | P | 14 | 301-P-P-P-P-P-P-P-P-P-P-P-P-P-P-133 |
| PtrPPR312 | Potri.008G044000.1 | POPTR_0008s04360.1 | Chr08 | - | 2524079 | 2524996 | 2 | exon-exon | (2524079-2524407)(2524456-2524996) | 289 | P | 8 | 3-P-P-P-18-P-P-P-P-P |
| PtrPPR313 | Potri.008G052200.1 | POPTR_0008s05220.1 | Chr08 | + | 3083083 | 3086401 | 1 | exon | (3083083-3085720) | 878 | E2 | 25 | 32-L1-S1-P1-L1-S1-P1-L1-S1-P1-L1-S1-P1-L1-S1-P1-L1-S1-P1-L1-S1-P2-L2-S2-E1-E2-7 |
| PtrPPR314 | Potri.008G083000.1 | POPTR_0008s08250.1 | Chr08 | + | 5224404 | 5225896 | 6 | exon-exon-exon-exon-exon-exon | (5224404-5224756)(5224984-5225088)(5225119-5225263)(5225294-5225381)(5225417-5225695)(5225748-5225896) | 372 | P | 3 | 9-P-P-P-258 |
| PtrPPR315 | Potri.008G092900.1 | POPTR_0008s09240.1 | Chr08 | + | 5824225 | 5826360 | 5 | exon-exon-exon-exon-exon | (5824225-5824361)(5824509-5824638)(5824756-5825071)(5825208-5825589)(5825756-5826093) | 433 | PLS | 2 | 57-S1-30-S1-284 |
| PtrPPR316 | Potri.008G093000.1 | POPTR_0008s09260.1 | Chr08 | - | 5826856 | 5829298 | 1 | exon | reverse(5826856-5829094) | 745 | E2 | 22 | L1-S1-P1-L1-S1-P1-L1-S1-P1-L1-S1-P1-L1-S1-P1-L1-S1-P2-L2-S2-E1-E2-7 |
| PtrPPR317 | Potri.008G095800.1 | POPTR_0008s09530.1 | Chr08 | + | 5994529 | 5997551 | 1 | exon | (5994534-5996499) | 654 | P | 9 | 213-P-35-P-42-P-P-P-P-P-P-P-42 |
| PtrPPR318 | Potri.008G098700.1 | POPTR_0008s09820.1 | Chr08 | + | 6190162 | 6192370 | 1 | exon | (6190940-6192049) | 369 | P | 10 | 14-P-P-P-P-P-P-P-P-P-P-3 |
| PtrPPR319 | Potri.008G102100.1 | POPTR_0008s10170.1 | Chr08 | - | 6485373 | 6486006 | 2 | exon-exon | (6485373-6485763)(6485897-6486006) | 166 | P | 5 | 9-P-P-P-P-P |
| PtrPPR320 | Potri.008G106500.1 | POPTR_0008s10600.1 | Chr08 | + | 6754971 | 6758558 | 1 | exon | (6754994-6756778) | 594 | E+ | 15 | 65-S1-P1-L1-S1-SS-SS-P1-L1-S1-P2-L2-S2-E1-E2-E+ |
| PtrPPR321 | Potri.008G108300.1 | scaffold_8:F:6775184-6777340 | Chr08 | + | 6891727 | 6894566 | 1 | exon | (6891727-6893887) | 719 | P | 15 | 154-P-P-P-P-P-35-P-P-P-P-P-P-P-P-P-P-5 |
| PtrPPR322 | Potri.008G113100.1 | POPTR_0008s11230.1 | Chr08 | - | 7223173 | 7224965 | 5 | exon-exon-exon-exon-exon | (7223225-7223671)(7223710-7223778)(7223890-7224286)(7224443-7224558)(7224682-7224965) | 447 | PLS | 8 | 19-L1-S1-SS-34-P1-L1-S1-P2-L2-125 |
| PtrPPR323 | Potri.008G121400.1 | POPTR_0008s12050.1 | Chr08 | + | 7903148 | 7905035 | 2 | exon-exon | (7903148-7903154)(7903204-7905035 ) | 612 | E+ | 17 | 23-L1-S1-P1-L1-S1-SS-SS-SS-P1-L1-S1-P2-L2-S2-E1-E2-E+ |
| PtrPPR324 | Potri.008G128100.1 | POPTR_0008s12710.1 | Chr08 | + | 8346565 | 8347323 | 2 | exon-exon | (8346895-8346998)(8347062-8347323) | 122 | P | 3 | 27-P-P-P |
| PtrPPR325 | Potri.008G141700.1 | POPTR_0008s14110.1 | Chr08 | + | 9454367 | 9455259 | 2 | exon-exon | (9454367-9454556)(9454718-9455259) | 243 | P | 5 | 85-P-P-P-P-P |
| PtrPPR326 | Potri.008G142900.1 | POPTR_0008s14240.1 | Chr08 | - | 9573922 | 9575936 | 2 | exon-exon | (9573922-9574317)(9574399-9575734) | 576 | P | 12 | 131-P-P-P-P-P-P-P-P-P-P-P-P-21 |
| PtrPPR327 | Potri.008G143900.1 | POPTR_0008s14290.1 | Chr08 | + | 9665648 | 9666783 | 3 | exon-exon-exon | (9665648-9665802)(9665850-9666349)(9666500-9666783) | 312 | P | 4 | 73-P-P-P-P-99 |
| PtrPPR328 | Potri.008G153700.1 | POPTR_0008s15360.1 | Chr08 | - | 10425381 | 10426463 | 2 | exon-exon | (10425381-10425959)(10426016-10426295) | 285 | PLS | 6 | 6-L1-S1-P1-L1-80-S1-P2 |
| PtrPPR329 | Potri.008G154300.1 | POPTR_0008s15410.1 | Chr08 | + | 10451523 | 10453646 | 5 | exon-exon-exon-exon-exon | (10451523-10451705)(10451734-10451930)(10452049-10452169)(10452327-10452551)(10453451-10453646) | 306 | P | 5 | 60-L1-S1-38-SS-P1-L1-44 |
| PtrPPR330 | Potri.008G160300.1 | POPTR_0008s16020.1 | Chr08 | - | 10888283 | 10889480 | 3 | exon-exon-exon | (10888283-10888359)(10888434-10888720)(10889137-10889436) | 220 | P | 5 | 40-P-P-P-P-P-5 |
| PtrPPR331 | Potri.008G173500.1 | POPTR_0008s17360.1 | Chr08 | + | 11843604 | 11845630 | 6 | exon-exon-exon-exon-exon-exon | (11843604-11843626)(11843943-11844082)(11844126-11844215)(11844257-11844442)(11844654-11845287)(11845336-11845630) | 457 | E2 | 10 | 55-P1-20-S1-SS-P1-L1-S1-P2-L2-16-E1-E2-21 |
| PtrPPR332 | Potri.008G179200.1 |  | Chr08 | - | 12230577 | 12232388 | 5 | exon-exon-exon-exon-exon | (12230577-12230664)(12230735-12230817)(12230978-12231251)(12231454-12231861)(12232004-12232114) | 320 | P | 4 | 142-P-P-P-P-37 |
| PtrPPR333 | Potri.008G185000.1 | POPTR_0008s18880.1 | Chr08 | - | 12682821 | 12684458 | 1 | exon | (12682821-12684360) | 512 | P | 14 | 8-P-P-P-P-P-P-P-P-P-P-P-P-P-P-13 |
| PtrPPR334 | Potri.008G191200.1 | POPTR_0008s19551.1 | Chr08 | + | 13284005 | 13284803 | 2 | exon-exon | (13284005-13284268)(13284302-13284803) | 235 | P | 5 | 8-P-P-23-P-P-P-29 |
| PtrPPR335 | Potri.008G212000.1 | POPTR_0008s23140.1 | Chr08 | - | 16629029 | 16631215 | 1 | exon | (16629029-16631215) | 728 | E1 | 3 | 561-L2-S2-E1-65 |
| PtrPPR336 | Potri.008G217000.1 | POPTR_0008s22620.1 | Chr08 | - | 17742472 | 17744226 | 1 | exon | (17742472..17744226) | 584 | DYW | 13 | 40-S1-P1-L1-S1-P1-L1-S1-P2-L2-S2-E1-E2-DYW |
| PtrPPR337 | Potri.009G027400.1 | POPTR_0009s03250.1 | Chr09 | + | 3840253 | 3843504 | 1 | exon | (3840253-3842671) | 805 | E2 | 22 | 52-L1-S1-P1-L1-S1-P1-L1-S1-P1-L1-S1-P1-L1-S1-P1-L1-S1-P2-L2-S2-E1-E2-10 |
| PtrPPR338 | Potri.009G030900.1 | POPTR_0009s03620.1 | Chr09 | + | 4144592 | 4146553 | 3 | exon-exon-exon | (4144592-4144595)(4144853-4145173)(4145281-4146531) | 524 | P | 7 | 155-P-35-P-69-P-P-P-P-P-13 |
| PtrPPR339 | Potri.009G035100.1 | POPTR_0009s04000.1 | Chr09 | + | 4495744 | 4498179 | 1 | exon | (4495766-4497619) | 617 | E2 | 16 | 68-L1-S1-P1-L1-S1-P1-L1-S1-P1-L1-S1-P2-L2-S2-E1-E2-7 |
| PtrPPR340 | Potri.009G037600.1 | POPTR_0009s04240.1 | Chr09 | - | 4632498 | 4634621 | 1 | exon | (4632523-4634475) | 650 | DYW | 14 | 69-L1-S1-P1-L1-S1-P1-L1-S1-P2-L2-S2-E1-E2-DYW |
| PtrPPR341 | Potri.009G038200.1 | POPTR_0009s04310.1 | Chr09 | + | 4663962 | 4666059 | 2 | exon-exon | (4664057-4664440)(4664611-4665000) | 257 | P | 2 | 142-P-P-45 |
| PtrPPR342 | Potri.009G044700.1 | POPTR_0009s04930.1 | Chr09 | + | 5094750 | 5097888 | 1 | exon | (5094903-5097119) | 738 | DYW | 18 | 26-L1-S1-P1-L1-S1-P1-L1-S1-SS-P1-L1-S1-P2-L2-S2-E1-E2-DYW |
| PtrPPR343 | Potri.009G053600.1 | POPTR_0009s05820.1 | Chr09 | - | 5749330 | 5750862 | 4 | exon-exon-exon-exon | (5749330-5749440)(5749626-5749965)(5750043-5750433)(5750703-5750862) | 333 | PLS | 7 | 46-L1-S1-SS-SS-P1-L1-S1-46 |
| PtrPPR344 | Potri.009G058300.1 |  | Chr09 | - | 6078663 | 6081852 | 3 | exon-exon-exon | (6078791-6079120)(6079494-6080589)(6081054-6081076) | 482 | P | 11 | 88-P-P-P-P-P-P-P-P-P-P-P-6 |
| PtrPPR345 | Potri.009G058400.1 | POPTR_0009s06340.1 | Chr09 | - | 6108774 | 6110634 | 2 | exon-exon | (6108808-6109137)(6109511-6110634) | 484 | P | 11 | 88-P-P-P-P-P-P-P-P-P-P-P-8 |
| PtrPPR346 | Potri.009G067000.1 | POPTR_0009s07140.1 | Chr09 | - | 6713328 | 6721149 | 2 | exon-exon | (6713328-6713426)(6718818-6721044) | 774 | E2 | 20 | 66-P1-L1-S1-P1-L1-S1-P1-L1-S1-P1-L1-S1-P1-L1-S1-P2-L2-S2-E1-E2-34 |
| PtrPPR347 | Potri.009G067400.1 | POPTR_0009s07170.1 | Chr09 | - | 6738850 | 6740917 | 2 | exon-exon | (6739108-6739108)(6739484-6740539) | 382 | P | 6 | 92-P-70-P-P-P-P-P-10 |
| PtrPPR348 | Potri.009G069600.1 | POPTR_0009s07380.1 | Chr09 | - | 6867517 | 6870067 | 1 | exon | (6867577-6869739) | 720 | P | 17 | 65-P-P-P-P-P-P-P-P-P-P-P-P-P-P-P-P-P-58 |
| PtrPPR349 | Potri.009G071200.1 | POPTR_0009s07550.1 | Chr09 | - | 7012385 | 7014866 | 4 | exon-exon-exon-exon | (7012385-7012532)(7013706-7014052)(7014106-7014248)(7014491-7014866) | 337 | P | 8 | 43-P-P-P-P-P-22-P-P-P-9 |
| PtrPPR350 | Potri.009G075200.1 | POPTR_0009s07900.1 | Chr09 | + | 7299100 | 7303127 | 2 | exon-exon | (7299440-7300168)(7300986-7301759) | 500 | P | 5 | 153-P-P-P-72-P-35-P-57 |
| PtrPPR351 | Potri.009G092100.1 | POPTR_0009s09510.1 | Chr09 | - | 8389078 | 8392924 | 1 | exon | (8389369-8391858) | 829 | P | 19 | 145-P-P-P-P-P-P-P-P-P-P-P-P-P-P-P-P-P-P-P-15 |
| PtrPPR352 | Potri.009G105600.1 | POPTR_0009s10870.1 | Chr09 | - | 9221660 | 9223915 | 1 | exon | (9221660-9223915) | 751 | P | 17 | 131-P-P-P-P-P-P-P-P-P-P-P-P-P-P-P-P-35-P-9 |
| PtrPPR353 | Potri.009G106800.1 | POPTR_0009s11000.1 | Chr09 | - | 9299985 | 9302760 | 2 | exon-exon | (9299985-9300564)(9300881-9302760) | 819 | P | 7 | 207-P-P-79-P-P-P-34-P-P-250 |
| PtrPPR354 | Potri.009G109000.1 | POPTR_0009s11220.1 | Chr09 | - | 9401739 | 9403190 | 5 | exon-exon-exon-exon-exon | (9401739-9402215)(9402296-9402411)(9402446-9402591)(9402764-9402886)(9402931-9403190) | 373 | P | 7 | 101-P-P-P-P-P-P-20-P-P |
| PtrPPR355 | Potri.009G110400.1 | POPTR_0009s11350.1 | Chr09 | - | 9470925 | 9472758 | 2 | exon-exon | (9471499-9472082)(9472149-9472260) | 231 | P | 6 | 10-P-13-P-P-P-P-P |
| PtrPPR356 | Potri.009G113500.1 | POPTR_0009s11640.1 | Chr09 | - | 9640778 | 9644196 | 1 | exon | (9640778-9642812) | 677 | E+ | 19 | 22-S1-SS-P1-L1-S1-SS-P1-L1-S1-SS-P1-L1-S1-P2-L2-S2-E1-E2-E+ |
| PtrPPR357 | Potri.009G139200.1 | POPTR_0009s14110.1 | Chr09 | - | 11126826 | 11128895 | 1 | exon | (11126944-11128895) | 650 | E2 | 18 | 34-SS-SS-SS-SS-SS-SS-SS-SS-SS-SS-P1-L1-S1-P2-L2-S2-E1-E2-12 |
| PtrPPR358 | Potri.009G139300.1 | POPTR_0009s14120.1 | Chr09 | - | 11130907 | 11132806 | 2 | exon-exon | (11131004-11131360)(11131463-11132527) | 473 | P | 3 | 211-P-P-P-157 |
| PtrPPR359 | Potri.009G144700.1 |  | Chr09 | - | 11450685 | 11452716 | 6 | exon-exon-exon-exon-exon-exon | (11450685-11450901)(11450939-11451114)(11451243-11451446)(11451503-11451943)(11452020-11452239)(11452274-11452716) | 484 | E1 | 11 | S1-SS-SS-P1-16-S1-P1-31-S1-63-P2-L2-S2-E1-23 |
| PtrPPR360 | Potri.009G145200.1 | POPTR_0009s14760.1 | Chr09 | - | 11497331 | 11502006 | 6 | exon-exon-exon-exon-exon-exon | (11497354-11498262)(11498984-11499085)(11499206-11499384)(11499641-11499761)(11500819-11500887)(11501038-11501370) | 570 | P | 3 | 18-P-P-35-P-404 |
| PtrPPR361 | Potri.009G162100.1 | POPTR_0009s16360.1 | Chr09 | - | 12459909 | 12464235 | 5 | exon-exon-exon-exon-exon | (12460057-12461070)(12461353-12462041)(12462397-12462710)(12463187-12463257)(12464120-12464235) | 734 | P | 14 | 125-P-40-P-P-P-P-46-P-P-P-P-P-Pi-P-P-P-41 |
| PtrPPR362 | Potri.010G014100.1 | POPTR_0010s01780.1 | Chr10 | + | 1747499 | 1751759 | 2 | exon-exon | (1747603-1747621)(1747828-1750505) | 898 | P | 22 | 124-P-P-P-P-P-P-P-P-P-P-P-P-P-P-P-P-P-P-P-P-P-P-5 |
| PtrPPR363 | Potri.010G018900.1 |  | Chr10 | - | 2594841 | 2596820 | 1 | exon | (2594950-2596820) | 623 | E+ | 18 | 4-SS-P1-L1-S1-SS-P1-L1-S1-SS-P1-L1-S1-P2-L2-S2-E1-E2-E+ |
| PtrPPR364 | Potri.010G020500.1 | POPTR_0010s02060.1 | Chr10 | - | 2896413 | 2898503 | 1 | exon | (2896537-2898408) | 623 | E+ | 18 | 4-SS-P1-L1-S1-SS-P1-L1-S1-SS-P1-L1-S1-P2-L2-S2-E1-E2-E+ |
| PtrPPR365 | Potri.010G029900.1 | POPTR_0010s03090.1 | Chr10 | - | 4335183 | 4337318 | 1 | exon | (4335183-4337318) | 711 | E2 | 19 | 68-L1-S1-SS-P1-L1-S1-SS-P1-L1-S1-SS-P1-L1-S1-P2-L2-S2-E1-E2-8 |
| PtrPPR366 | Potri.010G035700.1 | POPTR_0010s04510.1 | Chr10 | - | 6198788 | 6201143 | 1 | exon | (6198898-6201143) | 748 | P | 15 | 181-P-36-P-P-P-P-P-P-P-P-P-P-P-P-P-P-6 |
| PtrPPR367 | Potri.010G048900.1 | POPTR_0010s05880.1 | Chr10 | + | 7975879 | 7976825 | 3 | exon-exon-exon | (7975879-7975984)(7976208-7976440)(7976526-7976825) | 212 | P | 7 | P-P-P-P-P-P-P |
| PtrPPR368 | Potri.010G055500.1 | POPTR_0010s06570.1 | Chr10 | + | 8537029 | 8538922 | 1 | exon | (8537211-8538752) | 513 | P | 10 | 132-P-P-P-P-P-P-P-P-P-P-30 |
| PtrPPR369 | Potri.010G063900.1 | POPTR_0010s07440.1 | Chr10 | - | 9149228 | 9151849 | 1 | exon | (9149228-9151127) | 632 | E+ | 17 | 26-L1-S1-P1-L1-S1-P1-L1-S1-P1-L1-S1-P2-L2-S2-E1-E2-E+ |
| PtrPPR370 | Potri.010G077400.1 | POPTR_0010s08800.1 | Chr10 | - | 10294464 | 10297710 | 1 | exon | (10294865-10297147) | 760 | P | 16 | 111-P-P-P-P-P-P-P-P-P-P-P-P-P-P-32-P-P-47 |
| PtrPPR371 | Potri.010G078900.1 | POPTR_0010s08940.1 | Chr10 | + | 10396885 | 10399632 | 2 | exon-exon | (10396885-10398521)(10398563-10399464) | 845 | P | 17 | 155-P-P-P-P-21-P-P-P-P-P-P-20-P-P-P-P-P-P-P-55 |
| PtrPPR372 | Potri.010G083700.1 | POPTR_0010s09380.1 | Chr10 | + | 10976079 | 10978862 | 1 | exon | (10976079-10978617) | 845 | DYW | 19 | 103-P1-L1-S1-P1-L1-S1-P1-L1-S1-SS-P1-L1-S1-P2-L2-S2-E1-E2-DYW |
| PtrPPR373 | Potri.010G083800.1 | POPTR_0010s09390.1 | Chr10 | + | 10980261 | 10982569 | 1 | exon | (10980485-10982179) | 564 | P | 10 | 179-P-P-P-P-P-P-P-P-P-P-36 |
| PtrPPR374 | Potri.010G083900.1 | POPTR_0010s09400.1 | Chr10 | + | 10983933 | 10985808 | 1 | exon | (10983933-10985562) | 542 | P | 11 | 159-P-P-P-P-P-P-P-P-P-P-P |
| PtrPPR375 | Potri.010G084000.1 | POPTR_0010s09410.1 | Chr10 | + | 10987837 | 10989729 | 1 | exon | (10987837-10989499) | 553 | P | 10 | 168-P-P-P-P-P-P-P-P-P-P-36 |
| PtrPPR376 | Potri.010G086200.1 | POPTR_0010s09620.1 | Chr10 | - | 11104569 | 11107136 | 2 | exon-exon | (11104569-11105141)(11105214-11106807) | 721 | DYW | 17 | 17-L1-26-P1-L1-S1-P1-L1-S1-SS-P1-L1-S1-P2-L2-S2-E1-E2-DYW |
| PtrPPR377 | Potri.010G086900.1 | POPTR_0010s09690.1 | Chr10 | + | 11133243 | 11136146 | 2 | exon-exon | (11133243-11134673)(11135618-11136083) | 631 | DYW | 15 | 22-P1-L1-S1-P1-L1-S1-P1-L1-S1-P2-L2-S2-E1-E2-DYW |
| PtrPPR378 | Potri.010G098400.1 | POPTR_0010s10870.1 | Chr10 | - | 12016411 | 12020761 | 1 | exon | (12016411-12018757) | 781 | P | 17 | 146-P-18-P-P-P-P-P-P-P-P-P-P-P-P-P-P-P-P-22 |
| PtrPPR379 | Potri.010G098600.1 | POPTR_0010s10890.1 | Chr10 | - | 12029004 | 12030695 | 1 | exon | (12029004-12030531) | 508 | P | 9 | 102-P-36-P-P-P-P-P-P-P-P-53 |
| PtrPPR380 | Potri.010G098700.1 | POPTR_0010s10900.1 | Chr10 | + | 12033411 | 12035516 | 1 | exon | (12033436-12035277) | 613 | P | 13 | 124-P-P-P-P-P-P-P-P-P-P-P-P-P-30 |
| PtrPPR381 | Potri.010G114700.1 | POPTR_0010s12490.1 | Chr10 | - | 13299862 | 13302331 | 1 | exon | (13299932-13301557) | 541 | P | 10 | 155-P-P-P-P-P-P-P-P-P-P-37 |
| PtrPPR382 | Potri.010G136200.1 | POPTR_0010s14610.1 | Chr10 | + | 14977538 | 14980244 | 1 | exon | (14977789-14980020) | 743 | DYW | 19 | 4-L1-S1-SS-P1-L1-S1-SS-P1-L1-S1-P1-L1-S1-P2-L2-S2-E1-E2-DYW |
| PtrPPR383 | Potri.010G137200.1 | POPTR_0010s14700.1 | Chr10 | - | 15031437 | 15034167 | 2 | exon-exon | (15031437-15031891)(15032112-15034167) | 836 | P | 19 | 120-P-18-P-32-P-P-P-P-P-P-P-P-P-P-P-P-P-P-P-P-P |
| PtrPPR384 | Potri.010G141200.1 | POPTR_0010s15110.1 | Chr10 | - | 15286678 | 15287889 | 4 | exon-exon-exon-exon | (15286678.-15286892)(15286947-15287039)(15287080-15287358)(15287443-15287840) | 327 | P | 4 | 20-P-17-P-P-P-148 |
| PtrPPR385 | Potri.010G148700.1 | POPTR_0010s15860.1 | Chr10 | + | 15774072 | 15776246 | 1 | exon | (15774072-15776061) | 662 | P | 16 | 72-P-P-P-P-P-P-P-P-P-P-P-P-P-P-P-P-34 |
| PtrPPR386 | Potri.010G153900.1 | POPTR_0010s16360.1 | Chr10 | - | 16155129 | 16158092 | 2 | exon-exon | (16155269-16155625)(16155905-16157011) | 487 | P | 10 | 133-P-P-P-P-P-P-P-P-P-P-2 |
| PtrPPR387 | Potri.010G158600.1 | POPTR_0010s16610.1 | Chr10 | - | 16501097 | 16502231 | 4 | exon-exon-exon-exon | (16501097-16501239)(16501400-16501445)(16501479-16502007)(16502089-16502231) | 286 | P | 2 | 159-SS-P-57 |
| PtrPPR388 | Potri.010G159100.1 | POPTR_0010s16660.1 | Chr10 | + | 16528323 | 16532651 | 6 | exon-exon-exon-exon-exon-exon | (16528450-16529081)(16529496-16529727)(16529827-16530309)(16530800-16531006)(16531360-16531497)(16531609-16531794) | 625 | P | 6 | 185-P-40-P-88-P-48-P-P-P-53 |
| PtrPPR389 | Potri.010G161700.1 |  | Chr10 | + | 16687165 | 16689230 | 5 | exon-exon-exon-exon-exon | (16687165-16687391)(16687492-16687646)(16687780-16687944)(16688147-16688351)(16688942-16689243) | 350 | PLS | 8 | 64-L1-S1-P1-30-L1-S1-P2-L2-S2 |
| PtrPPR390 | Potri.010G161800.1 | POPTR_0010s16920.1 | Chr10 | - | 16689554 | 16692997 | 1 | exon | (16689554-16691552) | 665 | E2 | 19 | 1-L1-S1-P1-L1-S1-P1-L1-S1-P1-L1-S1-P1-L1-S1-P2-L2-S2-E1-E2-24 |
| PtrPPR391 | Potri.010G168800.1 | POPTR_0010s17590.1 | Chr10 | - | 17098655 | 17100780 | 1 | exon | (17098655-17100461) | 601 | DYW | 14 | 22-L1-S1-P1-L1-S1-P1-L1-S1-P2-L2-S2-E1-E2-DYW |
| PtrPPR392 | Potri.010G173400.1 | POPTR_0010s18060.1 | Chr10 | - | 17419881 | 17422452 | 1 | exon | (17419881-17422239) | 785 | P | 10 | 154-P-P-P-P-P-P-P-P-P-141-P-138 |
| PtrPPR393 | Potri.010G217600.1 | POPTR_0010s22460.1 | Chr10 | + | 20399710 | 20402591 | 1 | exon | (20399710-20402065) | 784 | P | 18 | 138-P-P-P-P-P-P-P-P-P-P-P-P-P-P-P-P-23-P-P |
| PtrPPR394 | Potri.010G234500.1 | POPTR_0010s24080.1 | Chr10 | + | 21516729 | 21520635 | 1 | exon | (21517030-21519315) | 761 | P | 19 | 39-P-P-P-P-P-P-P-P-P-P-P-P-P-P-P-P-P-P-P-55 |
| PtrPPR395 | Potri.010G241100.1 | POPTR_0010s24750.1 | Chr10 | + | 21823425 | 21825203 | 1 | exon | (21823445-21825076) | 543 | E+ | 14 | 48-L1-S1-P1-L1-S1-P1-L1-S1-P2-L2-S2-E1-E2-E+ |
| PtrPPR396 | Potri.010G241300.1 | POPTR_0010s24770.1 | Chr10 | + | 21828379 | 21836597 | 10 | exon-exon-exon-exon-exon-exon-exon-exon-exon-exon | (21828410-21829282)(21829665-21829792)(21830231-21830371)(21830762-21830848)(21831370-21831523)(21832510-21832569)(21833593-21834021)(21834622-21834917)(21835096-21835227)(21835695-21836240) | 947 | P | 15 | 293-P-P-P-P-P-P-P-P-P-P-P-P-P-P-P-129 |
| PtrPPR397 | Potri.010G243000.1 | POPTR_0010s24920.1 | Chr10 | + | 21913390 | 21915468 | 6 | exon-exon-exon-exon-exon-exon | (21913390-21913402)(21913450-21913804)(21913902-21914032)(21914069-21914417)(21914851-21915000)(21915152-21915468) | 457 | P | 10 | P-31-P-22-P-P-23-P-P-P-P-18-P-P-18 |
| PtrPPR398 | Potri.010G249800.1 | POPTR_0010s25620.1 | Chr10 | + | 22253385 | 22259187 | 2 | exon-exon | (22253385-22255820)(22255869-22255923) | 829 | P | 19 | 100-P-P-P-P-P-P-P-P-P-P-P-P-P-P-P-34-P-P-P-P-29 |
| PtrPPR399 | Potri.011G001100.1 | POPTR_0021s01000.1 | Chr11 | + | 74647 | 76227 | 1 | exon | (74675-75880) | 401 | P | 7 | 101-P-P-P-36-P-P-P-P-12 |
| PtrPPR400 | Potri.011G003700.1 | POPTR_0021s00720.1 | Chr11 | + | 273161 | 275512 | 1 | exon | (273161-275198) | 678 | P | 15 | 115-P-17-P-P-P-P-P-P-P-P-P-P-P-P-P-P-19 |
| PtrPPR401 | Potri.011G032400.1 | POPTR_0011s03500.1 | Chr11 | - | 2673287 | 2674555 | 1 | exon | (2673309-2674514) | 401 | PLS | 6 | 93-S1-P1-P1-P1-P1-P1-97 |
| PtrPPR402 | Potri.011G032500.1 | POPTR_0011s03510.1 | Chr11 | - | 2677924 | 2679370 | 1 | exon | (2677962-2679134) | 390 | PLS | 5 | 98-S1-P1-P1-P1-35-P1-81 |
| PtrPPR403 | Potri.011G051500.1 | POPTR_0011s05120.1 | Chr11 | - | 4418365 | 4420624 | 2 | exon-exon | (4418408-4418653)(4419155-4420393) | 494 | P | 8 | 60-P-34-P-P-P-P-P-P-P-119 |
| PtrPPR404 | Potri.011G051700.1 | POPTR_0011s05140.1 | Chr11 | + | 4434068 | 4435194 | 3 | exon-exon-exon | (4434068-4434082)(4434112-4434344)(4434495-4435194) | 315 | P | 5 | 33-P-21-P-P-P-P-86 |
| PtrPPR405 | Potri.011G052300.1 | POPTR_0011s05200.1 | Chr11 | + | 4477225 | 4479567 | 1 | exon | (4477225.-4478983) | 585 | DYW | 14 | 8-L1-S1-P1-L1-S1-P1-L1-S1-P2-L2-S2-E1-E2-DYW |
| PtrPPR406 | Potri.011G057900.1 | POPTR_0011s05680.1 | Chr11 | + | 5112324 | 5115452 | 1 | exon | (5112470-5113906) | 478 | P | 11 | 49-P-P-P-P-P-P-P-P-P-P-P-36 |
| PtrPPR407 | Potri.011G069400.1 | POPTR_0011s07060.1 | Chr11 | + | 6470681 | 6471396 | 3 | exon-exon-exon | (6470681-6470699)(6470779-6470829)(6471134-6471396) | 110 | P | 2 | 10-SS-P1-34 |
| PtrPPR408 | Potri.011G082300.1 | POPTR_0011s02450.1 | Chr11 | - | 8256557 | 8258824 | 1 | exon | (8256557-21915445) | 684 | P | 17 | 82-P-P-P-P-P-P-P-P-P-P-P-P-P-P-P-P-P-9 |
| PtrPPR409 | Potri.011G108500.1 | POPTR_0011s10980.1 | Chr11 | - | 13309905 | 13313299 | 1 | exon | (13310053-13311438) | 461 | P | 10 | 44-P-P-P-P-P-P-P-P-P-P-66 |
| PtrPPR410 | Potri.011G115500.1 | POPTR_0011s11610.1 | Chr11 | - | 14045866 | 14048806 | 1 | exon | (14045887-14047524) | 545 | P | 10 | 148-P-P-P-P-P-P-P-P-P-P-49 |
| PtrPPR411 | Potri.011G120900.1 | POPTR_0011s12150.1 | Chr11 | + | 14614235 | 14617913 | 2 | exon-exon | (14614624-14614884)(14616405-14617646) | 500 | P | 10 | 65-P-34-P-P-P-P-P-P-P-P-39-P-12 |
| PtrPPR412 | Potri.011G133100.1 | POPTR_0011s13640.1 | Chr11 | - | 15775964 | 15783839 | 11 | exon-exon-exon-exon-exon-exon-exon-exon-exon-exon-exon | (15775994-15776237)(15776331-15776393)(15776842-15777468)(15779297-15779476)(15779953-15780017)(15781023-15781130)(15781348-15781461)(15781886-15781981)(15782144-15782242)(15783080-15783199)(15783826-15783839) | 576 | P | 4 | 202-P-176-P-35-P-P-23 |
| PtrPPR413 | Potri.011G144300.1 | POPTR_0011s14750.1 | Chr11 | + | 16475604 | 16477307 | 2 | exon-exon | (16475604-16476031)(16476239-16477307) | 498 | P | 11 | 61-P-P-P-P-P-P-P-34-P-P-P-P-17 |
| PtrPPR414 | Potri.011G156800.1 | POPTR_0011s16020.1 | Chr11 | - | 17451038 | 17453282 | 1 | exon | (17451038-17453159) | 706 | E+ | 20 | 9-L1-S1-P1-L1-S1-P1-L1-S1-P1-L1-S1-P1-L1-S1-P2-L2-S2-E1-E2-E+ |
| PtrPPR415 | Potri.012G015400.1 | POPTR_0012s02240.1 | Chr12 | + | 1489445 | 1492072 | 2 | exon-exon | (1489445-1491378)(1491458-1491522) | 665 | P | 10 | 168-P-P-P-P-P-P-P-P-P-P-135 |
| PtrPPR416 | Potri.012G018800.1 | POPTR_0012s02580.1 | Chr12 | + | 1738027 | 1740912 | 1 | exon | (1738036-1740594) | 852 | E+ | 23 | 58-L1-S1-P1-L1-S1-P1-P1-L1-S1-SS-P1-L1-S1-P1-P1-L1-S1-P2-L2-S2-E1-E2-E+ |
| PtrPPR417 | Potri.012G031600.1 | POPTR_0012s01390.1 | Chr12 | - | 2801594 | 2805198 | 1 | exon | (2801594-2803232) | 545 | P | 12 | 92-P-P-P-P-P-P-P-P-P-P-P-P-30 |
| PtrPPR418 | Potri.012G041200.1 | POPTR_0012s03810.1 | Chr12 | + | 3706985 | 3709825 | 1 | exon | (3706985-3709535) | 849 | DWY | 19 | 85-P1-L1-S1-P1-L1-S1-P1-P1-L1-S1-P1-L1-S1-P2-L2-S2-E1-E2-DYW |
| PtrPPR419 | Potri.012G044200.1 | POPTR_0012s04120.1 | Chr12 | - | 3998749 | 4000129 | 5 | exon-exon-exon-exon-exon | (3998749-3999037)(3999160-3999560)(3999640-3999757)(3999804-3999872)(4000104-4000129) | 300 | PLS | 6 | 21-S1-P1-21-P1-L1-S1-L2-46 |
| PtrPPR420 | Potri.012G048800.1 | POPTR_0012s04610.1 | Chr12 | - | 4591308 | 4594364 | 2 | exon-exon | (4591414-4592922)(4593253-4594059) | 7771 | DYW | 19 | 53-SS-SS-SS-SS-SS-SS-SS-SS-SS-SS-P1-L1-S1-P2-L2-S2-E1-E2-DYW |
| PtrPPR421 | Potri.012G068400.1 | POPTR_0012s07030.1 | Chr12 | - | 9080756 | 9082277 | 1 | exon | (9080756-9082214) | 485 | P | 9 | 106-P-P-P-P-P-P-P-P-P-55 |
| PtrPPR422 | Potri.012G074200.1 |  | Chr12 | - | 9937201 | 9939542 | 5 | exon-exon-exon-exon-exon | (9937201-9937541)(9937658-9937861)(9937913-9937971)(9938994-9939164)(9939433-9939542) | 294 | P | 6 | P-P-P-14-P-P-19-P-56 |
| PtrPPR423 | Potri.012G080200.1 | POPTR_0012s08170.1 | Chr12 | - | 10653761 | 10657341 | 1 | exon | (10653811-10656258) | 815 | E+ | 21 | 73-P1-L1-S1-P1-L1-S1-P1-L1-S1-P1-L1-S1-P1-L1-S1-P2-L2-S2-E1-E2-E+ |
| PtrPPR424 | Potri.012G082900.1 | POPTR_0012s08460.1 | Chr12 | - | 10992316 | 10994085 | 2 | exon-exon | (10992316-10992470)(10992924-10994085) | 438 | E1 | 11 | 80-P1-L1-S1-SS-P1-L1-S1-P2-L2-S2-E1 |
| PtrPPR425 | Potri.012G091800.1 | POPTR_0012s09340.1 | Chr12 | + | 11887191 | 11890412 | 2 | exon-exon | (11887279-11888135)(11888769-11890080) | 722 | P | 11 | 270-P-P-P-P-P-P-P-P-P-P-P-65 |
| PtrPPR426 | Potri.012G108000.1 | POPTR_0012s10980.1 | Chr12 | + | 13126369 | 13128972 | 2 | exon-exon | (13126447-13127447)(13127664-13128606) | 647 | E+ | 17 | 59-L1-S1-P1-L1-S1-P1-L1-S1-P1-L1-S1-P2-L2-S2-E1-E2-E+ |
| PtrPPR427 | Potri.012G109300.1 | POPTR_0012s11110.1 | Chr12 | - | 13208342 | 13211297 | 2 | exon-exon | (13208453-13208567)(13208815-13210331) | 543 | DYW | 10 | 107-SS-P1-L1-S1-P2-L2-S2-E1-E2-DYW |
| PtrPPR428 | Potri.012G110800.1 | POPTR_0012s11260.1 | Chr12 | + | 13290615 | 13292344 | 1 | exon | (13290615-13292226) | 536 | E+ | 15 | 11-L1-S1-P1-L1-S1-SS-P1-L1-S1-P2-L2-S2-E1-E2-E+ |
| PtrPPR429 | Potri.012G111900.1 | POPTR_0012s11360.1 | Chr12 | + | 13342379 | 13344962 | 1 | exon | (13342464-13344614) | 716 | DYW | 17 | 36-L1-S1-P1-L1-S1-P1-L1-S1-P1-L1-S1-P2-L2-S2-E1-E2-DYW |
| PtrPPR430 | Potri.012G122800.1 | POPTR_0012s12330.1 | Chr12 | + | 14207271 | 14211651 | 8 | exon-exon-exon-exon-exon-exon-exon-exon | (14207271-14207384)(14208094-14208278)(14208366-14208598)(14208919-14209169)(14209788-14209933)(14210197-14210402)(14210536-14210660)(14211040-14211560) | 592 | P | 11 | 191-P-P-P-P-P-P-P-P-P-P-P-16 |
| PtrPPR431 | Potri.012G123600.1 | POPTR_0012s14970.1 | Chr12 | + | 14275449 | 14280063 | 7 | exon-exon-exon-exon-exon-exon-exon | (14276377-14275577)(14276365-14276549)(14276637-14276869)(14277190-14277439)(14278059-14278204)(14278468-14278673)(14278807-14278872)(14279309-14279892) | 576 | P | 12 | 154-P-P-P-P-P-P-P-P-P-P-P-P-2 |
| PtrPPR432 | Potri.012G135600.1 | POPTR_0012s13730.1 | Chr12 | - | 15174029 | 15176916 | 2 | exon-exon | (15174151-15175957)(15176102-15176109) | 604 | E+ | 15 | 73-P1-L1-S1-P1-L1-S1-P1-L1-S1-P2-L2-S2-E1-E2-E+ |
| PtrPPR433 | Potri.012G140400.1 | POPTR_0012s13270.1 | Chr12 | - | 15431976 | 15433767 | 2 | exon-exon | (15431976-15432457)(15432656-15433638) | 487 | E2 | 14 | 1-L1-S1-P1-L1-SS-SS-P1-L1-S1-P2-L2-S2-E1-E2-7 |
| PtrPPR434 | Potri.012G141400.1 | POPTR_0012s13170.1 | Chr12 | + | 15496159 | 15497596 | 6 | exon-exon-exon-exon-exon-exon | (15496159-15496308)(15496359-15496574)(15496659-15496875)(15496915-15497076)(15497126-15497355)(15497441-15497596 ) | 376 | P | 7 | 112-P-P-22-P-P-P-P-P-11 |
| PtrPPR435 | Potri.013G002800.1 | POPTR_0013s00430.1 | Chr13 | + | 194300 | 196682 | 1 | exon | (194509-196682) | 724 | P | 16 | 91-P-P-P-P-P-P-P-P-P-P-P-P-P-P-P-35-P-42 |
| PtrPPR436 | Potri.013G005400.1 | POPTR_0013s00680.1 | Chr13 | - | 350302 | 353572 | 1 | exon | (350302-352414) | 703 | DYW | 18 | 15-SS-SS-SS-SS-SS-SS-SS-SS-SS-P1-L1-S1-P2-L2-S2-E1-E2-DYW |
| PtrPPR437 | Potri.013G006700.1 | POPTR_0013s00810.1 | Chr13 | + | 431468 | 434404 | 2 | exon-exon | (431468-432089)(432270-433481) | 610 | E+ | 16 | 43-SS-P1-L1-S1-P1-L1-S1-P1-L1-S1-P2-L2-S2-E1-E2-E+ |
| PtrPPR438 | Potri.013G006800.1 | POPTR_0013s00820.1 | Chr13 | + | 439349 | 441472 | 1 | exon | (439349..441472) | 707 | E+ | 20 | 6-L1-S1-P1-L1-S1-P1-L1-S1-P1-L1-S1-P1-L1-S1-P2-L2-S2-E1-E2-E+ |
| PtrPPR439 | Potri.013G008000.1 | POPTR_0013s00940.1 | Chr13 | + | 505023 | 505981 | 5 | exon-exon-exon-exon-exon | (505023-505239)(505271-505497)(505527-505617)(505655-505820)(505897-505981) | 261 | DWY | 5 | 3-S1-P2-21-S2-E1-DYW |
| PtrPPR440 | Potri.013G021100.1 | POPTR_0013s02200.1 | Chr13 | - | 1398221 | 1400364 | 1 | exon | (1398221-1400234) | 670 | E+ | 16 | 84-S1-P1-L1-S1-P1-L1-S1-P1-L1-S1-P2-L2-S2-E1-E2-E+ |
| PtrPPR441 | Potri.013G032600.1 | POPTR_0013s03290.1 | Chr13 | - | 2162117 | 2164007 | 1 | exon | (2162515-2164007) | 497 | P | 12 | 46-P-P-P-P-P-P-P-P-P-P-P-P-31 |
| PtrPPR442 | Potri.013G034200.1 | POPTR_0030s00250.1 | Chr13 | + | 2284185 | 2286272 | 1 | exon | (2284243-2286078) | 611 | P | 14 | 88-P-P-P-P-P-P-P-P-P-P-P-P-P-P-31 |
| PtrPPR443 | Potri.013G034300.1 | POPTR_0030s00260.1 | Chr13 | + | 2288253 | 2289635 | 1 | exon | (2288253_2289635) | 460 | P | 12 | 9-P-P-P-P-P-P-P-P-P-P-P-P-31 |
| PtrPPR444 | Potri.013G034400.1 | POPTR_0030s00270.1 | Chr13 | + | 2291013 | 2293074 | 1 | exon | (2291075-2292832) | 585 | P | 14 | 68-P-P-P-P-P-P-P-P-P-P-P-P-P-P-25 |
| PtrPPR445 | Potri.013G041100.1 |  | Chr13 | + | 2875834 | 2876529 | 2 | exon-exon | (2875834-2876420)(2876521-2876529) | 217 | PLS | 7 | SS-P1-L1-S1-SS-P1-L1 |
| PtrPPR446 | Potri.013G044700.1 | POPTR_0013s04190.1 | Chr13 | - | 3168618 | 3171392 | 3 | exon-exon-exon | (3168618-3168885)(3168943-3169231)(3169475-3171036) | 705 | DYW | 15 | 108-L1-S1-P1-P1-L1-S1-P1-L1-S1-P2-L2-S2-E1-E2-DYW |
| PtrPPR447 | Potri.013G047000.1 | POPTR_0013s04410.1 | Chr13 | - | 3395701 | 3397883 | 1 | exon | (3395701-3397165) | 487 | P | 13 | 9-P-P-P-P-P-P-P-P-P-P-P-P-P-23 |
| PtrPPR448 | Potri.013G053600.1 | POPTR_0671s00210.1 | Chr13 | + | 3997943 | 4000334 | 1 | exon | (3998253-4000052) | 599 | P | 9 | 169-P-P-P-P-P-P-P-32-P-P-80 |
| PtrPPR449 | Potri.013G054400.1 | POPTR_0013s05130.1 | Chr13 | + | 4065590 | 4067195 | 2 | exon-exon | (4065590-4065639)(4065703-4067150) | 498 | E2 | 12 | 74-P1-L1-S1-SS-P1-L1-S1-P2-L2-S2-E1-E2-7 |
| PtrPPR450 | Potri.013G058900.1 | POPTR_0013s05550.1 | Chr13 | + | 4405873 | 4415960 | 1 | exon | (4405873-4408843) | 989 | DYW | 26 | 9-L1-S1-P1-L1-S1-P1-L1-S1-P1-L1-S1-P1-L1-S1-P1-L1-S1-P1-L1-S1-P2-L2-S2-E1-E2-DYW |
| PtrPPR451 | Potri.013G089400.1 | POPTR_0013s09580.1 | Chr13 | - | 9410646 | 9412429 | 1 | exon | (9410646-9412224) | 525 | E+ | 12 | 76-P1-30-S1-SS-P1-L1-S1-P2-L2-S2-E1-E2-E+ |
| PtrPPR452 | Potri.013G090600.1 | POPTR_0013s09430.1 | Chr13 | - | 9566157 | 9569091 | 2 | exon-exon | (9566218-9568831)(9568901-9568929) | 880 | E+ | 23 | 48-P1-L1-S1-P1-33-S1-P1-L1-S1-P1-L1-S1-P1-L1-S1-P1-L1-S1-P2-L2-S2-E1-E2-E+ |
| PtrPPR453 | Potri.013G098800.1 | POPTR_0013s10600.1 | Chr13 | - | 10816682 | 10821261 | 3 | exon-exon-exon | (10816682-10816767)(10818995-10819120)(10819155-10821261) | 772 | DYW | 16 | 85-L1-S1-P1-L1-S1-P1-L1-31-P1-L1-S1-P2-L2-S2-E1-E2-DYW |
| PtrPPR454 | Potri.013G103600.1 | POPTR_0013s10100.1 | Chr13 | + | 11719185 | 11725873 | 2 | exon-exon | (11719185-11719225)(11720035-11721890) | 631 | E+ | 15 | 102-P1-L1-S1-P1-L1-S1-P1-L1-S1-P2-L2-S2-E1-E2-E+ |
| PtrPPR455 | Potri.013G105000.1 | POPTR_0013s10970.1 | Chr13 | + | 11876645 | 11879331 | 2 | exon-exon | (11876645-11877428)(11877624-11878829) | 662 | P | 14 | 143-P-P-P-P-P-P-P-P-P-P-P-P-P-P-35 |
| PtrPPR456 | Potri.013G121600.1 |  | Chr13 | - | 13552825 | 13553533 | 2 | exon-exon | (13552850-13553105)(13553164-13553533) | 208 | P | 4 | 21-P-P-16-P-P-31 |
| PtrPPR457 | Potri.013G122600.1 | POPTR_0191s00220.1 | Chr13 | - | 13668379 | 13669137 | 2 | exon-exon | (13668379-13668454)(13668860-13669137) | 117 | P | 3 | 12-P-P-P |
| PtrPPR458 | Potri.013G124100.1 | POPTR_0013s12790.1 | Chr13 | + | 13772952 | 13775347 | 1 | exon | (13773000-13774025) | 341 | P | 4 | 133-P-P-P-36-P-32 |
| PtrPPR459 | Potri.013G129100.1 | POPTR_0013s13270.1 | Chr13 | - | 14116889 | 14120050 | 1 | exon | (14117085-14119889) | 937 | DWY | 24 | 5-P1-L1-S1-P1-L1-S1-P1-L1-S1-P1-L1-S1-P1-L1-S1-P1-L1-S1-P2-L2-S2-E1-E2-DYW |
| PtrPPR460 | Potri.013G129500.1 | POPTR_0013s13310.1 | Chr13 | - | 14138704 | 14141508 | 2 | exon-exon | (14138704-14140459)(14140703-14141508) | 853 | E1 | 21 | 96-L1-S1-P1-L1-S1-P1-L1-S1-P1-L1-S1-P1-L1-S1-22-P1-L1-S1-P2-L2-S2-E1-14 |
| PtrPPR461 | Potri.013G129700.1 | POPTR_0013s13330.1 | Chr13 | + | 14152049 | 14153131 | 4 | exon-exon-exon-exon | (14152049-14152351)(14152461-14152703)(14152834-14152993)(14153040-14153131) | 265 | PLS | 4 | L1-S1-P2-L2-148 |
| PtrPPR462 | Potri.013G130600.1 | POPTR_0013s13420.1 | Chr13 | - | 14212198 | 14217006 | 2 | exon-exon | (14212198-14213656)(14216024-14216083) | 505 | P | 12 | 33-P-P-P-P-P-P-P-P-P-P-P-P-52 |
| PtrPPR463 | Potri.013G132500.1 | POPTR_0013s13600.1 | Chr13 | - | 14373811 | 14377004 | 2 | exon-exon | (14373866-14374147)(14374980-14376101) | 469 | P | 8 | 74-P-34-P-P-P-P-P-P-P-78 |
| PtrPPR464 | Potri.013G132600.1 | POPTR_0013s13610.1 | Chr13 | - | 14378280 | 14379634 | 2 | exon-exon | (14378280-14378497)(14378795-14379009) | 143 | P | 3 | 1-P-P-P-28 |
| PtrPPR465 | Potri.013G132700.1 | POPTR_0013s13620.1 | Chr13 | - | 14382169 | 14384199 | 2 | exon-exon | (14382169-14382368)(14382765-14384012) | 501 | P | 7 | 133-P-P-P-P-P-P-P-122 |
| PtrPPR466 | Potri.013G133000.1 | POPTR_0013s13650.1 | Chr13 | - | 14392832 | 14395171 | 3 | exon-exon-exon | (14392967-14393266)(14393745-14394760)(14394825-14394957) | 482 | P | 7 | 147-P-P-P-P-P-P-P-88 |
| PtrPPR467 | Potri.013G144800.1 | POPTR_0013s14110.1 | Chr13 | + | 15077481 | 15080795 | 1 | exon | (15077525-15080584) | 1019 | P | 19 | 183-P-P-P-53-P-P-P-P-P-P-P-P-P-P-P-P-P-P-68-P-P-52 |
| PtrPPR468 | Potri.013G145000.1 | POPTR_0013s14130.1 | Chr13 | - | 15082007 | 15083827 | 1 | exon | (15082007-15083827) | 606 | E2 | 16 | 59-L1-S1-P1-L1-S1-P1-L1-S1-P1-L1-S1-P2-L2-S2-E1-E2-9 |
| PtrPPR469 | Potri.013G149800.1 | POPTR_0013s14620.1 | Chr13 | + | 15433815 | 15435908 | 1 | exon | (15433869-15435761) | 630 | P | 14 | 61-P-P-P-P-P-P-P-P-P-P-P-P-P-P-80 |
| PtrPPR470 | Potri.013G150100.1 | POPTR_0013s14640.1 | Chr13 | - | 15439936 | 15441771 | 1 | exon | (15440096-15441771) | 558 | P | 14 | 58-P-P-P-P-P-P-P-P-P-P-P-P-P-P-10 |
| PtrPPR471 | Potri.013G152000.1 | POPTR_0013s14820.1 | Chr13 | - | 15576931 | 15579171 | 1 | exon | (15576931-15579171) | 746 | DYW | 16 | 106-S1-P1-L1-S1-P1-L1-S1-P1-L1-S1-P2-L2-S2-E1-E2-DYW |
| PtrPPR472 | Potri.014G012500.1 | POPTR_0014s01290.1 | Chr14 | - | 1287886 | 1290048 | 2 | exon-exon | (1287886-1289773)(1289819-1290048) | 705 | DYW | 17 | 7-L1-S1-SS-P1-33-S1-P1-L1-S1-P1-L1-S1-P2-L2-S2-E1-E2-DYW |
| PtrPPR473 | Potri.014G029500.1 | POPTR_0014s02950.1 | Chr14 | + | 2501883 | 2503747 | 1 | exon | (2501907-2503712) | 601 | E2 | 16 | 5-P1-L1-S1-P1-29-S1-P1-L1-S1-P1-L1-S1-P2-L2-S2-E1-E2-27 |
| PtrPPR474 | Potri.014G040200.1 | POPTR_0014s03970.1 | Chr14 | + | 3274750 | 3280667 | 1 | exon | (3274933-3277779) | 948 | P | 22 | 94-P-19-P-P-P-P-P-P-P-P-P-P-P-P-P-P-P-35-P-P-P-P-P-P-29 |
| PtrPPR475 | Potri.014G046200.1 | POPTR_0014s04560.1 | Chr14 | - | 3629563 | 3631350 | 1 | exon | (3629563-3631350) | 595 | E+ | 17 | 12-L1-S1-P1-L1-S1-SS-SS-SS-P1-L1-S1-P2-L2-S2-E1-E2-E+ |
| PtrPPR476 | Potri.014G050300.1 | POPTR_0014s04920.1 | Chr14 | + | 4041392 | 4044219 | 2 | exon-exon | (4041549-4041914)(4042523-4043776) | 539 | P | 9 | 100-P-35-P-P-P-P-P-P-P-P-88 |
| PtrPPR477 | Potri.014G052800.1 | POPTR_0014s05170.1 | Chr14 | - | 4173864 | 4178390 | 1 | exon | (4173952-4176624) | 890 | P | 18 | 195-P-P-P-P-P-P-P-P-P-P-P-P-P-P-P-P-P-P-66 |
| PtrPPR478 | Potri.014G056400.1 | POPTR_0014s05550.1 | Chr14 | - | 4379278 | 4382386 | 2 | exon-exon | (4379349-4379449)(4379543-4381628) | 728 | P | 14 | 122-P-P-P-P-P-P-P-P-P-P-P-P-67-P-P-36 |
| PtrPPR479 | Potri.014G068800.1 | POPTR_0014s06440.1 | Chr14 | - | 5584990 | 5587283 | 2 | exon-exon | (5584990-5586817)(5587258-5587283) | 617 | E2 | 15 | 91-P1-L1-S1-SS-SS-SS-SS-P1-L1-S1-P2-L2-S2-E1-E2-25 |
| PtrPPR480 | Potri.014G070500.1 | POPTR_0014s06610.1 | Chr14 | - | 5717037 | 5720826 | 2 | exon-exon | (5717228-5717686)(5719851-5720615) | 407 | P | 6 | 167-P-P-P-P-P-P-30 |
| PtrPPR481 | Potri.014G087100.1 | POPTR_0014s08300.1 | Chr14 | + | 6871016 | 6874474 | 1 | exon | (6871016-6872675) | 552 | E2 | 16 | L1-S1-P1-L1-S1-P1-L1-S1-P1-L1-S1-P2-L2-S2-E1-E2-20 |
| PtrPPR482 | Potri.014G090400.1 | POPTR_0014s08611.1 | Chr14 | - | 7101707 | 7104034 | 1 | exon | (7101707_7104034) | 775 | P | 14 | 189-P-45-P-P-P-P-P-P-P-P-P-P-P-P-P-41 |
| PtrPPR483 | Potri.014G097000.1 | POPTR_0014s09270.1 | Chr14 | + | 7579003 | 7582116 | 1 | exon | (7579003-7581796) | 930 | DYW | 23 | 48-L1-S1-P1-L1-S1-P1-L1-S1-P1-P1-L1-S1-P1-P1-P1-L1-S1-P2-L2-S2-E1-E2-DYW |
| PtrPPR484 | Potri.014G105900.1 | POPTR_0014s10150.1 | Chr14 | - | 8339169 | 8342053 | 1 | exon | (8339324-8340751) | 475 | P | 9 | 99-P-P-P-P-P-P-P-P-P-60 |
| PtrPPR485 | Potri.014G116600.1 | POPTR_0014s11190.1 | Chr14 | + | 9065816 | 9070717 | 1 | exon | (9065906-9067954) | 682 | E1 | 6 | 319-L1-S1-P2-L2-S2-E1-160 |
| PtrPPR486 | Potri.014G117600.1 | POPTR_0014s11290.1 | Chr14 | - | 9144410 | 9146434 | 1 | exon | (9144410-9145835) | 474 | P | 10 | 96-P-P-P-P-P-P-P-P-P-P-28 |
| PtrPPR487 | Potri.014G118500.1 | POPTR_0014s11380.1 | Chr14 | - | 9218678 | 9222807 | 4 | exon-exon-exon-exon | (9218897-9220857)(9221314-9221530)(9221679-9221863)(9221955-9222219) | 875 | P | 17 | 194-SS-P1-P1-P1-P1-P1-P1-P1-P1-P1-P1-27-P1-P1-198 |
| PtrPPR488 | Potri.014G119000.1 | POPTR_0014s11420.1 | Chr14 | - | 9234045 | 9236246 | 2 | exon-exon | (9234045-9234306)(9234825-9236084) | 506 | P | 8 | 65-P-30-P-P-P-P-P-P-P-119 |
| PtrPPR489 | Potri.014G121400.1 | POPTR_0014s11650.1 | Chr14 | + | 9380564 | 9383534 | 2 | exon-exon | (9380564-9380723)(9381198-9383183) | 714 | P | 11 | 289-P-P-P-P-P-P-P-P-P-P-P-42 |
| PtrPPR490 | Potri.014G132100.1 | POPTR_0014s12660.1 | Chr14 | + | 10048448 | 10050593 | 1 | exon | (10048448-10049810) | 453 | E1 | 10 | 117-P1-L1-S1-P1-L1-S1-P2-L2-S2-E1 |
| PtrPPR491 | Potri.014G139800.1 | POPTR_0014s13590.1 | Chr14 | + | 10664295 | 10665435 | 3 | exon-exon-exon | (10664295-10664345)(10664410-10664630)(10665186-10665435) | 174 | P | 3 | 8-L1-S1-P1-65 |
| PtrPPR492 | Potri.014G149500.1 | POPTR_0014s14700.1 | Chr14 | - | 11444427 | 11446519 | 2 | exon-exon | (11444501-11444887)(11444929-11446068) | 508 | P | 6 | 143-P-P-P-P-P-69-P-86 |
| PtrPPR493 | Potri.014G166000.1 | POPTR_0014s16390.1 | Chr14 | + | 13173782 | 13177283 | 2 | exon-exon | (13173853-13174237)(13174631-13175400) | 384 | P | 7 | 119-P-P-P-P-P-P-P-20 |
| PtrPPR494 | Potri.014G191200.1 | POPTR_0014s18910.1 | Chr14 | + | 17527519 | 17530060 | 1 | exon | (17527594-17529483) | 629 | DYW | 15 | 11-L1-S1-P1-L1-S1-SS-P1-L1-S1-P2-L2-S2-E1-E2-DYW |
| PtrPPR495 | Potri.015G018700.1 |  | Chr15 | + | 1329438 | 1333563 | 3 | exon-exon-exon | (1329438-1329681)(1330200-1330203)(1331062-1333430) | 871 | DWY | 20 | 87-L1-S1-P1-L1-S1-P1-L1-S1-P1-L1-S1-P1-L1-S1-P2-L2-S2-E1-E2-DYW |
| PtrPPR496 | Potri.015G034900.1 | POPTR_0015s04990.1 | Chr15 | - | 3000818 | 3003267 | 2 | exon-exon | (3000818-3002250)(3003048-3003267) | 550 | E2 | 13 | 32-L1-S1-P1-L1-S1-P1-L1-S1-P2-L2-S2-E1-E2-76 |
| PtrPPR497 | Potri.015G036400.1 | POPTR_0015s04820.1 | Chr15 | + | 3205720 | 3209638 | 1 | exon | (3206173-3208752) | 859 | P | 19 | 118-P-18-P-35-P-P-P-P-P-P-P-P-P-P-P-P-P-P-P-P-P-22 |
| PtrPPR498 | Potri.015G039300.1 | POPTR_0015s04490.1 | Chr15 | + | 3603942 | 3606354 | 2 | exon-exon | (3603942-3604088)(3604212-3605949) | 627 | P | 15 | 43-P-37-P-P-P-P-P-P-P-P-P-P-P-P-P-P-16 |
| PtrPPR499 | Potri.015G047600.1 | POPTR_0015s03610.1 | Chr15 | + | 4849535 | 4852567 | 1 | exon | (4849535-4852517) | 993 | DYW | 23 | 111-L1-S1-P1-L1-S1-P1-L1-S1-P1-L1-S1-P1-L1-S1-P1-L1-S1-P2-L2-S2-E1-E2-DYW |
| PtrPPR500 | Potri.015G066400.1 | POPTR_0015s07760.1 | Chr15 | + | 9117747 | 9122361 | 1 | exon | (9119272-9121986) | 904 | P | 10 | 407-P-P-P-P-P-P-P-P-P-35-P-112 |
| PtrPPR501 | Potri.015G069100.1 | POPTR_0015s08030.1 | Chr15 | - | 9365160 | 9370108 | 4 | exon-exon-exon-exon | (9365312-9367152)(9367847-9368161)(9368242-9368349)(9368886-9369222) | 866 | P | 16 | 106-P-P-P-P-P-P-P-P-P-P-P-P-P-P-P-36-P-162 |
| PtrPPR502 | Potri.015G070200.1 | POPTR_0015s08150.1 | Chr15 | + | 9470370 | 9471902 | 1 | exon | (9470370-9471846) | 491 | P | 10 | 135-P-P-P-P-P-P-P-P-P-P-4 |
| PtrPPR503 | Potri.015G082400.1 | POPTR_0015s09390.1 | Chr15 | - | 10664176 | 10668061 | 2 | exon-exon | (10664176-10665097)(10666150-10667793) | 854 | E2 | 16 | 303-L1-S1-P1-L1-S1-P1-L1-S1-P1-L1-S1-P2-L2-S2-E1-E2-10 |
| PtrPPR504 | Potri.015G084800.1 | POPTR_0015s09640.1 | Chr15 | + | 10864880 | 10866933 | 1 | exon | (10864900-10866798) | 632 | P | 8 | 267-P-P-37-P-P-P-P-P-31-P-16 |
| PtrPPR505 | Potri.015G095100.1 | POPTR_0015s10720.1 | Chr15 | - | 11611643 | 11613757 | 1 | exon | 11611643-11613757 | 704 | DYW | 14 | 130-L1-S1-P1-L1-S1-P1-L1-S1-P2-L2-S2-E1-E2-DYW |
| PtrPPR506 | Potri.015G105400.1 | POPTR_0015s11670.1 | Chr15 | + | 12348770 | 12353505 | 1 | exon | (12348851-12351976) | 1041 | P | 25 | 100-P-30-P-P-P-P-P-P-P-P-P-P-P-P-P-P-P-P-P-P-P-P-P-P-P-P-37 |
| PtrPPR507 | Potri.015G109000.1 | POPTR_0015s12050.1 | Chr15 | + | 12592676 | 12593301 | 3 | exon-exon-exon | (12592676-12592769)(12592877-12592946)(12592983-12593301) | 160 | PLS | 4 | 9-SS-SS-P1-23-L1-6 |
| PtrPPR508 | Potri.015G121700.1 | POPTR_0015s15360.1 | Chr15 | - | 13588790 | 13590748 | 1 | exon | (13588965-13590748) | 594 | P | 15 | 39-P-P-P-P-P-P-P-P-P-P-P-P-P-P-P-32 |
| PtrPPR509 | Potri.015G128800.1 | POPTR_0015s14630.1 | Chr15 | - | 14047310 | 14049413 | 1 | exon | (14047418-14049007) | 529 | E2 | 12 | 95-L1-S1-P1-L1-S1-P1-32-S1-SS-L2-S2-E1-E2 |
| PtrPPR510 | Potri.015G138000.1 | POPTR_0015s13680.1 | Chr15 | - | 14679222 | 14682261 | 3 | exon-exon-exon | (14679222-14679424)(14679517-14679772)(14679868-14681005) | 531 | E+ | 12 | 75-L1-S1-34-S1-P1-L1-S1-P2-L2-S2-E1-E2-E+ |
| PtrPPR511 | Potri.015G144100.1 | POPTR_0015s13120.1 | Chr15 | + | 14996205 | 14998371 | 1 | exon | (14996599-14998041) | 480 | P | 9 | 155-P-P-P-P-P-P-P-P-P-10 |
| PtrPPR512 | Potri.016G005700.1 | POPTR_0016s00750.1 | Chr16 | + | 280133 | 280567 | 1 | exon | (280133-280567) | 144 | P | 4 | 10-P-P-P-P |
| PtrPPR513 | Potri.016G005800.1 |  | Chr16 | + | 281535 | 281975 | 2 | exon-exon | (281535-281605)(281744-281975) | 100 | P | 3 | 9-P-P-P |
| PtrPPR514 | Potri.016G006800.1 | POPTR_0016s00860.1 | Chr16 | + | 334383 | 336797 | 1 | exon | (334423-336525) | 700 | P | 10 | 175-P-P-P-P-P-P-P-P-P-66-P-109 |
| PtrPPR515 | Potri.016G010100.1 | POPTR_0016s01140.1 | Chr16 | - | 506075 | 508028 | 2 | exon-exon | (506075-506413)(506751-508028) | 538 | E+ | 11 | 148-L1-S1-P1-L1-S1-P2-L2-S2-E1-E2-E+ |
| PtrPPR516 | Potri.016G015900.1 |  | Chr16 | + | 864852 | 865481 | 3 | exon-exon-exon | (864852-865041)(865105-865315)(865356-865396) | 164 | PLS | 2 | 41-S1-L1-25 |
| PtrPPR517 | Potri.016G017800.1 | POPTR_0016s01930.1 | Chr16 | + | 952946 | 954381 | 2 | exon-exon | (952946-953345)(953886-954281) | 264 | P | 7 | 6-P-P-P-P-17-P-P-P |
| PtrPPR518 | Potri.016G019900.1 | POPTR_0016s02110.1 | Chr16 | + | 1085400 | 1087940 | 1 | exon | (1085479-1087710) | 742 | DYW | 22 | 15-SS-SS-SS-SS-SS-SS-SS-SS-SS-SS-SS-SS-SS-P1-L1-S1-P2-L2-S2-E1-E2-DYW |
| PtrPPR519 | Potri.016G025600.1 | POPTR_0016s02630.1 | Chr16 | + | 1443070 | 1445182 | 2 | exon-exon | (1443609-1444828)(1444904-1445182) | 499 | P | 13 | P-P-P-P-P-P-P-P-P-P-P-P-P-35 |
| PtrPPR520 | Potri.016G026400.1 |  | Chr16 | + | 1492900 | 1496651 | 3 | exon-exon-exon | (1492900-1493473)(1493644-1494290)(1496169-1496225) | 405 | P | 9 | 9-P-P-P-P-P-30-P-P-P-P-51 |
| PtrPPR521 | Potri.016G026700.1 |  | Chr16 | + | 1508876 | 1509379 | 1 | exon | (1508876-1509379) | 167 | P | 4 | 44-P-P-P-P |
| PtrPPR522 | Potri.016G029500.1 | POPTR_0016s02930.1 | Chr16 | - | 1661304 | 1663058 | 1 | exon | (1661304-1663058) | 584 | DYW | 14 | 5-L1-S1-P1-L1-S1-P1-L1-S1-P2-L2-S2-E1-E2-DYW |
| PtrPPR523 | Potri.016G036100.1 | POPTR_0016s03540.1 | Chr16 | + | 2130779 | 2134634 | 3 | exon-exon-exon | (2130966-2131000)(2131818-2132391)(2132948-2134195) | 618 | P | 8 | 250-P-P-P-P-32-P-P-P-P-56 |
| PtrPPR524 | Potri.016G038400.1 | POPTR_0016s03790.1 | Chr16 | + | 2362341 | 2364020 | 1 | exon | (2362341-2364020) | 559 | E+ | 16 | 5-SS-SS-SS-SS-SS-SS-SS-P1-L1-S1-P2-L2-S2-E1-E2-E+ |
| PtrPPR525 | Potri.016G040700.1 |  | Chr16 | - | 2523335 | 2524293 | 2 | exon-exon | (2523332-2523848)(2524124-2524293) | 228 | PLS | 5 | 45-S1-P1-L1-16-P2-L2 |
| PtrPPR526 | Potri.016G051300.1 | POPTR_0016s05180.1 | Chr16 | + | 3276583 | 3279811 | 2 | exon-exon | (3276745-3277060)(3277699-3279143) | 586 | DYW | 14 | 9-L1-S1-P1-L1-S1-P1-L1-S1-P2-L2-S2-E1-E2-DYW |
| PtrPPR527 | Potri.016G053500.1 | POPTR_0016s05390.1 | Chr16 | + | 3461733 | 3464361 | 1 | exon | (3461874-3463757) | 627 | DYW | 14 | 53-L1-S1-P1-L1-S1-P1-L1-S1-P2-L2-S2-E1-E2-DYW |
| PtrPPR528 | Potri.016G063300.1 | POPTR_0016s06320.1 | Chr16 | + | 4453533 | 4457116 | 2 | exon-exon | (4453692-4454027)(4455125-4456306) | 505 | P | 6 | 159-P-P-36-P-P-P-P-99 |
| PtrPPR529 | Potri.016G063400.1 | POPTR_0016s06330.1 | Chr16 | + | 4459329 | 4462607 | 2 | exon-exon | (4459340-4459645)(4461062-4462315) | 519 | P | 7 | 149-P-P-36-P-33-P-P-P-P-53 |
| PtrPPR530 | Potri.016G065500.1 | POPTR_0016s06560.1 | Chr16 | + | 4618268 | 4620531 | 1 | exon | (4618489-4620342) | 617 | DYW | 15 | 11-L1-S1-P1-L1-S1-SS-P1-L1-S1-P2-L2-S2-E1-E2-DYW |
| PtrPPR531 | Potri.016G075000.1 | POPTR_0016s07590.1 | Chr16 | + | 5680329 | 5682844 | 1 | exon | (5680329-5682342) | 670 | DYW | 15 | 63-L1-S1-SS-P1-L1-S1-P1-L1-S1-P2-L2-S2-E1-E2-DYW |
| PtrPPR532 | Potri.016G077700.1 | POPTR_0016s07840.1 | Chr16 | - | 5894481 | 5896355 | 1 | exon | (5894481-5896355) | 624 | DYW | 14 | 49-L1-S1-P1-L1-S1-P1-L1-S1-P2-L2-S2-E1-E2-DYW |
| PtrPPR533 | Potri.016G082200.1 | POPTR_0016s08300.1 | Chr16 | - | 6366461 | 6368405 | 2 | exon-exon | (6366461-6368006)(6368158-6368405) | 597 | DYW | 11 | 127-L1-S1-P1-L1-S1-P2-L2-S2-E1-E2-DYW |
| PtrPPR534 | Potri.016G085300.1 | POPTR_0016s08650.1 | Chr16 | + | 6716799 | 6719449 | 1 | exon | (6716913-6719168) | 751 | P | 12 | 143-P-48-P-P-P-55-P-P-P-P-P-P-P-17-P-63 |
| PtrPPR535 | Potri.016G086100.1 | POPTR_0016s08740.1 | Chr16 | + | 6794083 | 6797777 | 1 | exon | (6794502-6796034) | 510 | P | 9 | 114-P-P-P-P-P-P-P-P-23-P-63 |
| PtrPPR536 | Potri.016G096400.1 | POPTR_0016s11000.1 | Chr16 | - | 9244062 | 9246960 | 1 | exon | (9244062-9246810) | 915 | DYW | 21 | 103-L1-S1-P1-L1-S1-P1-L1-S1-SS-P1-L1-S1-P1-L1-S1-P2-L2-S2-E1-E2-DYW |
| PtrPPR537 | Potri.016G103000.1 | POPTR_0016s10300.1 | Chr16 | - | 10129446 | 10137666 | 7 | exon-exon-exon-exon-exon-exon-exon | (10129984-10130103)(10130244-10130378)(10130454-10130552)(10130749-10130892)(10134798-10135010)(10135938-10136139)(10137421-10137512) | 334 | P | 3 | 144-P-P-P-84 |
| PtrPPR538 | Potri.016G111600.1 | POPTR_0016s11910.1 | Chr16 | + | 11449239) | 11451981 | 1 | exon | 11449299-11451383 | 694 | P | 17 | 77-P-P-P-P-P-P-P-P-P-P-P-P-P-P-P-P-P-21 |
| PtrPPR539 | Potri.016G119600.1 | POPTR_0016s12710.1 | Chr16 | + | 12446630 | 12450073 | 2 | exon-exon | (12446713-12447237)(12447602-12448726) | 549 | P | 8 | 191-P-P-P-P-P-P-72-P-P-6 |
| PtrPPR540 | Potri.016G128900.1 | POPTR_0016s13660.1 | Chr16 | - | 13152175 | 13156076 | 1 | exon | (13152175-13154224) | 682 | DYW | 17 | 6-L1-S1-P1-L1-S1-P1-L1-S1-P1-L1-S1-P2-L2-S2-E1-E2-DYW |
| PtrPPR541 | Potri.016G130200.1 | POPTR_0016s13790.1 | Chr16 | - | 13280151 | 13280584 | 2 | exon-exon | (13280151-13280258)(13280300-13280585) | 130 | P | 3 | 13-P-P-P-1 |
| PtrPPR542 | Potri.016G136700.1 | POPTR_0016s14370.1 | Chr16 | - | 13971860 | 13973518 | 1 | exon | (13971860-13973471) | 536 | P | 11 | 141-P-P-P-P-P-P-P-P-P-P-P-5 |
| PtrPPR543 | Potri.017G001000.1 | POPTR_0017s00440.1 | Chr17 | - | 57150 | 60481 | 4 | exon-exon-exon-exon | (57286-57693)(58912-60054)(60226-60283)(60463-60481) | 542 | E+ | 14 | 5-P1-L1-S1-P1-21-S1-P1-L1-S1-P2-L2-S2-E1-E2-E+ |
| PtrPPR544 | Potri.017G032100.1 | POPTR_0017s06060.1 | Chr17 | + | 2783857 | 2785624 | 1 | exon | (2783857-2785492) | 544 | P | 12 | 116-P-P-P-P-P-P-P-P-P-P-P-P-8 |
| PtrPPR545 | Potri.017G033000.1 | POPTR_0017s06150.1 | Chr17 | + | 2845207 | 2846969 | 1 | exon | (2845234-2846969) | 578 | P | 9 | 167-P-P-P-65-P-P-P-P-24-P-P-6 |
| PtrPPR546 | Potri.017G036400.1 | POPTR_0017s06420.1 | Chr17 | + | 3060184 | 3065260 | 4 | exon-exon-exon-exon | (3060184-3063786)(3063898-3064098)(3064243-3064333)(3064759-3065250) | 1461 | P | 25 | 255-P-P-P-P-P-P-P-P-P-P-31-P-P-P-P-P-P-P-P-P-P-P-P-P-P-P-297 |
| PtrPPR547 | Potri.017G042100.1 | POPTR_0017s06960.1 | Chr17 | - | 3566310 | 3568775 | 1 | exon | (3566310-3568622) | 770 | PLS | 17 | 65-L1-S1-P1-L1-S1-P1-L1-S1-30-L1-S1-P1-L1-S1-P1-L1-S1-35-P1-69 |
| PtrPPR548 | Potri.017G048500.1 | POPTR_0017s07580.1 | Chr17 | - | 4051659 | 4055239 | 4 | exon-exon-exon-exon | (4051698-4051732)(4051878-4052166)(4053489-4054390)(4054810-4055008) | 474 | P | 6 | 86-P-34-P-P-P-74-P-P-69 |
| PtrPPR549 | Potri.017G057000.1 | POPTR_0017s08385.1 | Chr17 | + | 5135977 | 5140627 | 4 | exon-exon-exon-exon | (5135977-5136090)(5137096-5137311)(5140190-5140397)(5140443-5140627) | 240 | E+ | 6 | 9-L1-L1-S1-S2-E1-22-E+ |
| PtrPPR550 | Potri.017G060500.1 | POPTR_0017s08750.1 | Chr17 | + | 5592099 | 5592620 | 3 | exon-exon-exon | (5592099-5592292)(5592327-5592376)(5592562-5592620) | 100 | P | 3 | 1-S1-P1-23-S1 |
| PtrPPR551 | Potri.017G060600.1 |  | Chr17 | + | 5598106 | 5598298 | 1 | exon | (5598106-5598298) | 63 | E2 | 2 | 6-E1-E2 |
| PtrPPR552 | Potri.017G070800.1 | POPTR_0017s10700.1 | Chr17 | - | 8109615 | 8112484 | 1 | exon | (8109947-8112226) | 759 | P | 11 | 202-P-P-P-P-92-P-P-P-P-P-P-P-68 |
| PtrPPR553 | Potri.017G071600.1 | POPTR_0017s10790.1 | Chr17 | - | 8255895 | 8257992 | 1 | exon | (8255895-8111496) | 626 | P | 13 | 129-P-17-P-P-P-P-P-P-P-P-P-P-P-P-25 |
| PtrPPR554 | Potri.017G075700.1 | POPTR_0017s11210.1 | Chr17 | - | 8866595 | 8868620 | 2 | exon-exon | (8866601-8866752)(8866846-8868613) | 639 | P | 14 | 116-P-P-P-P-P-P-P-P-P-P-P-P-P-35-P-2 |
| PtrPPR555 | Potri.017G083900.1 | POPTR_0017s12020.1 | Chr17 | - | 10109768 | 10111295 | 1 | exon | (10109779-10111295) | 505 | E2 | 11 | 83-P1-L1-S1-P1-L1-S1-P2-L2-S2-E1-E2-45 |
| PtrPPR556 | Potri.017G084900.1 | POPTR_0017s12110.1 | Chr17 | - | 10215194 | 10218795 | 1 | exon | (10215194-10217318) | 707 | DYW | 17 | 28-L1-S1-P1-L1-S1-P1-L1-S1-P1-L1-S1-P2-L2-S2-E1-E2-DYW |
| PtrPPR557 | Potri.017G086000.1 | POPTR_0017s12190.1 | Chr17 | + | 10324302 | 10328495 | 1 | exon | (10324302-10328031) | 1242 | P | 23 | 153-P-P-38-P-66-P-P-P-65-P-36-P-P-P-35-P-P-P-P-P-P-P-P-P-P-P-P-P-47 |
| PtrPPR558 | Potri.017G086100.1 | POPTR_0017s12200.1 | Chr17 | - | 10330119 | 10331759 | 1 | exon | (10330119-10331759) | 546 | E2 | 15 | 9-L1-S1-P1-L1-S1-SS-SS-P1-L1-S1-P2-L2-S2-E1-E2-32 |
| PtrPPR559 | Potri.017G087000.1 | POPTR_0017s12280.1 | Chr17 | + | 10447088 | 10449066 | 1 | exon | (10447088-10448417) | 442 | E1 | 13 | L1-S1-P1-L1-S1-SS-P1-L1-S1-P2-L2-S2-E1-5 |
| PtrPPR560 | Potri.017G087600.1 | POPTR_0017s12350.1 | Chr17 | - | 10502016 | 10503990 | 1 | exon | (10502081-10503990) | 636 | DYW | 15 | 14-L1-S1-P1-L1-S1-SS-P1-L1-S1-P2-L2-S2-E1-E2-DYW |
| PtrPPR561 | Potri.017G090500.1 | POPTR_0017s12620.1 | Chr17 | + | 10784419 | 10787039 | 2 | exon-exon | (10784492-10786243)(10786561-10786752) | 647 | P | 12 | 206-P-P-P-P-P-P-P-P-P-P-P-P-23 |
| PtrPPR562 | Potri.017G090900.1 | POPTR_0017s12660.1 | Chr17 | - | 10831559 | 10831726 | 1 | exon | 10831559-10831726 | 55 | PLS | 2 | 4-L2-S2 |
| PtrPPR563 | Potri.017G091600.1 | POPTR_0017s12720.1 | Chr17 | - | 10858408 | 10860973 | 1 | exon | (10858437-10860524) | 695 | DYW | 15 | 88-L1-S1-SS-P1-L1-S1-P1-L1-S1-P2-L2-S2-E1-E2-DYW |
| PtrPPR564 | Potri.017G108100.1 | POPTR_0017s14350.1 | Chr17 | - | 12473082 | 12475492 | 1 | exon | (12473132-12475108) | 658 | P | 9 | 149-P-102-P-P-37-P-P-P-P-P-31-P-22 |
| PtrPPR565 | Potri.017G108600.1 | POPTR_0017s14300.1 | Chr17 | - | 12496280 | 12500525 | 1 | exon | (12496379-12498160) | 593 | P | 12 | 135-P-P-P-P-P-P-P-P-P-P-P-P-40 |
| PtrPPR566 | Potri.017G126600.1 | POPTR_0005s14380.1 | Chr17 | - | 13855258 | 13857245 | 1 | exon | (13855258-13856839) | 526 | P | 9 | 140-P-P-P-P-P-36-P-P-P-P-33 |
| PtrPPR567 | Potri.017G131400.1 | POPTR_0017s02740.1 | Chr17 | + | 14206461 | 14208342 | 1 | exon | (14206461-14208261) | 599 | P | 13 | 109-P-17-P-P-P-P-P-P-P-P-P-P-P-P-18 |
| PtrPPR568 | Potri.017G131600.1 | POPTR_0017s02730.1 | Chr17 | + | 14210427 | 14212372 | 1 | exon | (14210427-14212122) | 564 | P | 13 | 92-P-P-P-P-P-P-P-P-P-P-P-P-P-18 |
| PtrPPR569 | Potri.017G133400.1 | POPTR_0017s02600.1 | Chr17 | + | 14306798 | 14309740 | 1 | exon | (14306933-14308483) | 516 | P | 8 | 139-P-P-35-P-P-P-P-P-P-56 |
| PtrPPR570 | Potri.017G137200.1 | POPTR_0017s02260.1 | Chr17 | - | 14573380 | 14575344 | 1 | exon | (14573380-14575327) | 648 | DYW | 14 | 65-L1-S1-P1-L1-S1-P1-L1-S1-P2-L2-S2-E1-E2-DYW |
| PtrPPR571 | Potri.017G141200.1 | POPTR_0017s01850.1 | Chr17 | + | 14869521 | 14871380 | 1 | exon | (14869538-14871160) | 540 | E+ | 13 | 76-P1-L1-S1-SS-P1-L1-S1-P2-L2-S2-E1-E2-E+ |
| PtrPPR572 | Potri.017G141500.1 | POPTR_0017s01825.1 | Chr17 | - | 14893184 | 14893517 | 2 | exon-exon | (14893184-14893336)(14893381-14893517) | 96 | E2 | 2 | 10-S2-E2-6 |
| PtrPPR573 | Potri.017G144900.1 | POPTR_0017s01370.1 | Chr17 | + | 15274370 | 15278666 | 6 | exon-exon-exon-exon-exon-exon | (15274646-15275821)(15276195-15276284)(15276384-15276565)(15277490-15277610)(15278096-15278164)(15278265-15278363) | 578 | P | 4 | 106-P-P-P-P-326 |
| PtrPPR574 | Potri.017G148700.1 | POPTR_0017s00990.1 | Chr17 | + | 15479010 | 15482186 | 5 | exon-exon-exon-exon-exon | (15479397-15479483)(15479585-15479683)(15479894-15480037)(15480152-15480364)(15481167-15481691) | 355 | P | 2 | 123-P-P-161 |
| PtrPPR575 | Potri.017G153700.1 | POPTR_0017s03440.1 | Chr17 | - | 15978693 | 15980299 | 1 | exon | (15978708-15980299) | 530 | E+ | 15 | L1-S1-P1-L1-S1-SS-P1-L1-S1-P2-L2-S2-E1-E2-E+ |
| PtrPPR576 | Potri.018G003300.1 | POPTR_0018s03960.1 | Chr18 | - | 224969 | 226393 | 1 | exon | (224969-226393) | 474 | E2 | 12 | 52-P1-L1-S1-SS-P1-L1-S1-P2-L2-S2-E1-E2-10 |
| PtrPPR577 | Potri.018G004400.2 | POPTR_0018s04350.1 | Chr18 | - | 324830 | 329224 | 4 | exon-exon-exon-exon | (324988-324993)(325078-325235)(325318-325464)(326187-327657) | 593 | P | 13 | 122-P-P-P-P-P-P-P-P-P-P-P-P-P-14 |
| PtrPPR578 | Potri.018G029000.1 |  | Chr18 | - | 2288179 | 2289172 | 4 | exon-exon-exon-exon | (2288204-2288388)(2288448-2288556)(2288636-2288782)(2288937-2289172 ) | 225 | PLS | 5 | 11-L1-P1-P1-P1-L1-20 |
| PtrPPR579 | Potri.018G030400.1 | POPTR_0018s01450.1 | Chr18 | + | 2393717 | 2394167 | 3 | exon-exon-exon | (2393717-2393801)(2393832-2393948)(2394028-2394167) | 113 | P | 3 | 9-P-P-P |
| PtrPPR580 | Potri.018G035300.1 | POPTR_0018s00990.1 | Chr18 | + | 2850310 | 2852036 | 4 | exon-exon-exon-exon | (2850332-2850539)(2851101-2851373)(2851633-2851713)(2851769-2852036) | 276 | PLS | 5 | P1-L1-25-S1-P2-L2-94 |
| PtrPPR581 | Potri.018G035900.1 | POPTR_0018s00920.1 | Chr18 | - | 2907601 | 2909440 | 1 | exon | (2907700-2909343) | 547 | E+ | 15 | 10-L1-S1-P1-L1-S1-SS-P1-L1-S1-P2-L2-S2-E1-E2-E+ |
| PtrPPR582 | Potri.018G036000.1 | POPTR_0018s00915.1 | Chr18 | + | 2909505 | 2910590 | 4 | exon-exon-exon-exon | (2909505-2909555)(2909604-2910011)(2910117-2910246)(2910358-2910591) | 273 | PLS | 7 | 22-P1-L1-S1-SS-SS-22-SS-P2 |
| PtrPPR583 | Potri.018G037200.1 | POPTR_0018s00800.1 | Chr18 | + | 3075578 | 3079845 | 3 | exon-exon-exon | (3076451-3076552)(3077178-3078089)(3078479-3079378) | 637 | P | 12 | 70-P-P-P-P-P-P-P-P-P-P-P-P-146 |
| PtrPPR584 | Potri.018G038300.1 | POPTR_0018s00690.1 | Chr18 | + | 3179228 | 3181467 | 1 | exon | (3179696-3181467) | 590 | P | 13 | 115-P-P-P-P-P-P-P-P-P-P-P-P-P-20 |
| PtrPPR585 | Potri.018G040100.1 | POPTR_0018s00520.1 | Chr18 | - | 3319994 | 3322210 | 1 | exon | (3319994-3322210) | 738 | DYW | 18 | 26-L1-S1-P1-L1-S1-SS-P1-L1-S1-P1-L1-S1-P2-L2-S2-E1-E2-DYW |
| PtrPPR586 | Potri.018G067500.1 | POPTR_0018s06910.1 | Chr18 | - | 8589525 | 8593631 | 1 | exon | (8589525-8592513) | 995 | DYW | 23 | 117-L1-S1-P1-L1-S1-P1-L1-S1-P1-L1-S1-P1-L1-S1-P1-L1-S1-P2-L2-S2-E1-E2-DYW |
| PtrPPR587 | Potri.018G071800.1 | POPTR_0018s06450.1 | Chr18 | - | 9217843 | 9220099 | 1 | exon | (9217885-9220038) | 717 | DYW | 16 | 41-L1-S1-P1-32-S1-P1-L1-S1-P1-L1-S1-P2-L2-S2-E1-E2-DYW |
| PtrPPR588 | Potri.018G073700.1 |  | Chr18 | - | 9667717 | 9669960 | 1 | exon | (9667717-9669871) | 717 | DWY | 16 | 41-L1-S1-P1-32-S1-P1-L1-S1-P1-L1-S1-P2-L2-S2-E1-E2-DYW |
| PtrPPR589 | Potri.018G079600.1 | POPTR_0018s05820.1 | Chr18 | - | 10531326 | 10534071 | 2 | exon-exon | (10531869-10532096)(10532534-10533778) | 490 | P | 9 | 122-P-P-P-P-P-P-P-41-P-P-11 |
| PtrPPR590 | Potri.018G081700.1 | POPTR_0018s08840.1 | Chr18 | + | 10882167 | 10886085 | 3 | exon-exon-exon | (10882942-10882995)(10883873-10884784)(10884873-10885760) | 616 | P | 10 | 124-P-P-P-P-P-P-P-P-P-P-142 |
| PtrPPR591 | Potri.018G085800.1 | POPTR_0018s09280.1 | Chr18 | - | 11279288 | 11282044 | 1 | exon | (11279288-11281376) | 695 | DYW | 16 | 57-S1-SS-P1-L1-S1-P1-P1-P1-L1-S1-P2-L2-S2-E1-E2-DYW |
| PtrPPR592 | Potri.018G092400.1 | POPTR_0018s10010.1 | Chr18 | - | 12181720 | 12183678 | 1 | exon | (12181720-12183544) | 607 | DYW | 14 | 18-L1-S1-P1-L1-S1-P1-L1-S1-P2-L2-S2-E1-E2-DYW |
| PtrPPR593 | Potri.018G093700.1 | POPTR_0018s10150.1 | Chr18 | + | 12263469 | 12266953 | 2 | exon-exon | (12264896-12266218) | 440 | P | 7 | 158-P-P-P-P-P-P-P-25 |
| PtrPPR594 | Potri.018G143300.1 |  | Chr18 | - | 16295556 | 16296330 | 3 | exon-exon-exon | (16295571-16295751)(16295804-16296173)(16296210-16296330) | 223 | E2 | 5 | S1-P2-23-S2-E1-E2-43 |
| PtrPPR595 | Potri.018G143800.1 | POPTR_0018s14060.1 | Chr18 | + | 16342876 | 16348630 | 1 | exon | (16342876-16345855) | 992 | P | 22 | 50-P-P-P-P-P-P-P-P-P-P-P-P-P-69-P-33-P-P-P-P-P-P-P-63-P |
| PtrPPR596 | Potri.018G145000.1 |  | Chr18 | - | 16475247 | 16475976 | 2 | exon-exon | (16475247-16475298)(16475588-16475976) | 146 | E1 | 4 | 1-P2-L2-S2-E1-6 |
| PtrPPR597 | Potri.018G146900.1 | POPTR_0018s14360.1 | Chr18 | + | 16619180 | 16620972 | 2 | exon-exon | (16619241-16619366)(16619813-16620972) | 428 | P | 6 | 58-P-P-P-P-70-P-35-P-54 |
| PtrPPR598 | Potri.018G147900.1 | POPTR_0018s14430.1 | Chr18 | + | 16652290 | 16654117 | 5 | exon-exon-exon-exon-exon | (16652290-16652459)(16652541-16653027)(16653436-16653487)(16653517-16653894)(16653936-16654117) | 422 | PLS | 9 | 45-L1-S1-P1-L1-S1-P1-P1-L1-S1-83 |
| PtrPPR599 | Potri.018G152300.1 | POPTR_0018s14930.1 | Chr18 | + | 16928068 | 16929884 | 1 | exon | (16928068-16929832) | 587 | DYW | 14 | 7-L1-S1-P1-L1-S1-P1-L1-S1-P2-L2-S2-E1-E2-DYW |
| PtrPPR600 | Potri.019G019200.1 | POPTR_0019s03610.1 | Chr19 | + | 2247057 | 2250280 | 1 | exon | (2248021-2249802) | 593 | P | 13 | 133-P-P-P-P-P-P-P-P-P-P-P-P-P-5 |
| PtrPPR601 | Potri.019G019300.1 | POPTR_0019s03630.1 | Chr19 | - | 2250928 | 2253512 | 1 | exon | (2251162-2252922) | 586 | P | 14 | 72-P-P-P-P-P-P-P-P-P-P-P-P-P-P-24 |
| PtrPPR602 | Potri.019G021200.1 | POPTR_0019s03770.1 | Chr19 | - | 2413135 | 2415638 | 1 | exon | (2413266-2415026) | 586 | P | 13 | 125-P-P-P-P-P-P-P-P-P-P-P-P-P-6 |
| PtrPPR603 | Potri.019G025100.1 | POPTR_0019s04180.1 | Chr19 | + | 2857530 | 2865486 | 12 | exon-exon-exon-exon-exon-exon-exon-exon-exon-exon-exon-exon | (2857587-2857943)(2858070-2858241)(2858333-2858550)(2858861-2858921)(2859419-2859491)(2860052-2860129)(2861075-2861232)(2862260-2862428)(2862666-2863128)(2863339-2863749)(2864077-2864742)(2864909-2865083) | 890 | P | 6 | 134-P-34-P-37-P-P-P-439-P-23 |
| PtrPPR604 | Potri.019G025700.1 | POPTR_0019s04240.1 | Chr19 | + | 2933306 | 2935426 | 1 | exon | (2933343-2934698) | 451 | P | 9 | 137-P-P-P-P-P-P-P-P-P |
| PtrPPR605 | Potri.019G043100.1 | POPTR_0019s06000.1 | Chr19 | + | 5033625 | 5036468 | 4 | exon-exon-exon-exon-exon | (5033625-5033838)(5034292-5035229)(5035719-5036205)(5036302-5036468 ) | 601 | P | 14 | 87-P-P-P-P-P-P-P-P-P-P-P-P-P-15-P |
| PtrPPR606 | Potri.019G049400.1 | POPTR_0019s07590.1 | Chr19 | + | 7384481 | 7390659 | 8 | exon-exon-exon-exon-exon-exon-exon-exon | (7384481-7385021)(7385308-7386697)(7386831-7387142)(7387648-7387752)(7387830-7387917)(7388035-7388156)(7389582-7389646)(7390067-7390659) | 1071 | p | 24 | 186-P-P-P-P-P-P-P-P-P-P-P-26-P-P-P-P-P-P-P-P-P-P-P-P-P-24 |
| PtrPPR607 | Potri.019G062300.1 | POPTR_0019s09040.1 | Chr19 | + | 9486350 | 9487751 | 4 | exon-exon-exon-exon | (9486350-9486588)(9486785-9487361)(9487459-9487498)(9487528-9487751) | 359 | P | 8 | 9-P-P-P-P-P-P-59-P-P-6 |
| PtrPPR608 | Potri.019G074700.1 | scaffold_19:F:11928400-11930601 | Chr19 | + | 10897415 | 10900545 | 1 | exon | (10898778-10900433) | 551 | E+ | 15 | 9-L1-S1-P1-P1-L1-S1-P1-L1-S1-P2-L2-S2-E1-E2-E+ |
| PtrPPR609 | Potri.019G075400.1 | POPTR_0019s10410.1 | Chr19 | + | 10969552 | 10972214 | 1 | exon | (10969605-10971650) | 681 | PLS | 17 | 105-L1-S1-P1-L1-S1-P1-L1-S1-P1-L1-S1-P1-L1-S1-P2-L2-S2 |
| PtrPPR610 | Potri.019G075900.1 | POPTR_0019s10460.1 | Chr19 | - | 11019826 | 11023797 | 1 | exon | (11019870-11021363) | 497 | P | 6 | 188-P-65-P-P-P-P-P-34 |
| PtrPPR611 | Potri.019G091000.1 | POPTR_0019s12080.1 | Chr19 | - | 12208024 | 12210508 | 1 | exon | (12208071-12210284) | 737 | PLS | 20 | 10-L1-S1-P1-34-S1-P1-L1-S1-P1-L1-S1-P1-30-S1-P1-P1-L1-S1-P2-L2-S2-E1 |
| PtrPPR612 | Potri.019G099700.1 |  | Chr19 | + | 12951471 | 12954374 | 3 | exon-exon-exon | (12951471-12951711)(12952146-12952345)(12954222-12954374) | 197 | P | 6 | P-P-P-17-P-P-P-15 |
| PtrPPR613 | Potri.019G102100.1 |  | Chr19 | + | 13119339 | 13119764 | 1 | exon | (13119351-13119764) | 137 | P | 3 | 15-P-P-P-17 |
| PtrPPR614 | Potri.019G103400.1 | POPTR_0019s13440.1 | Chr19 | + | 13223513 | 13226241 | 1 | exon | (13223525-13225504) | 659 | P | 14 | 155-P-P-P-P-P-P-P-P-P-P-P-P-P-P-15 |
| PtrPPR615 | Potri.T035800.1 | POPTR_0002s20840.1 | scaffold_36 | - | 288110 | 288827 | 2 | exon-exon | (288110-288513)(288575-288827) | 218 | E2 | 6 | L1-S1-P2-L2-13-E1-E2-3 |
| PtrPPR616 | Potri.T071500.1 | POPTR_0194s00200.1 | scaffold_76 | + | 97684 | 104285 | 8 | exon-exon-exon-exon-exon-exon-exon-exon | (97684-98224)(98517-99906)(100041-100352)(100859-100963)(101039-101126)(101244-101365)(102858-102922)(103344-103937) | 1071 | P | 22 | 186-P-P-P-P-P-P-P-P-P-P-P-59-P-P-P-P-P-P-P-P-P-P-P-58 |
| PtrPPR617 | Potri.T120800.1 |  | scaffold_201 | - | 72622 | 74884 | 2 | exon-exon | reverse(72846-72865)(72987-74541) | 524 | P | 13 | 63-P-P-P-P-P-P-P-P-P-P-P-P-P-6 |
| PtrPPR618 | Potri.T130900.1 | POPTR_0018s03860.1 | scaffold_250 | + | 35264 | 39688 | 2 | exon-exon | (35524-35651)(36373-37843) | 532 | P | 13 | 61-P-P-P-P-P-P-P-P-P-P-P-P-P-14 |
| PtrPPR619 | Potri.T137000.1 | POPTR_0015s02060.1 | scaffold_291 | + | 32616 | 37236 | 3 | exon-exon-exon | (32616-33006)(34201-34820)(35582-37130) | 852 | DYW | 19 | 112-P1-L1-S1-P1-L1-S1-SS-P1-L1-S1-P1-L1-S1-P2-L2-S2-E1-E2-DYW |
| PtrPPR620 | Potri.T146600.1 | POPTR_0006s05170.1 | scaffold_463 | + | 12277 | 14568 | 2 | exon-exon | (12277-13943)(14055-14568) | 726 | E+ | 18 | 72-L1-S1-P1-L1-S1-31-L1-S1-P1-L1-S1-P1-L1-S1-P2-S2-E1-E2-E+ |
| PtrPPR621 | Potri.T146900.1 |  | scaffold_467 | + | 13731 | 15119 | 2 | exon-exon | (13731-14192)(14658-15119) | 307 | PLS | 7 | 12-L1-S1-P1-20-S1-P2-L2-S2-38 |
| PtrPPR622 | Potri.T151200.1 | POPTR_0013s05050.1 | scaffold_531 | + | 2621 | 4505 | 1 | exon | (2621-4178) | 518 | P | 9 | 88-P-P-P-P-P-P-P-32-P-P-80 |
| PtrPPR623 | Potri.T161700.1 | POPTR_0516s00230.1 | scaffold_752 | + | 8220 | 10606 | 5 | exon-exon-exon-exon-exon | (8220-8458)(9012-9337)(9369-9446)(9709-10353)(10395-10606) | 499 | E1 | 11 | 68-L1-S1-P1-L1-S1-L1-S1-P2-L2-S2-E1-58 |
| PtrPPR624 | Potri.T178000.1 | POPTR_2670s00200.1 | scaffold_1726 | + | 102 | 5903 | 1 | exon | (102-972)(5359-5903) | 471 | P | 12 | 9-P-P-P-P-P-P-P-P-P-P-P-P-31 |
| PtrPPR625 | Potri.T178100.1 |  | scaffold_1726 | + | 4079 | 5041 | 2 | exon-exon | (4079-4159)(4553-5041) | 189 | P | 5 | 25-P-P-P-P-P |
| PtrPPR626 | Potri.T178500.1 | POPTR_1313s00210.1 | scaffold_1748 | + | 4479 | 5979 | 1 | exon | (4479-5670) | 396 | PLS | 6 | 93-S1-P1-P1-P1-P1-P1-92 |

| **Supplemental Table 2. Other PPR proteins from different species** | | | | | | | | | | | | | | | | | | | | |  |  |  |  |  |  |  |  |  |
| --- | --- | --- | --- | --- | --- | --- | --- | --- | --- | --- | --- | --- | --- | --- | --- | --- | --- | --- | --- | --- | --- | --- | --- | --- | --- | --- | --- | --- | --- |
| **Common name** | | | | | **Species** | | | | | | | | | **Gene ID** | | | | | | |  |  |  |  |  |  |  |  |  |
| CLB19 | | | | | *Arabidopsis thaliana* | | | | | | | | | AT1G05750.1 | | | | | | |  |  |  |  |  |  |  |  |  |
| VAC1 | | | | | *Arabidopsis thaliana* | | | | | | | | | AT1G15510.1 | | | | | | |  |  |  |  |  |  |  |  |  |
| *YS1* | | | | | *Arabidopsis thaliana* | | | | | | | | | AT3G22690.1 | | | | | | |  |  |  |  |  |  |  |  |  |
| SLO1 | | | | | *Arabidopsis thaliana* | | | | | | | | | AT2G22410.1 | | | | | | |  |  |  |  |  |  |  |  |  |
| SLO2 | | | | | *Arabidopsis thaliana* | | | | | | | | | AT2G13600.1 | | | | | | |  |  |  |  |  |  |  |  |  |
| RIP1 | | | | | *Arabidopsis thaliana* | | | | | | | | | AT3G15000.1 | | | | | | |  |  |  |  |  |  |  |  |  |
| OTP87 | | | | | *Arabidopsis thaliana* | | | | | | | | | AT1G74600.1 | | | | | | |  |  |  |  |  |  |  |  |  |
| OTP43 | | | | | *Arabidopsis thaliana* | | | | | | | | | AT1G74900.1 | | | | | | |  |  |  |  |  |  |  |  |  |
| GRS1 | | | | | *Arabidopsis thaliana* | | | | | | | | | AT4G32430.1 | | | | | | |  |  |  |  |  |  |  |  |  |
| EMB3103 | | | | | *Arabidopsis thaliana* | | | | | | | | | AT1G10910.1 | | | | | | |  |  |  |  |  |  |  |  |  |
| EMB88 | | | | | *Arabidopsis thaliana* | | | | | | | | | AT1G30610.1 | | | | | | |  |  |  |  |  |  |  |  |  |
| PTAC2 | | | | | *Arabidopsis thaliana* | | | | | | | | | AT1G74850.1 | | | | | | |  |  |  |  |  |  |  |  |  |
| EMB975 | | | | | *Arabidopsis thaliana* | | | | | | | | | AT2G01860.1 | | | | | | |  |  |  |  |  |  |  |  |  |
| AtPPR2 | | | | | *Arabidopsis thaliana* | | | | | | | | | AT3G06430.1 | | | | | | |  |  |  |  |  |  |  |  |  |
| EMB1270 | | | | | *Arabidopsis thaliana* | | | | | | | | | AT3G18110.1 | | | | | | |  |  |  |  |  |  |  |  |  |
| EMB2261 | | | | | *Arabidopsis thaliana* | | | | | | | | | AT3G49170.1 | | | | | | |  |  |  |  |  |  |  |  |  |
| EMB1796 | | | | | *Arabidopsis thaliana* | | | | | | | | | AT3G49240.1 | | | | | | |  |  |  |  |  |  |  |  |  |
| EMB3131 | | | | | *Arabidopsis thaliana* | | | | | | | | | AT4G20740.1 | | | | | | |  |  |  |  |  |  |  |  |  |
| EMB1025 | | | | | *Arabidopsis thaliana* | | | | | | | | | AT4G20090.1 | | | | | | |  |  |  |  |  |  |  |  |  |
| MRL1 | | | | | *Arabidopsis thaliana* | | | | | | | | | AT4G34830.1 | | | | | | |  |  |  |  |  |  |  |  |  |
| AtPPR5 | | | | | *Arabidopsis thaliana* | | | | | | | | | AT4G39620.1 | | | | | | |  |  |  |  |  |  |  |  |  |
| EMB175 | | | | | *Arabidopsis thaliana* | | | | | | | | | AT5G03800.1 | | | | | | |  |  |  |  |  |  |  |  |  |
| AtPPR4 | | | | | *Arabidopsis thaliana* | | | | | | | | | AT5G04810.1 | | | | | | |  |  |  |  |  |  |  |  |  |
| EMB976 | | | | | *Arabidopsis thaliana* | | | | | | | | | AT5G27270.1 | | | | | | |  |  |  |  |  |  |  |  |  |
| EMB3140 | | | | | *Arabidopsis thaliana* | | | | | | | | | AT5G39980.1 | | | | | | |  |  |  |  |  |  |  |  |  |
| EMB1006 | | | | | *Arabidopsis thaliana* | | | | | | | | | AT5G50280.1 | | | | | | |  |  |  |  |  |  |  |  |  |
| EMB3141 | | | | | *Arabidopsis thaliana* | | | | | | | | | AT5G50390.1 | | | | | | |  |  |  |  |  |  |  |  |  |
| DG1 | | | | | *Arabidopsis thaliana* | | | | | | | | | AT5G67570.1 | | | | | | |  |  |  |  |  |  |  |  |  |
| GRP23 | | | | | *Arabidopsis thaliana* | | | | | | | | | AT1G10270.1 | | | | | | |  |  |  |  |  |  |  |  |  |
| PPR40 | | | | | *Arabidopsis thaliana* | | | | | | | | | AT3G16890.1 | | | | | | |  |  |  |  |  |  |  |  |  |
| ABO5 | | | | | *Arabidopsis thaliana* | | | | | | | | | AT1G51965.1 | | | | | | |  |  |  |  |  |  |  |  |  |
| AHG1 | | | | | *Arabidopsis thaliana* | | | | | | | | | AT2G44880.1 | | | | | | |  |  |  |  |  |  |  |  |  |
| LOI1 | | | | | *Arabidopsis thaliana* | | | | | | | | | AT4G14850.1 | | | | | | |  |  |  |  |  |  |  |  |  |
| EMP4 | | | | | *Zea mays* | | | | | | | | | GRMZM2G092198_T01 | | | | | | |  |  |  |  |  |  |  |  |  |
| Rf1 | | | | | *Oryza sativa* | | | | | | | | | LOC_Os10g35436.1 | | | | | | |  |  |  |  |  |  |  |  |  |
| SLO3 | | | | | *Arabidopsis thaliana* | | | | | | | | | AT3G61360.1 | | | | | | |  |  |  |  |  |  |  |  |  |
| LPA66 | | | | | *Arabidopsis thaliana* | | | | | | | | | AT5G48910.1 | | | | | | |  |  |  |  |  |  |  |  |  |
| CRR2 | | | | | *Arabidopsis thaliana* | | | | | | | | | AT3G46790.1 | | | | | | |  |  |  |  |  |  |  |  |  |
| HCF152 | | | | | *Arabidopsis thaliana* | | | | | | | | | AT3G09650.1 | | | | | | |  |  |  |  |  |  |  |  |  |
| PGR3 | | | | | *Arabidopsis thaliana* | | | | | | | | | AT4G31850.1 | | | | | | |  |  |  |  |  |  |  |  |  |
| SVR7 | | | | | *Arabidopsis thaliana* | | | | | | | | | AT4G16390.1 | | | | | | |  |  |  |  |  |  |  |  |  |
| CRR2 | | | | | *Arabidopsis thaliana* | | | | | | | | | AT3G46790.1 | | | | | | |  |  |  |  |  |  |  |  |  |
| AtPPR4 | | | | | *Arabidopsis thaliana* | | | | | | | | | AT1G14470.1 | | | | | | |  |  |  |  |  |  |  |  |  |
| SLG1 | | | | | *Arabidopsis thaliana* | | | | | | | | | AT5G08490.1 | | | | | | |  |  |  |  |  |  |  |  |  |
| PGN | | | | | *Arabidopsis thaliana* | | | | | | | | | AT1G56570.1 | | | | | | |  |  |  |  |  |  |  |  |  |
| LOJ | | | | | *Arabidopsis thaliana* | | | | | | | | | AT2G39230.1 | | | | | | |  |  |  |  |  |  |  |  |  |
| SOAR1 | | | | | *Arabidopsis thaliana* | | | | | | | | | AT5G11310.1 | | | | | | |  |  |  |  |  |  |  |  |  |
| **Supplemental Table 3. MicroRNA target site in *PtrPPR* genes** | | | | | | | | | | | | | | | | | | | | | | | | | | | | | |
| **miRNA_Acc.** | | | **miRNA_Acc.** | | | **Gene Name** | | | **Target_Acc.** | | **Expectation** | **UPE** | | **miRNA_start** | | | **miRNA_end** | **Target_start** | **Target_end** | | **miRNA_aligned_fragment** | | | | **Target_aligned_fragment** | | | **Inhibition** | **Multiplicity** |
| PtrmiR1448 | | | PtrmiR1448 | | | PtrPPR259 | | | Potri.006G155400.1 | | 3 | 16.499 | | 1 | | | 19 | 1585 | 1604 | | CUUUCCAAC-GCCUCCCAUA | | | | UAUGGGAUGCAGUUGGAAAG | | | Translation | 1 |
| PtrmiR156 | | | PtrmiR156l | | | PtrPPR274 | | | Potri.006G223300.1 | | 3 | 18.106 | | 1 | | | 21 | 1539 | 1559 | | UUGACAGAAGAUGGAGAGCAC | | | | GAGCUUUUCAUUUUUUGUCAA | | | Cleavage | 1 |
| PtrmiR396 | | | PtrmiR396a | | | PtrPPR17 | | | Potri.001G116300.1 | | 3 | 22.077 | | 1 | | | 20 | 1072 | 1091 | | UUCCACAGCUUUCUUGAACU | | | | AGAUUGAGGAAGUUGUGGAA | | | Cleavage | 1 |
|  |  |  | PtrmiR396a | | | PtrPPR121 | | | Potri.003G116100.1 | | 2.5 | 16.196 | | 1 | | | 20 | 1766 | 1785 | | UUCCACAGCUUUCUUGAACU | | | | AGAUUGAGAAAGUUGUGGAA | | | Cleavage | 1 |
|  |  |  | PtrmiR396a | | | PtrPPR485 | | | Potri.014G116600.1 | | 2.5 | 16.526 | | 1 | | | 20 | 988 | 1007 | | UUCCACAGCUUUCUUGAACU | | | | AGGUGAAGGAAGCUGUGGAA | | | Cleavage | 1 |
|  |  |  | PtrmiR396b | | | PtrPPR17 | | | Potri.001G116300.1 | | 3 | 22.077 | | 1 | | | 20 | 1072 | 1091 | | UUCCACAGCUUUCUUGAACU | | | | AGAUUGAGGAAGUUGUGGAA | | | Cleavage | 1 |
|  |  |  | PtrmiR396b | | | PtrPPR121 | | | Potri.003G116100.1 | | 2.5 | 16.196 | | 1 | | | 20 | 1766 | 1785 | | UUCCACAGCUUUCUUGAACU | | | | AGAUUGAGAAAGUUGUGGAA | | | Cleavage | 1 |
|  |  |  | PtrmiR396b | | | PtrPPR485 | | | Potri.014G116600.1 | | 2.5 | 16.526 | | 1 | | | 20 | 988 | 1007 | | UUCCACAGCUUUCUUGAACU | | | | AGGUGAAGGAAGCUGUGGAA | | | Cleavage | 1 |
|  |  |  | PtrmiR396c | | | PtrPPR17 | | | Potri.001G116300.1 | | 3 | 22.077 | | 1 | | | 21 | 1071 | 1091 | | UUCCACAGCUUUCUUGAACUU | | | | GAGAUUGAGGAAGUUGUGGAA | | | Cleavage | 1 |
|  |  |  | PtrmiR396c | | | PtrPPR121 | | | Potri.003G116100.1 | | 2.5 | 16.196 | | 1 | | | 21 | 1765 | 1785 | | UUCCACAGCUUUCUUGAACUU | | | | GAGAUUGAGAAAGUUGUGGAA | | | Cleavage | 1 |
|  |  |  | PtrmiR396c | | | PtrPPR485 | | | Potri.014G116600.1 | | 2.5 | 16.526 | | 1 | | | 21 | 987 | 1007 | | UUCCACAGCUUUCUUGAACUU | | | | AAGGUGAAGGAAGCUGUGGAA | | | Cleavage | 1 |
|  |  |  | PtrmiR396d | | | PtrPPR17 | | | Potri.001G116300.1 | | 3 | 22.077 | | 1 | | | 21 | 1071 | 1091 | | UUCCACAGCUUUCUUGAACUU | | | | GAGAUUGAGGAAGUUGUGGAA | | | Cleavage | 1 |
|  |  |  | PtrmiR396d | | | PtrPPR121 | | | Potri.003G116100.1 | | 2.5 | 16.196 | | 1 | | | 21 | 1765 | 1785 | | UUCCACAGCUUUCUUGAACUU | | | | GAGAUUGAGAAAGUUGUGGAA | | | Cleavage | 1 |
|  |  |  | PtrmiR396d | | | PtrPPR485 | | | Potri.014G116600.1 | | 2.5 | 16.526 | | 1 | | | 21 | 987 | 1007 | | UUCCACAGCUUUCUUGAACUU | | | | AAGGUGAAGGAAGCUGUGGAA | | | Cleavage | 1 |
|  |  |  | PtrmiR396e-5p | | | PtrPPR17 | | | Potri.001G116300.1 | | 3 | 22.077 | | 1 | | | 21 | 1071 | 1091 | | UUCCACAGCUUUCUUGAACUU | | | | GAGAUUGAGGAAGUUGUGGAA | | | Cleavage | 1 |
|  |  |  | PtrmiR396e-5p | | | PtrPPR121 | | | Potri.003G116100.1 | | 2.5 | 16.196 | | 1 | | | 21 | 1765 | 1785 | | UUCCACAGCUUUCUUGAACUU | | | | GAGAUUGAGAAAGUUGUGGAA | | | Cleavage | 1 |
|  |  |  | PtrmiR396e-5p | | | PtrPPR485 | | | Potri.014G116600.1 | | 2.5 | 16.526 | | 1 | | | 21 | 987 | 1007 | | UUCCACAGCUUUCUUGAACUU | | | | AAGGUGAAGGAAGCUGUGGAA | | | Cleavage | 1 |
|  |  |  | PtrmiR396f | | | PtrPPR121 | | | Potri.003G116100.1 | | 3 | 16.196 | | 1 | | | 20 | 1766 | 1785 | | UUCCACGGCUUUCUUGAACU | | | | AGAUUGAGAAAGUUGUGGAA | | | Cleavage | 1 |
|  |  |  | PtrmiR396f | | | PtrPPR485 | | | Potri.014G116600.1 | | 3 | 16.526 | | 1 | | | 20 | 988 | 1007 | | UUCCACGGCUUUCUUGAACU | | | | AGGUGAAGGAAGCUGUGGAA | | | Cleavage | 1 |
|  |  |  | PtrmiR396g-5p | | | PtrPPR121 | | | Potri.003G116100.1 | | 3 | 16.196 | | 1 | | | 21 | 1765 | 1785 | | UUCCACGGCUUUCUUGAACUU | | | | GAGAUUGAGAAAGUUGUGGAA | | | Cleavage | 1 |
|  |  |  | PtrmiR396g-5p | | | PtrPPR485 | | | Potri.014G116600.1 | | 3 | 16.526 | | 1 | | | 21 | 987 | 1007 | | UUCCACGGCUUUCUUGAACUU | | | | AAGGUGAAGGAAGCUGUGGAA | | | Cleavage | 1 |
| PtrmiR472 | | | PtrmiR472a | | | PtrPPR169 | | | Potri.004G209500.1 | | 3 | 23.145 | | 1 | | | 20 | 1066 | 1085 | | UUUUCCCUACUCCACCCAUC | | | | GAUGGCUUGUGUAGGGAAAA | | | Translation | 1 |
|  |  |  | PtrmiR472b | | | PtrPPR181 | | | Potri.005G014500.1 | | 2.5 | 9.933 | | 1 | | | 20 | 1323 | 1342 | | UUUUCCCAACUCCACCCAUC | | | | GAUGGGUAGAAUUGGGAAGA | | | Translation | 1 |
|  |  |  | PtrmiR472b | | | PtrPPR182 | | | Potri.005G017500.1 | | 2.5 | 9.933 | | 1 | | | 20 | 1173 | 1192 | | UUUUCCCAACUCCACCCAUC | | | | GAUGGGUAGAAUUGGGAAGA | | | Translation | 1 |
| PtrmiR474 | | | PtrmiR474a | | | PtrPPR80 | | | Potri.002G176100.1 | | 2 | 15.442 | | 1 | | | 20 | 1778 | 1797 | | CAAAAGUUGCUGGGUUUGGC | | | | GCCAAAACCAGAAACUUUUG | | | Translation | 1 |
|  |  |  | PtrmiR474a | | | PtrPPR250 | | | Potri.006G076100.1 | | 3 | 15.137 | | 1 | | | 21 | 1205 | 1225 | | CAAAAGUUGCUGGGUUUGGCU | | | | AGCCAGAUUCUGUAACUUUUG | | | Translation | 1 |
|  |  |  | PtrmiR474b | | | PtrPPR80 | | | Potri.002G176100.1 | | 2.5 | 15.442 | | 1 | | | 20 | 1778 | 1797 | | CAAAAGUUGUUGGGUUUGGC | | | | GCCAAAACCAGAAACUUUUG | | | Translation | 1 |
|  |  |  | PtrmiR474b | | | PtrPPR133 | | | Potri.004G018000.1 | | 3 | 10.948 | | 1 | | | 23 | 262 | 284 | | CAAAAGUUGUUGGGUUUGGCUGG | | | | CUCGCCAAAUCCAACAACUUCUC | | | Cleavage | 1 |
|  |  |  | PtrmiR474b | | | PtrPPR214 | | | Potri.005G137900.1 | | 3 | 18.748 | | 1 | | | 22 | 1254 | 1275 | | CAAAAGUUGUUGGGUUUGGCUG | | | | CUGCCUAACUCAAUUACUUUUG | | | Cleavage | 1 |
|  |  |  | PtrmiR474b | | | PtrPPR216 | | | Potri.005G151200.1 | | 3 | 22.078 | | 1 | | | 24 | 1442 | 1465 | | CAAAAGUUGUUGGGUUUGGCUGGG | | | | UCAGGCCAAACCAGAUCACUUUUG | | | Cleavage | 1 |
|  |  |  | PtrmiR474b | | | PtrPPR217 | | | Potri.005G155000.1 | | 3 | 22.078 | | 1 | | | 24 | 1172 | 1195 | | CAAAAGUUGUUGGGUUUGGCUGGG | | | | UCAGGCCAAACCAGAUCACUUUUG | | | Cleavage | 1 |
|  |  |  | PtrmiR474b | | | PtrPPR399 | | | Potri.011G001100.1 | | 3 | 7.681 | | 1 | | | 23 | 253 | 275 | | CAAAAGUUGUUGGGUUUGGCUGG | | | | CUCGCCAAAUCCAACAACUUCUC | | | Cleavage | 1 |
| PtrmiR475 | | | PtrmiR475a-3p | | | PtrPPR49 | | | Potri.001G368700.1 | | 2.5 | 11.927 | | 1 | | | 21 | 1128 | 1148 | | UUACAGUGCCCAUUGAUUAAG | | | | UUUGAUUGAUGGGCAUUGUGA | | | Cleavage | 1 |
|  |  |  | PtrmiR475a-3p | | | PtrPPR120 | | | Potri.003G105700.1 | | 2 | 8.505 | | 1 | | | 20 | 1117 | 1136 | | UUACAGUGCCCAUUGAUUAA | | | | UUGAUUGAUGGGUACUGUAA | | | Cleavage | 1 |
|  |  |  | PtrmiR475a-3p | | | PtrPPR142 | | | Potri.004G066600.1 | | 2 | 18.029 | | 1 | | | 21 | 2208 | 2228 | | UUACAGUGCCCAUUGAUUAAG | | | | UUUGAUUGAUGGGCAUUGUAA | | | Cleavage | 1 |
|  |  |  | PtrmiR475a-3p | | | PtrPPR145 | | | Potri.004G074500.1 | | 2.5 | 19 | | 1 | | | 21 | 668 | 688 | | UUACAGUGCCCAUUGAUUAAG | | | | CUUAACCAAUGGGCUCUGUAA | | | Cleavage | 1 |
|  |  |  | PtrmiR475a-3p | | | PtrPPR146 | | | Potri.004G074700.1 | | 3 | 20.552 | | 1 | | | 21 | 423 | 443 | | UUACAGUGCCCAUUGAUUAAG | | | | CUUAAUCAAUGGGCUCGGUAA | | | Cleavage | 1 |
|  |  |  | PtrmiR475a-3p | | | PtrPPR274 | | | Potri.006G223300.1 | | 3 | 16.134 | | 1 | | | 21 | 705 | 725 | | UUACAGUGCCCAUUGAUUAAG | | | | CUUGAUUAAUGGUUAUUGUAA | | | Translation | 1 |
|  |  |  | PtrmiR475a-3p | | | PtrPPR276 | | | Potri.006G242200.1 | | 1.5 | 12.735 | | 1 | | | 21 | 510 | 530 | | UUACAGUGCCCAUUGAUUAAG | | | | CUUAAUCAAUGGGCUCUGUAA | | | Cleavage | 1 |
|  |  |  | PtrmiR475a-3p | | | PtrPPR277 | | | Potri.006G242500.1 | | 2.5 | 14.973 | | 1 | | | 21 | 652 | 672 | | UUACAGUGCCCAUUGAUUAAG | | | | CUUAAUCAAUUGGCUCUGUAA | | | Translation | 2 |
|  |  |  | PtrmiR475a-3p | | | PtrPPR277 | | | Potri.006G242500.1 | | 2.5 | 11.886 | | 1 | | | 21 | 1282 | 1302 | | UUACAGUGCCCAUUGAUUAAG | | | | CUUAAUUAAUGGAUAUUGUAA | | | Translation | 2 |
|  |  |  | PtrmiR475a-3p | | | PtrPPR284 | | | Potri.006G257300.1 | | 1.5 | 16.223 | | 1 | | | 21 | 779 | 799 | | UUACAGUGCCCAUUGAUUAAG | | | | CUUAAUCAAUGGGCUCUGUAA | | | Cleavage | 2 |
|  |  |  | PtrmiR475a-3p | | | PtrPPR284 | | | Potri.006G257300.1 | | 2.5 | 12.947 | | 1 | | | 21 | 1409 | 1429 | | UUACAGUGCCCAUUGAUUAAG | | | | CUUAAUUAAUGGAUAUUGUAA | | | Translation | 2 |
|  |  |  | PtrmiR475a-3p | | | PtrPPR287 | | | Potri.006G271200.1 | | 1.5 | 16.349 | | 1 | | | 21 | 389 | 409 | | UUACAGUGCCCAUUGAUUAAG | | | | CUUAAUCAAUGGGCUCUGUAA | | | Cleavage | 1 |
|  |  |  | PtrmiR475a-3p | | | PtrPPR288 | | | Potri.006G271400.1 | | 1.5 | 17.779 | | 1 | | | 21 | 504 | 524 | | UUACAGUGCCCAUUGAUUAAG | | | | CUUAAUCAAUGGGCUCUGUAA | | | Cleavage | 2 |
|  |  |  | PtrmiR475a-3p | | | PtrPPR288 | | | Potri.006G271400.1 | | 2.5 | 12.912 | | 1 | | | 21 | 1134 | 1154 | | UUACAGUGCCCAUUGAUUAAG | | | | CUUAAUUAAUGGAUAUUGUAA | | | Translation | 2 |
|  |  |  | PtrmiR475a-3p | | | PtrPPR297 | | | Potri.007G068400.1 | | 3 | 15.233 | | 1 | | | 21 | 674 | 694 | | UUACAGUGCCCAUUGAUUAAG | | | | CUUCAUCAAUGGGUACUGCAA | | | Cleavage | 1 |
|  |  |  | PtrmiR475a-3p | | | PtrPPR312 | | | Potri.008G044000.1 | | 2.5 | 20.085 | | 1 | | | 21 | 240 | 260 | | UUACAGUGCCCAUUGAUUAAG | | | | UUUGAUAAGUGGGCAUUGUAA | | | Cleavage | 1 |
|  |  |  | PtrmiR475a-3p | | | PtrPPR352 | | | Potri.009G105600.1 | | 3 | 13.222 | | 1 | | | 21 | 1569 | 1589 | | UUACAGUGCCCAUUGAUUAAG | | | | CUUGAUUAAUGGUUAUUGUAA | | | Translation | 1 |
|  |  |  | PtrmiR475a-3p | | | PtrPPR366 | | | Potri.010G035700.1 | | 3 | 12.456 | | 1 | | | 21 | 2038 | 2058 | | UUACAGUGCCCAUUGAUUAAG | | | | UUUGAUAAAAGGGCAUUGUAA | | | Cleavage | 1 |
|  |  |  | PtrmiR475a-3p | | | PtrPPR394 | | | Potri.010G234500.1 | | 3 | 14.611 | | 1 | | | 20 | 1465 | 1484 | | UUACAGUGCCCAUUGAUUAA | | | | UUGAUUGAUGGCUACUGUAA | | | Translation | 1 |
|  |  |  | PtrmiR475a-3p | | | PtrPPR400 | | | Potri.011G003700.1 | | 2.5 | 14.923 | | 1 | | | 21 | 1473 | 1493 | | UUACAGUGCCCAUUGAUUAAG | | | | UUUGAUCGAUGGGCACUGCAA | | | Cleavage | 1 |
|  |  |  | PtrmiR475a-3p | | | PtrPPR406 | | | Potri.011G057900.1 | | 2 | 14.06 | | 1 | | | 20 | 1049 | 1068 | | UUACAGUGCCCAUUGAUUAA | | | | UUAAUUAAUGGGCUCUGUAA | | | Cleavage | 1 |
|  |  |  | PtrmiR475a-3p | | | PtrPPR441 | | | Potri.013G032600.1 | | 1.5 | 20.164 | | 1 | | | 20 | 1069 | 1088 | | UUACAGUGCCCAUUGAUUAA | | | | UUGAUCAACGGGCACUGUAA | | | Cleavage | 1 |
|  |  |  | PtrmiR475a-3p | | | PtrPPR442 | | | Potri.013G034200.1 | | 1.5 | 17.057 | | 1 | | | 20 | 1186 | 1205 | | UUACAGUGCCCAUUGAUUAA | | | | UUGAUCAAGGGGCACUGUAA | | | Cleavage | 1 |
|  |  |  | PtrmiR475a-3p | | | PtrPPR443 | | | Potri.013G034300.1 | | 1.5 | 15.858 | | 1 | | | 20 | 676 | 695 | | UUACAGUGCCCAUUGAUUAA | | | | UUGAUCAAGGGGCACUGUAA | | | Cleavage | 1 |
|  |  |  | PtrmiR475a-3p | | | PtrPPR444 | | | Potri.013G034400.1 | | 1 | 17.242 | | 1 | | | 20 | 1130 | 1149 | | UUACAGUGCCCAUUGAUUAA | | | | CUAAUCAAUGGGCACUGUAA | | | Cleavage | 1 |
|  |  |  | PtrmiR475a-3p | | | PtrPPR469 | | | Potri.013G149800.1 | | 3 | 16.309 | | 1 | | | 20 | 1197 | 1216 | | UUACAGUGCCCAUUGAUUAA | | | | CUGAUCAAUGGAUACUGUAA | | | Translation | 1 |
|  |  |  | PtrmiR475a-3p | | | PtrPPR474 | | | Potri.014G040200.1 | | 2.5 | 17.875 | | 1 | | | 20 | 2643 | 2662 | | UUACAGUGCCCAUUGAUUAA | | | | UUGAUCGAUGGCUACUGUAA | | | Translation | 2 |
|  |  |  | PtrmiR475a-3p | | | PtrPPR474 | | | Potri.014G040200.1 | | 3 | 14.189 | | 1 | | | 21 | 1277 | 1297 | | UUACAGUGCCCAUUGAUUAAG | | | | UUUGAUUAACGGGUAUUGUAA | | | Cleavage | 2 |
|  |  |  | PtrmiR475a-3p | | | PtrPPR477 | | | Potri.014G052800.1 | | 3 | 12.509 | | 1 | | | 21 | 2160 | 2180 | | UUACAGUGCCCAUUGAUUAAG | | | | UUUGAUUGAUGGAUACUGUAA | | | Translation | 1 |
|  |  |  | PtrmiR475a-3p | | | PtrPPR478 | | | Potri.014G056400.1 | | 1.5 | 16.774 | | 1 | | | 21 | 1543 | 1563 | | UUACAGUGCCCAUUGAUUAAG | | | | CUUGAUCAUUGGGCACUGUAA | | | Cleavage | 1 |
|  |  |  | PtrmiR475a-3p | | | PtrPPR486 | | | Potri.014G117600.1 | | 3 | 11.853 | | 1 | | | 21 | 726 | 746 | | UUACAGUGCCCAUUGAUUAAG | | | | UUUGAUGAAUGGAUACUGUAA | | | Translation | 1 |
|  |  |  | PtrmiR475a-3p | | | PtrPPR519 | | | Potri.016G025600.1 | | 2 | 16.825 | | 1 | | | 21 | 956 | 976 | | UUACAGUGCCCAUUGAUUAAG | | | | CUUAAUCAAUGGAUGCUGUAA | | | Translation | 1 |
|  |  |  | PtrmiR475a-3p | | | PtrPPR520 | | | Potri.016G026400.1 | | 2 | 16.774 | | 1 | | | 21 | 465 | 485 | | UUACAGUGCCCAUUGAUUAAG | | | | CUUAAUCAAUGGAUGCUGUAA | | | Translation | 1 |
|  |  |  | PtrmiR475a-3p | | | PtrPPR544 | | | Potri.017G032100.1 | | 2 | 16.925 | | 1 | | | 21 | 1101 | 1121 | | UUACAGUGCCCAUUGAUUAAG | | | | CUUAAUCAAUGGAUAUUGUAA | | | Translation | 1 |
|  |  |  | PtrmiR475a-3p | | | PtrPPR567 | | | Potri.017G131400.1 | | 3 | 11.129 | | 1 | | | 21 | 816 | 836 | | UUACAGUGCCCAUUGAUUAAG | | | | UUUGAUUAAUGGUUAUUGUAA | | | Translation | 1 |
|  |  |  | PtrmiR475a-3p | | | PtrPPR584 | | | Potri.018G038300.1 | | 2.5 | 23.926 | | 1 | | | 21 | 1276 | 1296 | | UUACAGUGCCCAUUGAUUAAG | | | | CUUAAUCAAUGGAUGUUGUAA | | | Translation | 1 |
|  |  |  | PtrmiR475a-3p | | | PtrPPR595 | | | Potri.018G143800.1 | | 3 | 16.422 | | 1 | | | 20 | 1213 | 1232 | | UUACAGUGCCCAUUGAUUAA | | | | UUGAUCGAUGGAUAUUGUAA | | | Translation | 1 |
|  |  |  | PtrmiR475a-3p | | | PtrPPR602 | | | Potri.019G021200.1 | | 1.5 | 15.146 | | 1 | | | 21 | 628 | 648 | | UUACAGUGCCCAUUGAUUAAG | | | | CUUAAUCAAUGGGCUCUGUAA | | | Cleavage | 1 |
|  |  |  | PtrmiR475a-3p | | | PtrPPR607 | | | Potri.019G062300.1 | | 1.5 | 12.774 | | 1 | | | 21 | 150 | 170 | | UUACAGUGCCCAUUGAUUAAG | | | | CUUAAUCAAUGGGCUCUGUAA | | | Cleavage | 2 |
|  |  |  | PtrmiR475a-3p | | | PtrPPR607 | | | Potri.019G062300.1 | | 2.5 | 11.065 | | 1 | | | 21 | 585 | 605 | | UUACAGUGCCCAUUGAUUAAG | | | | CUUAAUUAAUGGAUAUUGUAA | | | Translation | 2 |
|  |  |  | PtrmiR475a-3p | | | PtrPPR617 | | | Potri.T120800.1 | | 3 | 21.237 | | 1 | | | 21 | 535 | 555 | | UUACAGUGCCCAUUGAUUAAG | | | | CUUAAUCAAUGGGCUCGGUAA | | | Cleavage | 1 |
|  |  |  | PtrmiR475b-3p | | | PtrPPR49 | | | Potri.001G368700.1 | | 2.5 | 11.927 | | 1 | | | 21 | 1128 | 1148 | | UUACAGUGCCCAUUGAUUAAG | | | | UUUGAUUGAUGGGCAUUGUGA | | | Cleavage | 1 |
|  |  |  | PtrmiR475b-3p | | | PtrPPR120 | | | Potri.003G105700.1 | | 2 | 8.505 | | 1 | | | 20 | 1117 | 1136 | | UUACAGUGCCCAUUGAUUAA | | | | UUGAUUGAUGGGUACUGUAA | | | Cleavage | 1 |
|  |  |  | PtrmiR475b-3p | | | PtrPPR142 | | | Potri.004G066600.1 | | 2 | 18.029 | | 1 | | | 21 | 2208 | 2228 | | UUACAGUGCCCAUUGAUUAAG | | | | UUUGAUUGAUGGGCAUUGUAA | | | Cleavage | 1 |
|  |  |  | PtrmiR475b-3p | | | PtrPPR145 | | | Potri.004G074500.1 | | 2.5 | 19 | | 1 | | | 21 | 668 | 688 | | UUACAGUGCCCAUUGAUUAAG | | | | CUUAACCAAUGGGCUCUGUAA | | | Cleavage | 1 |
|  |  |  | PtrmiR475b-3p | | | PtrPPR146 | | | Potri.004G074700.1 | | 3 | 20.552 | | 1 | | | 21 | 423 | 443 | | UUACAGUGCCCAUUGAUUAAG | | | | CUUAAUCAAUGGGCUCGGUAA | | | Cleavage | 1 |
|  |  |  | PtrmiR475b-3p | | | PtrPPR274 | | | Potri.006G223300.1 | | 3 | 16.134 | | 1 | | | 21 | 705 | 725 | | UUACAGUGCCCAUUGAUUAAG | | | | CUUGAUUAAUGGUUAUUGUAA | | | Translation | 1 |
|  |  |  | PtrmiR475b-3p | | | PtrPPR276 | | | Potri.006G242200.1 | | 1.5 | 12.735 | | 1 | | | 21 | 510 | 530 | | UUACAGUGCCCAUUGAUUAAG | | | | CUUAAUCAAUGGGCUCUGUAA | | | Cleavage | 1 |
|  |  |  | PtrmiR475b-3p | | | PtrPPR277 | | | Potri.006G242500.1 | | 2.5 | 14.973 | | 1 | | | 21 | 652 | 672 | | UUACAGUGCCCAUUGAUUAAG | | | | CUUAAUCAAUUGGCUCUGUAA | | | Translation | 2 |
|  |  |  | PtrmiR475b-3p | | | PtrPPR277 | | | Potri.006G242500.1 | | 2.5 | 11.886 | | 1 | | | 21 | 1282 | 1302 | | UUACAGUGCCCAUUGAUUAAG | | | | CUUAAUUAAUGGAUAUUGUAA | | | Translation | 2 |
|  |  |  | PtrmiR475b-3p | | | PtrPPR284 | | | Potri.006G257300.1 | | 1.5 | 16.223 | | 1 | | | 21 | 779 | 799 | | UUACAGUGCCCAUUGAUUAAG | | | | CUUAAUCAAUGGGCUCUGUAA | | | Cleavage | 2 |
|  |  |  | PtrmiR475b-3p | | | PtrPPR284 | | | Potri.006G257300.1 | | 2.5 | 12.947 | | 1 | | | 21 | 1409 | 1429 | | UUACAGUGCCCAUUGAUUAAG | | | | CUUAAUUAAUGGAUAUUGUAA | | | Translation | 2 |
|  |  |  | PtrmiR475b-3p | | | PtrPPR287 | | | Potri.006G271200.1 | | 1.5 | 16.349 | | 1 | | | 21 | 389 | 409 | | UUACAGUGCCCAUUGAUUAAG | | | | CUUAAUCAAUGGGCUCUGUAA | | | Cleavage | 1 |
|  |  |  | PtrmiR475b-3p | | | PtrPPR288 | | | Potri.006G271400.1 | | 1.5 | 17.779 | | 1 | | | 21 | 504 | 524 | | UUACAGUGCCCAUUGAUUAAG | | | | CUUAAUCAAUGGGCUCUGUAA | | | Cleavage | 2 |
|  |  |  | PtrmiR475b-3p | | | PtrPPR288 | | | Potri.006G271400.1 | | 2.5 | 12.912 | | 1 | | | 21 | 1134 | 1154 | | UUACAGUGCCCAUUGAUUAAG | | | | CUUAAUUAAUGGAUAUUGUAA | | | Translation | 2 |
|  |  |  | PtrmiR475b-3p | | | PtrPPR297 | | | Potri.007G068400.1 | | 3 | 15.233 | | 1 | | | 21 | 674 | 694 | | UUACAGUGCCCAUUGAUUAAG | | | | CUUCAUCAAUGGGUACUGCAA | | | Cleavage | 1 |
|  |  |  | PtrmiR475b-3p | | | PtrPPR312 | | | Potri.008G044000.1 | | 2.5 | 20.085 | | 1 | | | 21 | 240 | 260 | | UUACAGUGCCCAUUGAUUAAG | | | | UUUGAUAAGUGGGCAUUGUAA | | | Cleavage | 1 |
|  |  |  | PtrmiR475b-3p | | | PtrPPR352 | | | Potri.009G105600.1 | | 3 | 13.222 | | 1 | | | 21 | 1569 | 1589 | | UUACAGUGCCCAUUGAUUAAG | | | | CUUGAUUAAUGGUUAUUGUAA | | | Translation | 1 |
|  |  |  | PtrmiR475b-3p | | | PtrPPR366 | | | Potri.010G035700.1 | | 3 | 12.456 | | 1 | | | 21 | 2038 | 2058 | | UUACAGUGCCCAUUGAUUAAG | | | | UUUGAUAAAAGGGCAUUGUAA | | | Cleavage | 1 |
|  |  |  | PtrmiR475b-3p | | | PtrPPR394 | | | Potri.010G234500.1 | | 3 | 14.611 | | 1 | | | 20 | 1465 | 1484 | | UUACAGUGCCCAUUGAUUAA | | | | UUGAUUGAUGGCUACUGUAA | | | Translation | 1 |
|  |  |  | PtrmiR475b-3p | | | PtrPPR400 | | | Potri.011G003700.1 | | 2.5 | 14.923 | | 1 | | | 21 | 1473 | 1493 | | UUACAGUGCCCAUUGAUUAAG | | | | UUUGAUCGAUGGGCACUGCAA | | | Cleavage | 1 |
|  |  |  | PtrmiR475b-3p | | | PtrPPR406 | | | Potri.011G057900.1 | | 2 | 14.06 | | 1 | | | 20 | 1049 | 1068 | | UUACAGUGCCCAUUGAUUAA | | | | UUAAUUAAUGGGCUCUGUAA | | | Cleavage | 1 |
|  |  |  | PtrmiR475b-3p | | | PtrPPR441 | | | Potri.013G032600.1 | | 1.5 | 20.164 | | 1 | | | 20 | 1069 | 1088 | | UUACAGUGCCCAUUGAUUAA | | | | UUGAUCAACGGGCACUGUAA | | | Cleavage | 1 |
|  |  |  | PtrmiR475b-3p | | | PtrPPR442 | | | Potri.013G034200.1 | | 1.5 | 17.057 | | 1 | | | 20 | 1186 | 1205 | | UUACAGUGCCCAUUGAUUAA | | | | UUGAUCAAGGGGCACUGUAA | | | Cleavage | 1 |
|  |  |  | PtrmiR475b-3p | | | PtrPPR443 | | | Potri.013G034300.1 | | 1.5 | 15.858 | | 1 | | | 20 | 676 | 695 | | UUACAGUGCCCAUUGAUUAA | | | | UUGAUCAAGGGGCACUGUAA | | | Cleavage | 1 |
|  |  |  | PtrmiR475b-3p | | | PtrPPR444 | | | Potri.013G034400.1 | | 1 | 17.242 | | 1 | | | 20 | 1130 | 1149 | | UUACAGUGCCCAUUGAUUAA | | | | CUAAUCAAUGGGCACUGUAA | | | Cleavage | 1 |
|  |  |  | PtrmiR475b-3p | | | PtrPPR469 | | | Potri.013G149800.1 | | 3 | 16.309 | | 1 | | | 20 | 1197 | 1216 | | UUACAGUGCCCAUUGAUUAA | | | | CUGAUCAAUGGAUACUGUAA | | | Translation | 1 |
|  |  |  | PtrmiR475b-3p | | | PtrPPR474 | | | Potri.014G040200.1 | | 2.5 | 17.875 | | 1 | | | 20 | 2643 | 2662 | | UUACAGUGCCCAUUGAUUAA | | | | UUGAUCGAUGGCUACUGUAA | | | Translation | 2 |
|  |  |  | PtrmiR475b-3p | | | PtrPPR474 | | | Potri.014G040200.1 | | 3 | 14.189 | | 1 | | | 21 | 1277 | 1297 | | UUACAGUGCCCAUUGAUUAAG | | | | UUUGAUUAACGGGUAUUGUAA | | | Cleavage | 2 |
|  |  |  | PtrmiR475b-3p | | | PtrPPR477 | | | Potri.014G052800.1 | | 3 | 12.509 | | 1 | | | 21 | 2160 | 2180 | | UUACAGUGCCCAUUGAUUAAG | | | | UUUGAUUGAUGGAUACUGUAA | | | Translation | 1 |
|  |  |  | PtrmiR475b-3p | | | PtrPPR478 | | | Potri.014G056400.1 | | 1.5 | 16.774 | | 1 | | | 21 | 1543 | 1563 | | UUACAGUGCCCAUUGAUUAAG | | | | CUUGAUCAUUGGGCACUGUAA | | | Cleavage | 1 |
|  |  |  | PtrmiR475b-3p | | | PtrPPR486 | | | Potri.014G117600.1 | | 3 | 11.853 | | 1 | | | 21 | 726 | 746 | | UUACAGUGCCCAUUGAUUAAG | | | | UUUGAUGAAUGGAUACUGUAA | | | Translation | 1 |
|  |  |  | PtrmiR475b-3p | | | PtrPPR519 | | | Potri.016G025600.1 | | 2 | 16.825 | | 1 | | | 21 | 956 | 976 | | UUACAGUGCCCAUUGAUUAAG | | | | CUUAAUCAAUGGAUGCUGUAA | | | Translation | 1 |
|  |  |  | PtrmiR475b-3p | | | PtrPPR520 | | | Potri.016G026400.1 | | 2 | 16.774 | | 1 | | | 21 | 465 | 485 | | UUACAGUGCCCAUUGAUUAAG | | | | CUUAAUCAAUGGAUGCUGUAA | | | Translation | 1 |
|  |  |  | PtrmiR475b-3p | | | PtrPPR544 | | | Potri.017G032100.1 | | 2 | 16.925 | | 1 | | | 21 | 1101 | 1121 | | UUACAGUGCCCAUUGAUUAAG | | | | CUUAAUCAAUGGAUAUUGUAA | | | Translation | 1 |
|  |  |  | PtrmiR475b-3p | | | PtrPPR567 | | | Potri.017G131400.1 | | 3 | 11.129 | | 1 | | | 21 | 816 | 836 | | UUACAGUGCCCAUUGAUUAAG | | | | UUUGAUUAAUGGUUAUUGUAA | | | Translation | 1 |
|  |  |  | PtrmiR475b-3p | | | PtrPPR584 | | | Potri.018G038300.1 | | 2.5 | 23.926 | | 1 | | | 21 | 1276 | 1296 | | UUACAGUGCCCAUUGAUUAAG | | | | CUUAAUCAAUGGAUGUUGUAA | | | Translation | 1 |
|  |  |  | PtrmiR475b-3p | | | PtrPPR595 | | | Potri.018G143800.1 | | 3 | 16.422 | | 1 | | | 20 | 1213 | 1232 | | UUACAGUGCCCAUUGAUUAA | | | | UUGAUCGAUGGAUAUUGUAA | | | Translation | 1 |
|  |  |  | PtrmiR475b-3p | | | PtrPPR602 | | | Potri.019G021200.1 | | 1.5 | 15.146 | | 1 | | | 21 | 628 | 648 | | UUACAGUGCCCAUUGAUUAAG | | | | CUUAAUCAAUGGGCUCUGUAA | | | Cleavage | 1 |
|  |  |  | PtrmiR475b-3p | | | PtrPPR607 | | | Potri.019G062300.1 | | 1.5 | 12.774 | | 1 | | | 21 | 150 | 170 | | UUACAGUGCCCAUUGAUUAAG | | | | CUUAAUCAAUGGGCUCUGUAA | | | Cleavage | 2 |
|  |  |  | PtrmiR475b-3p | | | PtrPPR607 | | | Potri.019G062300.1 | | 2.5 | 11.065 | | 1 | | | 21 | 585 | 605 | | UUACAGUGCCCAUUGAUUAAG | | | | CUUAAUUAAUGGAUAUUGUAA | | | Translation | 2 |
|  |  |  | PtrmiR475b-3p | | | PtrPPR617 | | | Potri.T120800.1 | | 3 | 21.237 | | 1 | | | 21 | 535 | 555 | | UUACAGUGCCCAUUGAUUAAG | | | | CUUAAUCAAUGGGCUCGGUAA | | | Cleavage | 1 |
|  |  |  | PtrmiR475c | | | PtrPPR49 | | | Potri.001G368700.1 | | 2.5 | 11.927 | | 1 | | | 21 | 1128 | 1148 | | UUACAAUGUCCAUUGAUUAAG | | | | UUUGAUUGAUGGGCAUUGUGA | | | Cleavage | 1 |
|  |  |  | PtrmiR475c | | | PtrPPR70 | | | Potri.002G080300.1 | | 2.5 | 12.285 | | 1 | | | 21 | 1141 | 1161 | | UUACAAUGUCCAUUGAUUAAG | | | | UUUGAUAAAUGGAUGUUGUAA | | | Cleavage | 1 |
|  |  |  | PtrmiR475c | | | PtrPPR142 | | | Potri.004G066600.1 | | 2 | 18.029 | | 1 | | | 21 | 2208 | 2228 | | UUACAAUGUCCAUUGAUUAAG | | | | UUUGAUUGAUGGGCAUUGUAA | | | Cleavage | 1 |
|  |  |  | PtrmiR475c | | | PtrPPR145 | | | Potri.004G074500.1 | | 2 | 15.076 | | 1 | | | 21 | 1298 | 1318 | | UUACAAUGUCCAUUGAUUAAG | | | | CUUAAUUAACGGAUAUUGUAA | | | Cleavage | 1 |
|  |  |  | PtrmiR475c | | | PtrPPR146 | | | Potri.004G074700.1 | | 2 | 14.864 | | 1 | | | 21 | 1053 | 1073 | | UUACAAUGUCCAUUGAUUAAG | | | | CUUAAUUAACGGAUAUUGUAA | | | Cleavage | 1 |
|  |  |  | PtrmiR475c | | | PtrPPR181 | | | Potri.005G014500.1 | | 3 | 13.675 | | 1 | | | 21 | 2046 | 2066 | | UUACAAUGUCCAUUGAUUAAG | | | | UUUGAUGGAUGGAUAUUGUAG | | | Cleavage | 1 |
|  |  |  | PtrmiR475c | | | PtrPPR182 | | | Potri.005G017500.1 | | 3 | 13.212 | | 1 | | | 21 | 1896 | 1916 | | UUACAAUGUCCAUUGAUUAAG | | | | UUUGAUGGAUGGAUAUUGUAG | | | Cleavage | 1 |
|  |  |  | PtrmiR475c | | | PtrPPR185 | | | Potri.005G038400.1 | | 2.5 | 14.385 | | 1 | | | 21 | 1156 | 1176 | | UUACAAUGUCCAUUGAUUAAG | | | | CUUGAUCAAUGGAUAUUGCAA | | | Cleavage | 1 |
|  |  |  | PtrmiR475c | | | PtrPPR186 | | | Potri.005G038500.1 | | 2.5 | 16.909 | | 1 | | | 21 | 171 | 191 | | UUACAAUGUCCAUUGAUUAAG | | | | CUUGAUCAAUGGAUAUUGCAA | | | Cleavage | 1 |
|  |  |  | PtrmiR475c | | | PtrPPR187 | | | Potri.005G045000.1 | | 2.5 | 12.764 | | 1 | | | 21 | 1143 | 1163 | | UUACAAUGUCCAUUGAUUAAG | | | | CUUGAUCAAUGGAUAUUGCAA | | | Cleavage | 1 |
|  |  |  | PtrmiR475c | | | PtrPPR188 | | | Potri.005G046000.1 | | 2.5 | 14.241 | | 1 | | | 21 | 1836 | 1856 | | UUACAAUGUCCAUUGAUUAAG | | | | CUUGAUCAAUGGAUAUUGCAA | | | Cleavage | 1 |
|  |  |  | PtrmiR475c | | | PtrPPR189 | | | Potri.005G046100.1 | | 2.5 | 15.753 | | 1 | | | 21 | 936 | 956 | | UUACAAUGUCCAUUGAUUAAG | | | | CUUGAUCAAUGGAUAUUGCAA | | | Cleavage | 1 |
|  |  |  | PtrmiR475c | | | PtrPPR190 | | | Potri.005G046200.1 | | 2.5 | 15.029 | | 1 | | | 21 | 1131 | 1151 | | UUACAAUGUCCAUUGAUUAAG | | | | CUUGAUCAAUGGAUAUUGCAA | | | Cleavage | 1 |
|  |  |  | PtrmiR475c | | | PtrPPR194 | | | Potri.005G050100.1 | | 2.5 | 16.862 | | 1 | | | 21 | 936 | 956 | | UUACAAUGUCCAUUGAUUAAG | | | | CUUGAUCAAUGGAUAUUGCAA | | | Cleavage | 1 |
|  |  |  | PtrmiR475c | | | PtrPPR195 | | | Potri.005G050200.1 | | 2.5 | 18.906 | | 1 | | | 21 | 975 | 995 | | UUACAAUGUCCAUUGAUUAAG | | | | CUUGAUCAAUGGAUUUUGUAA | | | Cleavage | 1 |
|  |  |  | PtrmiR475c | | | PtrPPR195 | | | Potri.005G050200.1 | | 2.5 | 18.906 | | 1 | | | 21 | 975 | 995 | | UUACAAUGUCCAUUGAUUAAG | | | | CUUGAUCAAUGGAUUUUGUAA | | | Cleavage | 1 |
|  |  |  | PtrmiR475c | | | PtrPPR198 | | | Potri.005G050500.1 | | 2.5 | 13.257 | | 1 | | | 21 | 1224 | 1244 | | UUACAAUGUCCAUUGAUUAAG | | | | CUUGAUCAAUGGAUAUUGCAA | | | Cleavage | 1 |
|  |  |  | PtrmiR475c | | | PtrPPR205 | | | Potri.005G097200.1 | | 2.5 | 14.947 | | 1 | | | 21 | 627 | 647 | | UUACAAUGUCCAUUGAUUAAG | | | | CUUGAUCUGUGGAUAUUGUAA | | | Cleavage | 1 |
|  |  |  | PtrmiR475c | | | PtrPPR256 | | | Potri.006G124900.1 | | 3 | 13.661 | | 1 | | | 20 | 1717 | 1736 | | UUACAAUGUCCAUUGAUUAA | | | | UUGAUUGAUGGACAUUGCAA | | | Cleavage | 1 |
|  |  |  | PtrmiR475c | | | PtrPPR274 | | | Potri.006G223300.1 | | 2.5 | 16.134 | | 1 | | | 21 | 705 | 725 | | UUACAAUGUCCAUUGAUUAAG | | | | CUUGAUUAAUGGUUAUUGUAA | | | Translation | 1 |
|  |  |  | PtrmiR475c | | | PtrPPR277 | | | Potri.006G242500.1 | | 1 | 11.886 | | 1 | | | 21 | 1282 | 1302 | | UUACAAUGUCCAUUGAUUAAG | | | | CUUAAUUAAUGGAUAUUGUAA | | | Cleavage | 1 |
|  |  |  | PtrmiR475c | | | PtrPPR284 | | | Potri.006G257300.1 | | 1 | 12.947 | | 1 | | | 21 | 1409 | 1429 | | UUACAAUGUCCAUUGAUUAAG | | | | CUUAAUUAAUGGAUAUUGUAA | | | Cleavage | 1 |
|  |  |  | PtrmiR475c | | | PtrPPR288 | | | Potri.006G271400.1 | | 1 | 12.912 | | 1 | | | 21 | 1134 | 1154 | | UUACAAUGUCCAUUGAUUAAG | | | | CUUAAUUAAUGGAUAUUGUAA | | | Cleavage | 1 |
|  |  |  | PtrmiR475c | | | PtrPPR312 | | | Potri.008G044000.1 | | 2.5 | 20.085 | | 1 | | | 21 | 240 | 260 | | UUACAAUGUCCAUUGAUUAAG | | | | UUUGAUAAGUGGGCAUUGUAA | | | Cleavage | 1 |
|  |  |  | PtrmiR475c | | | PtrPPR352 | | | Potri.009G105600.1 | | 2.5 | 13.222 | | 1 | | | 21 | 1569 | 1589 | | UUACAAUGUCCAUUGAUUAAG | | | | CUUGAUUAAUGGUUAUUGUAA | | | Translation | 1 |
|  |  |  | PtrmiR475c | | | PtrPPR366 | | | Potri.010G035700.1 | | 3 | 12.456 | | 1 | | | 21 | 2038 | 2058 | | UUACAAUGUCCAUUGAUUAAG | | | | UUUGAUAAAAGGGCAUUGUAA | | | Cleavage | 1 |
|  |  |  | PtrmiR475c | | | PtrPPR394 | | | Potri.010G234500.1 | | 3 | 11.277 | | 1 | | | 21 | 1044 | 1064 | | UUACAAUGUCCAUUGAUUAAG | | | | UUUAAUUGAUGGAUAUUGCAA | | | Cleavage | 1 |
|  |  |  | PtrmiR475c | | | PtrPPR408 | | | Potri.011G082300.1 | | 2.5 | 14.859 | | 1 | | | 21 | 579 | 599 | | UUACAAUGUCCAUUGAUUAAG | | | | UUUGAUCAAGGGAUAUUGUGA | | | Cleavage | 1 |
|  |  |  | PtrmiR475c | | | PtrPPR444 | | | Potri.013G034400.1 | | 3 | 17.242 | | 1 | | | 20 | 1130 | 1149 | | UUACAAUGUCCAUUGAUUAA | | | | CUAAUCAAUGGGCACUGUAA | | | Cleavage | 1 |
|  |  |  | PtrmiR475c | | | PtrPPR462 | | | Potri.013G130600.1 | | 2.5 | 12.105 | | 1 | | | 21 | 747 | 767 | | UUACAAUGUCCAUUGAUUAAG | | | | CUUGCUUAAUGGAUAUUGUAA | | | Cleavage | 1 |
|  |  |  | PtrmiR475c | | | PtrPPR474 | | | Potri.014G040200.1 | | 3 | 14.189 | | 1 | | | 21 | 1277 | 1297 | | UUACAAUGUCCAUUGAUUAAG | | | | UUUGAUUAACGGGUAUUGUAA | | | Cleavage | 2 |
|  |  |  | PtrmiR475c | | | PtrPPR474 | | | Potri.014G040200.1 | | 3 | 14.568 | | 1 | | | 20 | 1065 | 1084 | | UUACAAUGUCCAUUGAUUAA | | | | UUGAUCAAGGGUUAUUGUAA | | | Translation | 2 |
|  |  |  | PtrmiR475c | | | PtrPPR478 | | | Potri.014G090400.1 | | 3 | 13.051 | | 1 | | | 20 | 1165 | 1184 | | UUACAAUGUCCAUUGAUUAA | | | | UUGAUUGAUGGACUUUGUAA | | | Cleavage | 1 |
|  |  |  | PtrmiR475c | | | PtrPPR519 | | | Potri.016G025600.1 | | 2 | 19.392 | | 1 | | | 21 | 1166 | 1186 | | UUACAAUGUCCAUUGAUUAAG | | | | CUUAAUCAAUGGAUCUUGUAA | | | Cleavage | 2 |
|  |  |  | PtrmiR475c | | | PtrPPR519 | | | Potri.016G025600.1 | | 2.5 | 16.825 | | 1 | | | 21 | 956 | 976 | | UUACAAUGUCCAUUGAUUAAG | | | | CUUAAUCAAUGGAUGCUGUAA | | | Cleavage | 2 |
|  |  |  | PtrmiR475c | | | PtrPPR520 | | | Potri.016G026400.1 | | 2.5 | 16.774 | | 1 | | | 21 | 465 | 485 | | UUACAAUGUCCAUUGAUUAAG | | | | CUUAAUCAAUGGAUGCUGUAA | | | Cleavage | 1 |
|  |  |  | PtrmiR475c | | | PtrPPR544 | | | Potri.017G032100.1 | | 0.5 | 16.925 | | 1 | | | 21 | 1101 | 1121 | | UUACAAUGUCCAUUGAUUAAG | | | | CUUAAUCAAUGGAUAUUGUAA | | | Cleavage | 1 |
|  |  |  | PtrmiR475c | | | PtrPPR567 | | | Potri.017G131400.1 | | 2.5 | 11.129 | | 1 | | | 21 | 816 | 836 | | UUACAAUGUCCAUUGAUUAAG | | | | UUUGAUUAAUGGUUAUUGUAA | | | Translation | 1 |
|  |  |  | PtrmiR475c | | | PtrPPR584 | | | Potri.018G038300.1 | | 1 | 23.926 | | 1 | | | 21 | 1276 | 1296 | | UUACAAUGUCCAUUGAUUAAG | | | | CUUAAUCAAUGGAUGUUGUAA | | | Cleavage | 1 |
|  |  |  | PtrmiR475c | | | PtrPPR595 | | | Potri.018G143800.1 | | 1.5 | 16.422 | | 1 | | | 20 | 1213 | 1232 | | UUACAAUGUCCAUUGAUUAA | | | | UUGAUCGAUGGAUAUUGUAA | | | Cleavage | 1 |
|  |  |  | PtrmiR475c | | | PtrPPR602 | | | Potri.019G021200.1 | | 2 | 12.733 | | 1 | | | 21 | 1258 | 1278 | | UUACAAUGUCCAUUGAUUAAG | | | | CUUAAUUAAUGGAUAUUGUAU | | | Cleavage | 1 |
|  |  |  | PtrmiR475c | | | PtrPPR607 | | | Potri.019G062300.1 | | 1 | 11.065 | | 1 | | | 21 | 585 | 605 | | UUACAAUGUCCAUUGAUUAAG | | | | CUUAAUUAAUGGAUAUUGUAA | | | Cleavage | 1 |
|  |  |  | PtrmiR475c | | | PtrPPR617 | | | Potri.T120800.1 | | 2 | 14.864 | | 1 | | | 21 | 1165 | 1185 | | UUACAAUGUCCAUUGAUUAAG | | | | CUUAAUUAACGGAUAUUGUAA | | | Cleavage | 1 |
|  |  |  | PtrmiR475d-3p | | | PtrPPR20 | | | Potri.001G139200.1 | | 2.5 | 12.171 | | 1 | | | 20 | 866 | 885 | | UUACAGAGUCCAUUGAUUAA | | | | AUGAUUAAUGGGCUCUGUAA | | | Cleavage | 2 |
|  |  |  | PtrmiR475d-3p | | | PtrPPR20 | | | Potri.001G139200.1 | | 3 | 18.235 | | 1 | | | 21 | 1390 | 1410 | | UUACAGAGUCCAUUGAUUAAG | | | | UUUAAUGAAUGGACUUUGCAA | | | Cleavage | 2 |
|  |  |  | PtrmiR475d-3p | | | PtrPPR85 | | | Potri.002G192100.1 | | 3 | 14.316 | | 1 | | | 21 | 297 | 317 | | UUACAGAGUCCAUUGAUUAAG | | | | CUUAUUGAAUGGGCUUUGUAA | | | Cleavage | 1 |
|  |  |  | PtrmiR475d-3p | | | PtrPPR122 | | | Potri.003G154800.1 | | 3 | 14.307 | | 1 | | | 20 | 733 | 752 | | UUACAGAGUCCAUUGAUUAA | | | | UUAAUCAAUGGCUUGUGUAA | | | Translation | 1 |
|  |  |  | PtrmiR475d-3p | | | PtrPPR145 | | | Potri.004G074500.1 | | 1.5 | 19 | | 1 | | | 21 | 668 | 688 | | UUACAGAGUCCAUUGAUUAAG | | | | CUUAACCAAUGGGCUCUGUAA | | | Cleavage | 2 |
|  |  |  | PtrmiR475d-3p | | | PtrPPR145 | | | Potri.004G074500.1 | | 2.5 | 9.013 | | 1 | | | 20 | 1719 | 1738 | | UUACAGAGUCCAUUGAUUAA | | | | AUAAUAAAUGGACUUUGUAA | | | Cleavage | 2 |
|  |  |  | PtrmiR475d-3p | | | PtrPPR146 | | | Potri.004G074700.1 | | 2 | 20.552 | | 1 | | | 21 | 423 | 443 | | UUACAGAGUCCAUUGAUUAAG | | | | CUUAAUCAAUGGGCUCGGUAA | | | Cleavage | 2 |
|  |  |  | PtrmiR475d-3p | | | PtrPPR146 | | | Potri.004G074700.1 | | 2.5 | 10.317 | | 1 | | | 20 | 1474 | 1493 | | UUACAGAGUCCAUUGAUUAA | | | | AUAAUAAAUGGACUUUGUAA | | | Cleavage | 2 |
|  |  |  | PtrmiR475d-3p | | | PtrPPR195 | | | Potri.005G050200.1 | | 1.5 | 18.906 | | 1 | | | 21 | 975 | 995 | | UUACAGAGUCCAUUGAUUAAG | | | | CUUGAUCAAUGGAUUUUGUAA | | | Cleavage | 1 |
|  |  |  | PtrmiR475d-3p | | | PtrPPR195 | | | Potri.005G050200.1 | | 1.5 | 18.906 | | 1 | | | 21 | 975 | 995 | | UUACAGAGUCCAUUGAUUAAG | | | | CUUGAUCAAUGGAUUUUGUAA | | | Cleavage | 1 |
|  |  |  | PtrmiR475d-3p | | | PtrPPR197 | | | Potri.005G050400.1 | | 3 | 16.716 | | 1 | | | 21 | 1140 | 1160 | | UUACAGAGUCCAUUGAUUAAG | | | | CUUGAUCAAUGGAUUUUGCAA | | | Cleavage | 1 |
|  |  |  | PtrmiR475d-3p | | | PtrPPR276 | | | Potri.006G242200.1 | | 0.5 | 12.735 | | 1 | | | 21 | 510 | 530 | | UUACAGAGUCCAUUGAUUAAG | | | | CUUAAUCAAUGGGCUCUGUAA | | | Cleavage | 1 |
|  |  |  | PtrmiR475d-3p | | | PtrPPR277 | | | Potri.006G242500.1 | | 1.5 | 14.973 | | 1 | | | 21 | 652 | 672 | | UUACAGAGUCCAUUGAUUAAG | | | | CUUAAUCAAUUGGCUCUGUAA | | | Translation | 3 |
|  |  |  | PtrmiR475d-3p | | | PtrPPR277 | | | Potri.006G242500.1 | | 3 | 11.886 | | 1 | | | 21 | 1282 | 1302 | | UUACAGAGUCCAUUGAUUAAG | | | | CUUAAUUAAUGGAUAUUGUAA | | | Cleavage | 3 |
|  |  |  | PtrmiR475d-3p | | | PtrPPR277 | | | Potri.006G242500.1 | | 2.5 | 11.091 | | 1 | | | 20 | 1703 | 1722 | | UUACAGAGUCCAUUGAUUAA | | | | AUAAUAAAUGGACUUUGUAA | | | Cleavage | 3 |
|  |  |  | PtrmiR475d-3p | | | PtrPPR284 | | | Potri.006G257300.1 | | 0.5 | 16.223 | | 1 | | | 21 | 779 | 799 | | UUACAGAGUCCAUUGAUUAAG | | | | CUUAAUCAAUGGGCUCUGUAA | | | Cleavage | 3 |
|  |  |  | PtrmiR475d-3p | | | PtrPPR284 | | | Potri.006G257300.1 | | 3 | 12.947 | | 1 | | | 21 | 1409 | 1429 | | UUACAGAGUCCAUUGAUUAAG | | | | CUUAAUUAAUGGAUAUUGUAA | | | Cleavage | 3 |
|  |  |  | PtrmiR475d-3p | | | PtrPPR284 | | | Potri.006G257300.1 | | 2.5 | 9.769 | | 1 | | | 20 | 1830 | 1849 | | UUACAGAGUCCAUUGAUUAA | | | | AUAAUAAAUGGACUUUGUAA | | | Cleavage | 3 |
|  |  |  | PtrmiR475d-3p | | | PtrPPR287 | | | Potri.006G271200.1 | | 0.5 | 16.349 | | 1 | | | 21 | 389 | 409 | | UUACAGAGUCCAUUGAUUAAG | | | | CUUAAUCAAUGGGCUCUGUAA | | | Cleavage | 1 |
|  |  |  | PtrmiR475d-3p | | | PtrPPR288 | | | Potri.006G271400.1 | | 0.5 | 17.779 | | 1 | | | 21 | 504 | 524 | | UUACAGAGUCCAUUGAUUAAG | | | | CUUAAUCAAUGGGCUCUGUAA | | | Cleavage | 3 |
|  |  |  | PtrmiR475d-3p | | | PtrPPR288 | | | Potri.006G271400.1 | | 3 | 12.912 | | 1 | | | 21 | 1134 | 1154 | | UUACAGAGUCCAUUGAUUAAG | | | | CUUAAUUAAUGGAUAUUGUAA | | | Cleavage | 3 |
|  |  |  | PtrmiR475d-3p | | | PtrPPR288 | | | Potri.006G271400.1 | | 2.5 | 10.18 | | 1 | | | 20 | 1555 | 1574 | | UUACAGAGUCCAUUGAUUAA | | | | AUAAUAAAUGGACUUUGUAA | | | Cleavage | 3 |
|  |  |  | PtrmiR475d-3p | | | PtrPPR305 | | | Potri.007G123600.1 | | 3 | 16.855 | | 1 | | | 21 | 1905 | 1925 | | UUACAGAGUCCAUUGAUUAAG | | | | UUUAAUCGAUGGUCUAUGUAA | | | Translation | 1 |
|  |  |  | PtrmiR475d-3p | | | PtrPPR385 | | | Potri.010G148700.1 | | 3 | 14.448 | | 1 | | | 21 | 744 | 764 | | UUACAGAGUCCAUUGAUUAAG | | | | UUUGAUUAAUGGGUUUUGUAG | | | Cleavage | 1 |
|  |  |  | PtrmiR475d-3p | | | PtrPPR406 | | | Potri.011G057900.1 | | 1 | 14.06 | | 1 | | | 20 | 1049 | 1068 | | UUACAGAGUCCAUUGAUUAA | | | | UUAAUUAAUGGGCUCUGUAA | | | Cleavage | 1 |
|  |  |  | PtrmiR475d-3p | | | PtrPPR441 | | | Potri.013G032600.1 | | 2.5 | 14.214 | | 1 | | | 21 | 858 | 878 | | UUACAGAGUCCAUUGAUUAAG | | | | UUUAAUUGAUGGACUCUGCAA | | | Cleavage | 1 |
|  |  |  | PtrmiR475d-3p | | | PtrPPR444 | | | Potri.013G034400.1 | | 3 | 17.242 | | 1 | | | 20 | 1130 | 1149 | | UUACAGAGUCCAUUGAUUAA | | | | CUAAUCAAUGGGCACUGUAA | | | Cleavage | 1 |
|  |  |  | PtrmiR475d-3p | | | PtrPPR456 | | | Potri.013G121600.1 | | 3 | 15.428 | | 1 | | | 20 | 364 | 383 | | UUACAGAGUCCAUUGAUUAA | | | | GUAAUGAAUGGACUUUGUAG | | | Cleavage | 1 |
|  |  |  | PtrmiR475d-3p | | | PtrPPR462 | | | Potri.013G130600.1 | | 2.5 | 17.132 | | 1 | | | 21 | 432 | 452 | | UUACAGAGUCCAUUGAUUAAG | | | | CUUGGUAAAUGGGCUCUGUAA | | | Cleavage | 2 |
|  |  |  | PtrmiR475d-3p | | | PtrPPR462 | | | Potri.013G130600.1 | | 3 | 11.667 | | 1 | | | 20 | 1168 | 1187 | | UUACAGAGUCCAUUGAUUAA | | | | UUAAUUGGUGGAAUUUGUAA | | | Cleavage | 2 |
|  |  |  | PtrmiR475d-3p | | | PtrPPR469 | | | Potri.013G149800.1 | | 3 | 16.445 | | 1 | | | 21 | 566 | 586 | | UUACAGAGUCCAUUGAUUAAG | | | | CUUAGUUAAUGGGCUUUGUAU | | | Cleavage | 2 |
|  |  |  | PtrmiR475d-3p | | | PtrPPR469 | | | Potri.013G149800.1 | | 3 | 9.435 | | 1 | | | 20 | 1617 | 1636 | | UUACAGAGUCCAUUGAUUAA | | | | AUAAUCAAUGGACUUUGCAA | | | Cleavage | 2 |
|  |  |  | PtrmiR475d-3p | | | PtrPPR470 | | | Potri.013G150100.1 | | 3 | 11.44 | | 1 | | | 21 | 666 | 686 | | UUACAGAGUCCAUUGAUUAAG | | | | CUUAAUUAAUGGGUUUUGUAU | | | Cleavage | 1 |
|  |  |  | PtrmiR475d-3p | | | PtrPPR474 | | | Potri.014G040200.1 | | 3 | 11.523 | | 1 | | | 21 | 2747 | 2767 | | UUACAGAGUCCAUUGAUUAAG | | | | CUUGAUUAAUGGUUUUUGUAA | | | Translation | 1 |
|  |  |  | PtrmiR475d-3p | | | PtrPPR478 | | | Potri.014G090400.1 | | 2.5 | 14.17 | | 1 | | | 21 | 1272 | 1292 | | UUACAGAGUCCAUUGAUUAAG | | | | CUUGAUCGAUGGGUUUUGUAA | | | Cleavage | 3 |
|  |  |  | PtrmiR475d-3p | | | PtrPPR478 | | | Potri.014G090400.1 | | 2 | 13.051 | | 1 | | | 20 | 1165 | 1184 | | UUACAGAGUCCAUUGAUUAA | | | | UUGAUUGAUGGACUUUGUAA | | | Cleavage | 3 |
|  |  |  | PtrmiR475d-3p | | | PtrPPR478 | | | Potri.014G090400.1 | | 3 | 19.513 | | 1 | | | 21 | 1482 | 1502 | | UUACAGAGUCCAUUGAUUAAG | | | | UUUGAUCAAUGCUUUCUGUAA | | | Translation | 3 |
|  |  |  | PtrmiR475d-3p | | | PtrPPR497 | | | Potri.015G036400.1 | | 3 | 11.568 | | 1 | | | 20 | 2244 | 2263 | | UUACAGAGUCCAUUGAUUAA | | | | CUGAUUGAUGGACUUUGUAA | | | Cleavage | 1 |
|  |  |  | PtrmiR475d-3p | | | PtrPPR506 | | | Potri.015G105400.1 | | 2.5 | 12.303 | | 1 | | | 21 | 2168 | 2188 | | UUACAGAGUCCAUUGAUUAAG | | | | UUUGAUCAAUGGACUUUGCAA | | | Cleavage | 1 |
|  |  |  | PtrmiR475d-3p | | | PtrPPR519 | | | Potri.016G025600.1 | | 2 | 16.825 | | 1 | | | 21 | 956 | 976 | | UUACAGAGUCCAUUGAUUAAG | | | | CUUAAUCAAUGGAUGCUGUAA | | | Cleavage | 2 |
|  |  |  | PtrmiR475d-3p | | | PtrPPR519 | | | Potri.016G025600.1 | | 2.5 | 19.392 | | 1 | | | 21 | 1166 | 1186 | | UUACAGAGUCCAUUGAUUAAG | | | | CUUAAUCAAUGGAUCUUGUAA | | | Cleavage | 2 |
|  |  |  | PtrmiR475d-3p | | | PtrPPR520 | | | Potri.016G026400.1 | | 2 | 16.774 | | 1 | | | 21 | 465 | 485 | | UUACAGAGUCCAUUGAUUAAG | | | | CUUAAUCAAUGGAUGCUGUAA | | | Cleavage | 1 |
|  |  |  | PtrmiR475d-3p | | | PtrPPR544 | | | Potri.017G032100.1 | | 2.5 | 16.925 | | 1 | | | 21 | 1101 | 1121 | | UUACAGAGUCCAUUGAUUAAG | | | | CUUAAUCAAUGGAUAUUGUAA | | | Cleavage | 1 |
|  |  |  | PtrmiR475d-3p | | | PtrPPR553 | | | Potri.017G071600.1 | | 2.5 | 21.447 | | 1 | | | 20 | 982 | 1001 | | UUACAGAGUCCAUUGAUUAA | | | | UUAAUGAAUGGCCUUUGUAA | | | Translation | 1 |
|  |  |  | PtrmiR475d-3p | | | PtrPPR584 | | | Potri.018G038300.1 | | 2.5 | 23.926 | | 1 | | | 21 | 1276 | 1296 | | UUACAGAGUCCAUUGAUUAAG | | | | CUUAAUCAAUGGAUGUUGUAA | | | Cleavage | 2 |
|  |  |  | PtrmiR475d-3p | | | PtrPPR584 | | | Potri.018G038300.1 | | 3 | 15.996 | | 1 | | | 21 | 646 | 666 | | UUACAGAGUCCAUUGAUUAAG | | | | UUUAAUUAAUGGCCUUUGUAU | | | Translation | 2 |
|  |  |  | PtrmiR475d-3p | | | PtrPPR602 | | | Potri.019G021200.1 | | 0.5 | 15.146 | | 1 | | | 21 | 628 | 648 | | UUACAGAGUCCAUUGAUUAAG | | | | CUUAAUCAAUGGGCUCUGUAA | | | Cleavage | 2 |
|  |  |  | PtrmiR475d-3p | | | PtrPPR602 | | | Potri.019G021200.1 | | 2.5 | 11.736 | | 1 | | | 20 | 1679 | 1698 | | UUACAGAGUCCAUUGAUUAA | | | | AUAAUAAAUGGACUUUGUAA | | | Cleavage | 2 |
|  |  |  | PtrmiR475d-3p | | | PtrPPR607 | | | Potri.019G062300.1 | | 0.5 | 12.774 | | 1 | | | 21 | 150 | 170 | | UUACAGAGUCCAUUGAUUAAG | | | | CUUAAUCAAUGGGCUCUGUAA | | | Cleavage | 3 |
|  |  |  | PtrmiR475d-3p | | | PtrPPR607 | | | Potri.019G062300.1 | | 3 | 11.065 | | 1 | | | 21 | 585 | 605 | | UUACAGAGUCCAUUGAUUAAG | | | | CUUAAUUAAUGGAUAUUGUAA | | | Cleavage | 3 |
|  |  |  | PtrmiR475d-3p | | | PtrPPR607 | | | Potri.019G062300.1 | | 2.5 | 9.753 | | 1 | | | 20 | 868 | 887 | | UUACAGAGUCCAUUGAUUAA | | | | AUAAUAAAUGGACUUUGUAA | | | Cleavage | 3 |
|  |  |  | PtrmiR475d-3p | | | PtrPPR617 | | | Potri.T120800.1 | | 2 | 21.237 | | 1 | | | 21 | 535 | 555 | | UUACAGAGUCCAUUGAUUAAG | | | | CUUAAUCAAUGGGCUCGGUAA | | | Cleavage | 2 |
|  |  |  | PtrmiR475d-3p | | | PtrPPR617 | | | Potri.T120800.1 | | 2.5 | 10.335 | | 1 | | | 20 | 1586 | 1605 | | UUACAGAGUCCAUUGAUUAA | | | | AUAAUAAAUGGACUUUGUAA | | | Cleavage | 2 |
|  |  |  | PtrmiR475d-3p | | | PtrPPR624 | | | Potri.T178000.1 | | 3 | 14.992 | | 1 | | | 21 | 675 | 695 | | UUACAGAGUCCAUUGAUUAAG | | | | CUUGAUCAAUGGAUUUUGCAA | | | Cleavage | 1 |
| PtrmiR476 | | | PtrmiR476a | | | PtrPPR51 | | | Potri.001G379500.1 | | 2.5 | 16.274 | | 1 | | | 21 | 985 | 1005 | | UAGUAAUCCUUCUUUGCAAAG | | | | CUUUGCAAGGAAAGGUUGCUA | | | Translation | 1 |
|  |  |  | PtrmiR476a | | | PtrPPR83 | | | Potri.002G182900.1 | | 3 | 16.972 | | 1 | | | 21 | 1758 | 1778 | | UAGUAAUCCUUCUUUGCAAAG | | | | UUUUGUGAAGAAGGGUUAUUU | | | Cleavage | 1 |
|  |  |  | PtrmiR476a | | | PtrPPR84 | | | Potri.002G183100.1 | | 3 | 18.216 | | 1 | | | 21 | 1842 | 1862 | | UAGUAAUCCUUCUUUGCAAAG | | | | UUUUGUGAAGAAGGGUUAUUU | | | Cleavage | 1 |
|  |  |  | PtrmiR476a | | | PtrPPR145 | | | Potri.004G074500.1 | | 1.5 | 9.884 | | 1 | | | 21 | 1731 | 1751 | | UAGUAAUCCUUCUUUGCAAAG | | | | CUUUGUAAAGAAGGAUUGUUA | | | Cleavage | 1 |
|  |  |  | PtrmiR476a | | | PtrPPR146 | | | Potri.004G074700.1 | | 2 | 10.836 | | 1 | | | 21 | 1486 | 1506 | | UAGUAAUCCUUCUUUGCAAAG | | | | CUUUGUAAAGAAGGGUUGUUA | | | Cleavage | 1 |
|  |  |  | PtrmiR476a | | | PtrPPR276 | | | Potri.006G242200.1 | | 2 | 16.909 | | 1 | | | 21 | 1573 | 1593 | | UAGUAAUCCUUCUUUGCAAAG | | | | CUUUGUAAAGAAGGGUUGUUA | | | Cleavage | 1 |
|  |  |  | PtrmiR476a | | | PtrPPR277 | | | Potri.006G242500.1 | | 1.5 | 11.566 | | 1 | | | 21 | 1715 | 1735 | | UAGUAAUCCUUCUUUGCAAAG | | | | CUUUGUAAAGAAGGAUUGUUA | | | Cleavage | 1 |
|  |  |  | PtrmiR476a | | | PtrPPR284 | | | Potri.006G257300.1 | | 1.5 | 9.649 | | 1 | | | 21 | 1842 | 1862 | | UAGUAAUCCUUCUUUGCAAAG | | | | CUUUGUAAAGAAGGAUUGUUA | | | Cleavage | 1 |
|  |  |  | PtrmiR476a | | | PtrPPR287 | | | Potri.006G271200.1 | | 1.5 | 11.977 | | 1 | | | 21 | 1452 | 1472 | | UAGUAAUCCUUCUUUGCAAAG | | | | CUUUGUAAAGAAGGAUUGUUA | | | Cleavage | 1 |
|  |  |  | PtrmiR476a | | | PtrPPR288 | | | Potri.006G271400.1 | | 1.5 | 9.337 | | 1 | | | 21 | 1567 | 1587 | | UAGUAAUCCUUCUUUGCAAAG | | | | CUUUGUAAAGAAGGAUUGUUA | | | Cleavage | 1 |
|  |  |  | PtrmiR476a | | | PtrPPR456 | | | Potri.013G121600.1 | | 2 | 12.511 | | 1 | | | 21 | 376 | 396 | | UAGUAAUCCUUCUUUGCAAAG | | | | CUUUGUAGAGAGGGAUUGCUA | | | Cleavage | 1 |
|  |  |  | PtrmiR476a | | | PtrPPR469 | | | Potri.013G149800.1 | | 0.5 | 9.022 | | 1 | | | 21 | 1629 | 1649 | | UAGUAAUCCUUCUUUGCAAAG | | | | CUUUGCAAAGAAGGAUUGCUA | | | Cleavage | 1 |
|  |  |  | PtrmiR476a | | | PtrPPR470 | | | Potri.013G150100.1 | | 1.5 | 18.657 | | 1 | | | 20 | 1730 | 1749 | | UAGUAAUCCUUCUUUGCAAA | | | | UUUGCAAAGGAGGGUUGCUA | | | Cleavage | 1 |
|  |  |  | PtrmiR476a | | | PtrPPR486 | | | Potri.014G117600.1 | | 3 | 16.766 | | 1 | | | 21 | 1159 | 1179 | | UAGUAAUCCUUCUUUGCAAAG | | | | CUUUGCAAAGAAGGACUAAUA | | | Cleavage | 1 |
|  |  |  | PtrmiR476a | | | PtrPPR520 | | | Potri.016G026400.1 | | 1 | 16.11 | | 1 | | | 21 | 883 | 903 | | UAGUAAUCCUUCUUUGCAAAG | | | | CUUUGCAGAGAAGGAUUGCUA | | | Cleavage | 1 |
|  |  |  | PtrmiR476a | | | PtrPPR564 | | | Potri.017G108100.1 | | 3 | 22.863 | | 1 | | | 20 | 1339 | 1358 | | UAGUAAUCCUUCUUUGCAAA | | | | UUUGCAGAGAAAGAUUGCUU | | | Translation | 1 |
|  |  |  | PtrmiR476a | | | PtrPPR584 | | | Potri.018G038300.1 | | 1 | 14.64 | | 1 | | | 21 | 1709 | 1729 | | UAGUAAUCCUUCUUUGCAAAG | | | | CUUUGCAGAGAAGGAUUGCUA | | | Cleavage | 1 |
|  |  |  | PtrmiR476a | | | PtrPPR593 | | | Potri.018G093700.1 | | 3 | 15.63 | | 1 | | | 21 | 1629 | 1649 | | UAGUAAUCCUUCUUUGCAAAG | | | | UUUUGUAAAGAAGAAUUGCUU | | | Cleavage | 1 |
|  |  |  | PtrmiR476a | | | PtrPPR602 | | | Potri.019G021200.1 | | 1.5 | 12.863 | | 1 | | | 21 | 1691 | 1711 | | UAGUAAUCCUUCUUUGCAAAG | | | | CUUUGUAAAGAAGGAUUGUUA | | | Cleavage | 1 |
|  |  |  | PtrmiR476a | | | PtrPPR607 | | | Potri.019G062300.1 | | 1 | 10.172 | | 1 | | | 21 | 880 | 900 | | UAGUAAUCCUUCUUUGCAAAG | | | | CUUUGUAAAGAAGGAUUAUUA | | | Cleavage | 1 |
|  |  |  | PtrmiR476a | | | PtrPPR613 | | | Potri.019G102100.1 | | 2 | 12.716 | | 1 | | | 21 | 193 | 213 | | UAGUAAUCCUUCUUUGCAAAG | | | | CUUUGCAAAGAAGGAUUGGUA | | | Cleavage | 1 |
|  |  |  | PtrmiR476a | | | PtrPPR617 | | | Potri.T120800.1 | | 2 | 10.839 | | 1 | | | 21 | 1598 | 1618 | | UAGUAAUCCUUCUUUGCAAAG | | | | CUUUGUAAAGAAGGGUUGUUA | | | Cleavage | 1 |
|  |  |  | PtrmiR476b | | | PtrPPR51 | | | Potri.001G379500.1 | | 3 | 16.274 | | 1 | | | 20 | 986 | 1005 | | UAGUAAUUCUUCUUUGCAAA | | | | UUUGCAAGGAAAGGUUGCUA | | | Translation | 1 |
|  |  |  | PtrmiR476b | | | PtrPPR145 | | | Potri.004G074500.1 | | 2 | 9.884 | | 1 | | | 20 | 1732 | 1751 | | UAGUAAUUCUUCUUUGCAAA | | | | UUUGUAAAGAAGGAUUGUUA | | | Cleavage | 1 |
|  |  |  | PtrmiR476b | | | PtrPPR146 | | | Potri.004G074700.1 | | 2.5 | 10.836 | | 1 | | | 20 | 1487 | 1506 | | UAGUAAUUCUUCUUUGCAAA | | | | UUUGUAAAGAAGGGUUGUUA | | | Cleavage | 1 |
|  |  |  | PtrmiR476b | | | PtrPPR276 | | | Potri.006G242200.1 | | 2.5 | 16.909 | | 1 | | | 20 | 1574 | 1593 | | UAGUAAUUCUUCUUUGCAAA | | | | UUUGUAAAGAAGGGUUGUUA | | | Cleavage | 1 |
|  |  |  | PtrmiR476b | | | PtrPPR277 | | | Potri.006G242500.1 | | 2 | 11.566 | | 1 | | | 20 | 1716 | 1735 | | UAGUAAUUCUUCUUUGCAAA | | | | UUUGUAAAGAAGGAUUGUUA | | | Cleavage | 1 |
|  |  |  | PtrmiR476b | | | PtrPPR284 | | | Potri.006G257300.1 | | 2 | 9.649 | | 1 | | | 20 | 1843 | 1862 | | UAGUAAUUCUUCUUUGCAAA | | | | UUUGUAAAGAAGGAUUGUUA | | | Cleavage | 1 |
|  |  |  | PtrmiR476b | | | PtrPPR287 | | | Potri.006G271200.1 | | 2 | 11.977 | | 1 | | | 20 | 1453 | 1472 | | UAGUAAUUCUUCUUUGCAAA | | | | UUUGUAAAGAAGGAUUGUUA | | | Cleavage | 1 |
|  |  |  | PtrmiR476b | | | PtrPPR288 | | | Potri.006G271400.1 | | 2 | 9.337 | | 1 | | | 20 | 1568 | 1587 | | UAGUAAUUCUUCUUUGCAAA | | | | UUUGUAAAGAAGGAUUGUUA | | | Cleavage | 1 |
|  |  |  | PtrmiR476b | | | PtrPPR456 | | | Potri.013G121600.1 | | 2.5 | 12.511 | | 1 | | | 20 | 377 | 396 | | UAGUAAUUCUUCUUUGCAAA | | | | UUUGUAGAGAGGGAUUGCUA | | | Cleavage | 1 |
|  |  |  | PtrmiR476b | | | PtrPPR469 | | | Potri.013G149800.1 | | 1 | 9.022 | | 1 | | | 20 | 1630 | 1649 | | UAGUAAUUCUUCUUUGCAAA | | | | UUUGCAAAGAAGGAUUGCUA | | | Cleavage | 1 |
|  |  |  | PtrmiR476b | | | PtrPPR470 | | | Potri.013G150100.1 | | 2 | 18.657 | | 1 | | | 20 | 1730 | 1749 | | UAGUAAUUCUUCUUUGCAAA | | | | UUUGCAAAGGAGGGUUGCUA | | | Cleavage | 1 |
|  |  |  | PtrmiR476b | | | PtrPPR520 | | | Potri.016G026400.1 | | 1.5 | 16.11 | | 1 | | | 20 | 884 | 903 | | UAGUAAUUCUUCUUUGCAAA | | | | UUUGCAGAGAAGGAUUGCUA | | | Cleavage | 1 |
|  |  |  | PtrmiR476b | | | PtrPPR584 | | | Potri.018G038300.1 | | 1.5 | 14.64 | | 1 | | | 20 | 1710 | 1729 | | UAGUAAUUCUUCUUUGCAAA | | | | UUUGCAGAGAAGGAUUGCUA | | | Cleavage | 1 |
|  |  |  | PtrmiR476b | | | PtrPPR593 | | | Potri.018G093700.1 | | 2 | 15.63 | | 1 | | | 21 | 1629 | 1649 | | UAGUAAUUCUUCUUUGCAAAA | | | | UUUUGUAAAGAAGAAUUGCUU | | | Cleavage | 1 |
|  |  |  | PtrmiR476b | | | PtrPPR602 | | | Potri.019G021200.1 | | 2 | 12.863 | | 1 | | | 20 | 1692 | 1711 | | UAGUAAUUCUUCUUUGCAAA | | | | UUUGUAAAGAAGGAUUGUUA | | | Cleavage | 1 |
|  |  |  | PtrmiR476b | | | PtrPPR607 | | | Potri.019G062300.1 | | 1.5 | 10.172 | | 1 | | | 20 | 881 | 900 | | UAGUAAUUCUUCUUUGCAAA | | | | UUUGUAAAGAAGGAUUAUUA | | | Cleavage | 1 |
|  |  |  | PtrmiR476b | | | PtrPPR613 | | | Potri.019G102100.1 | | 2.5 | 12.716 | | 1 | | | 20 | 194 | 213 | | UAGUAAUUCUUCUUUGCAAA | | | | UUUGCAAAGAAGGAUUGGUA | | | Cleavage | 1 |
|  |  |  | PtrmiR476b | | | PtrPPR617 | | | Potri.T120800.1 | | 2.5 | 10.839 | | 1 | | | 20 | 1599 | 1618 | | UAGUAAUUCUUCUUUGCAAA | | | | UUUGUAAAGAAGGGUUGUUA | | | Cleavage | 1 |
| PtrmiR477 | | | PtrmiR477a-3p | | | PtrPPR392 | | | Potri.010G173400.1 | | 3 | 8.878 | | 1 | | | 21 | 14 | 33 | | GGAUGCCUUUGGGGGAGAUUG | | | | CAAUUUCCCC-AAAGGUAUCC | | | Translation | 1 |
|  |  |  | PtrmiR477c | | | PtrPPR335 | | | Potri.008G212000.1 | | 3 | 16.863 | | 1 | | | 20 | 1538 | 1557 | | GGAAACCUUUUGUGGGGGUU | | | | AACUUUCAAAAAAGGUUUUC | | | Cleavage | 1 |
| PtrmiR482 | | | PtrmiR482d-5p | | | PtrPPR417 | | | Potri.012G031600.1 | | 3 | 20.645 | | 1 | | | 20 | 2586 | 2605 | | GGACAUGGGUUGGUUUGCAA | | | | UUGUAAAUUAACUUAUGUUC | | | Cleavage | 1 |
| PtrmiR6421 | | | PtrmiR6421-3p | | | PtrPPR58 | | | Potri.002G010900.1 | | 2 | 13.411 | | 1 | | | 21 | 633 | 653 | | UAGAGCAGAUUGUAAGGGAAG | | | | UUUCUCUUACGAGCUGCUCUA | | | Translation | 1 |
| PtrmiR6423 | | | PtrmiR6423 | | | PtrPPR287 | | | Potri.006G271200.1 | | 2.5 | 18.111 | | 1 | | | 20 | 214 | 233 | | CCGCUGUCGCCACUAUCUUC | | | | GAUGAUGGUGGCGGCGGCGG | | | Cleavage | 1 |
| PtrmiR6425 | | | PtrmiR6425a-3p | | | PtrPPR94 | | | Potri.002G243600.1 | | 3 | 20.918 | | 1 | | | 21 | 100 | 120 | | UCCAUGGAAGAUAAUGACUCG | | | | UGAGUUGUUAACUUCCAUGGC | | | Translation | 1 |
|  |  |  | PtrmiR6425b-3p | | | PtrPPR94 | | | Potri.002G243600.1 | | 3 | 20.918 | | 1 | | | 21 | 100 | 120 | | UCCAUGGAAGAUAAUGACUCG | | | | UGAGUUGUUAACUUCCAUGGC | | | Translation | 1 |
|  |  |  | PtrmiR6425c-3p | | | PtrPPR94 | | | Potri.002G243600.1 | | 3 | 20.918 | | 1 | | | 21 | 100 | 120 | | UCCAUGGAAGAUAAUGACUCG | | | | UGAGUUGUUAACUUCCAUGGC | | | Translation | 1 |
|  |  |  | PtrmiR6425d-3p | | | PtrPPR94 | | | Potri.002G243600.1 | | 3 | 20.918 | | 1 | | | 21 | 100 | 120 | | UCCAUGGAAGAUAAUGACUCG | | | | UGAGUUGUUAACUUCCAUGGC | | | Translation | 1 |
| PtrmiR6428 | | | PtrmiR6428 | | | PtrPPR450 | | | Potri.013G058900.1 | | 3 | 16.814 | | 1 | | | 20 | 2020 | 2039 | | UCUGGCAACUCAUUAGACUC | | | | GAGUUUUGUGAGAUGCCAGA | | | Cleavage | 1 |
| PtrmiR6445 | | | PtrmiR6445a | | | PtrPPR615 | | | Potri.T035800.1 | | 2.5 | 10.19 | | 1 | | | 20 | 65 | 84 | | UUCAUUCCUCUUCCUAAAAU | | | | AUUUUAUAAGGAGGAAUGAA | | | Cleavage | 1 |
|  |  |  | PtrmiR6445b | | | PtrPPR615 | | | Potri.T035800.1 | | 2.5 | 10.19 | | 1 | | | 20 | 65 | 84 | | UUCAUUCCUCUUCCUAAAAU | | | | AUUUUAUAAGGAGGAAUGAA | | | Cleavage | 1 |
| PtrmiR6450 | | | PtrmiR6450a | | | PtrPPR115 | | | Potri.003G084400.1 | | 3 | 13.206 | | 1 | | | 20 | 431 | 450 | | CUUUGUCAGGACUCAAGGCU | | | | AGUUUCCAGUCCUGACAAAG | | | Cleavage | 1 |
|  |  |  | PtrmiR6450b | | | PtrPPR34 | | | Potri.001G258500.1 | | 3 | 13.796 | | 1 | | | 20 | 681 | 700 | | CGAACACAGGACUCAAGGCU | | | | AGUUUCGAGUCUUGUGUUUG | | | Cleavage | 1 |
| PtrmiR6463 | | | PtrmiR6463 | | | PtrPPR432 | | | Potri.012G135600.1 | | 3 | 15.79 | | 1 | | | 21 | 2232 | 2252 | | UGGAUGAUCAUGUUGGCAACC | | | | GCUUGUCACCAUGGUCAUCCA | | | Cleavage | 1 |
| PtrmiR6466 | | | PtrmiR6466-5p | | | PtrPPR96 | | | Potri.002G248100.1 | | 3 | 16.964 | | 1 | | | 20 | 1619 | 1638 | | UCUGGUAUGAGCAUUUGAUG | | | | CAUCAAGUGUGCAUGUCAGA | | | Translation | 1 |
| PtrmiR6470 | | | PtrmiR6470 | | | PtrPPR62 | | | Potri.002G030200.1 | | 3 | 21.324 | | 1 | | | 20 | 1262 | 1281 | | CUCUGAUAUCAUAUUAAAAA | | | | UAUUUGAUUUGAUGUCAGAG | | | Cleavage | 1 |
|  |  |  | PtrmiR6470 | | | PtrPPR218 | | | Potri.005G160100.1 | | 3 | 16.656 | | 1 | | | 20 | 750 | 769 | | CUCUGAUAUCAUAUUAAAAA | | | | UUUCAAGUAUGAUAUUAGAG | | | Cleavage | 1 |
| PtrmiR6475 | | | PtrmiR6475 | | | PtrPPR488 | | | Potri.014G119000.1 | | 3 | 18.72 | | 1 | | | 20 | 1156 | 1175 | | UCUUGAGAAGUAAAGAACGA | | | | UUGUUCUUUAAUGCUCGAGA | | | Translation | 1 |
| PtrmiR6477 | | | PtrmiR6477 | | | PtrPPR146 | | | Potri.004G147700.1 | | 3 | 11.22 | | 1 | | | 21 | 957 | 977 | | UGAACAGUAGACGUGAAUUAU | | | | AUAAUUCAUGGAUAUUGUUCA | | | Translation | 1 |
|  |  |  | PtrmiR6477 | | | PtrPPR354 | | | Potri.009G109000.1 | | 3 | 11.616 | | 1 | | | 21 | 556 | 576 | | UGAACAGUAGACGUGAAUUAU | | | | AUAAUUCAUGGAUAUUGUUCA | | | Translation | 1 |
| PtrmiR6480 | | | PtrmiR6480 | | | PtrPPR20 | | | Potri.001G139200.1 | | 3 | 15.773 | | 1 | | | 21 | 2472 | 2492 | | UUGCUGAAACGAUUGAACUAU | | | | AUAAUUUAAUUGUCUCAGCAA | | | Cleavage | 1 |
| PtrmiR7814 | | | PtrmiR7814 | | | PtrPPR76 | | | Potri.002G139400.1 | | 2.5 | 16.184 | | 1 | | | 21 | 2027 | 2047 | | UAGAUUGUUUUUAUGCUUUGA | | | | UCAAGGCACAAAAGCAAUUUA | | | Cleavage | 1 |
| PtrmiR7816 | | | PtrmiR7816 | | | PtrPPR235 | | | Potri.006G001200.1 | | 1 | 18.344 | | 1 | | | 20 | 3133 | 3152 | | AAUGUUGUUAUUAACACUGU | | | | ACAGUGUUAGUAACAAUAUU | | | Cleavage | 2 |
|  |  |  | PtrmiR7816 | | | PtrPPR235 | | | Potri.006G001200.1 | | 2 | 13.695 | | 1 | | | 20 | 3082 | 3101 | | AAUGUUGUUAUUAACACUGU | | | | ACAGUGUAAGUAAUAACAUU | | | Cleavage | 2 |
| PtrmiR7817 | | | PtrmiR7817b | | | PtrPPR450 | | | Potri.013G058900.1 | | 3 | 12.893 | | 1 | | | 20 | 3346 | 3365 | | UCUCUUCUGUUCCUGAACGG | | | | UUGGUCAGGAAAAGAAGAGA | | | Translation | 1 |
| PtrmiR7823 | | | PtrmiR7823 | | | PtrPPR34 | | | Potri.001G258500.1 | | 3 | 17.564 | | 1 | | | 20 | 1195 | 1214 | | UUGCAUGCAUGAACUUGAAA | | | | UUACAUGCUCAUGCAUGCAA | | | Cleavage | 1 |
| PtrmiR7826 | | | PtrmiR7826 | | | PtrPPR303 | | | Potri.007G085500.1 | | 3 | 14.667 | | 1 | | | 21 | 872 | 892 | | UUACCAAGUUUCAAAUUCUCA | | | | UGAGAAGUUGAGUUUUGGUAA | | | Translation | 1 |
| **Supplemental Table 4. Results of transcriptomic analysis** | | | | | | | | | | | | | | | | | | | | | | | | | |  |  |  |  |
| **Gene name** | | | | **PPR type** | | | **log2 Ratio**  **(cold/WT)** | | **log2 Ratio (MeJA/WT)** | | | | | **log2 Ratio**  **(M.b/WT)** | | | | | **log2 Ratio**  **(SA/WT)** | | | | **log2 Ratio**  **(salt/WT)** | | **log2 Ratio**  **(wounding/WT)** |  |  |  |  |
| PtrPPR1 | | | | E2 | | | -0.050715639 | | 0.203717429 | | | | | -2.363886259 | | | | | 0.354232836 | | | | -1.364386571 | | -0.709919077 |  |  |  |  |
| PtrPPR2 | | | | E+ | | | -0.033642126 | | 0.236732486 | | | | | -0.124420324 | | | | | 1.508809873 | | | | -0.124920636 | | 0.792581263 |  |  |  |  |
| PtrPPR3 | | | | P | | | -9.692071963 | | 1.121255269 | | | | | -0.861385918 | | | | | 1.634340755 | | | | -9.692071963 | | 0.207618763 |  |  |  |  |
| PtrPPR4 | | | | P | | | -0.886084937 | | 0.590740552 | | | | | -0.391900635 | | | | | 0.808370155 | | | | 0.961236008 | | 0.092141545 |  |  |  |  |
| PtrPPR5 | | | | P | | | 1.814354781 | | 1.285642087 | | | | | 1.008553541 | | | | | 1.877897786 | | | | 1.429879894 | | 1.37200558 |  |  |  |  |
| PtrPPR6 | | | | PLS | | | 1.814354781 | | 1.121255269 | | | | | 2.138614082 | | | | | 2.371306349 | | | | 0.13811377 | | 1.792581263 |  |  |  |  |
| PtrPPR7 | | | | PLS | | | -9.574888424 | | -9.315779806 | | | | | 0.138614082 | | | | | -9.315779806 | | | | -9.574888424 | | -9.574888424 |  |  |  |  |
| PtrPPR8 | | | | E1 | | | 9.073467336 | | NA | | | | | 8.723580521 | | | | | 9.634344693 | | | | NA | | NA |  |  |  |  |
| PtrPPR9 | | | | E+ | | | NA | | 9.693943378 | | | | | NA | | | | | NA | | | | 10.9699105 | | NA |  |  |  |  |
| PtrPPR10 | | | | DYW | | | -0.133177799 | | -0.726741638 | | | | | -0.223955998 | | | | | 0.271770675 | | | | -1.446848731 | | -0.640378144 |  |  |  |  |
| PtrPPR11 | | | | P | | | -1.993000141 | | 0.313900347 | | | | | -1.08377834 | | | | | -1.173014167 | | | | -0.084278652 | | -1.277808065 |  |  |  |  |
| PtrPPR12 | | | | DYW | | | -1.221269129 | | 0.872227721 | | | | | -0.174543803 | | | | | -0.72321125 | | | | -1.312547639 | | -2.149933242 |  |  |  |  |
| PtrPPR13 | | | | P | | | 0.966357874 | | 1.858220863 | | | | | -0.124420324 | | | | | 0.786343848 | | | | -0.446848731 | | 0.622656262 |  |  |  |  |
| PtrPPR14 | | | | P | | | -0.125702679 | | 1.276533494 | | | | | 0.334534292 | | | | | 0.233802825 | | | | -1.835891022 | | -0.164350015 |  |  |  |  |
| PtrPPR15 | | | | E+ | | | 0.714819107 | | -1.200672826 | | | | | -3.183314013 | | | | | 0.534805081 | | | | 0.516625393 | | 0.055615669 |  |  |  |  |
| PtrPPR16 | | | | E2 | | | -1.355570221 | | -0.463707232 | | | | | -1.446348419 | | | | | -10.05730008 | | | | -1.446848731 | | -0.792381237 |  |  |  |  |
| PtrPPR17 | | | | E2 | | | NA | | NA | | | | | NA | | | | | NA | | | | NA | | NA |  |  |  |  |
| PtrPPR18 | | | | P | | | 0.814354781 | | 0.410761886 | | | | | -0.124420324 | | | | | -0.033083906 | | | | -0.124920636 | | -0.055415643 |  |  |  |  |
| PtrPPR19 | | | | DYW | | | -0.14556778 | | -0.627612415 | | | | | -0.471974227 | | | | | -0.842981023 | | | | -1.535899919 | | -0.897734238 |  |  |  |  |
| PtrPPR20 | | | | P | | | -0.448679625 | | 0.706217769 | | | | | -1.861385918 | | | | | 0.749817972 | | | | 0.72307627 | | 0.792581263 |  |  |  |  |
| PtrPPR21 | | | | P | | | 1.022941402 | | 1.423818039 | | | | | 0.401648488 | | | | | 0.786343848 | | | | 1.401148176 | | 1.001167885 |  |  |  |  |
| PtrPPR22 | | | | PLS | | | NA | | NA | | | | | NA | | | | | NA | | | | NA | | NA |  |  |  |  |
| PtrPPR23 | | | | P | | | 0.477319793 | | 0.706217769 | | | | | 0.530931505 | | | | | 0.136841095 | | | | -0.691961229 | | -0.792381237 |  |  |  |  |
| PtrPPR24 | | | | P | | | NA | | NA | | | | | NA | | | | | NA | | | | NA | | NA |  |  |  |  |
| PtrPPR25 | | | | P | | | -0.422684417 | | -0.33817635 | | | | | -0.998889442 | | | | | -0.503162769 | | | | -0.073390335 | | -1.081887855 |  |  |  |  |
| PtrPPR26 | | | | E+ | | | 0.077389186 | | 0.384289674 | | | | | 0.401648488 | | | | | 0.727450159 | | | | 0.275617293 | | -1.114309332 |  |  |  |  |
| PtrPPR27 | | | | PLS | | | NA | | NA | | | | | NA | | | | | NA | | | | NA | | NA |  |  |  |  |
| PtrPPR28 | | | | P | | | 0.498578913 | | 1.025677609 | | | | | -0.546875295 | | | | | -0.191629845 | | | | -0.937835083 | | -0.207418737 |  |  |  |  |
| PtrPPR29 | | | | E+ | | | 0.103861398 | | 0.34364769 | | | | | 0.254091299 | | | | | 0.049378254 | | | | -0.639493809 | | -1.055415643 |  |  |  |  |
| PtrPPR30 | | | | P | | | -0.030084306 | | 1.06062891 | | | | | -0.698735581 | | | | | -0.210098332 | | | | -0.928717739 | | -0.404310786 |  |  |  |  |
| PtrPPR31 | | | | P | | | 0.406270042 | | 0.182655813 | | | | | -0.478057279 | | | | | -0.014752083 | | | | -0.063520091 | | 0.005984901 |  |  |  |  |
| PtrPPR32 | | | | E2 | | | -2.471047438 | | 0.121255269 | | | | | -0.976863136 | | | | | 0.156293458 | | | | -1.977363448 | | -1.492820956 |  |  |  |  |
| PtrPPR33 | | | | E2 | | | NA | | 9.835119441 | | | | | NA | | | | | NA | | | | 10.11108656 | | NA |  |  |  |  |
| PtrPPR34 | | | | E+ | | | 0.907464185 | | 1.799327174 | | | | | 0.624040909 | | | | | 1.534805081 | | | | -0.183814325 | | 1.207618763 |  |  |  |  |
| PtrPPR35 | | | | P | | | NA | | NA | | | | | NA | | | | | NA | | | | NA | | 8.757151612 |  |  |  |  |
| PtrPPR36 | | | | E+ | | | NA | | NA | | | | | NA | | | | | NA | | | | NA | | NA |  |  |  |  |
| PtrPPR37 | | | | P | | | 1.551320375 | | 0.706217769 | | | | | -9.008247932 | | | | | 0.049378254 | | | | 0.13811377 | | 0.792581263 |  |  |  |  |
| PtrPPR38 | | | | P | | | -0.448679625 | | 0.028145864 | | | | | 0.530931505 | | | | | -0.250182028 | | | | 0.661675726 | | -0.332949619 |  |  |  |  |
| PtrPPR39 | | | | P | | | 0.22939228 | | -8.097779154 | | | | | -8.097779154 | | | | | -8.097779154 | | | | -8.356887772 | | -8.356887772 |  |  |  |  |
| PtrPPR40 | | | | P | | | 1.492426686 | | 1.499766892 | | | | | 0.401648488 | | | | | 1.634340755 | | | | 0.986110676 | | 1.69304559 |  |  |  |  |
| PtrPPR41 | | | | P | | | 0.109098046 | | 1.92585958 | | | | | 1.610235109 | | | | | 1.336259402 | | | | 1.920522335 | | 1.379679508 |  |  |  |  |
| PtrPPR42 | | | | DYW | | | -1.886084937 | | 0.005778051 | | | | | -0.239897541 | | | | | 0.670866631 | | | | -0.240397854 | | -1.170892861 |  |  |  |  |
| PtrPPR43 | | | | DYW | | | 1.814354781 | | 1.821694987 | | | | | 0.723576582 | | | | | 2.297305767 | | | | 0.72307627 | | 1.377543764 |  |  |  |  |
| PtrPPR44 | | | | DYW | | | -0.285180893 | | 0.499766892 | | | | | 0.276117606 | | | | | 1.049378254 | | | | 0.72307627 | | 0.470653168 |  |  |  |  |
| PtrPPR45 | | | | P | | | 0.22939228 | | 0.121255269 | | | | | -0.276423418 | | | | | 0.049378254 | | | | -7.422118143 | | -7.422118143 |  |  |  |  |
| PtrPPR46 | | | | P | | | 0.969748463 | | 0.453331319 | | | | | -0.515889352 | | | | | 1.02369161 | | | | 0.706002757 | | 0.398140669 |  |  |  |  |
| PtrPPR47 | | | | DYW | | | 0.22939228 | | 0.858220863 | | | | | -8.639414223 | | | | | 1.271770675 | | | | 1.13811377 | | -0.377343738 |  |  |  |  |
| PtrPPR48 | | | | P | | | -0.136479163 | | -1.116608561 | | | | | -1.183314013 | | | | | NA | | | | -0.827670515 | | -0.925275508 |  |  |  |  |
| PtrPPR49 | | | | P | | | 8.362631474 | | 3.32288913 | | | | | 2.571573489 | | | | | 1.634340755 | | | | 8.271352964 | | NA |  |  |  |  |
| PtrPPR50 | | | | DYW | | | -1.677498316 | | -0.576181961 | | | | | -0.768276514 | | | | | -0.68758734 | | | | -0.961421904 | | -1.611808992 |  |  |  |  |
| PtrPPR51 | | | | P | | | 0.451784701 | | 0.34364769 | | | | | -1.446348419 | | | | | -0.535584247 | | | | -0.124920636 | | -1.377343738 |  |  |  |  |
| PtrPPR52 | | | | P | | | 0.030083472 | | -0.247978541 | | | | | -0.493654134 | | | | | 0.002072539 | | | | -0.568155027 | | 0.060777374 |  |  |  |  |
| PtrPPR53 | | | | P | | | -0.285180893 | | 1.781179827 | | | | | 0.539152011 | | | | | 0.405522064 | | | | 0.376900629 | | -0.628882505 |  |  |  |  |
| PtrPPR54 | | | | P | | | -0.455405488 | | 0.660774799 | | | | | -0.243256554 | | | | | -0.662640983 | | | | -0.658794365 | | -0.843606561 |  |  |  |  |
| PtrPPR55 | | | | E2 | | | -0.507573314 | | 0.121255269 | | | | | -1.446348419 | | | | | 0.727450159 | | | | -0.768776826 | | -0.377343738 |  |  |  |  |
| PtrPPR56 | | | | DYW | | | -0.185645219 | | 0.706217769 | | | | | -0.276423418 | | | | | 1.049378254 | | | | 0.72307627 | | 0.792581263 |  |  |  |  |
| PtrPPR57 | | | | E2 | | | 1.288285969 | | 2.20871811 | | | | | 3.183008201 | | | | | 1.95626885 | | | | 2.64061411 | | 3.114509358 |  |  |  |  |
| PtrPPR58 | | | | P | | | -0.108189079 | | 0.541473007 | | | | | 0.49540644 | | | | | -0.138825481 | | | | -0.488974207 | | -0.116906444 |  |  |  |  |
| PtrPPR59 | | | | PLS | | | -0.536142466 | | -0.966207573 | | | | | -1.948848759 | | | | | -0.578652969 | | | | -1.141994149 | | -0.709919077 |  |  |  |  |
| PtrPPR60 | | | | DYW | | | 0.431026141 | | -0.200672826 | | | | | -2.183314013 | | | | | 0.186881778 | | | | 0.064113188 | | -0.207418737 |  |  |  |  |
| PtrPPR61 | | | | DYW | | | 1.22939228 | | 1.536292768 | | | | | 0.553651581 | | | | | 1.923847372 | | | | 0.875079364 | | 1.207618763 |  |  |  |  |
| PtrPPR62 | | | | DYW | | | 0.250765931 | | 0.340568297 | | | | | -0.172587607 | | | | | 0.005656877 | | | | -0.095085407 | | -0.432205673 |  |  |  |  |
| PtrPPR63 | | | | P | | | NA | | NA | | | | | 7.946486814 | | | | | NA | | | | NA | | 8.274600112 |  |  |  |  |
| PtrPPR64 | | | | E2 | | | 7.619651509 | | 8.25240588 | | | | | NA | | | | | 8.180528866 | | | | NA | | NA |  |  |  |  |
| PtrPPR65 | | | | P | | | NA | | NA | | | | | NA | | | | | NA | | | | NA | | NA |  |  |  |  |
| PtrPPR66 | | | | E2 | | | 1.103861398 | | 1.34364769 | | | | | 0.138614082 | | | | | 1.987977709 | | | | 1.360506191 | | 1.08208788 |  |  |  |  |
| PtrPPR67 | | | | E1 | | | NA | | NA | | | | | NA | | | | | 7.707047887 | | | | NA | | NA |  |  |  |  |
| PtrPPR68 | | | | E2 | | | 0.644429779 | | 0.858220863 | | | | | -0.446348419 | | | | | 2.464415753 | | | | 1.13811377 | | 0.944584357 |  |  |  |  |
| PtrPPR69 | | | | E1 | | | -0.645076838 | | 0.469178572 | | | | | 0.486537385 | | | | | 0.982264058 | | | | 0.000610246 | | 0.207618763 |  |  |  |  |
| PtrPPR70 | | | | P | | | 0.059467278 | | 1.199257781 | | | | | 1.138614082 | | | | | 1.523309442 | | | | 1.668628486 | | 0.845048683 |  |  |  |  |
| PtrPPR71 | | | | E2 | | | 0.22939228 | | 1.34364769 | | | | | 0.553651581 | | | | | 0.049378254 | | | | 0.553151269 | | -1.377343738 |  |  |  |  |
| PtrPPR72 | | | | PLS | | | 8.749075202 | | NA | | | | | NA | | | | | NA | | | | NA | | NA |  |  |  |  |
| PtrPPR73 | | | | P | | | -0.08471631 | | 0.685156154 | | | | | -0.175494509 | | | | | 0.675563417 | | | | 0.369439316 | | -0.030540975 |  |  |  |  |
| PtrPPR74 | | | | PLS | | | NA | | NA | | | | | NA | | | | | NA | | | | NA | | NA |  |  |  |  |
| PtrPPR75 | | | | P | | | -0.234294382 | | -0.198785717 | | | | | -0.773478829 | | | | | -0.753263514 | | | | -0.196141075 | | -0.505623921 |  |  |  |  |
| PtrPPR76 | | | | P | | | -0.121919622 | | 0.241735645 | | | | | -0.702882096 | | | | | -0.226746151 | | | | -0.789282481 | | -0.320312793 |  |  |  |  |
| PtrPPR77 | | | | DYW | | | -0.185645219 | | 0.443183364 | | | | | -0.276423418 | | | | | 0.856733176 | | | | -0.27692373 | | 1.014973685 |  |  |  |  |
| PtrPPR78 | | | | P | | | NA | | NA | | | | | NA | | | | | NA | | | | NA | | NA |  |  |  |  |
| PtrPPR79 | | | | E+ | | | -0.355570221 | | 0.121255269 | | | | | -0.66874084 | | | | | 0.357500549 | | | | 0.205227966 | | 0.063228853 |  |  |  |  |
| PtrPPR80 | | | | P | | | -0.488836752 | | 0.003418778 | | | | | 0.11004493 | | | | | -0.337644869 | | | | 1.109544618 | | -0.879844079 |  |  |  |  |
| PtrPPR81 | | | | P | | | NA | | NA | | | | | NA | | | | | NA | | | | NA | | NA |  |  |  |  |
| PtrPPR82 | | | | P | | | 1.644429779 | | 0.752021459 | | | | | 0.091308367 | | | | | 0.58703504 | | | | 0.875079364 | | 0.207618763 |  |  |  |  |
| PtrPPR83 | | | | P | | | -7.112668988 | | 0.121255269 | | | | | -6.85356037 | | | | | -6.85356037 | | | | -7.112668988 | | -7.112668988 |  |  |  |  |
| PtrPPR84 | | | | P | | | 1.814354781 | | 0.121255269 | | | | | 1.138614082 | | | | | -6.85356037 | | | | -7.112668988 | | -7.112668988 |  |  |  |  |
| PtrPPR85 | | | | P | | | NA | | NA | | | | | NA | | | | | NA | | | | NA | | NA |  |  |  |  |
| PtrPPR86 | | | | PLS | | | -1.033642126 | | -0.111831754 | | | | | -1.030778265 | | | | | -0.5821443 | | | | -1.446848731 | | -0.129416225 |  |  |  |  |
| PtrPPR87 | | | | P | | | NA | | NA | | | | | 7.59394524 | | | | | 9.826637507 | | | | 8.852553546 | | 7.922058539 |  |  |  |  |
| PtrPPR88 | | | | PLS | | | NA | | NA | | | | | NA | | | | | NA | | | | NA | | NA |  |  |  |  |
| PtrPPR89 | | | | DYW | | | 0.036747202 | | 0.443183364 | | | | | -0.276423418 | | | | | 0.749817972 | | | | 0.597545388 | | -1.792381237 |  |  |  |  |
| PtrPPR90 | | | | DWY | | | NA | | NA | | | | | NA | | | | | NA | | | | NA | | NA |  |  |  |  |
| PtrPPR91 | | | | PLS | | | NA | | NA | | | | | NA | | | | | NA | | | | NA | | NA |  |  |  |  |
| PtrPPR92 | | | | DYW | | | 0.814354781 | | 0.443183364 | | | | | 0.945969004 | | | | | 1.049378254 | | | | 0.460041865 | | 0.792581263 |  |  |  |  |
| PtrPPR93 | | | | DYW | | | 0.714819107 | | 0.121255269 | | | | | 0.138614082 | | | | | 1.049378254 | | | | 0.903648516 | | -0.529346832 |  |  |  |  |
| PtrPPR94 | | | | P | | | 0.557446478 | | 1.181376261 | | | | | 0.793478596 | | | | | 0.352770398 | | | | 0.773349477 | | 0.380950365 |  |  |  |  |
| PtrPPR95 | | | | E+ | | | -0.577962642 | | -0.101137153 | | | | | -1.08377834 | | | | | 0.411948333 | | | | -1.669241152 | | -1.014773659 |  |  |  |  |
| PtrPPR96 | | | | P | | | -0.433572733 | | -0.603110289 | | | | | -0.408873714 | | | | | -0.340568264 | | | | 0.797076852 | | -0.647991328 |  |  |  |  |
| PtrPPR97 | | | | E+ | | | 0.644429779 | | 0.858220863 | | | | | 0.138614082 | | | | | 0.786343848 | | | | 0.13811377 | | -1.377343738 |  |  |  |  |
| PtrPPR98 | | | | P | | | NA | | NA | | | | | NA | | | | | NA | | | | NA | | NA |  |  |  |  |
| PtrPPR99 | | | | P | | | 0.451784701 | | 0.34364769 | | | | | -0.124420324 | | | | | 1.049378254 | | | | 1.012582888 | | -0.792381237 |  |  |  |  |
| PtrPPR100 | | | | DYW | | | 0.22939228 | | 0.34364769 | | | | | -0.446348419 | | | | | 0.634340755 | | | | -2.446848731 | | -0.377343738 |  |  |  |  |
| PtrPPR101 | | | | P | | | 0.303392861 | | 1.121255269 | | | | | 0.64557407 | | | | | 0.325012697 | | | | 0.645073758 | | 0.714578751 |  |  |  |  |
| PtrPPR102 | | | | PLS | | | NA | | NA | | | | | NA | | | | | NA | | | | NA | | NA |  |  |  |  |
| PtrPPR103 | | | | PLS | | | 0.22939228 | | 0.121255269 | | | | | 0.138614082 | | | | | 2.049378254 | | | | 1.13811377 | | 0.207618763 |  |  |  |  |
| PtrPPR104 | | | | PLS | | | NA | | NA | | | | | NA | | | | | NA | | | | NA | | NA |  |  |  |  |
| PtrPPR105 | | | | P | | | 0.607903903 | | 0.969252175 | | | | | 0.517125705 | | | | | 0.31241266 | | | | 0.401148176 | | 0.207618763 |  |  |  |  |
| PtrPPR106 | | | | PLS | | | -1.577962642 | | -0.101137153 | | | | | -0.346812745 | | | | | 1.049378254 | | | | -0.347313057 | | -1.59973616 |  |  |  |  |
| PtrPPR107 | | | | E+ | | | 8.236472924 | | 7.869227295 | | | | | 7.886586108 | | | | | 8.79735028 | | | | 8.145194414 | | NA |  |  |  |  |
| PtrPPR108 | | | | E+ | | | NA | | NA | | | | | NA | | | | | NA | | | | NA | | NA |  |  |  |  |
| PtrPPR109 | | | | E+ | | | NA | | NA | | | | | NA | | | | | NA | | | | NA | | NA |  |  |  |  |
| PtrPPR110 | | | | P | | | NA | | NA | | | | | NA | | | | | NA | | | | NA | | NA |  |  |  |  |
| PtrPPR111 | | | | DYW | | | -1.256034547 | | -0.811630536 | | | | | -2.253703341 | | | | | -0.88350755 | | | | -0.794772034 | | -0.862770565 |  |  |  |  |
| PtrPPR112 | | | | P | | | NA | | 1.121255269 | | | | | 0.33125916 | | | | | 1.329486173 | | | | NA | | NA |  |  |  |  |
| PtrPPR113 | | | | DYW | | | -0.963252798 | | -0.178305013 | | | | | -1.054030996 | | | | | -0.365659245 | | | | -0.691961229 | | -0.792381237 |  |  |  |  |
| PtrPPR114 | | | | DYW | | | -0.618604627 | | -0.241314811 | | | | | -1.03131092 | | | | | 0.464415753 | | | | 0.290116863 | | -0.962306239 |  |  |  |  |
| PtrPPR115 | | | | P | | | -0.033642126 | | -0.615710326 | | | | | -0.183314013 | | | | | -0.050157419 | | | | 0.401148176 | | 0.388191008 |  |  |  |  |
| PtrPPR116 | | | | E2 | | | -0.993000141 | | -1.686099653 | | | | | -1.08377834 | | | | | -0.173014167 | | | | -1.084278652 | | -1.59973616 |  |  |  |  |
| PtrPPR117 | | | | E+ | | | 0.091888756 | | 0.362263368 | | | | | -2.320817537 | | | | | -0.825090864 | | | | -0.513962927 | | -1.251812856 |  |  |  |  |
| PtrPPR118 | | | | E+ | | | 0.22939228 | | 0.443183364 | | | | | 0.138614082 | | | | | -0.365659245 | | | | 0.72307627 | | -0.792381237 |  |  |  |  |
| PtrPPR119 | | | | P | | | 0.404780564 | | 2.006877247 | | | | | 0.546596824 | | | | | 1.445377304 | | | | 0.095258291 | | 0.956498836 |  |  |  |  |
| PtrPPR120 | | | | P | | | 1.029093629 | | 1.038793108 | | | | | -0.294345326 | | | | | 0.720755507 | | | | 1.383226268 | | 0.700658774 |  |  |  |  |
| PtrPPR121 | | | | E2 | | | 2.036747202 | | 3.369182782 | | | | | 2.308539083 | | | | | 1.634340755 | | | | 3.045004365 | | 1.529546857 |  |  |  |  |
| PtrPPR122 | | | | P | | | -0.355570221 | | -0.048669733 | | | | | -0.03131092 | | | | | -0.535584247 | | | | 0.668628486 | | -0.377343738 |  |  |  |  |
| PtrPPR123 | | | | E2 | | | -0.185645219 | | 0.443183364 | | | | | -0.276423418 | | | | | 0.371306349 | | | | -0.27692373 | | -0.207418737 |  |  |  |  |
| PtrPPR124 | | | | DYW | | | -1.024364312 | | -0.511012947 | | | | | -1.35615061 | | | | | -0.904818056 | | | | -1.646157539 | | -0.839686952 |  |  |  |  |
| PtrPPR125 | | | | PLS | | | NA | | NA | | | | | NA | | | | | NA | | | | NA | | NA |  |  |  |  |
| PtrPPR126 | | | | DYW | | | 2.036747202 | | 2.121255269 | | | | | 0.945969004 | | | | | 1.508809873 | | | | 0.72307627 | | 0.792581263 |  |  |  |  |
| PtrPPR127 | | | | DYW | | | -1.519068953 | | 0.236732486 | | | | | -0.931775246 | | | | | -0.039158421 | | | | -0.896651648 | | -1.59973616 |  |  |  |  |
| PtrPPR128 | | | | P | | | 0.22939228 | | -0.200672826 | | | | | -0.861385918 | | | | | 0.634340755 | | | | 0.275617293 | | 0.207618763 |  |  |  |  |
| PtrPPR129 | | | | P | | | 1.136282876 | | 0.29118027 | | | | | 0.945969004 | | | | | 0.371306349 | | | | 1.945468692 | | 0.667050381 |  |  |  |  |
| PtrPPR130 | | | | P | | | -0.77060772 | | 1.068787849 | | | | | -0.66874084 | | | | | 0.996910834 | | | | 0.330758848 | | -0.59973616 |  |  |  |  |
| PtrPPR131 | | | | E+ | | | 0.399317281 | | 1.369182782 | | | | | 0.308539083 | | | | | 1.219303255 | | | | 2.182507889 | | 0.792581263 |  |  |  |  |
| PtrPPR132 | | | | P | | | -1.253000487 | | -0.632683404 | | | | | -0.950114837 | | | | | -1.391194337 | | | | -1.929241498 | | -1.303343157 |  |  |  |  |
| PtrPPR133 | | | | P | | | -0.111644638 | | -2.126672245 | | | | | -1.408873714 | | | | | 0.159002745 | | | | 0.316451011 | | -0.870383749 |  |  |  |  |
| PtrPPR134 | | | | P | | | 0.27830188 | | 0.935699615 | | | | | 0.450558088 | | | | | -0.349171122 | | | | 0.565534993 | | 0.437100609 |  |  |  |  |
| PtrPPR135 | | | | PLS | | | -0.481101103 | | -0.775651238 | | | | | -1.092711464 | | | | | 0.101845674 | | | | -0.529310891 | | -0.54726874 |  |  |  |  |
| PtrPPR136 | | | | P | | | -0.092535815 | | 0.121255269 | | | | | -0.112924685 | | | | | 0.61497543 | | | | -0.046310801 | | -0.114309332 |  |  |  |  |
| PtrPPR137 | | | | P | | | 0.935661077 | | 0.19525585 | | | | | -0.30195851 | | | | | 0.608805663 | | | | 1.475148757 | | 0.714578751 |  |  |  |  |
| PtrPPR138 | | | | DYW | | | 0.22939228 | | 0.749286491 | | | | | -0.320817537 | | | | | 0.397301557 | | | | 0.678682151 | | -0.081887855 |  |  |  |  |
| PtrPPR139 | | | | DYW | | | 1.688823899 | | -1.878744731 | | | | | 0.460542177 | | | | | 0.049378254 | | | | -0.27692373 | | 0.207618763 |  |  |  |  |
| PtrPPR140 | | | | E2 | | | -0.77060772 | | -0.293782231 | | | | | -0.124420324 | | | | | 0.856733176 | | | | 0.253590987 | | -0.377343738 |  |  |  |  |
| PtrPPR141 | | | | DYW | | | 0.492426686 | | 0.799327174 | | | | | 0.986610988 | | | | | 1.31241266 | | | | 1.623540597 | | 0.207618763 |  |  |  |  |
| PtrPPR142 | | | | P | | | 1.892357293 | | 2.665575785 | | | | | 2.197507771 | | | | | 2.108271943 | | | | 1.64061411 | | 1.529546857 |  |  |  |  |
| PtrPPR143 | | | | E2 | | | -0.355570221 | | -1.463707232 | | | | | -1.446348419 | | | | | -1.535584247 | | | | 0.875079364 | | -0.377343738 |  |  |  |  |
| PtrPPR144 | | | | P | | | -0.156261413 | | 0.224348762 | | | | | -0.72550249 | | | | | -0.603971222 | | | | -1.135647043 | | -0.689287744 |  |  |  |  |
| PtrPPR145 | | | | P | | | 0.814354781 | | 1.384289674 | | | | | 0.723576582 | | | | | 0.31241266 | | | | 0.816185675 | | 0.345122286 |  |  |  |  |
| PtrPPR146 | | | | P | | | 0.528952562 | | 0.944377507 | | | | | -0.102394018 | | | | | -0.329133369 | | | | 0.829991474 | | 0.207618763 |  |  |  |  |
| PtrPPR147 | | | | P | | | 0.644429779 | | 1.236732486 | | | | | 0.875579676 | | | | | 1.371306349 | | | | 1.553151269 | | 1.207618763 |  |  |  |  |
| PtrPPR148 | | | | P | | | -0.633104196 | | 0.258758792 | | | | | -0.723882394 | | | | | -0.024622327 | | | | 0.064113188 | | -0.413869614 |  |  |  |  |
| PtrPPR149 | | | | P | | | -0.683144879 | | -0.556816636 | | | | | -1.003404923 | | | | | -0.04373115 | | | | -0.402454612 | | -0.885490642 |  |  |  |  |
| PtrPPR150 | | | | E+ | | | 0.881468977 | | 0.121255269 | | | | | -0.346812745 | | | | | 0.826985833 | | | | 0.652686943 | | -0.277808065 |  |  |  |  |
| PtrPPR151 | | | | E2 | | | -1.256034547 | | -0.101137153 | | | | | 0.031698878 | | | | | 0.765585288 | | | | 0.13811377 | | -0.59973616 |  |  |  |  |
| PtrPPR152 | | | | P | | | NA | | NA | | | | | NA | | | | | NA | | | | NA | | NA |  |  |  |  |
| PtrPPR153 | | | | E+ | | | 0.126298787 | | 0.689539028 | | | | | -0.812476318 | | | | | 0.191397259 | | | | -0.260435607 | | 0.10452527 |  |  |  |  |
| PtrPPR154 | | | | E+ | | | 1.399317281 | | 0.706217769 | | | | | -8.68880225 | | | | | 0.856733176 | | | | 0.945468692 | | 0.207618763 |  |  |  |  |
| PtrPPR155 | | | | P | | | 8.877742866 | | NA | | | | | 8.52785605 | | | | | NA | | | | 9.786464355 | | NA |  |  |  |  |
| PtrPPR156 | | | | P | | | 0.122477076 | | -0.101137153 | | | | | -0.209309222 | | | | | 0.826985833 | | | | 0.854320804 | | -0.277808065 |  |  |  |  |
| PtrPPR157 | | | | P | | | -0.548215299 | | 0.410761886 | | | | | 0.216616594 | | | | | -0.661115129 | | | | -0.27692373 | | -1.792381237 |  |  |  |  |
| PtrPPR158 | | | | E+ | | | NA | | 8.171444964 | | | | | NA | | | | | NA | | | | 7.447412083 | | NA |  |  |  |  |
| PtrPPR159 | | | | E+ | | | 7.538690593 | | 8.756407465 | | | | | 8.773766278 | | | | | 8.68453045 | | | | 9.032374583 | | NA |  |  |  |  |
| PtrPPR160 | | | | P | | | -0.233234678 | | 0.980556406 | | | | | 0.188799839 | | | | | -0.040160752 | | | | -0.419030787 | | -0.255008195 |  |  |  |  |
| PtrPPR161 | | | | PLS | | | NA | | NA | | | | | NA | | | | | NA | | | | NA | | NA |  |  |  |  |
| PtrPPR162 | | | | DYW | | | 1.036747202 | | 1.580686887 | | | | | 0.723576582 | | | | | 1.371306349 | | | | 1.838553488 | | 1.207618763 |  |  |  |  |
| PtrPPR163 | | | | DYW | | | 3.399317281 | | 2.121255269 | | | | | 1.723576582 | | | | | 2.634340755 | | | | 3.308038771 | | 3.667050381 |  |  |  |  |
| PtrPPR164 | | | | P | | | 0.534246861 | | 0.033792427 | | | | | -0.489417141 | | | | | 0.131840414 | | | | -0.627420977 | | -0.179404361 |  |  |  |  |
| PtrPPR165 | | | | P | | | -1.018535234 | | -0.426232527 | | | | | -1.109313432 | | | | | -0.028624258 | | | | -0.650382125 | | -1.45534625 |  |  |  |  |
| PtrPPR166 | | | | P | | | -0.301122437 | | 0.880247169 | | | | | -1.976863136 | | | | | 1.436401377 | | | | 0.961236008 | | 0.594641886 |  |  |  |  |
| PtrPPR167 | | | | E+ | | | -1.507573314 | | 0.606682096 | | | | | -0.861385918 | | | | | 0.31241266 | | | | 0.516625393 | | -2.114309332 |  |  |  |  |
| PtrPPR168 | | | | DYW | | | 2.22939228 | | 1.706217769 | | | | | 3.138614082 | | | | | 3.508809873 | | | | 3.597545388 | | 1.207618763 |  |  |  |  |
| PtrPPR169 | | | | P | | | -0.118531023 | | -0.878744731 | | | | | -1.1662405 | | | | | -0.88350755 | | | | -0.553763935 | | -0.792381237 |  |  |  |  |
| PtrPPR170 | | | | E+ | | | NA | | NA | | | | | NA | | | | | NA | | | | NA | | NA |  |  |  |  |
| PtrPPR171 | | | | P | | | -0.677498316 | | -1.3002085 | | | | | -1.282849687 | | | | | -0.372085514 | | | | -0.805302702 | | -1.32076021 |  |  |  |  |
| PtrPPR172 | | | | P | | | -0.858070561 | | -0.012011262 | | | | | 0.180434257 | | | | | 0.283843508 | | | | -0.425787115 | | -0.631916565 |  |  |  |  |
| PtrPPR173 | | | | PLS | | | NA | | NA | | | | | NA | | | | | NA | | | | NA | | NA |  |  |  |  |
| PtrPPR174 | | | | DYW | | | -0.567074326 | | -0.222699133 | | | | | -0.320817537 | | | | | 0.290386354 | | | | 0.047915961 | | -0.836775357 |  |  |  |  |
| PtrPPR175 | | | | P | | | 0.435843157 | | -0.994221949 | | | | | -0.391900635 | | | | | -0.651061464 | | | | -1.240397854 | | -0.685466034 |  |  |  |  |
| PtrPPR176 | | | | DYW | | | 0.22939228 | | 0.121255269 | | | | | 0.138614082 | | | | | 1.049378254 | | | | 0.72307627 | | 1.207618763 |  |  |  |  |
| PtrPPR177 | | | | DYW | | | -7.438315371 | | 0.121255269 | | | | | -7.179206753 | | | | | 1.049378254 | | | | 0.13811377 | | 1.792581263 |  |  |  |  |
| PtrPPR178 | | | | E2 | | | 0.22939228 | | 2.268096657 | | | | | 1.501184161 | | | | | 1.701454951 | | | | 2.031198566 | | 0.859695459 |  |  |  |  |
| PtrPPR179 | | | | DYW | | | -0.256034547 | | 0.220790942 | | | | | -0.861385918 | | | | | 0.634340755 | | | | 0.13811377 | | -0.014773659 |  |  |  |  |
| PtrPPR180 | | | | DYW | | | -0.332486608 | | 0.113477918 | | | | | -0.378176916 | | | | | -0.478553302 | | | | -1.02600561 | | -0.400802711 |  |  |  |  |
| PtrPPR181 | | | | P | | | NA | | NA | | | | | NA | | | | | NA | | | | NA | | NA |  |  |  |  |
| PtrPPR182 | | | | P | | | 0.881468977 | | 0.898862847 | | | | | 0.653187255 | | | | | 0.563951427 | | | | 1.500683849 | | 0.40026384 |  |  |  |  |
| PtrPPR183 | | | | E2 | | | -0.77060772 | | 0.536292768 | | | | | 0.723576582 | | | | | 0.786343848 | | | | 0.13811377 | | -0.792381237 |  |  |  |  |
| PtrPPR184 | | | | DYW | | | NA | | NA | | | | | NA | | | | | NA | | | | NA | | NA |  |  |  |  |
| PtrPPR185 | | | | P | | | 1.907464185 | | 1.706217769 | | | | | 1.401648488 | | | | | 2.371306349 | | | | 1.516625393 | | 1.055615669 |  |  |  |  |
| PtrPPR186 | | | | P | | | NA | | NA | | | | | NA | | | | | NA | | | | NA | | NA |  |  |  |  |
| PtrPPR187 | | | | P | | | 1.036747202 | | -0.878744731 | | | | | -0.861385918 | | | | | 0.049378254 | | | | 0.72307627 | | 0.207618763 |  |  |  |  |
| PtrPPR188 | | | | P | | | 1.22939228 | | 1.644817225 | | | | | 0.723576582 | | | | | 1.634340755 | | | | 1.045004365 | | 1.014973685 |  |  |  |  |
| PtrPPR189 | | | | P | | | 1.814354781 | | 1.121255269 | | | | | 0.723576582 | | | | | 0.371306349 | | | | 0.460041865 | | 1.207618763 |  |  |  |  |
| PtrPPR190 | | | | P | | | 0.22939228 | | -1.686099653 | | | | | -2.66874084 | | | | | -0.436048573 | | | | -0.347313057 | | -0.59973616 |  |  |  |  |
| PtrPPR191 | | | | P | | | NA | | NA | | | | | NA | | | | | NA | | | | NA | | NA |  |  |  |  |
| PtrPPR192 | | | | P | | | NA | | NA | | | | | NA | | | | | NA | | | | NA | | NA |  |  |  |  |
| PtrPPR193 | | | | P | | | 0.814354781 | | 0.580686887 | | | | | 0.460542177 | | | | | 0.95626885 | | | | 0.13811377 | | 0.014973685 |  |  |  |  |
| PtrPPR194 | | | | P | | | NA | | NA | | | | | NA | | | | | NA | | | | NA | | NA |  |  |  |  |
| PtrPPR195 | | | | P | | | 8.736817736 | | 7.369572107 | | | | | 8.38693092 | | | | | 8.882657593 | | | | 8.645539226 | | 7.715044218 |  |  |  |  |
| PtrPPR196 | | | | P | | | -7.661871725 | | 0.121255269 | | | | | 0.138614082 | | | | | -7.402763108 | | | | 0.13811377 | | 1.207618763 |  |  |  |  |
| PtrPPR197 | | | | P | | | -0.185645219 | | 0.121255269 | | | | | -0.539457823 | | | | | 0.049378254 | | | | -0.86188623 | | 0.377543764 |  |  |  |  |
| PtrPPR198 | | | | P | | | 9.489875567 | | 7.537667438 | | | | | 7.555026251 | | | | | 8.465790423 | | | | NA | | 8.883139549 |  |  |  |  |
| PtrPPR199 | | | | E+ | | | 9.186719889 | | 8.234511759 | | | | | 8.836833073 | | | | | NA | | | | 7.510478878 | | NA |  |  |  |  |
| PtrPPR200 | | | | E+ | | | 9.525979541 | | 9.573771411 | | | | | 9.176092725 | | | | | 8.501894397 | | | | 7.84973853 | | 7.919243523 |  |  |  |  |
| PtrPPR201 | | | | P | | | -0.185645219 | | -0.141779137 | | | | | -0.358885578 | | | | | 0.164855471 | | | | -0.27692373 | | -0.67690402 |  |  |  |  |
| PtrPPR202 | | | | E2 | | | -0.355570221 | | 1.121255269 | | | | | 0.875579676 | | | | | 0.786343848 | | | | 0.553151269 | | 0.207618763 |  |  |  |  |
| PtrPPR203 | | | | P | | | 0.140855605 | | 0.298793454 | | | | | -1.446348419 | | | | | -0.193192049 | | | | -0.084278652 | | -0.429811158 |  |  |  |  |
| PtrPPR204 | | | | P | | | -0.507573314 | | 0.799327174 | | | | | -0.598351512 | | | | | 0.897375161 | | | | 0.816185675 | | -0.529346832 |  |  |  |  |
| PtrPPR205 | | | | P | | | NA | | NA | | | | | NA | | | | | NA | | | | NA | | NA |  |  |  |  |
| PtrPPR206 | | | | P | | | -0.185645219 | | 0.236732486 | | | | | 0.361006503 | | | | | 0.786343848 | | | | 0.012582888 | | 0.792581263 |  |  |  |  |
| PtrPPR207 | | | | P | | | 0.080528894 | | -1.229241978 | | | | | -0.88995507 | | | | | 0.049378254 | | | | 0.594971445 | | 0.058755377 |  |  |  |  |
| PtrPPR208 | | | | E2 | | | 0.477319793 | | 1.075451579 | | | | | 0.530931505 | | | | | 1.334780473 | | | | 0.225576611 | | -0.091941519 |  |  |  |  |
| PtrPPR209 | | | | DYW | | | 1.714819107 | | 2.191644597 | | | | | 1.138614082 | | | | | 2.975377673 | | | | 1.275617293 | | 2.055615669 |  |  |  |  |
| PtrPPR210 | | | | P | | | -1.577962642 | | -0.811630536 | | | | | -1.253703341 | | | | | -0.535584247 | | | | -0.446848731 | | -2.59973616 |  |  |  |  |
| PtrPPR211 | | | | P | | | 1.814354781 | | 1.995724387 | | | | | 0.875579676 | | | | | 2.049378254 | | | | 2.012582888 | | 0.944584357 |  |  |  |  |
| PtrPPR212 | | | | P | | | 0.22939228 | | 0.121255269 | | | | | 0.723576582 | | | | | 0.371306349 | | | | 0.945468692 | | -0.792381237 |  |  |  |  |
| PtrPPR213 | | | | E2 | | | -0.993000141 | | -0.516174652 | | | | | -1.66874084 | | | | | 0.634340755 | | | | -0.499316151 | | -0.59973616 |  |  |  |  |
| PtrPPR214 | | | | E2 | | | 1.688823899 | | 1.121255269 | | | | | 1.308539083 | | | | | 0.634340755 | | | | 1.13811377 | | 0.792581263 |  |  |  |  |
| PtrPPR215 | | | | P | | | -0.256034547 | | 0.121255269 | | | | | -0.08377834 | | | | | 1.489950845 | | | | 1.652686943 | | 0.40026384 |  |  |  |  |
| PtrPPR216 | | | | DYW | | | -0.55242755 | | -0.05051108 | | | | | -0.476095762 | | | | | -0.395406589 | | | | -2.389133233 | | -0.980826327 |  |  |  |  |
| PtrPPR217 | | | | DWY | | | NA | | NA | | | | | NA | | | | | NA | | | | NA | | NA |  |  |  |  |
| PtrPPR218 | | | | P | | | 0.743965453 | | 0.773331965 | | | | | 0.790690778 | | | | | 1.049378254 | | | | 1.330758848 | | 0.570188842 |  |  |  |  |
| PtrPPR219 | | | | PLS | | | -0.398638943 | | 1.078186547 | | | | | -0.141493837 | | | | | 0.546877914 | | | | -0.489917453 | | -1.072489157 |  |  |  |  |
| PtrPPR220 | | | | P | | | 1.141929439 | | 0.355720522 | | | | | 0.299078754 | | | | | 0.605771603 | | | | 0.694507118 | | 0.368083435 |  |  |  |  |
| PtrPPR221 | | | | E+ | | | 2.551320375 | | 1.121255269 | | | | | 0.138614082 | | | | | 1.371306349 | | | | 0.72307627 | | 1.529546857 |  |  |  |  |
| PtrPPR222 | | | | E+ | | | -0.149119343 | | 0.880247169 | | | | | -0.561825636 | | | | | 0.741255959 | | | | 0.245028974 | | -0.492820956 |  |  |  |  |
| PtrPPR223 | | | | DYW | | | -0.940532722 | | 0.199257781 | | | | | 0.056151922 | | | | | 0.271770675 | | | | 0.360506191 | | -0.055415643 |  |  |  |  |
| PtrPPR224 | | | | E+ | | | 0.951858304 | | 1.29118027 | | | | | 0.861080106 | | | | | 0.049378254 | | | | -1.376459403 | | 0.409252624 |  |  |  |  |
| PtrPPR225 | | | | P | | | 0.462053037 | | -0.223392903 | | | | | -0.229117703 | | | | | 0.658187497 | | | | 0.623540597 | | 0.133618181 |  |  |  |  |
| PtrPPR226 | | | | P | | | 0.463857534 | | 0.033792427 | | | | | -0.041958164 | | | | | 0.662355131 | | | | 0.298578442 | | -0.557915984 |  |  |  |  |
| PtrPPR227 | | | | DYW | | | 9.266112414 | | 8.898866785 | | | | | 8.501188099 | | | | | 8.826989771 | | | | 9.174833904 | | 8.829301398 |  |  |  |  |
| PtrPPR228 | | | | P | | | NA | | NA | | | | | NA | | | | | NA | | | | NA | | NA |  |  |  |  |
| PtrPPR229 | | | | P | | | -0.741461374 | | -0.945017951 | | | | | -0.891133262 | | | | | -1.02927869 | | | | -1.458674166 | | -0.908840214 |  |  |  |  |
| PtrPPR230 | | | | P | | | 0.100109263 | | -0.030747825 | | | | | -0.467106979 | | | | | -0.351984308 | | | | 0.435095508 | | 0.009073083 |  |  |  |  |
| PtrPPR231 | | | | P | | | NA | | 0.199257781 | | | | | NA | | | | | NA | | | | NA | | NA |  |  |  |  |
| PtrPPR232 | | | | P | | | 0.059467278 | | NA | | | | | -1.446348419 | | | | | -0.213656152 | | | | -0.709883137 | | -0.962306239 |  |  |  |  |
| PtrPPR233 | | | | P | | | 0.297563783 | | 1.097796296 | | | | | 0.524267774 | | | | | 0.2037064 | | | | 0.393370825 | | 0.696365948 |  |  |  |  |
| PtrPPR234 | | | | P | | | -0.658132991 | | 0.818692499 | | | | | -0.611407665 | | | | | 0.697905884 | | | | 0.286212409 | | -0.246947101 |  |  |  |  |
| PtrPPR235 | | | | DYW | | | 0.591962359 | | 0.635828441 | | | | | -1.08377834 | | | | | -0.173014167 | | | | 0.790190466 | | -0.014773659 |  |  |  |  |
| PtrPPR236 | | | | P | | | -0.438032381 | | 0.858220863 | | | | | 0.513009597 | | | | | -0.483116827 | | | | -0.916334014 | | -0.739913818 |  |  |  |  |
| PtrPPR237 | | | | P | | | 0.814354781 | | 0.536292768 | | | | | -1.446348419 | | | | | 0.464415753 | | | | -0.124920636 | | -0.792381237 |  |  |  |  |
| PtrPPR238 | | | | E+ | | | -0.707413894 | | -0.086852927 | | | | | -1.120120187 | | | | | -0.624393514 | | | | -1.404413465 | | -1.771007587 |  |  |  |  |
| PtrPPR239 | | | | P | | | 1.103861398 | | 0.995724387 | | | | | -0.446348419 | | | | | 0.634340755 | | | | -0.86188623 | | 0.207618763 |  |  |  |  |
| PtrPPR240 | | | | P | | | 0.22939228 | | -0.615710326 | | | | | -2.183314013 | | | | | 1.534805081 | | | | 0.13811377 | | 0.207618763 |  |  |  |  |
| PtrPPR241 | | | | P | | | 0.366895804 | | 0.799327174 | | | | | -1.183314013 | | | | | 0.771844279 | | | | -0.483374607 | | 0.27800809 |  |  |  |  |
| PtrPPR242 | | | | P | | | NA | | NA | | | | | NA | | | | | NA | | | | NA | | NA |  |  |  |  |
| PtrPPR243 | | | | P | | | 0.591962359 | | 0.483825348 | | | | | -0.498815839 | | | | | 0.914448674 | | | | -0.581778311 | | 0.648191354 |  |  |  |  |
| PtrPPR244 | | | | P | | | -0.151352042 | | -0.820446091 | | | | | -1.46129876 | | | | | 0.121890754 | | | | -0.176396854 | | -0.358871078 |  |  |  |  |
| PtrPPR245 | | | | P | | | -0.735842302 | | -0.378315741 | | | | | -0.8266205 | | | | | -0.138248749 | | | | -0.412083313 | | -0.980008241 |  |  |  |  |
| PtrPPR246 | | | | P | | | -0.121104967 | | 0.176750381 | | | | | -1.010249304 | | | | | -0.493764071 | | | | -0.141994149 | | -0.510610269 |  |  |  |  |
| PtrPPR247 | | | | E+ | | | -3.677498316 | | -0.615710326 | | | | | -1.446348419 | | | | | -0.857512342 | | | | 0.401148176 | | -0.699271833 |  |  |  |  |
| PtrPPR248 | | | | E1 | | | NA | | NA | | | | | NA | | | | | NA | | | | NA | | NA |  |  |  |  |
| PtrPPR249 | | | | P | | | 0.688823899 | | -0.556816636 | | | | | -0.638993497 | | | | | 0.108271943 | | | | 0.597545388 | | -0.129416225 |  |  |  |  |
| PtrPPR250 | | | | E+ | | | 2.036747202 | | 1.236732486 | | | | | 0.138614082 | | | | | 0.786343848 | | | | 0.13811377 | | 0.207618763 |  |  |  |  |
| PtrPPR251 | | | | P | | | NA | | NA | | | | | NA | | | | | NA | | | | NA | | NA |  |  |  |  |
| PtrPPR252 | | | | E+ | | | -1.149119343 | | -0.771829527 | | | | | -0.754470714 | | | | | -0.651061464 | | | | -3.562325948 | | -0.907858455 |  |  |  |  |
| PtrPPR253 | | | | P | | | 0.59862609 | | 0.443183364 | | | | | 0.460542177 | | | | | -0.143266824 | | | | 0.682434286 | | 0.08208788 |  |  |  |  |
| PtrPPR254 | | | | DYW | | | -0.577962642 | | -0.570622436 | | | | | -0.1662405 | | | | | 0.464415753 | | | | -0.084278652 | | -0.014773659 |  |  |  |  |
| PtrPPR255 | | | | P | | | 1.366895804 | | 0.384289674 | | | | | 0.816685987 | | | | | 1.186881778 | | | | 1.401148176 | | 0.885690668 |  |  |  |  |
| PtrPPR256 | | | | P | | | 1.151389768 | | 0.680682677 | | | | | -0.649881813 | | | | | 0.260882359 | | | | 1.13811377 | | 0.483253205 |  |  |  |  |
| PtrPPR257 | | | | P | | | -0.131706412 | | 1.719892706 | | | | | -0.095851172 | | | | | 0.17748308 | | | | -1.236281745 | | 0.44465796 |  |  |  |  |
| PtrPPR258 | | | | P | | | 0.662351687 | | 0.32288913 | | | | | -0.095851172 | | | | | 0.371306349 | | | | 0.208503098 | | 0.27800809 |  |  |  |  |
| PtrPPR259 | | | | PLS | | | -0.371000261 | | 0.81459393 | | | | | -0.46177846 | | | | | 0.018351358 | | | | -1.287192065 | | -1.259507248 |  |  |  |  |
| PtrPPR260 | | | | P | | | 1.313456545 | | 0.56486192 | | | | | -0.045810489 | | | | | 1.61497543 | | | | 1.516625393 | | 0.811690086 |  |  |  |  |
| PtrPPR261 | | | | P | | | NA | | NA | | | | | NA | | | | | NA | | | | NA | | NA |  |  |  |  |
| PtrPPR262 | | | | P | | | 0.06666278 | | 0.514488398 | | | | | -0.147690103 | | | | | -1.272549841 | | | | -1.664440166 | | -0.279732942 |  |  |  |  |
| PtrPPR263 | | | | E2 | | | 2.22939228 | | 1.121255269 | | | | | 1.138614082 | | | | | 0.049378254 | | | | 1.72307627 | | 1.207618763 |  |  |  |  |
| PtrPPR264 | | | | P | | | -0.316176782 | | 1.822846533 | | | | | -0.489417141 | | | | | 0.236396345 | | | | -0.27526068 | | -0.767634575 |  |  |  |  |
| PtrPPR265 | | | | P | | | NA | | NA | | | | | NA | | | | | NA | | | | NA | | NA |  |  |  |  |
| PtrPPR266 | | | | DYW | | | 0.814354781 | | -0.141779137 | | | | | 0.553651581 | | | | | 0.923847372 | | | | 2.411132264 | | 1.08208788 |  |  |  |  |
| PtrPPR267 | | | | P | | | NA | | NA | | | | | NA | | | | | NA | | | | NA | | NA |  |  |  |  |
| PtrPPR268 | | | | P | | | 0.129856606 | | 0.914804391 | | | | | -0.067836796 | | | | | 0.897375161 | | | | -0.309345207 | | 0.54865568 |  |  |  |  |
| PtrPPR269 | | | | PLS | | | 0.059467278 | | 1.121255269 | | | | | -0.223955998 | | | | | 1.201381347 | | | | -0.031811232 | | 0.207618763 |  |  |  |  |
| PtrPPR270 | | | | E1 | | | -1.285180893 | | -0.878744731 | | | | | -2.598351512 | | | | | -0.572110123 | | | | -0.376459403 | | -1.30695441 |  |  |  |  |
| PtrPPR271 | | | | E+ | | | -0.993000141 | | -0.101137153 | | | | | -2.66874084 | | | | | -0.173014167 | | | | -2.669241152 | | -2.59973616 |  |  |  |  |
| PtrPPR272 | | | | E2 | | | NA | | NA | | | | | NA | | | | | NA | | | | NA | | NA |  |  |  |  |
| PtrPPR273 | | | | E2 | | | -0.223119925 | | 0.375011861 | | | | | -0.239897541 | | | | | 0.303134846 | | | | 0.525136893 | | -0.405358114 |  |  |  |  |
| PtrPPR274 | | | | P | | | 0.22939228 | | 1.038793108 | | | | | 0.875579676 | | | | | 0.338884871 | | | | 1.553151269 | | 0.037693761 |  |  |  |  |
| PtrPPR275 | | | | DYW | | | 1.22939228 | | 2.121255269 | | | | | 1.723576582 | | | | | 1.634340755 | | | | 2.460041865 | | 1.207618763 |  |  |  |  |
| PtrPPR276 | | | | P | | | 1.966357874 | | 2.059854724 | | | | | 0.361006503 | | | | | 1.049378254 | | | | 1.945468692 | | 0.207618763 |  |  |  |  |
| PtrPPR277 | | | | P | | | 1.351382804 | | 1.557354383 | | | | | 1.636113741 | | | | | 1.576625257 | | | | 1.22057593 | | 1.249438938 |  |  |  |  |
| PtrPPR278 | | | | P | | | -0.448679625 | | -0.178305013 | | | | | 0.045504677 | | | | | 0.508809873 | | | | 0.661675726 | | -0.622456236 |  |  |  |  |
| PtrPPR279 | | | | DYW | | | 0.22939228 | | 1.121255269 | | | | | -8.016539306 | | | | | -0.950621746 | | | | 1.460041865 | | -0.622456236 |  |  |  |  |
| PtrPPR280 | | | | DYW | | | -0.448679625 | | 1.443183364 | | | | | -0.539457823 | | | | | 1.371306349 | | | | 0.13811377 | | 0.792581263 |  |  |  |  |
| PtrPPR281 | | | | P | | | -0.899890737 | | -0.008027748 | | | | | -0.262748481 | | | | | -0.125708453 | | | | 0.053224872 | | -0.529346832 |  |  |  |  |
| PtrPPR282 | | | | P | | | 0.107401756 | | 0.197204122 | | | | | -0.485876783 | | | | | -0.030792095 | | | | -0.188696547 | | -0.716432384 |  |  |  |  |
| PtrPPR283 | | | | DYW | | | -0.355570221 | | -9.109556322 | | | | | 0.138614082 | | | | | -1.535584247 | | | | 2.13811377 | | 0.207618763 |  |  |  |  |
| PtrPPR284 | | | | P | | | 0.966357874 | | 2.706217769 | | | | | 1.361006503 | | | | | 0.464415753 | | | | 0.553151269 | | 0.622656262 |  |  |  |  |
| PtrPPR285 | | | | P | | | -0.053779771 | | 1.369934381 | | | | | 1.268305158 | | | | | 0.159351096 | | | | -0.411573256 | | 0.304294782 |  |  |  |  |
| PtrPPR286 | | | | E2 | | | 0.376233668 | | -0.516174652 | | | | | -0.498815839 | | | | | -1.298545049 | | | | -1.669241152 | | 0.307154436 |  |  |  |  |
| PtrPPR287 | | | | P | | | 1.22939228 | | 0.121255269 | | | | | 0.91622166 | | | | | 0.563951427 | | | | 0.915721348 | | 0.207618763 |  |  |  |  |
| PtrPPR288 | | | | P | | | 1.451784701 | | 1.536292768 | | | | | 1.361006503 | | | | | 1.049378254 | | | | 2.411132264 | | 1.710119103 |  |  |  |  |
| PtrPPR289 | | | | P | | | 0.446983715 | | 1.087307937 | | | | | -0.828219054 | | | | | -0.469995905 | | | | -1.381260389 | | -0.089362975 |  |  |  |  |
| PtrPPR290 | | | | P | | | 0.845051578 | | 0.881304476 | | | | | 0.393871137 | | | | | 0.361968484 | | | | 0.6917121 | | 0.520208993 |  |  |  |  |
| PtrPPR291 | | | | E+ | | | -0.507573314 | | -2.200672826 | | | | | -2.183314013 | | | | | 0.31241266 | | | | -1.183814325 | | -1.114309332 |  |  |  |  |
| PtrPPR292 | | | | E2 | | | 0.644429779 | | 0.121255269 | | | | | -8.960808875 | | | | | -1.535584247 | | | | 0.553151269 | | -9.219917493 |  |  |  |  |
| PtrPPR293 | | | | P | | | -0.355570221 | | -1.711634746 | | | | | -1.372347838 | | | | | -0.083072042 | | | | 0.005663474 | | -0.81791633 |  |  |  |  |
| PtrPPR294 | | | | DYW | | | -0.639363187 | | 0.268096657 | | | | | -0.581277999 | | | | | -0.320571356 | | | | -0.896651648 | | -1.18469866 |  |  |  |  |
| PtrPPR295 | | | | P | | | NA | | NA | | | | | NA | | | | | NA | | | | NA | | NA |  |  |  |  |
| PtrPPR296 | | | | P | | | NA | | NA | | | | | NA | | | | | NA | | | | NA | | NA |  |  |  |  |
| PtrPPR297 | | | | P | | | 0.381395373 | | 0.410761886 | | | | | -1.03131092 | | | | | 0.201381347 | | | | -1.031811232 | | -0.377343738 |  |  |  |  |
| PtrPPR298 | | | | DWY | | | 0.929831998 | | 0.706217769 | | | | | -0.276423418 | | | | | 1.136841095 | | | | 0.460041865 | | 0.908058481 |  |  |  |  |
| PtrPPR299 | | | | E2 | | | 3.399317281 | | 3.121255269 | | | | | -7.346806702 | | | | | 3.508809873 | | | | 2.460041865 | | 1.207618763 |  |  |  |  |
| PtrPPR300 | | | | E+ | | | -0.858070561 | | -0.796282571 | | | | | -0.626920665 | | | | | -0.337644869 | | | | -0.949349072 | | -0.557915984 |  |  |  |  |
| PtrPPR301 | | | | P | | | 0.688823899 | | 0.121255269 | | | | | 0.460542177 | | | | | -0.143266824 | | | | 0.308038771 | | -0.792381237 |  |  |  |  |
| PtrPPR302 | | | | P | | | NA | | NA | | | | | NA | | | | | NA | | | | NA | | NA |  |  |  |  |
| PtrPPR303 | | | | DYW | | | 1.22939228 | | 2.121255269 | | | | | 1.138614082 | | | | | 2.371306349 | | | | 2.945468692 | | 2.529546857 |  |  |  |  |
| PtrPPR304 | | | | E+ | | | 0.006999859 | | 0.121255269 | | | | | 0.653187255 | | | | | 1.634340755 | | | | 0.790190466 | | 0.985226341 |  |  |  |  |
| PtrPPR305 | | | | P | | | 0.644429779 | | 1.34364769 | | | | | 0.138614082 | | | | | 1.464415753 | | | | 2.012582888 | | 0.944584357 |  |  |  |  |
| PtrPPR306 | | | | P | | | -0.789973045 | | 0.846632889 | | | | | -0.183314013 | | | | | 0.263503059 | | | | -1.283349999 | | -0.722969427 |  |  |  |  |
| PtrPPR307 | | | | P | | | 0.381395373 | | 1.951330267 | | | | | 1.056151922 | | | | | 1.201381347 | | | | 0.13811377 | | 0.173776611 |  |  |  |  |
| PtrPPR308 | | | | E+ | | | -1.77060772 | | 0.443183364 | | | | | -0.861385918 | | | | | 1.219303255 | | | | -0.86188623 | | -1.563188983 |  |  |  |  |
| PtrPPR309 | | | | E+ | | | -0.422684417 | | 0.362263368 | | | | | -0.150892535 | | | | | 0.589946635 | | | | 0.678682151 | | -0.340796562 |  |  |  |  |
| PtrPPR310 | | | | PLS | | | NA | | NA | | | | | NA | | | | | NA | | | | NA | | NA |  |  |  |  |
| PtrPPR311 | | | | P | | | -0.791071823 | | 0.890371386 | | | | | 0.558722571 | | | | | -0.169631528 | | | | -0.178743336 | | -1.018868467 |  |  |  |  |
| PtrPPR312 | | | | P | | | 0.22939228 | | 1.29118027 | | | | | -0.276423418 | | | | | 1.371306349 | | | | 0.460041865 | | 1.021773517 |  |  |  |  |
| PtrPPR313 | | | | E2 | | | 1.22939228 | | 1.706217769 | | | | | 1.138614082 | | | | | 0.634340755 | | | | 1.72307627 | | 0.021773517 |  |  |  |  |
| PtrPPR314 | | | | P | | | NA | | NA | | | | | NA | | | | | NA | | | | NA | | NA |  |  |  |  |
| PtrPPR315 | | | | PLS | | | NA | | NA | | | | | NA | | | | | NA | | | | NA | | NA |  |  |  |  |
| PtrPPR316 | | | | E2 | | | 0.036747202 | | 0.443183364 | | | | | -0.276423418 | | | | | 1.297305767 | | | | -2.86188623 | | 0.507200345 |  |  |  |  |
| PtrPPR317 | | | | P | | | -0.394044369 | | 0.016285709 | | | | | -0.372347838 | | | | | -0.461583665 | | | | -0.787885649 | | 0.324336287 |  |  |  |  |
| PtrPPR318 | | | | P | | | 0.631490724 | | 0.483825348 | | | | | -0.66874084 | | | | | 0.148913928 | | | | 0.188739843 | | 0.373245888 |  |  |  |  |
| PtrPPR319 | | | | P | | | 9.264351957 | | NA | | | | | 8.914465141 | | | | | NA | | | | 9.173073446 | | 0.021773517 |  |  |  |  |
| PtrPPR320 | | | | E+ | | | 1.528952562 | | 1.880247169 | | | | | 0.608099365 | | | | | 1.518863537 | | | | -0.10289433 | | -0.300154577 |  |  |  |  |
| PtrPPR321 | | | | P | | | NA | | NA | | | | | NA | | | | | NA | | | | NA | | NA |  |  |  |  |
| PtrPPR322 | | | | PLS | | | NA | | 7.559778139 | | | | | NA | | | | | 8.487901125 | | | | 7.835745258 | | NA |  |  |  |  |
| PtrPPR323 | | | | E+ | | | 0.22939228 | | 1.121255269 | | | | | 0.428120699 | | | | | 1.049378254 | | | | 0.553151269 | | 0.606736018 |  |  |  |  |
| PtrPPR324 | | | | P | | | -9.183058316 | | -8.923949698 | | | | | 0.138614082 | | | | | 0.049378254 | | | | -9.183058316 | | NA |  |  |  |  |
| PtrPPR325 | | | | P | | | 1.22939228 | | 1.121255269 | | | | | 0.138614082 | | | | | 0.049378254 | | | | 0.401148176 | | 0.53634669 |  |  |  |  |
| PtrPPR326 | | | | P | | | -0.050715639 | | 0.203717429 | | | | | 1.138614082 | | | | | -2.453122086 | | | | 1.694507118 | | 0.829128439 |  |  |  |  |
| PtrPPR327 | | | | P | | | NA | | NA | | | | | NA | | | | | NA | | | | NA | | NA |  |  |  |  |
| PtrPPR328 | | | | PLS | | | NA | | NA | | | | | NA | | | | | NA | | | | NA | | -8.762187202 |  |  |  |  |
| PtrPPR329 | | | | P | | | NA | | 8.795226714 | | | | | 8.812585527 | | | | | 9.723349699 | | | | 9.071193832 | | NA |  |  |  |  |
| PtrPPR330 | | | | P | | | NA | | NA | | | | | NA | | | | | NA | | | | NA | | NA |  |  |  |  |
| PtrPPR331 | | | | E2 | | | -1.092535815 | | 0.606682096 | | | | | -0.183314013 | | | | | -0.68758734 | | | | -2.183814325 | | -0.563188983 |  |  |  |  |
| PtrPPR332 | | | | P | | | NA | | NA | | | | | NA | | | | | NA | | | | NA | | NA |  |  |  |  |
| PtrPPR333 | | | | P | | | 2.036747202 | | 2.513572691 | | | | | 0.460542177 | | | | | 2.371306349 | | | | 2.225576611 | | 0.659203438 |  |  |  |  |
| PtrPPR334 | | | | P | | | 9.123653094 | | 8.756407465 | | | | | NA | | | | | 8.68453045 | | | | 9.032374583 | | 0.021773517 |  |  |  |  |
| PtrPPR335 | | | | E1 | | | 1.814354781 | | 0.121255269 | | | | | 1.723576582 | | | | | 2.508809873 | | | | 1.945468692 | | 0.021773517 |  |  |  |  |
| PtrPPR336 | | | | DYW | | | -1.645076838 | | -0.530821428 | | | | | -0.150892535 | | | | | -0.410053365 | | | | -0.151392847 | | 0.606736018 |  |  |  |  |
| PtrPPR337 | | | | E2 | | | -0.77060772 | | 0.121255269 | | | | | -0.276423418 | | | | | 0.856733176 | | | | 0.460041865 | | -1.785581405 |  |  |  |  |
| PtrPPR338 | | | | P | | | 1.551320375 | | 2.793680611 | | | | | 0.945969004 | | | | | 2.371306349 | | | | 2.045004365 | | 0.256238771 |  |  |  |  |
| PtrPPR339 | | | | E2 | | | 1.644429779 | | 1.34364769 | | | | | 0.138614082 | | | | | 1.634340755 | | | | 0.553151269 | | 1.436811017 |  |  |  |  |
| PtrPPR340 | | | | DYW | | | -0.236271292 | | 0.217470584 | | | | | -0.195804957 | | | | | 0.617662014 | | | | 0.234329085 | | -0.22976525 |  |  |  |  |
| PtrPPR341 | | | | P | | | 0.406270042 | | 0.597693313 | | | | | -0.577592952 | | | | | 1.017669394 | | | | 1.658945933 | | 0.021773517 |  |  |  |  |
| PtrPPR342 | | | | DYW | | | -0.01161582 | | 0.508278392 | | | | | -0.654935041 | | | | | 0.903527388 | | | | -0.392400947 | | 0.896242635 |  |  |  |  |
| PtrPPR343 | | | | PLS | | | NA | | NA | | | | | NA | | | | | NA | | | | NA | | NA |  |  |  |  |
| PtrPPR344 | | | | P | | | NA | | NA | | | | | NA | | | | | NA | | | | NA | | NA |  |  |  |  |
| PtrPPR345 | | | | P | | | 0.280018353 | | 1.121255269 | | | | | -0.346812745 | | | | | -0.365659245 | | | | -0.145679196 | | -0.51256291 |  |  |  |  |
| PtrPPR346 | | | | E2 | | | -0.185645219 | | -0.141779137 | | | | | -3.446348419 | | | | | 0.786343848 | | | | -0.639493809 | | -0.393263982 |  |  |  |  |
| PtrPPR347 | | | | P | | | 1.551320375 | | 0.29118027 | | | | | 0.138614082 | | | | | 1.297305767 | | | | 1.597545388 | | 1.021773517 |  |  |  |  |
| PtrPPR348 | | | | P | | | -1.600682719 | | -0.630817218 | | | | | -0.539457823 | | | | | -0.628693651 | | | | 0.838553488 | | -1.200618904 |  |  |  |  |
| PtrPPR349 | | | | P | | | NA | | NA | | | | | NA | | | | | 8.793902979 | | | | 8.141747112 | | -8.211252105 |  |  |  |  |
| PtrPPR350 | | | | P | | | 0.167331312 | | 1.067099499 | | | | | 1.003386701 | | | | | 0.410259983 | | | | 0.718005281 | | 0.207640063 |  |  |  |  |
| PtrPPR351 | | | | P | | | -0.173963414 | | 0.318292116 | | | | | -0.411583001 | | | | | -0.220710909 | | | | 0.304123721 | | 0.516538209 |  |  |  |  |
| PtrPPR352 | | | | P | | | 0.14693012 | | -0.048669733 | | | | | -0.571879301 | | | | | -0.420107029 | | | | -1.22445631 | | 0.524273858 |  |  |  |  |
| PtrPPR353 | | | | P | | | -0.208728832 | | -0.686099653 | | | | | -1.931775246 | | | | | -0.757976668 | | | | -0.827938898 | | 1.583652405 |  |  |  |  |
| PtrPPR354 | | | | P | | | -1.577962642 | | -1.101137153 | | | | | -0.66874084 | | | | | -0.757976668 | | | | -0.084278652 | | 1.021773517 |  |  |  |  |
| PtrPPR355 | | | | P | | | -0.77060772 | | -0.878744731 | | | | | -0.446348419 | | | | | -0.535584247 | | | | -1.446848731 | | 10.81454904 |  |  |  |  |
| PtrPPR356 | | | | E+ | | | -1.092535815 | | -1.200672826 | | | | | -2.183314013 | | | | | -9.37211779 | | | | -2.183814325 | | 0.021773517 |  |  |  |  |
| PtrPPR357 | | | | E2 | | | -1.355570221 | | 0.121255269 | | | | | 0.138614082 | | | | | 1.049378254 | | | | 0.13811377 | | -1.978226483 |  |  |  |  |
| PtrPPR358 | | | | P | | | -0.070168002 | | 0.606682096 | | | | | -0.138919894 | | | | | -0.341812503 | | | | -0.183814325 | | -0.064383126 |  |  |  |  |
| PtrPPR359 | | | | E1 | | | NA | | NA | | | | | NA | | | | | NA | | | | NA | | NA |  |  |  |  |
| PtrPPR360 | | | | P | | | 0.446203669 | | -1.000735256 | | | | | -1.163948688 | | | | | -0.030792095 | | | | -0.427483406 | | 0.403644153 |  |  |  |  |
| PtrPPR361 | | | | P | | | 1.22939228 | | 1.258758792 | | | | | 0.138614082 | | | | | 1.727450159 | | | | 1.275617293 | | 1.758739112 |  |  |  |  |
| PtrPPR362 | | | | P | | | -0.067589458 | | 0.18684361 | | | | | -0.764088717 | | | | | 0.378001002 | | | | -0.002748766 | | 0.566094034 |  |  |  |  |
| PtrPPR363 | | | | E+ | | | NA | | NA | | | | | NA | | | | | NA | | | | NA | | NA |  |  |  |  |
| PtrPPR364 | | | | E+ | | | -0.993000141 | | -0.364171559 | | | | | -0.08377834 | | | | | 1.148913928 | | | | 0.13811377 | | 0.606736018 |  |  |  |  |
| PtrPPR365 | | | | E2 | | | 0.036747202 | | 0.928610191 | | | | | 0.045504677 | | | | | 0.804265756 | | | | 0.13811377 | | 0.659203438 |  |  |  |  |
| PtrPPR366 | | | | P | | | -0.092535815 | | -0.326203708 | | | | | 0.039078408 | | | | | 0.786343848 | | | | -1.768776826 | | 0.436811017 |  |  |  |  |
| PtrPPR367 | | | | P | | | 11.60754867 | | NA | | | | | 9.935733757 | | | | | 9.84649793 | | | | 10.77930456 | | 0.021773517 |  |  |  |  |
| PtrPPR368 | | | | P | | | -0.218066697 | | 0.021719595 | | | | | -0.960921592 | | | | | -0.272549841 | | | | 0.231223174 | | 0.311280135 |  |  |  |  |
| PtrPPR369 | | | | E+ | | | -0.185645219 | | 0.706217769 | | | | | -9.149269469 | | | | | 0.049378254 | | | | 2.225576611 | | -0.393263982 |  |  |  |  |
| PtrPPR370 | | | | P | | | -0.012682507 | | -0.890521899 | | | | | -1.312047327 | | | | | 0.129231562 | | | | -0.049513233 | | 0.552288234 |  |  |  |  |
| PtrPPR371 | | | | P | | | 0.921269985 | | 1.57056267 | | | | | 0.392370674 | | | | | 1.577757226 | | | | 1.546198508 | | 0.244165939 |  |  |  |  |
| PtrPPR372 | | | | DYW | | | -1.033642126 | | -0.463707232 | | | | | -1.124420324 | | | | | -0.728229325 | | | | -1.124920636 | | -0.46365331 |  |  |  |  |
| PtrPPR373 | | | | P | | | 0.607903903 | | 0.384289674 | | | | | -0.183314013 | | | | | 0.186881778 | | | | 0.623540597 | | 0.722213236 |  |  |  |  |
| PtrPPR374 | | | | P | | | 2.551320375 | | 2.706217769 | | | | | 1.138614082 | | | | | 3.371306349 | | | | 2.72307627 | | 0.343701612 |  |  |  |  |
| PtrPPR375 | | | | P | | | -0.355570221 | | 0.858220863 | | | | | -1.124420324 | | | | | 1.464415753 | | | | 0.801078782 | | 0.021773517 |  |  |  |  |
| PtrPPR376 | | | | DYW | | | -0.548215299 | | 0.928610191 | | | | | -0.861385918 | | | | | 0.634340755 | | | | -0.446848731 | | 0.829128439 |  |  |  |  |
| PtrPPR377 | | | | DYW | | | 0.22939228 | | 0.313900347 | | | | | -1.66874084 | | | | | 1.049378254 | | | | 0.500683849 | | 1.244165939 |  |  |  |  |
| PtrPPR378 | | | | P | | | 0.591962359 | | -1.101137153 | | | | | -0.346812745 | | | | | -0.436048573 | | | | 0.500683849 | | 0.191698519 |  |  |  |  |
| PtrPPR379 | | | | P | | | 0.022941402 | | -0.085195609 | | | | | -0.223955998 | | | | | 0.390415172 | | | | -1.183814325 | | 0.722213236 |  |  |  |  |
| PtrPPR380 | | | | P | | | 0.400292405 | | -0.12374885 | | | | | 0.195985261 | | | | | -0.066342847 | | | | -1.347898448 | | 0.237250671 |  |  |  |  |
| PtrPPR381 | | | | P | | | 0.583029235 | | 0.273258362 | | | | | -0.223955998 | | | | | 0.523309442 | | | | 0.491750724 | | 0.457872632 |  |  |  |  |
| PtrPPR382 | | | | DYW | | | -0.185645219 | | 1.513572691 | | | | | 0.308539083 | | | | | 1.57294021 | | | | 0.597545388 | | -0.715192077 |  |  |  |  |
| PtrPPR383 | | | | P | | | 1.344869497 | | 0.706217769 | | | | | 0.361006503 | | | | | 0.987977709 | | | | 0.553151269 | | 0.021773517 |  |  |  |  |
| PtrPPR384 | | | | P | | | -0.355570221 | | 0.536292768 | | | | | 0.553651581 | | | | | 0.049378254 | | | | 1.360506191 | | -0.563188983 |  |  |  |  |
| PtrPPR385 | | | | P | | | -0.448679625 | | -0.556816636 | | | | | -1.276423418 | | | | | -0.950621746 | | | | 0.597545388 | | 0.021773517 |  |  |  |  |
| PtrPPR386 | | | | P | | | -0.31012725 | | -0.096336166 | | | | | -1.16895872 | | | | | -0.27978141 | | | | -0.127230797 | | 0.038647336 |  |  |  |  |
| PtrPPR387 | | | | P | | | 0.22939228 | | -8.326048142 | | | | | 1.138614082 | | | | | -8.326048142 | | | | -8.585156759 | | 8.814549039 |  |  |  |  |
| PtrPPR388 | | | | P | | | -0.443313115 | | -0.428301896 | | | | | -0.963484106 | | | | | -0.327342314 | | | | -0.359913094 | | -0.061481986 |  |  |  |  |
| PtrPPR389 | | | | PLS | | | NA | | NA | | | | | NA | | | | | NA | | | | NA | | NA |  |  |  |  |
| PtrPPR390 | | | | E2 | | | 0.644429779 | | 1.706217769 | | | | | 0.875579676 | | | | | 1.634340755 | | | | 2.253590987 | | 1.021773517 |  |  |  |  |
| PtrPPR391 | | | | DYW | | | -1.185645219 | | -0.071389809 | | | | | -0.9869168 | | | | | -0.535584247 | | | | -0.86188623 | | 0.021773517 |  |  |  |  |
| PtrPPR392 | | | | P | | | -0.218066697 | | 0.145102011 | | | | | -0.680813673 | | | | | -0.129591887 | | | | -0.27692373 | | 0.673850214 |  |  |  |  |
| PtrPPR393 | | | | P | | | -0.01161582 | | -0.119752831 | | | | | -0.754470714 | | | | | -1.191629845 | | | | 0.13811377 | | 0.233277623 |  |  |  |  |
| PtrPPR394 | | | | P | | | NA | | 1.20871811 | | | | | 0.945969004 | | | | | 0.749817972 | | | | NA | | NA |  |  |  |  |
| PtrPPR395 | | | | E+ | | | 1.814354781 | | 2.121255269 | | | | | 0.138614082 | | | | | 3.297305767 | | | | 0.72307627 | | 0.606736018 |  |  |  |  |
| PtrPPR396 | | | | P | | | -0.067993631 | | 0.525645524 | | | | | 0.080898584 | | | | | -0.375119574 | | | | 0.050650928 | | 0.247065829 |  |  |  |  |
| PtrPPR397 | | | | P | | | 0.22939228 | | 2.121255269 | | | | | -7.557485702 | | | | | -7.557485702 | | | | -7.81659432 | | 0.021773517 |  |  |  |  |
| PtrPPR398 | | | | P | | | 1.136282876 | | 1.121255269 | | | | | 0.308539083 | | | | | 1.371306349 | | | | 1.72307627 | | 0.469232494 |  |  |  |  |
| PtrPPR399 | | | | P | | | -0.812427896 | | -2.686099653 | | | | | -1.66874084 | | | | | -0.556342807 | | | | -0.531737629 | | 0.787308264 |  |  |  |  |
| PtrPPR400 | | | | P | | | -0.564156843 | | 0.121255269 | | | | | -0.754470714 | | | | | -0.843706542 | | | | -0.754971026 | | 0.469232494 |  |  |  |  |
| PtrPPR401 | | | | PLS | | | -0.77060772 | | -0.293782231 | | | | | -3.446348419 | | | | | 0.108271943 | | | | -0.86188623 | | 0.021773517 |  |  |  |  |
| PtrPPR402 | | | | PLS | | | -0.577962642 | | -0.364171559 | | | | | -0.66874084 | | | | | -0.436048573 | | | | 0.652686943 | | -0.300154577 |  |  |  |  |
| PtrPPR403 | | | | P | | | -0.144066116 | | -1.389706651 | | | | | -2.372347838 | | | | | 0.406312799 | | | | -0.109813744 | | 0.481205136 |  |  |  |  |
| PtrPPR404 | | | | P | | | NA | | NA | | | | | NA | | | | | NA | | | | 8.713002193 | | NA |  |  |  |  |
| PtrPPR405 | | | | DYW | | | 1.22939228 | | 1.580686887 | | | | | -0.861385918 | | | | | 1.219303255 | | | | 1.945468692 | | 1.021773517 |  |  |  |  |
| PtrPPR406 | | | | P | | | 1.814354781 | | 1.443183364 | | | | | 1.460542177 | | | | | 2.219303255 | | | | 2.13811377 | | 0.284807923 |  |  |  |  |
| PtrPPR407 | | | | P | | | NA | | NA | | | | | NA | | | | | NA | | | | NA | | NA |  |  |  |  |
| PtrPPR408 | | | | P | | | -0.105026759 | | 0.070629196 | | | | | -0.4714394 | | | | | -0.7211399 | | | | -1.134904725 | | 1.085903855 |  |  |  |  |
| PtrPPR409 | | | | P | | | 1.714819107 | | 1.384289674 | | | | | 0.624040909 | | | | | 1.634340755 | | | | 0.986110676 | | 1.021773517 |  |  |  |  |
| PtrPPR410 | | | | P | | | -0.325196572 | | -0.625978661 | | | | | -0.831012269 | | | | | -1.505210598 | | | | -0.416475082 | | 1.436811017 |  |  |  |  |
| PtrPPR411 | | | | P | | | -1.355570221 | | -3.048669733 | | | | | -2.03131092 | | | | | 0.049378254 | | | | -0.446848731 | | -0.715192077 |  |  |  |  |
| PtrPPR412 | | | | P | | | 0.850880657 | | 0.42081555 | | | | | 0.138614082 | | | | | 0.670866631 | | | | 1.245028974 | | 0.343701612 |  |  |  |  |
| PtrPPR413 | | | | P | | | -0.033642126 | | 0.315902699 | | | | | -0.183314013 | | | | | -0.535584247 | | | | -0.709883137 | | 1.041138842 |  |  |  |  |
| PtrPPR414 | | | | E+ | | | 0.22939228 | | -0.463707232 | | | | | -0.446348419 | | | | | 0.464415753 | | | | 0.875079364 | | -1.200618904 |  |  |  |  |
| PtrPPR415 | | | | P | | | 0.271212456 | | -0.411618721 | | | | | -0.994652449 | | | | | 0.64841594 | | | | 0.558445569 | | 0.173776611 |  |  |  |  |
| PtrPPR416 | | | | E+ | | | -0.507573314 | | 0.384289674 | | | | | -0.375959091 | | | | | 0.049378254 | | | | -0.598851824 | | 0.284807923 |  |  |  |  |
| PtrPPR417 | | | | P | | | 1.22939228 | | 1.258758792 | | | | | 1.460542177 | | | | | 1.482337661 | | | | 1.623540597 | | 0.436811017 |  |  |  |  |
| PtrPPR418 | | | | DWY | | | -2.77060772 | | 0.443183364 | | | | | -1.276423418 | | | | | -1.365659245 | | | | -0.054531308 | | -1.978226483 |  |  |  |  |
| PtrPPR419 | | | | PLS | | | NA | | NA | | | | | NA | | | | | NA | | | | NA | | NA |  |  |  |  |
| PtrPPR420 | | | | DYW | | | 0.551320375 | | 1.121255269 | | | | | 0.138614082 | | | | | 1.219303255 | | | | 0.460041865 | | 0.021773517 |  |  |  |  |
| PtrPPR421 | | | | P | | | 1.22939228 | | 1.706217769 | | | | | 0.138614082 | | | | | 1.634340755 | | | | 0.72307627 | | -0.300154577 |  |  |  |  |
| PtrPPR422 | | | | P | | | NA | | NA | | | | | NA | | | | | NA | | | | NA | | NA |  |  |  |  |
| PtrPPR423 | | | | E+ | | | -0.149119343 | | -0.771829527 | | | | | -0.561825636 | | | | | -1.066098963 | | | | -1.240397854 | | 0.095774099 |  |  |  |  |
| PtrPPR424 | | | | E1 | | | -0.355570221 | | 0.536292768 | | | | | 0.138614082 | | | | | -0.535584247 | | | | 2.012582888 | | -1.563188983 |  |  |  |  |
| PtrPPR425 | | | | P | | | -0.159010093 | | 1.327556791 | | | | | 0.012268348 | | | | | 0.355085996 | | | | -0.048144866 | | 0.106307028 |  |  |  |  |
| PtrPPR426 | | | | E+ | | | 0.288285969 | | 1.34364769 | | | | | -0.446348419 | | | | | 1.192336208 | | | | 1.045004365 | | -0.034810011 |  |  |  |  |
| PtrPPR427 | | | | DYW | | | -0.256034547 | | 1.483825348 | | | | | 0.790690778 | | | | | 0.94246305 | | | | 0.500683849 | | 1.343701612 |  |  |  |  |
| PtrPPR428 | | | | E+ | | | 0.929831998 | | 0.706217769 | | | | | -0.276423418 | | | | | 1.297305767 | | | | 0.838553488 | | 0.552288234 |  |  |  |  |
| PtrPPR429 | | | | DYW | | | -9.694293203 | | -0.200672826 | | | | | -0.183314013 | | | | | -0.68758734 | | | | -2.183814325 | | -7.579983871 |  |  |  |  |
| PtrPPR430 | | | | P | | | 10.19967096 | | 12.83242533 | | | | | 12.11281855 | | | | | 13.29660122 | | | | 9.786464355 | | -3.356738106 |  |  |  |  |
| PtrPPR431 | | | | P | | | 2.155391698 | | 4.225591928 | | | | | 3.026139353 | | | | | 4.327363001 | | | | 1.401148176 | | -2.052227064 |  |  |  |  |
| PtrPPR432 | | | | E+ | | | -0.870143394 | | 0.021719595 | | | | | -0.308844895 | | | | | -1.050157419 | | | | -0.068337108 | | 0.244165939 |  |  |  |  |
| PtrPPR433 | | | | E2 | | | 9.033938972 | | 8.666693343 | | | | | 9.684052156 | | | | | 9.916744424 | | | | 8.942660462 | | 0.021773517 |  |  |  |  |
| PtrPPR434 | | | | P | | | NA | | 9.756407465 | | | | | 9.188803777 | | | | | 10.09956795 | | | | 10.76934018 | | -10.83884517 |  |  |  |  |
| PtrPPR435 | | | | P | | | 1.688823899 | | 2.165649388 | | | | | 0.460542177 | | | | | 1.219303255 | | | | 1.225576611 | | 0.481205136 |  |  |  |  |
| PtrPPR436 | | | | DYW | | | 2.22939228 | | 2.706217769 | | | | | 1.138614082 | | | | | 3.049378254 | | | | 0.13811377 | | 0.436811017 |  |  |  |  |
| PtrPPR437 | | | | E+ | | | 1.103861398 | | 0.706217769 | | | | | -0.446348419 | | | | | 1.271770675 | | | | 2.460041865 | | 0.481205136 |  |  |  |  |
| PtrPPR438 | | | | E+ | | | 7.476226506 | | 8.108980877 | | | | | 7.12633969 | | | | | NA | | | | NA | | 7.476226506 |  |  |  |  |
| PtrPPR439 | | | | DWY | | | 8.910409055 | | NA | | | | | NA | | | | | NA | | | | NA | | 8.910409055 |  |  |  |  |
| PtrPPR440 | | | | E+ | | | -0.908111244 | | 0.749286491 | | | | | -0.735855036 | | | | | 0.837874149 | | | | -0.321317849 | | -0.978226483 |  |  |  |  |
| PtrPPR441 | | | | P | | | 0.22939228 | | -0.463707232 | | | | | 0.0130832 | | | | | 0.464415753 | | | | -0.124920636 | | 0.021773517 |  |  |  |  |
| PtrPPR442 | | | | P | | | 1.814354781 | | 1.121255269 | | | | | 1.723576582 | | | | | 1.634340755 | | | | -0.86188623 | | 0.284807923 |  |  |  |  |
| PtrPPR443 | | | | P | | | 1.814354781 | | 1.706217769 | | | | | 2.723576582 | | | | | 1.634340755 | | | | 1.72307627 | | 1.021773517 |  |  |  |  |
| PtrPPR444 | | | | P | | | 0.077389186 | | -2.200672826 | | | | | -0.598351512 | | | | | -0.950621746 | | | | -0.598851824 | | -0.130229576 |  |  |  |  |
| PtrPPR445 | | | | PLS | | | NA | | NA | | | | | NA | | | | | NA | | | | NA | | NA |  |  |  |  |
| PtrPPR446 | | | | DYW | | | 0.22939228 | | 1.294903356 | | | | | 0.235911283 | | | | | -0.376886501 | | | | -0.243756866 | | 0.69315077 |  |  |  |  |
| PtrPPR447 | | | | P | | | 0.271212456 | | 0.583256247 | | | | | -0.522584005 | | | | | -0.31926634 | | | | -1.267525032 | | 0.284807923 |  |  |  |  |
| PtrPPR448 | | | | P | | | 1.399317281 | | 0.121255269 | | | | | -0.861385918 | | | | | 1.219303255 | | | | -0.27692373 | | -0.2677331 |  |  |  |  |
| PtrPPR449 | | | | E2 | | | 0.907464185 | | -0.200672826 | | | | | 0.138614082 | | | | | 1.186881778 | | | | 0.816185675 | | 1.436811017 |  |  |  |  |
| PtrPPR450 | | | | DYW | | | 0.122477076 | | -0.226668035 | | | | | -1.209309222 | | | | | -0.851086072 | | | | -0.581778311 | | 1.137250735 |  |  |  |  |
| PtrPPR451 | | | | E+ | | | -0.070168002 | | -0.178305013 | | | | | -1.276423418 | | | | | -0.365659245 | | | | 0.045004365 | | 0.262781617 |  |  |  |  |
| PtrPPR452 | | | | E+ | | | 0.966357874 | | 1.34364769 | | | | | 0.553651581 | | | | | 1.049378254 | | | | 0.13811377 | | 0.343701612 |  |  |  |  |
| PtrPPR453 | | | | DYW | | | 0.059467278 | | 0.273258362 | | | | | -2.03131092 | | | | | 0.049378254 | | | | -2.031811232 | | 0.436811017 |  |  |  |  |
| PtrPPR454 | | | | E+ | | | -1.462485425 | | 1.121255269 | | | | | -0.794271722 | | | | | -0.588051667 | | | | -2.084278652 | | 0.262781617 |  |  |  |  |
| PtrPPR455 | | | | P | | | NA | | NA | | | | | NA | | | | | NA | | | | NA | | NA |  |  |  |  |
| PtrPPR456 | | | | P | | | NA | | NA | | | | | NA | | | | | NA | | | | NA | | NA |  |  |  |  |
| PtrPPR457 | | | | P | | | 9.278496139 | | NA | | | | | NA | | | | | NA | | | | NA | | 9.278496139 |  |  |  |  |
| PtrPPR458 | | | | P | | | 0.22939228 | | 2.443183364 | | | | | 2.308539083 | | | | | 1.856733176 | | | | 1.13811377 | | -1.563188983 |  |  |  |  |
| PtrPPR459 | | | | DWY | | | 1.814354781 | | -0.463707232 | | | | | 0.553651581 | | | | | 0.786343848 | | | | 1.553151269 | | 1.191698519 |  |  |  |  |
| PtrPPR460 | | | | E1 | | | 2.399317281 | | -0.878744731 | | | | | 1.138614082 | | | | | -0.950621746 | | | | 1.460041865 | | 10.28403432 |  |  |  |  |
| PtrPPR461 | | | | PLS | | | NA | | 8.372015283 | | | | | NA | | | | | 8.300138268 | | | | NA | | NA |  |  |  |  |
| PtrPPR462 | | | | P | | | 1.22939228 | | 0.858220863 | | | | | 0.138614082 | | | | | 0.464415753 | | | | 0.553151269 | | 1.606736018 |  |  |  |  |
| PtrPPR463 | | | | P | | | -0.392096097 | | -0.500233108 | | | | | -4.183314013 | | | | | 0.049378254 | | | | 0.571073177 | | 0.400285141 |  |  |  |  |
| PtrPPR464 | | | | P | | | -11.12947728 | | -10.87036866 | | | | | -10.87036866 | | | | | -0.535584247 | | | | -1.446848731 | | NA |  |  |  |  |
| PtrPPR465 | | | | P | | | -0.600682719 | | -0.70881973 | | | | | -2.276423418 | | | | | -0.780696745 | | | | -1.539958135 | | -0.130229576 |  |  |  |  |
| PtrPPR466 | | | | P | | | -0.380661202 | | -0.929370804 | | | | | -1.549441912 | | | | | -0.7211399 | | | | -0.719867225 | | 0.569261313 |  |  |  |  |
| PtrPPR467 | | | | P | | | 0.91528369 | | 0.182655813 | | | | | 0.200014626 | | | | | 0.883368303 | | | | 1.447441828 | | 0.143764042 |  |  |  |  |
| PtrPPR468 | | | | E2 | | | -1.77060772 | | 0.121255269 | | | | | 0.723576582 | | | | | 0.371306349 | | | | 0.945468692 | | -0.978226483 |  |  |  |  |
| PtrPPR469 | | | | P | | | 0.759906997 | | 0.410761886 | | | | | -0.03131092 | | | | | -0.535584247 | | | | 0.427620387 | | 1.722213236 |  |  |  |  |
| PtrPPR470 | | | | P | | | NA | | 1.455674308 | | | | | 0.615052126 | | | | | 0.33317122 | | | | NA | | NA |  |  |  |  |
| PtrPPR471 | | | | DYW | | | 0.907464185 | | 1.706217769 | | | | | 0.624040909 | | | | | 1.049378254 | | | | 1.903648516 | | 0.214418595 |  |  |  |  |
| PtrPPR472 | | | | DYW | | | 0.470400379 | | 0.469178572 | | | | | -0.150892535 | | | | | 0.290386354 | | | | -0.321317849 | | 0.722213236 |  |  |  |  |
| PtrPPR473 | | | | E2 | | | 1.145868924 | | -0.808355403 | | | | | -1.229117703 | | | | | -0.572110123 | | | | 0.173737679 | | 1.282898333 |  |  |  |  |
| PtrPPR474 | | | | P | | | 1.714819107 | | 1.606682096 | | | | | 1.816685987 | | | | | 1.814913 | | | | 2.275617293 | | -0.258334402 |  |  |  |  |
| PtrPPR475 | | | | E+ | | | -0.092535815 | | 0.706217769 | | | | | -0.861385918 | | | | | -0.465194919 | | | | -0.86188623 | | 0.436811017 |  |  |  |  |
| PtrPPR476 | | | | P | | | 0.266590898 | | 0.839800924 | | | | | -0.679547595 | | | | | 0.471129097 | | | | -0.468543802 | | -0.098940348 |  |  |  |  |
| PtrPPR477 | | | | P | | | 0.399317281 | | 0.928610191 | | | | | 0.460542177 | | | | | 1.297305767 | | | | 0.460041865 | | 0.384343597 |  |  |  |  |
| PtrPPR478 | | | | P | | | 0.307394792 | | 0.858220863 | | | | | -0.223955998 | | | | | 0.686808175 | | | | 0.427620387 | | -0.122616392 |  |  |  |  |
| PtrPPR479 | | | | E2 | | | 1.022941402 | | 1.443183364 | | | | | 0.039078408 | | | | | 1.31241266 | | | | 0.44067654 | | 0.295948481 |  |  |  |  |
| PtrPPR480 | | | | P | | | -0.092535815 | | 0.56013951 | | | | | -1.183314013 | | | | | -0.420107029 | | | | -0.86188623 | | 0.358808505 |  |  |  |  |
| PtrPPR481 | | | | E2 | | | 1.551320375 | | 1.121255269 | | | | | 1.138614082 | | | | | 0.049378254 | | | | 1.13811377 | | 0.758739112 |  |  |  |  |
| PtrPPR482 | | | | P | | | -0.732133572 | | -0.043803978 | | | | | -0.263484362 | | | | | 0.594812391 | | | | -0.026945477 | | 0.569261313 |  |  |  |  |
| PtrPPR483 | | | | DYW | | | 1.122477076 | | 1.261432927 | | | | | 0.078493089 | | | | | 0.292649405 | | | | -0.084278652 | | 1.320114792 |  |  |  |  |
| PtrPPR484 | | | | P | | | 0.413816851 | | 0.519804645 | | | | | -0.072890023 | | | | | 0.233802825 | | | | 0.888135517 | | 0.417702194 |  |  |  |  |
| PtrPPR485 | | | | E1 | | | NA | | -0.168251349 | | | | | -0.513462615 | | | | | 0.634340755 | | | | NA | | NA |  |  |  |  |
| PtrPPR486 | | | | P | | | -0.060114337 | | -0.530821428 | | | | | -1.735855036 | | | | | -0.240128363 | | | | -0.151392847 | | 0.869770424 |  |  |  |  |
| PtrPPR487 | | | | P | | | -0.859558643 | | 0.221782145 | | | | | -0.749803854 | | | | | -0.321715898 | | | | -1.28787214 | | 0.130149678 |  |  |  |  |
| PtrPPR488 | | | | P | | | 1.551320375 | | -1.878744731 | | | | | 0.945969004 | | | | | 0.049378254 | | | | 1.13811377 | | 1.758739112 |  |  |  |  |
| PtrPPR489 | | | | P | | | -0.249775557 | | 0.597693313 | | | | | -0.137020361 | | | | | 0.525816298 | | | | 0.258408003 | | -0.255760458 |  |  |  |  |
| PtrPPR490 | | | | E1 | | | -0.448679625 | | 0.821694987 | | | | | -1.276423418 | | | | | 0.508809873 | | | | -0.539958135 | | 0.758739112 |  |  |  |  |
| PtrPPR491 | | | | P | | | NA | | NA | | | | | NA | | | | | NA | | | | NA | | -9.910831283 |  |  |  |  |
| PtrPPR492 | | | | P | | | 0.518898897 | | 1.038793108 | | | | | 0.843158198 | | | | | 0.101845674 | | | | 0.842657886 | | 0.159277041 |  |  |  |  |
| PtrPPR493 | | | | P | | | 0.463857534 | | 1.677648617 | | | | | 0.636113741 | | | | | 0.916111723 | | | | 0.908631924 | | 1.021773517 |  |  |  |  |
| PtrPPR494 | | | | DYW | | | -1.112999918 | | 0.643933491 | | | | | 0.138614082 | | | | | -0.100368865 | | | | -0.426670849 | | 0.128688721 |  |  |  |  |
| PtrPPR495 | | | | DWY | | | NA | | NA | | | | | NA | | | | | NA | | | | NA | | NA |  |  |  |  |
| PtrPPR496 | | | | E2 | | | 0.22939228 | | 0.121255269 | | | | | 0.723576582 | | | | | 0.634340755 | | | | 0.13811377 | | -0.978226483 |  |  |  |  |
| PtrPPR497 | | | | P | | | 1.853883145 | | 1.394273763 | | | | | 0.641114422 | | | | | 1.219303255 | | | | 1.762604635 | | 1.061301882 |  |  |  |  |
| PtrPPR498 | | | | P | | | -0.77060772 | | -1.200672826 | | | | | -0.861385918 | | | | | 0.31241266 | | | | -1.598851824 | | 0.758739112 |  |  |  |  |
| PtrPPR499 | | | | DYW | | | 0.577315583 | | 0.61601996 | | | | | -0.861385918 | | | | | -0.410053365 | | | | 0.322538341 | | 1.369696821 |  |  |  |  |
| PtrPPR500 | | | | P | | | -0.062788472 | | -0.952745313 | | | | | -0.861385918 | | | | | -0.31835353 | | | | 0.571073177 | | 0.26144393 |  |  |  |  |
| PtrPPR501 | | | | P | | | -0.491675687 | | 0.529282087 | | | | | -0.615773959 | | | | | -0.766996673 | | | | -0.721116637 | | 0.167203957 |  |  |  |  |
| PtrPPR502 | | | | P | | | 0.551320375 | | 0.29118027 | | | | | 0.308539083 | | | | | -0.143266824 | | | | -0.539958135 | | 0.53634669 |  |  |  |  |
| PtrPPR503 | | | | E2 | | | -0.225173584 | | -1.230217102 | | | | | -1.485876783 | | | | | -0.378715398 | | | | -0.86188623 | | 0.918680024 |  |  |  |  |
| PtrPPR504 | | | | P | | | 0.477319793 | | -0.70881973 | | | | | -0.861385918 | | | | | 0.634340755 | | | | 0.386041283 | | 0.68473853 |  |  |  |  |
| PtrPPR505 | | | | DYW | | | -0.225173584 | | -0.628766478 | | | | | -0.263484362 | | | | | -0.030792095 | | | | 0.45222236 | | 0.076221301 |  |  |  |  |
| PtrPPR506 | | | | P | | | -0.27017873 | | 0.740983188 | | | | | 0.138614082 | | | | | 0.183679346 | | | | 0.757841689 | | -0.164639607 |  |  |  |  |
| PtrPPR507 | | | | PLS | | | NA | | NA | | | | | NA | | | | | NA | | | | NA | | NA |  |  |  |  |
| PtrPPR508 | | | | P | | | 0.22939228 | | 0.928610191 | | | | | 0.138614082 | | | | | 1.297305767 | | | | 0.308038771 | | -0.300154577 |  |  |  |  |
| PtrPPR509 | | | | E2 | | | -0.714024192 | | 0.335380074 | | | | | -0.257314595 | | | | | 0.105961782 | | | | 0.742185093 | | 0.021773517 |  |  |  |  |
| PtrPPR510 | | | | E+ | | | -7.659157341 | | 1.706217769 | | | | | 0.138614082 | | | | | 0.049378254 | | | | -7.659157341 | | -7.866776103 |  |  |  |  |
| PtrPPR511 | | | | P | | | 0.366895804 | | -0.113209985 | | | | | 0.276117606 | | | | | 0.251012115 | | | | 0.460041865 | | 0.481205136 |  |  |  |  |
| PtrPPR512 | | | | P | | | NA | | NA | | | | | NA | | | | | NA | | | | NA | | NA |  |  |  |  |
| PtrPPR513 | | | | P | | | NA | | NA | | | | | NA | | | | | NA | | | | NA | | NA |  |  |  |  |
| PtrPPR514 | | | | P | | | 0.368750565 | | 1.008044658 | | | | | -0.501843532 | | | | | 0.349363796 | | | | -0.512991088 | | -0.193096784 |  |  |  |  |
| PtrPPR515 | | | | E+ | | | 0.184998161 | | -0.601210756 | | | | | -2.098425116 | | | | | 0.431248889 | | | | -0.658352836 | | 0.934310676 |  |  |  |  |
| PtrPPR516 | | | | PLS | | | NA | | NA | | | | | NA | | | | | NA | | | | NA | | NA |  |  |  |  |
| PtrPPR517 | | | | P | | | 1.22939228 | | 0.706217769 | | | | | 0.138614082 | | | | | 1.049378254 | | | | 1.460041865 | | 0.436811017 |  |  |  |  |
| PtrPPR518 | | | | DYW | | | 0.714819107 | | 0.606682096 | | | | | 0.624040909 | | | | | 1.534805081 | | | | 1.816185675 | | -0.170871561 |  |  |  |  |
| PtrPPR519 | | | | P | | | 1.979414027 | | 0.469178572 | | | | | -0.150892535 | | | | | 0.589946635 | | | | 0.486037073 | | 1.143764042 |  |  |  |  |
| PtrPPR520 | | | | P | | | NA | | NA | | | | | NA | | | | | NA | | | | NA | | NA |  |  |  |  |
| PtrPPR521 | | | | P | | | NA | | NA | | | | | NA | | | | | NA | | | | NA | | NA |  |  |  |  |
| PtrPPR522 | | | | DYW | | | -0.577962642 | | -1.101137153 | | | | | -1.66874084 | | | | | 0.701454951 | | | | -0.347313057 | | -0.300154577 |  |  |  |  |
| PtrPPR523 | | | | P | | | -0.24594573 | | -0.904279823 | | | | | -1.050419743 | | | | | 0.916927958 | | | | 0.590625974 | | 0.209400521 |  |  |  |  |
| PtrPPR524 | | | | E+ | | | 1.22939228 | | 0.121255269 | | | | | -0.861385918 | | | | | 0.634340755 | | | | -8.585156759 | | 0.436811017 |  |  |  |  |
| PtrPPR525 | | | | PLS | | | NA | | NA | | | | | NA | | | | | NA | | | | NA | | NA |  |  |  |  |
| PtrPPR526 | | | | DYW | | | 0.492426686 | | 0.606682096 | | | | | -0.183314013 | | | | | 0.897375161 | | | | -0.598851824 | | -0.978226483 |  |  |  |  |
| PtrPPR527 | | | | DYW | | | -0.285180893 | | 0.886790015 | | | | | -0.013389012 | | | | | -0.102624839 | | | | 0.13811377 | | -0.630303179 |  |  |  |  |
| PtrPPR528 | | | | P | | | 0.492426686 | | 0.121255269 | | | | | 0.401648488 | | | | | 0.31241266 | | | | 0.13811377 | | 1.021773517 |  |  |  |  |
| PtrPPR529 | | | | P | | | -0.050715639 | | -0.381245072 | | | | | -0.363886259 | | | | | 0.546877914 | | | | 1.336053147 | | -0.492799655 |  |  |  |  |
| PtrPPR530 | | | | DYW | | | 0.399317281 | | -0.071389809 | | | | | -0.861385918 | | | | | -0.628693651 | | | | -0.539958135 | | 0.869770424 |  |  |  |  |
| PtrPPR531 | | | | DYW | | | 2.551320375 | | 3.20871811 | | | | | 1.723576582 | | | | | 2.749817972 | | | | 1.13811377 | | 1.343701612 |  |  |  |  |
| PtrPPR532 | | | | DYW | | | -0.577962642 | | 0.220790942 | | | | | -3.66874084 | | | | | -0.173014167 | | | | -1.084278652 | | -0.678666201 |  |  |  |  |
| PtrPPR533 | | | | DYW | | | -0.230039339 | | -0.530821428 | | | | | -1.735855036 | | | | | 0.759871637 | | | | -0.321317849 | | 0.214418595 |  |  |  |  |
| PtrPPR534 | | | | P | | | -1.220085865 | | 1.06236158 | | | | | -0.061023583 | | | | | -0.170796342 | | | | -0.857453984 | | -0.475726142 |  |  |  |  |
| PtrPPR535 | | | | P | | | 0.551320375 | | 0.706217769 | | | | | 0.460542177 | | | | | 0.634340755 | | | | 0.553151269 | | 0.758739112 |  |  |  |  |
| PtrPPR536 | | | | DYW | | | -0.851527715 | | -0.660104445 | | | | | -0.998889442 | | | | | -0.208419503 | | | | -1.058283443 | | -0.13576776 |  |  |  |  |
| PtrPPR537 | | | | P | | | NA | | 0.010223956 | | | | | 0.38372658 | | | | | -0.00506953 | | | | NA | | NA |  |  |  |  |
| PtrPPR538 | | | | P | | | 0.981464766 | | 0.780218351 | | | | | -0.787385337 | | | | | 0.84584486 | | | | 0.282503679 | | 0.699845423 |  |  |  |  |
| PtrPPR539 | | | | P | | | -0.056009939 | | 0.262117804 | | | | | 0.138614082 | | | | | 0.49189649 | | | | 0.525136893 | | -0.836207478 |  |  |  |  |
| PtrPPR540 | | | | DYW | | | 1.644429779 | | 2.651769985 | | | | | 1.428120699 | | | | | 1.338884871 | | | | 1.290116863 | | -0.602717348 |  |  |  |  |
| PtrPPR541 | | | | P | | | 0.22939228 | | 2.121255269 | | | | | -9.840621314 | | | | | 0.049378254 | | | | -10.09972993 | | -0.978226483 |  |  |  |  |
| PtrPPR542 | | | | P | | | -1.821233793 | | -0.566800725 | | | | | -1.912011991 | | | | | -0.63867774 | | | | -2.719867225 | | 0.507200345 |  |  |  |  |
| PtrPPR543 | | | | E+ | | | 7.621903961 | | 9.254658332 | | | | | 7.272017145 | | | | | 8.767743818 | | | | 7.530625451 | | 0.021773517 |  |  |  |  |
| PtrPPR544 | | | | P | | | 0.22939228 | | -0.030747825 | | | | | 0.138614082 | | | | | 0.427889877 | | | | 0.72307627 | | 0.758739112 |  |  |  |  |
| PtrPPR545 | | | | P | | | -0.645076838 | | -0.431285754 | | | | | -0.233354696 | | | | | -0.322590523 | | | | 1.655962075 | | 0.147304399 |  |  |  |  |
| PtrPPR546 | | | | P | | | NA | | 1.325613767 | | | | | -0.255664857 | | | | | 0.2807038 | | | | NA | | NA |  |  |  |  |
| PtrPPR547 | | | | PLS | | | -0.355570221 | | -0.463707232 | | | | | -1.446348419 | | | | | -0.535584247 | | | | -2.446848731 | | 0.436811017 |  |  |  |  |
| PtrPPR548 | | | | P | | | NA | | 0.606682096 | | | | | -0.346812745 | | | | | 0.474684089 | | | | NA | | NA |  |  |  |  |
| PtrPPR549 | | | | E+ | | | NA | | NA | | | | | NA | | | | | NA | | | | NA | | NA |  |  |  |  |
| PtrPPR550 | | | | P | | | -11.64119079 | | 0.121255269 | | | | | -11.38208218 | | | | | -11.38208218 | | | | -11.64119079 | | NA |  |  |  |  |
| PtrPPR551 | | | | E2 | | | NA | | NA | | | | | NA | | | | | NA | | | | NA | | NA |  |  |  |  |
| PtrPPR552 | | | | P | | | 0.202920069 | | 0.889359375 | | | | | 0.605181882 | | | | | 0.182901927 | | | | -0.140745603 | | -0.260428299 |  |  |  |  |
| PtrPPR553 | | | | P | | | 0.563811319 | | -0.009989265 | | | | | 0.258908315 | | | | | 0.613279139 | | | | 0.614551814 | | 0.557826418 |  |  |  |  |
| PtrPPR554 | | | | P | | | -0.955032291 | | 0.335380074 | | | | | -1.920279607 | | | | | 0.449916184 | | | | -0.335817419 | | -0.688719865 |  |  |  |  |
| PtrPPR555 | | | | E2 | | | -1.355570221 | | 0.021719595 | | | | | -0.768276514 | | | | | 0.786343848 | | | | 0.231223174 | | -1.826223389 |  |  |  |  |
| PtrPPR556 | | | | DYW | | | 1.451784701 | | 1.34364769 | | | | | 0.138614082 | | | | | 1.271770675 | | | | 1.553151269 | | 0.244165939 |  |  |  |  |
| PtrPPR557 | | | | P | | | 1.857423503 | | 0.831748651 | | | | | 1.202744419 | | | | | -0.08812527 | | | | 0.379121869 | | 0.939311357 |  |  |  |  |
| PtrPPR558 | | | | E2 | | | 0.759906997 | | 1.199257781 | | | | | -0.223955998 | | | | | 1.271770675 | | | | 1.05565161 | | 0.137250735 |  |  |  |  |
| PtrPPR559 | | | | E1 | | | -0.507573314 | | -0.200672826 | | | | | -2.183314013 | | | | | 0.727450159 | | | | -0.598851824 | | 1.606736018 |  |  |  |  |
| PtrPPR560 | | | | DYW | | | -0.507573314 | | -1.200672826 | | | | | -0.183314013 | | | | | -1.272549841 | | | | -0.183814325 | | -0.978226483 |  |  |  |  |
| PtrPPR561 | | | | P | | | 0.692364256 | | 0.466390755 | | | | | 0.214562935 | | | | | 0.363486844 | | | | 0.250588499 | | 0.694198859 |  |  |  |  |
| PtrPPR562 | | | | PLS | | | NA | | NA | | | | | NA | | | | | NA | | | | NA | | NA |  |  |  |  |
| PtrPPR563 | | | | DYW | | | -0.481101103 | | -0.348230015 | | | | | -2.03131092 | | | | | 0.201381347 | | | | -0.86188623 | | 0.673850214 |  |  |  |  |
| PtrPPR564 | | | | P | | | -0.574883249 | | -0.599362331 | | | | | -1.003830183 | | | | | -0.206276621 | | | | -0.58250383 | | 0.133666397 |  |  |  |  |
| PtrPPR565 | | | | P | | | 1.103861398 | | 1.536292768 | | | | | 1.254091299 | | | | | 1.164855471 | | | | 1.945468692 | | 0.021773517 |  |  |  |  |
| PtrPPR566 | | | | P | | | 0.528952562 | | 0.880247169 | | | | | 0.608099365 | | | | | 0.872500492 | | | | 1.344564647 | | 0.562341899 |  |  |  |  |
| PtrPPR567 | | | | P | | | -0.398638943 | | -0.381245072 | | | | | -1.626920665 | | | | | -0.230729665 | | | | 0.751090647 | | -0.42568546 |  |  |  |  |
| PtrPPR568 | | | | P | | | 1.551320375 | | 0.928610191 | | | | | -0.276423418 | | | | | 1.219303255 | | | | 1.597545388 | | 0.53634669 |  |  |  |  |
| PtrPPR569 | | | | P | | | -1.03113527 | | -1.817344187 | | | | | -1.848894974 | | | | | -0.241522945 | | | | -0.800485686 | | -0.037120172 |  |  |  |  |
| PtrPPR570 | | | | DYW | | | -0.77060772 | | 0.858220863 | | | | | -0.223955998 | | | | | 0.201381347 | | | | -0.22445631 | | -0.508741199 |  |  |  |  |
| PtrPPR571 | | | | E+ | | | -1.577962642 | | 0.313900347 | | | | | -2.66874084 | | | | | 0.242023332 | | | | 0.13811377 | | -0.978226483 |  |  |  |  |
| PtrPPR572 | | | | E2 | | | NA | | NA | | | | | NA | | | | | NA | | | | NA | | NA |  |  |  |  |
| PtrPPR573 | | | | P | | | -0.086109546 | | -0.071389809 | | | | | -0.311188836 | | | | | 0.074913346 | | | | 0.439283304 | | -0.10108323 |  |  |  |  |
| PtrPPR574 | | | | P | | | 1.22939228 | | 1.883885746 | | | | | 0.926460845 | | | | | 1.313439855 | | | | 1.1503921 | | 0.345243777 |  |  |  |  |
| PtrPPR575 | | | | E+ | | | 1.22939228 | | 0.121255269 | | | | | 1.138614082 | | | | | 1.371306349 | | | | 1.13811377 | | -0.300154577 |  |  |  |  |
| PtrPPR576 | | | | E2 | | | 0.814354781 | | 0.706217769 | | | | | 0.138614082 | | | | | 1.049378254 | | | | 1.945468692 | | 1.606736018 |  |  |  |  |
| PtrPPR577 | | | | P | | | 0.662351687 | | 0.384289674 | | | | | -0.183314013 | | | | | 0.049378254 | | | | 0.401148176 | | -0.177535291 |  |  |  |  |
| PtrPPR578 | | | | PLS | | | NA | | NA | | | | | NA | | | | | NA | | | | NA | | NA |  |  |  |  |
| PtrPPR579 | | | | P | | | NA | | NA | | | | | NA | | | | | NA | | | | NA | | NA |  |  |  |  |
| PtrPPR580 | | | | PLS | | | NA | | NA | | | | | NA | | | | | 0.464415753 | | | | NA | | NA |  |  |  |  |
| PtrPPR581 | | | | E+ | | | -1.355570221 | | 0.858220863 | | | | | -1.446348419 | | | | | NA | | | | 0.13811377 | | -1.563188983 |  |  |  |  |
| PtrPPR582 | | | | PLS | | | 1.22939228 | | -8.963478062 | | | | | 0.138614082 | | | | | 1.049378254 | | | | -9.22258668 | | 0.021773517 |  |  |  |  |
| PtrPPR583 | | | | P | | | 0.55877618 | | 2.09865384 | | | | | 1.151636563 | | | | | 1.040003984 | | | | 0.46749767 | | -0.062010095 |  |  |  |  |
| PtrPPR584 | | | | P | | | 1.151389768 | | 1.23087976 | | | | | 0.283003991 | | | | | 0.608805663 | | | | -0.302458822 | | 1.021773517 |  |  |  |  |
| PtrPPR585 | | | | DYW | | | 0.22939228 | | 0.928610191 | | | | | 0.723576582 | | | | | 1.634340755 | | | | 0.72307627 | | 1.021773517 |  |  |  |  |
| PtrPPR586 | | | | DYW | | | 0.381395373 | | 1.080613284 | | | | | 0.669128798 | | | | | 0.833649563 | | | | 0.922385079 | | 0.095774099 |  |  |  |  |
| PtrPPR587 | | | | DYW | | | 0.507693442 | | 1.473557013 | | | | | 0.73377235 | | | | | 0.852341407 | | | | -0.131072863 | | -0.211425659 |  |  |  |  |
| PtrPPR588 | | | | DWY | | | NA | | NA | | | | | NA | | | | | NA | | | | NA | | NA |  |  |  |  |
| PtrPPR589 | | | | P | | | 0.475552867 | | 0.18684361 | | | | | -0.243256554 | | | | | 0.146675455 | | | | 0.355705205 | | 0.372270764 |  |  |  |  |
| PtrPPR590 | | | | P | | | 0.527791839 | | 0.307588027 | | | | | 0.526884671 | | | | | -2.083888277 | | | | -0.379493463 | | 1.679715463 |  |  |  |  |
| PtrPPR591 | | | | DYW | | | -0.77060772 | | -0.878744731 | | | | | 0.138614082 | | | | | 0.634340755 | | | | 0.13811377 | | -0.563188983 |  |  |  |  |
| PtrPPR592 | | | | DYW | | | -0.600682719 | | 0.765111458 | | | | | -0.054030996 | | | | | 1.136841095 | | | | -0.054531308 | | 0.869770424 |  |  |  |  |
| PtrPPR593 | | | | P | | | 0.085002371 | | -0.271062154 | | | | | -1.253703341 | | | | | 0.11649245 | | | | -0.932275558 | | 0.362810435 |  |  |  |  |
| PtrPPR594 | | | | E2 | | | NA | | NA | | | | | NA | | | | | NA | | | | NA | | NA |  |  |  |  |
| PtrPPR595 | | | | P | | | 1.167991735 | | 1.418935817 | | | | | 0.911203586 | | | | | 0.464415753 | | | | 0.553151269 | | 0.223407379 |  |  |  |  |
| PtrPPR596 | | | | E1 | | | NA | | NA | | | | | NA | | | | | NA | | | | NA | | NA |  |  |  |  |
| PtrPPR597 | | | | P | | | 8.198998219 | | 1.648502271 | | | | | -0.041958164 | | | | | 0.868806008 | | | | 9.692682209 | | 0.021773517 |  |  |  |  |
| PtrPPR598 | | | | PLS | | | 0.22939228 | | 0.121255269 | | | | | 0.138614082 | | | | | 0.049378254 | | | | -8.125725141 | | 0.021773517 |  |  |  |  |
| PtrPPR599 | | | | DYW | | | 1.036747202 | | -0.878744731 | | | | | -0.861385918 | | | | | 1.749817972 | | | | 0.460041865 | | 0.829128439 |  |  |  |  |
| PtrPPR600 | | | | P | | | 1.006999859 | | -0.101137153 | | | | | -0.420813327 | | | | | 0.885879522 | | | | 0.540212213 | | 0.799381096 |  |  |  |  |
| PtrPPR601 | | | | P | | | 0.316197107 | | 0.440944891 | | | | | -0.62468566 | | | | | -0.275348474 | | | | -2.229257296 | | 0.131120914 |  |  |  |  |
| PtrPPR602 | | | | P | | | 0.509500199 | | 0.635828441 | | | | | -0.08377834 | | | | | 0.826985833 | | | | 0.854320804 | | -0.138691155 |  |  |  |  |
| PtrPPR603 | | | | P | | | -0.492401003 | | -0.800175679 | | | | | -3.312765265 | | | | | -1.667982782 | | | | -1.38881661 | | 0.842507469 |  |  |  |  |
| PtrPPR604 | | | | P | | | 0.607903903 | | -0.200672826 | | | | | 0.723576582 | | | | | -0.102624839 | | | | -0.376459403 | | 0.137250735 |  |  |  |  |
| PtrPPR605 | | | | P | | | 0.451784701 | | 0.809311262 | | | | | 0.138614082 | | | | | 0.049378254 | | | | 1.13811377 | | 0.326628099 |  |  |  |  |
| PtrPPR606 | | | | p | | | 1.814354781 | | 1.369182782 | | | | | 0.811039424 | | | | | 0.721803596 | | | | 2.945468692 | | -0.130229576 |  |  |  |  |
| PtrPPR607 | | | | P | | | NA | | NA | | | | | NA | | | | | NA | | | | NA | | NA |  |  |  |  |
| PtrPPR608 | | | | E+ | | | NA | | NA | | | | | NA | | | | | NA | | | | NA | | NA |  |  |  |  |
| PtrPPR609 | | | | PLS | | | -0.033642126 | | 1.180148958 | | | | | -0.638993497 | | | | | 0.634340755 | | | | 0.253590987 | | 0.173776611 |  |  |  |  |
| PtrPPR610 | | | | P | | | 1.129856606 | | 0.214364673 | | | | | 0.624040909 | | | | | 0.464415753 | | | | 0.75478513 | | 0.305566483 |  |  |  |  |
| PtrPPR611 | | | | PLS | | | 1.22939228 | | -0.878744731 | | | | | 1.460542177 | | | | | 1.371306349 | | | | 2.945468692 | | -0.563188983 |  |  |  |  |
| PtrPPR612 | | | | P | | | NA | | NA | | | | | NA | | | | | NA | | | | NA | | NA |  |  |  |  |
| PtrPPR613 | | | | P | | | NA | | NA | | | | | NA | | | | | NA | | | | NA | | NA |  |  |  |  |
| PtrPPR614 | | | | P | | | 0.14693012 | | -0.241314811 | | | | | -0.223955998 | | | | | 0.579892971 | | | | 0.05565161 | | -1.414325597 |  |  |  |  |
| PtrPPR615 | | | | E2 | | | NA | | NA | | | | | NA | | | | | NA | | | | NA | | NA |  |  |  |  |
| PtrPPR616 | | | | P | | | 1.644429779 | | 2.784220281 | | | | | 2.460542177 | | | | | 1.786343848 | | | | 2.875079364 | | -0.148151484 |  |  |  |  |
| PtrPPR617 | | | | P | | | NA | | NA | | | | | NA | | | | | NA | | | | NA | | NA |  |  |  |  |
| PtrPPR618 | | | | P | | | 1.22939228 | | 1.121255269 | | | | | 0.460542177 | | | | | 0.371306349 | | | | 0.945468692 | | 0.021773517 |  |  |  |  |
| PtrPPR619 | | | | DYW | | | 0.451784701 | | 0.858220863 | | | | | 0.138614082 | | | | | 0.271770675 | | | | 0.253590987 | | 1.021773517 |  |  |  |  |
| PtrPPR620 | | | | E+ | | | -3.677498316 | | NA | | | | | -1.446348419 | | | | | -0.857512342 | | | | 0.401148176 | | -2.978226483 |  |  |  |  |
| PtrPPR621 | | | | PLS | | | NA | | NA | | | | | NA | | | | | NA | | | | NA | | NA |  |  |  |  |
| PtrPPR622 | | | | P | | | -0.355570221 | | -1.463707232 | | | | | -1.446348419 | | | | | 0.049378254 | | | | 0.13811377 | | -0.563188983 |  |  |  |  |
| PtrPPR623 | | | | E1 | | | NA | | 8.006730819 | | | | | 8.024089632 | | | | | 7.934853804 | | | | 8.282697938 | | -8.35220293 |  |  |  |  |
| PtrPPR624 | | | | P | | | 1.22939228 | | -8.037478644 | | | | | -8.037478644 | | | | | 1.634340755 | | | | 0.13811377 | | 9.525979541 |  |  |  |  |
| PtrPPR625 | | | | P | | | NA | | NA | | | | | NA | | | | | NA | | | | NA | | NA |  |  |  |  |
| PtrPPR626 | | | | PLS | | | -0.77060772 | | -0.141779137 | | | | | -0.124420324 | | | | | 0.464415753 | | | | -0.86188623 | | -0.393263982 |  |  |  |  |
| **Supplemental Table 5 . The primers of *PtrPPR* genes for qRT-PCR** | | | | | | | | | | | | | | | | | | | |  |  |  |  |  |  |  |  |  |  |
| **Gene name** | | **Forward primers** | | | | | | | | | **Reverse primers** | | | | | | | | |  |  |  |  |  |  |  |  |  |  |
| *PtrPPR5* | | 5'-TTGCTACCTTCAAGCCATAC-3' | | | | | | | | | 5'-CAGCCTCAACGAGTGAATG-3' | | | | | | | | |  |  |  |  |  |  |  |  |  |  |
| *PtrPPR8* | | 5'-GAGCCAGATGAGATGAC-3' | | | | | | | | | 5'-CTCGCAACATAGACATCG-3' | | | | | | | | |  |  |  |  |  |  |  |  |  |  |
| *PtrPPR28* | | 5'-AGAGGAGGCTTGTTTG-3' | | | | | | | | | 5'-TCATTGTTCGCATCAC-3' | | | | | | | | |  |  |  |  |  |  |  |  |  |  |
| *PtrPPR30* | | 5'-TGGCTGAGCGAGGAGT-3' | | | | | | | | | 5'-TGACGACGTTGGGTTT-3' | | | | | | | | |  |  |  |  |  |  |  |  |  |  |
| *PtrPPR41* | | 5'-ACCAACAACGCCCACT-3' | | | | | | | | | 5'-CTTGCCACCGCTACTT-3' | | | | | | | | |  |  |  |  |  |  |  |  |  |  |
| *PtrPPR119* | | 5'-AACTCTTCCACCTCAG-3' | | | | | | | | | 5'-TGGTGGGTATGGGATT-3' | | | | | | | | |  |  |  |  |  |  |  |  |  |  |
| *PtrPPR121* | | 5'-TGTTCGACCTTGCCATCC-3' | | | | | | | | | 5'-GACATTCCACCCATCT-3' | | | | | | | | |  |  |  |  |  |  |  |  |  |  |
| *PtrPPR185* | | 5'-CGGGACATTCCACCAG-3' | | | | | | | | | 5'-TCTGGCTCGGCACCTT-3' | | | | | | | | |  |  |  |  |  |  |  |  |  |  |
| *PtrPPR257* | | 5'-CCCCTTTGATTACTCCC-3' | | | | | | | | | 5'-ACTAACCTGCCCGTCT-3' | | | | | | | | |  |  |  |  |  |  |  |  |  |  |
| *PtrPPR275* | | 5'-AAGTGAAGCCCAGGAG-3' | | | | | | | | | 5'-TCAACTCCAGCCAAGC-3' | | | | | | | | |  |  |  |  |  |  |  |  |  |  |
| *PtrPPR277* | | 5'-GCAGCAGTAGCCGTAA-3' | | | | | | | | | 5'-AAGCCATTGATAATCGTAG-3' | | | | | | | | |  |  |  |  |  |  |  |  |  |  |
| *PtrPPR431* | | 5'-TCAAGCCAGGTGTATC-3' | | | | | | | | | 5'-TTGACTTCTCATTTCG-3' | | | | | | | | |  |  |  |  |  |  |  |  |  |  |
| *PtrPPR481* | | 5'-TTTGCCAGGTTTAGAG -3' | | | | | | | | | 5'-GCCACATCATGCCAGT-3' | | | | | | | | |  |  |  |  |  |  |  |  |  |  |
| *PtrPPR540* | | 5'-GGACGGCGACAATAAG-3' | | | | | | | | | 5'-CATCCCATCAAAGACG-3' | | | | | | | | |  |  |  |  |  |  |  |  |  |  |
| *PtrPPR574* | | 5'-AAAGGGCAAGGAAGGACA-3' | | | | | | | | | 5'-CCATTTCGGTGGTGGATA-3' | | | | | | | | |  |  |  |  |  |  |  |  |  |  |
| *PtrPPR583* | | 5'-AGTTCCAAAGGGCAATC-3' | | | | | | | | | 5'-TCAAGGCAGCCAAGAG-3' | | | | | | | | |  |  |  |  |  |  |  |  |  |  |
| *PtrPPR587* | | 5'-CTCCAGTATGCTCGTC-3' | | | | | | | | | 5'-TAACCTCATTAGGCTTC-3' | | | | | | | | |  |  |  |  |  |  |  |  |  |  |
| **Supplemental Table 5 . The primers of *PtrPPR* genes for qRT-PCR** | | | | | | | | | | | | | | | | | | | |  |  |  |  |  |  |  |  |  |  |
| **Gene name** | | **Forward primers** | | | | | | | | | **Reverse primers** | | | | | | | | |  |  |  |  |  |  |  |  |  |  |
| *PtrPPR5* | | 5'-TTGCTACCTTCAAGCCATAC-3' | | | | | | | | | 5'-CAGCCTCAACGAGTGAATG-3' | | | | | | | | |  |  |  |  |  |  |  |  |  |  |
| *PtrPPR8* | | 5'-GAGCCAGATGAGATGAC-3' | | | | | | | | | 5'-CTCGCAACATAGACATCG-3' | | | | | | | | |  |  |  |  |  |  |  |  |  |  |
| *PtrPPR28* | | 5'-AGAGGAGGCTTGTTTG-3' | | | | | | | | | 5'-TCATTGTTCGCATCAC-3' | | | | | | | | |  |  |  |  |  |  |  |  |  |  |
| *PtrPPR30* | | 5'-TGGCTGAGCGAGGAGT-3' | | | | | | | | | 5'-TGACGACGTTGGGTTT-3' | | | | | | | | |  |  |  |  |  |  |  |  |  |  |
| *PtrPPR41* | | 5'-ACCAACAACGCCCACT-3' | | | | | | | | | 5'-CTTGCCACCGCTACTT-3' | | | | | | | | |  |  |  |  |  |  |  |  |  |  |
| *PtrPPR119* | | 5'-AACTCTTCCACCTCAG-3' | | | | | | | | | 5'-TGGTGGGTATGGGATT-3' | | | | | | | | |  |  |  |  |  |  |  |  |  |  |
| *PtrPPR121* | | 5'-TGTTCGACCTTGCCATCC-3' | | | | | | | | | 5'-GACATTCCACCCATCT-3' | | | | | | | | |  |  |  |  |  |  |  |  |  |  |
| *PtrPPR185* | | 5'-CGGGACATTCCACCAG-3' | | | | | | | | | 5'-TCTGGCTCGGCACCTT-3' | | | | | | | | |  |  |  |  |  |  |  |  |  |  |
| *PtrPPR257* | | 5'-CCCCTTTGATTACTCCC-3' | | | | | | | | | 5'-ACTAACCTGCCCGTCT-3' | | | | | | | | |  |  |  |  |  |  |  |  |  |  |
| *PtrPPR275* | | 5'-AAGTGAAGCCCAGGAG-3' | | | | | | | | | 5'-TCAACTCCAGCCAAGC-3' | | | | | | | | |  |  |  |  |  |  |  |  |  |  |
| *PtrPPR277* | | 5'-GCAGCAGTAGCCGTAA-3' | | | | | | | | | 5'-AAGCCATTGATAATCGTAG-3' | | | | | | | | |  |  |  |  |  |  |  |  |  |  |
| *PtrPPR431* | | 5'-TCAAGCCAGGTGTATC-3' | | | | | | | | | 5'-TTGACTTCTCATTTCG-3' | | | | | | | | |  |  |  |  |  |  |  |  |  |  |
| *PtrPPR481* | | 5'-TTTGCCAGGTTTAGAG -3' | | | | | | | | | 5'-GCCACATCATGCCAGT-3' | | | | | | | | |  |  |  |  |  |  |  |  |  |  |
| *PtrPPR540* | | 5'-GGACGGCGACAATAAG-3' | | | | | | | | | 5'-CATCCCATCAAAGACG-3' | | | | | | | | |  |  |  |  |  |  |  |  |  |  |
| *PtrPPR574* | | 5'-AAAGGGCAAGGAAGGACA-3' | | | | | | | | | 5'-CCATTTCGGTGGTGGATA-3' | | | | | | | | |  |  |  |  |  |  |  |  |  |  |
| *PtrPPR583* | | 5'-AGTTCCAAAGGGCAATC-3' | | | | | | | | | 5'-TCAAGGCAGCCAAGAG-3' | | | | | | | | |  |  |  |  |  |  |  |  |  |  |
| *PtrPPR587* | | 5'-CTCCAGTATGCTCGTC-3' | | | | | | | | | 5'-TAACCTCATTAGGCTTC-3' | | | | | | | | |  |  |  |  |  |  |  |  |  |  |
